# Reconstruction of circRNA-miRNA-mRNA associated ceRNA networks reveal functional circRNAs in intracerebral hemorrhage

**DOI:** 10.1038/s41598-021-91059-9

**Published:** 2021-06-02

**Authors:** Zhen Liu, Xinran Wu, Zihan Yu, Xiaobo Tang

**Affiliations:** Department of Biopharmaceutical Sciences (State-Province Key Laboratories of Biomedicine-Pharmaceutics of China), College of Pharmacy, Harbin Medical University, 157 Baojian Road, Nangang District, P. O. Box 19, Harbin, 150081 Heilongjiang China

**Keywords:** Genetics, Molecular biology, Neuroscience

## Abstract

Circular RNA (circRNA), a novel class of noncoding RNAs, has been used extensively to complement transcriptome remodeling in the central nervous system, although the genomic coverage provided has rarely been studied in intracerebral hemorrhage (ICH) and is limited and fails to provide a detailed picture of the cerebral transcriptome landscape. Here, we described sequencing-based transcriptome profiling, providing comprehensive analysis of cerebral circRNA, messenger RNA (mRNA) and microRNA (miRNA) expression in ICH rats. In the study, male Sprague–Dawley rats were subjected to ICH, and next-generation sequencing of RNAs isolated from non-hemorrhagic (Sham) and hemorrhagic (ICH) rat brain samples collected 7 (early phase) and 28 (chronic phase) days after insults, was conducted. Bioinformatics analysis was performed to determine miRNA binding sites and gene ontology of circRNAs, target genes of miRNAs, as well as biological functions of mRNAs, altered after ICH. These analyses revealed different expression profiles of circRNAs, mRNAs and miRNAs in day-7 and day-28 ICH groups, respectively, compared with the Sham. In addition, the expression signature of circRNAs was more sensitive to disease progression than that of mRNAs or miRNAs. Further analysis suggested two temporally specific circRNA-miRNA-mRNA networks based on the competitive endogenous RNA theory, which had profound impacts on brain activities after ICH. In summary, these results suggested an important role for circRNAs in the pathogenesis of ICH and in reverse remodeling based on self-protection support, providing deep insights into diverse possibilities for ICH therapy through targeting circRNAs.

## Introduction

Spontaneous intracerebral hemorrhage (ICH) has been a global burden^1^. Transcriptional profiling for coding and noncoding RNAs has been utilized extensively in ICH to gain new insights into complex disease pathways, to identify biomarkers for better diagnostic and prognostic accuracy, and to examine the impact of therapeutic treatments^2–4^. Hence, more comprehensive analyses of these RNAs in ICH may help us to establish effective interventions to mitigate brain damages. The mechanisms of brain damage after ICH are complex and diverse, mainly involving cytotoxicity, excitotoxicity, oxidative stress and inflammatory response, which lead to apoptosis or necrosis as well as survival and proliferation of neurons, glial cells and vascular endothelial cells, etc., and ultimately affect the permeability and integrity of the blood–brain barrier (BBB)^1^. BBB dysfunction is a hallmark of ICH-induced brain injury. Such disruption will contribute to brain edema formation, participate in neuroinflammation through facilitating leukocyte influx, and allow the entry of potentially neuroactive agents into the perihematomal brain, all of which may contribute to brain injury^1^. Thus, finding potential targets to promote cerebrovascular integrity represents a promising therapeutic approach to treat ICH.

Circular RNAs (circRNAs), a heterogeneous group of noncoding transcripts with covalent bonds between head 3’ and tail 5’ ends to cause a circular pattern, are highly conserved and abundantly expressed in the central nervous system (CNS)^5^. Even though circRNAs have been less targeted in previous brain transcriptional profiling studies when compared with messenger RNAs (mRNAs) and microRNAs (miRNAs), there are several circRNAs showing to be functional and involved in specific physiological and pathological processes through competitive endogenous RNA (ceRNA) mechanism^5^. To date, however, circRNAs have never been included in other analyses of the brain transcriptome of ICH except one conducted by Dou et al*.*^6^, which explored the circRNA expression profile and the possible involvement in molecular mechanisms and signaling pathways in acute ICH (within 24 h). Therefore, we sought to determine whether a more comprehensive profiling on cerebral coding (mRNA) and noncoding (circRNA and miRNA) transcriptomes, would provide a more complete transcriptional landscape in ICH, and whether these changes were dynamically regulated following disease progresses.

In the present study, a molecular and bioinformatic pipeline optimized for comprehensive analysis and quantification of cerebral circRNA, mRNA and miRNA expression with next-generation sequencing was developed. The cerebral transcriptional signatures of ICH, on days 7 and 28 post insults, were analyzed and compared with those subjected to sham operations, respectively. These analyses revealed that while the coding and noncoding transcriptomes were each dynamically altered with ICH, circRNA expression profile was more sensitive to hemorrhagic stroke attack when compared with the mRNA and miRNA expression profiles. In addition, the pathological expression pattern of circRNAs associated with cerebral hemorrhage improved in response to disease development to a greater extent than that of either mRNAs or miRNAs, suggesting a biological role for circRNAs in ICH-induced brain activities. Finally, leveraging the comprehensive rat cerebral transcriptome data obtained, we demonstrated that novel_circ_0004272-mediated rno-miR-134-3p/ENSRNOT00000082593(Grk3) network on day 7, and novel_circ_0020253-mediated rno-miR-1224/ENSRNOT00000042790(Vegfa) network on day 28, likely were the predominant mechanisms of action of cerebral circRNAs. Taken together, the results presented here provided a transcriptome blueprint to identify novel molecular targets and pathways in ICH, as well as new insights into the mechanisms involved in the brain activities responding to ICH progression.

## Materials and methods

### Animal preparation

Male Sprague–Dawley rats (250–300 g) obtained from Experimental Animal Center of the Second Affiliated Hospital of Harbin Medical University in Harbin, China, were housed in an environment with a natural 12-h light–dark cycle. The environmental temperature was maintained at 25 °C and the humidity was maintained at 50%. All rats had free access to food and water pre- and post-surgery. The present study was approved by the *Animal Care and Use Committee of Harbin Medical University* (2017102) and all experiments were performed in accordance with relevant guidelines and regulations detailed in the *NIH Guide for the Care and Use of Laboratory Animals* and *Animal Research: Reporting of *In Vivo* Experiments*. All experiments following were performed by experimenters blinded to the identity of the groups.

### Induction of experimental ICH

The animal ICH model was conducted as we previously reported^7^. Briefly, the rat was intraperitoneally anesthetized with 1% pentobarbital (35 mg/kg) and was immobilized in a stereotactic apparatus frame. Then the autologous whole blood of 100 μL collected from the tail was injected into the right brain striatum (0.7 mm anterior and 3.0 mm lateral of the bregma and 5.5 mm depth) at a rate of 10 μL/min using a 26-gauge needle microinjector. Rats subjected to sham operation were infused with an equivalent volume of normal saline. During the operation, blood glucose, pH, PaO_2_, PaCO_2_, arterial blood pressure and hematocrit were monitored by catheterization of the right femoral artery of rats. The rectal temperature was maintained at approximately 37 °C using a feedback-controlled heating pad.

### RNA profiling analyses

Animals in day-7-Sham, day-7-ICH, day-28-Sham, and day-28-ICH (n = 4 per group) groups were euthanized after 7 and 28 days of surgery, respectively, and the brain tissues ipsilateral to the hematoma were obtained for RNA extraction. For circRNA and mRNA sequencing, total RNA was isolated with TRIzol (Life technologies, USA) using the described method^6^. For miRNA sequencing, total RNA was prepared using a miRNeasy Micro Kit (QIAGEN, Germany) according to the manufacturer’s protocol. RNA degradation and contamination were monitored on 1% agarose gels (Biowest, Spain) for direct visibility. RNA concentration was measured using Qubit RNA Assay Kit in Qubit2.0 Flurometer (Life Technologies, USA). RNA purity was checked using the NanoPhotometer spectrophotometer (IMPLEN, USA). RNA integrity was assessed using the RNA Nano 6000 Assay Kit of the Agilent Bioanalyzer 2100 system (Agilent Technologies, USA). Only RNA samples with a qualified ratio of OD260 to OD280 (1.8–2.1) and an RNA integrity number of at least 8 were used for subsequent library construction and sequencing. RNA library preparation and RNA sequencing were performed by Novogene Technology Co., Ltd. (Beijing, China).

### Library preparation for circRNA and mRNA sequencing and data analyses

A total amount of 5 μg RNA per sample was used as input material for the RNA sample preparations. Firstly, ribosomal RNA (rRNA) was removed by Ribo-Zero rRNA Removal Kit (Epicentre, USA). Subsequently, sequencing libraries were generated using NEBNext Ultra Directional RNA Library Prep Kit for Illumina (NEB, USA) following manufacturer’s recommendations. The library was purified with AMPure XP system (Beckman Coulter, USA) for double-stranded cDNA fragments of preferentially 150–200 bp in length and the library quality was assessed on the Agilent Bioanalyzer 2100 system. The clustering of the index-coded samples was performed on a cBot Cluster Generation System using TruSeq PE Cluster Kit v3-cBot-HS (Illumina, USA) according to the manufacturer's instructions. After cluster generation, the libraries were sequenced on an Illumina Hiseq 4000 platform (Illumina, USA) and finally 150 bp paired-end reads were generated.

Raw data (raw reads) of FASTQ format were firstly processed through in-house perlscripts, in which clean data (clean reads) were obtained by removing reads containing adapter and ploy-N and with low quality (bases with a Phred value less than 20 account for more than 30% of the total bases) from the raw. For circRNA analysis, the clean reads were aligned to the reference genome with Bowtie^8^ (v2.0.6) and the resulting alignment files were compiled and reconstructed using Cufflinks^9^ (v2.0.0) for novel transcript identification. The classification of mapped reads was detailed in Supplementary Table S1. The circRNAs were detected and identified using CIRI^10^ (v2.0.6) and find_circ^11^ (v1.1). In view of the high false positives in circRNA identification^12^ and according to the position of circRNA on the chromosome, the intersection results of the two software were focused on, and eventually the circRNAs with junction reads no less than 2 in all samples in at least one set of replicates were kept for subsequent analysis. For mRNA analysis, the sequence reads were aligned to the reference genome with HISAT^13^ (v2.0.4), and the mapped reads of each sample were assembled by StringTie^14^ (v1.3.1).

### Library preparation for miRNA sequencing and data analyses

A total amount of 3 μg total RNA per sample was used as input material for the small RNA library. Sequencing libraries were generated using NEBNext Multiplex Small RNA Library Prep Set for Illumina (NEB, USA) following manufacturer's recommendations. Briefly, 3’ and 5’ adapters were sequentially ligated to small RNAs, followed by a reverse transcription reaction to create single-stranded cDNA, which was then PCR amplified and purified on an 8% polyacrylamide gel. DNA fragments corresponding to 140–160 bp were recovered and the library quality was assessed on the Agilent Bioanalyzer 2100 system. The clustering of the index-coded samples was performed on a cBot Cluster Generation System using TruSeq SR Cluster Kit v3-cBot-HS (Illumina, USA) according to the manufacturer’s instructions. After cluster generation, the library preparations were sequenced on an Illumina Hiseq 2500 platform (Illumina, USA) and finally 50 bp single-end reads were generated.

Raw reads of FASTQ format were firstly processed through custom perl and python scripts. Then the clean reads were obtained by removing reads containing ploy-A/T/G/C/N, with 5’ adapter contaminants, without 3’ adapter or the insert tag, and with low quality from the raw. Then, a certain range of 18–26 nt clean reads were mapped to reference sequence by Bowtie without mismatch to analyze their expression and distribution on the reference. The mapped small RNA tags were used to annotate known miRNAs through miRbase^15^ (v22.1), and to align novel miRNAs through miRDeep^16^ (v38) and miREvo^17^ (v1.1).

### Real-time qPCR

Real-time qPCR (qRT-PCR) with SYBER green analysis, conducted as previously reported^18^, was used to validate the expression of the selected circRNAs, mRNAs and miRNAs from RNA sequencing (n = 6 per group). In brief, total RNA isolated from individual brain samples was used for first-strand cDNA synthesis according to the manufacturer's instructions (TOYOBO, Japan). Then the first-strand cDNA was used for PCR by the Applied Biosystems 7300 Real-Time PCR System. The parameter settings were 95 °C denaturation (10 min), 95 °C (15 s), 60 °C (30 s), and 72 °C (30 s), which was repeated for 40 cycles. After amplification, the procedure was performed as follows: 95 °C (15 s), 60 °C (60 s), and 95 °C (15 s). All data were analyzed using the threshold cycle relative quantification (∆∆CT) method. GAPDH and U6 were employed as the endogenous control genes for circRNAs and mRNAs, and miRNAs, respectively.

### Statistical analysis

The read counts of circRNA and miRNA were normalized with TPM^19^ (transcript per million) through the following criteria: Normalized expression level = (mapped read count*1,000,000)/libsize (libsize is the sum of circRNA read count), and the read count of mRNA was normalized with FPKM^19^ (expected number of Fragments Per Kilobase of transcript sequence per Millions base pairs sequenced). Differential expression analysis of two conditions/groups was performed using the DESeq R package^20^ (v1.12.0), and the resulting *P* values were corrected using the Benjamini–Hochberg approach for controlling the false discovery rate. The RNA with an adjusted *P* value less than 0.05 was assigned as differentially expressed. The miRNA target sites in exons of circRNA loci were identified using miRanda^21^ (v3.3a). Prediction of the target genes of miRNAs was performed by miRanda and RNAhybrid^22^ (v2.1.2). Gene Ontology (GO) and Kyoto Encyclopedia of Genes and Genomes (KEGG) pathway enrichment analyses of differentially expressed mRNAs were implemented by the GOseq R package^23^ (v2.12) and KOBAS^24^ (v2.0) software, respectively. MultiExperiment Viewer (v4.9.0) was utilized for preparation of heat-map and hierarchical clustering analyses. Origin (v8.5) was utilized for principal component analysis (PCA). The networks of circRNAs, mRNAs and miRNAs were generated by Cytoscape^25^ (v3.8.2). The analyses of data derived from qRT-PCR were performed with GraphPad Prism (v8.4.3, GraphPad Software Inc., USA) via one-way analysis of variance followed by Newman-Keuls multiple comparison test. The results were shown as “mean ± SEM”, and a two-tailed *P* value less than 0.05 was considered statistically significant.

## Results

### Deep RNA sequencing revealed distinct expression signatures of coding and noncoding RNAs in ICH rat brain

A total of 16 barcoded RNA and small RNA libraries were prepared from rat brain samples, including non-hemorrhagic (Sham-7 and Sham-28) and hemorrhagic (ICH-7 and ICH-28) samples collected 7 and 28 days after insults. From these samples, a total of 367,183,400 read pairs and 274,459,600 reads were generated from RNASeq and miRNASeq experiments, respectively. RNASeq reads first underwent transcriptome reconstruction using Cufflinks, of which 11,620 candidate circRNAs were detected and identified, and 9,625 (82.83%) of which were found to be exonic circRNAs composed of the protein coding exons, 730 (6.28%) were intronic circRNAs derived from intron lariats and 1,265 (10.89%) were intergenic ones that consist of unannotated regions of the gene. Besides, total 28,937 transcripts in RNASeq were aligned to mRNAs. Using the criterion that a miRNA sequence must be detected in ≥ 2 small RNA libraries, miRNASeq reads were mapped to 689 mature miRNAs annotated in miRBase and 229 novel miRNAs.

**Figure 1:**
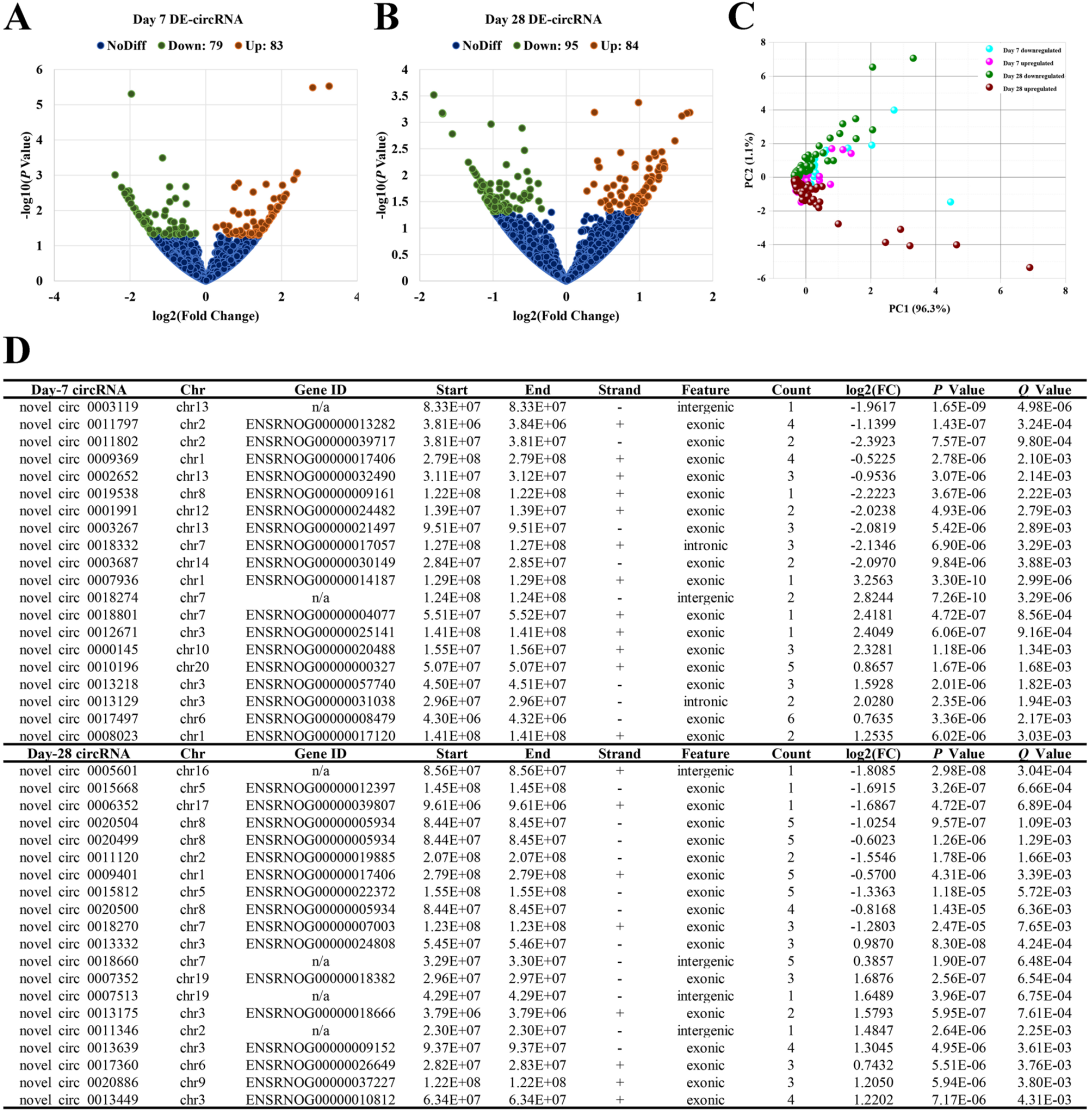
Differences in the circRNA expression profile between Sham and ICH groups. (**A**) Volcano plot showed differential expression of circRNAs between Sham and ICH groups on the 7th day. (**B**) Volcano plot showed differential expression of circRNAs between Sham and ICH groups on the 28th day. (**C**) PCA plot revealed the discrimination between day-7 and day-28 samples. (**D**) Top 10 differentially downregulated and top 10 differentially upregulated circRNAs, in ICH samples, on days 7 and 28, respectively. The horizontal line in the volcano map represented the fold (log_2_ scaled) down or up changes; the vertical line represented a corrected *P* value of 0.05 (-log_10_ scaled); green spots indicated the differentially down-expressed RNAs with statistical significance; red spots indicated the differentially up-expressed RNAs with statistical significance; blue spots indicated RNAs with no statistically significant expression. The variances explained by the principal components chosen were shown as PC1 and PC2.

**Figure 2:**
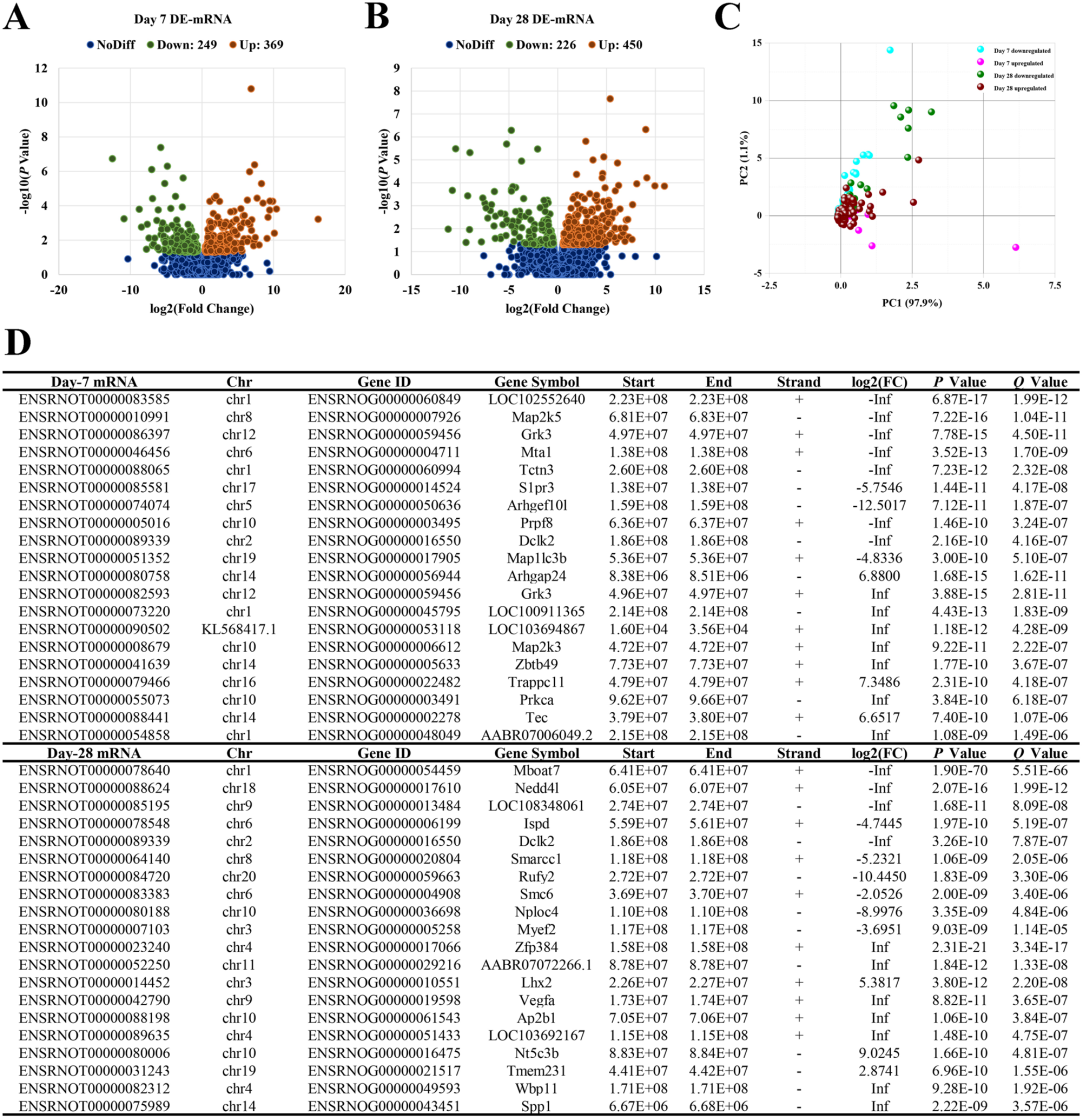
Differences in the mRNA expression profile between Sham and ICH groups. (**A**) Volcano plot showed differential expression of mRNAs between Sham and ICH groups on the 7th day. (**B**) Volcano plot showed differential expression of mRNAs between Sham and ICH groups on the 28th day. (**C**) PCA plot revealed the discrimination between day-7 and day-28 samples. (**D**) Top 10 differentially downregulated and top 10 differentially upregulated mRNAs, in ICH samples, on days 7 and 28, respectively. The horizontal line in the volcano map represented the fold (log_2_ scaled) down or up changes; the vertical line represented a corrected *P* value of 0.05 (-log_10_ scaled); green spots indicated the differentially down-expressed RNAs with statistical significance; red spots indicated the differentially up-expressed RNAs with statistical significance; blue spots indicated RNAs with no statistically significant expression. The variances explained by the principal components chosen were shown as PC1 and PC2.

**Figure 3:**
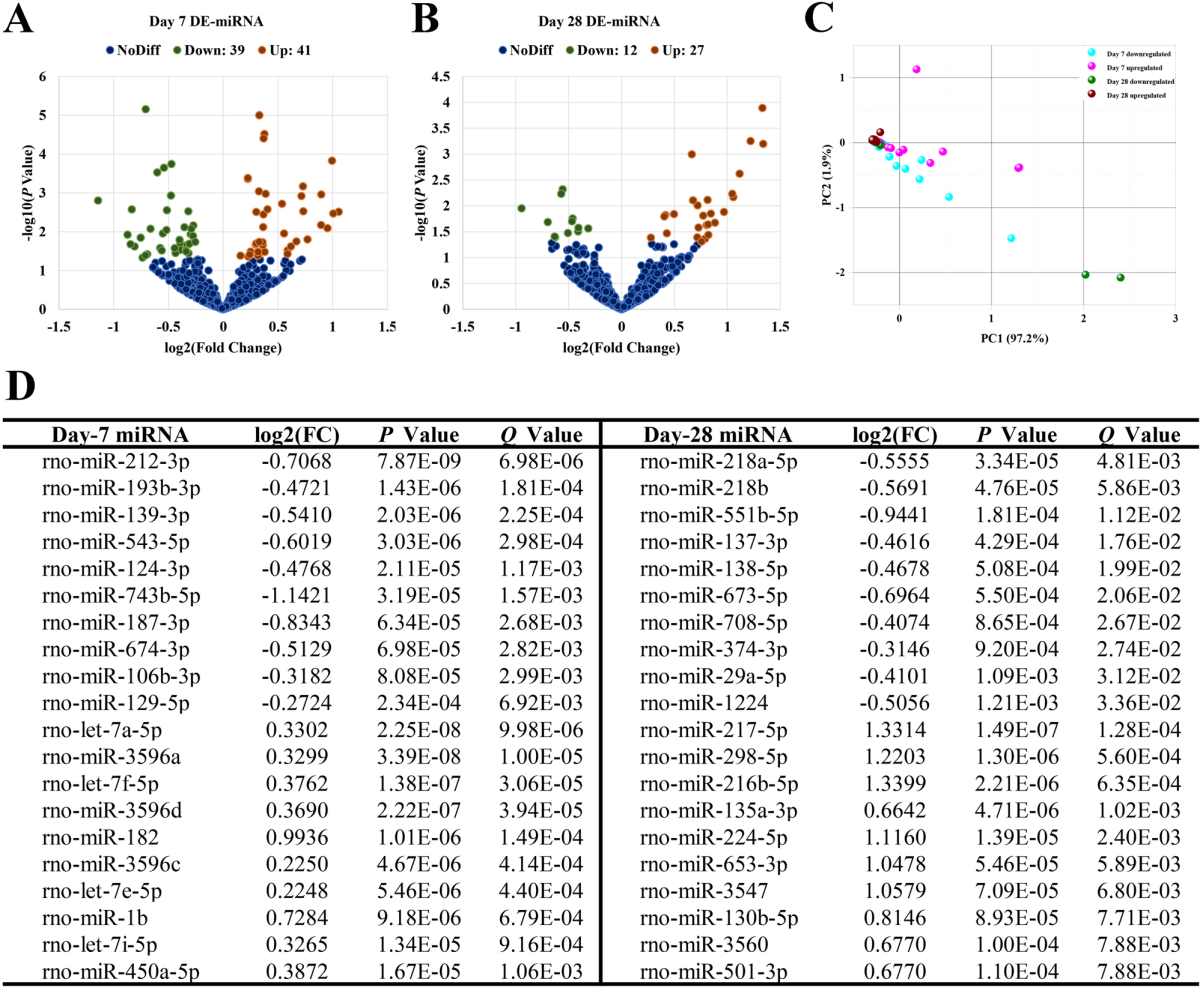
Differences in the miRNA expression profile between Sham and ICH groups. (**A**) Volcano plot showed differential expression of miRNAs between Sham and ICH groups on the 7th day. (**B**) Volcano plot showed differential expression of miRNAs between Sham and ICH groups on the 28th day. (**C**) PCA plot revealed the discrimination between day-7 and day-28 samples. (**D**) Top 10 differentially downregulated and top 10 differentially upregulated miRNAs, in ICH samples, on days 7 and 28, respectively. The horizontal line in the volcano map represented the fold (log_2_ scaled) down or up changes; the vertical line represented a corrected *P* value of 0.05 (-log_10_ scaled); green spots indicated the differentially down-expressed RNAs with statistical significance; red spots indicated the differentially up-expressed RNAs with statistical significance; blue spots indicated RNAs with no statistically significant expression. The variances explained by the principal components chosen were shown as PC1 and PC2.

Volcano and PCA plots showed that the circRNA, mRNA and miRNA expression levels were clearly distinguished between Sham and ICH groups on the 7th and 28th days, respectively. Totally 11,620 circRNA transcripts were identified in the rat brain tissues, including 79 statistically downregulated circRNAs and 83 statistically upregulated circRNAs in ICH compared with Sham on day 7 (Fig. 1A), and 95 statistically downregulated circRNAs and 84 statistically upregulated circRNAs in ICH compared with Sham on day 28 (Fig. 1B). In addition, the variance (97.4%) explained by the principal components chosen distinguished day-7 samples from day-28 ones (Fig. 1C). The detail of these differentially expressed circRNAs in individual samples and the feature of these circRNAs were summarized in Supplementary Table S2 and S3, respectively. The top 10 most significantly up- and downregulated circRNAs in each time point were shown in Fig. 1D. Of the 28,937 mRNAs detected in the rat brain tissues, 249 were found to be statistically downregulated and 369 were statistically upregulated in ICH compared with Sham on day 7 (Fig. 2A), and 226 statistically downregulated and 450 statistically upregulated mRNAs were found in ICH compared with Sham on day 28 (Fig. 2B). Moreover, the variance (99.0%) explained by the principal components revealed a discrimination between day-7 and day-28 samples (Fig. 2C). The detail of these differentially expressed mRNAs was summarized in Supplementary Table S4. The top 10 most significantly up- and downregulated mRNAs in each time point were shown in Fig. 2D. Among the 918 miRNAs detected, there were 39 statistically downregulated and 41 statistically upregulated miRNAs in ICH compared with Sham on day 7 (Fig. 3A), and 12 statistically downregulated and 27 statistically upregulated miRNAs in ICH compared with Sham on day 28 (Fig. 3B). And the variance (99.1%) explained by the principal components distinguished day-7 samples from day-28 ones (Fig. 3C). The detail of the differentially expressed miRNAs in individual samples was summarized in Supplementary Table S5. The top 10 most significantly up- and downregulated miRNAs in each time point were shown in Fig. 3D.

**Figure 4:**
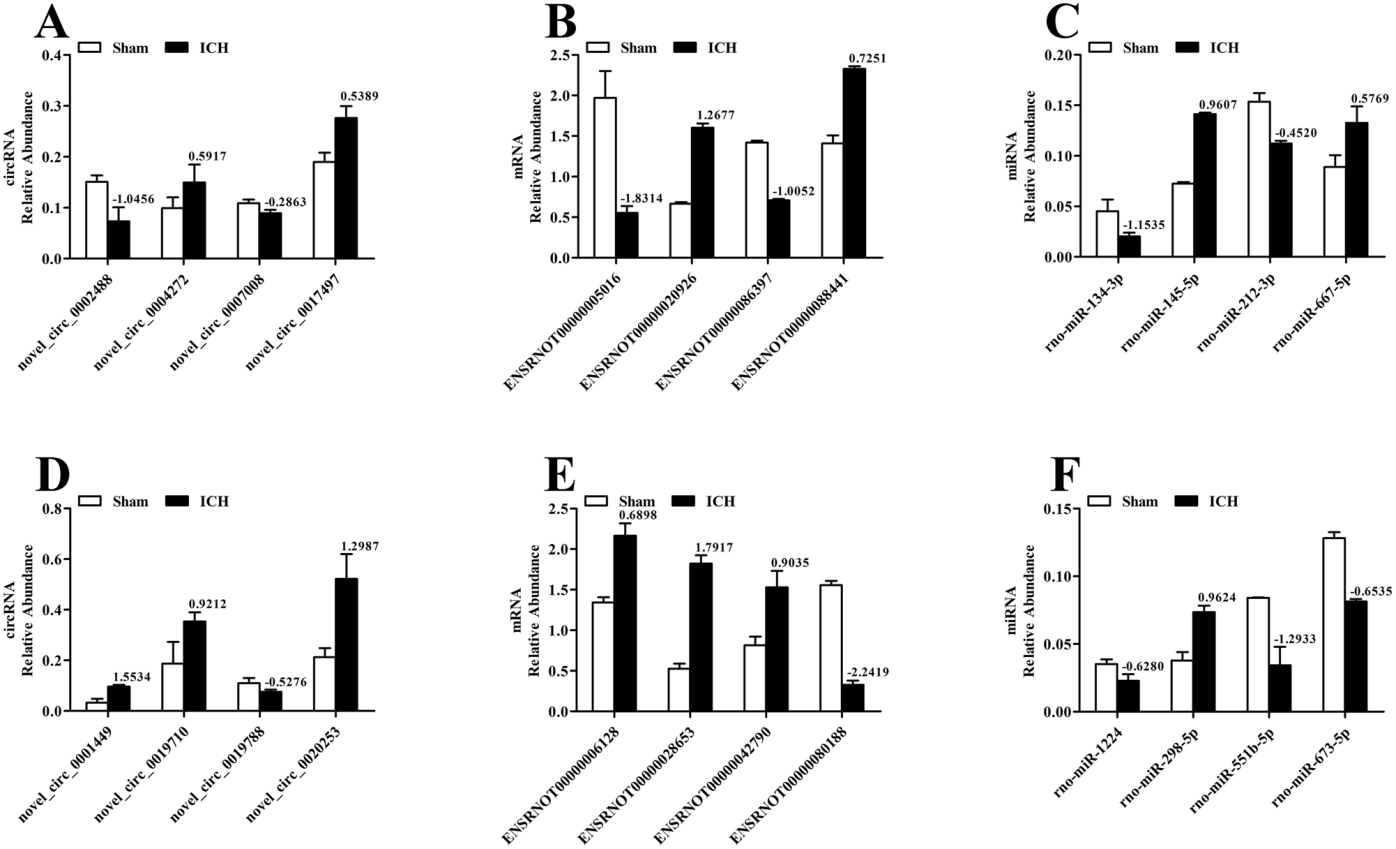
Validation of selected transcripts using qRT-PCR. (**A**) The expression and fold down or up changes (log_2_ scaled) of selected day-7 circRNAs between ICH and Sham groups. (**B**) The expression and fold down or up changes (log_2_ scaled) of selected day-7 mRNAs between ICH and Sham groups. (**C**) The expression and fold down or up changes (log_2_ scaled) of selected day-7 miRNAs between ICH and Sham groups. (**D**) The expression and fold down or up changes (log_2_ scaled) of selected day-28 circRNAs between ICH and Sham groups. (**E**) The expression and fold down or up changes (log_2_ scaled) of selected day-28 mRNAs between ICH and Sham groups. (**F**) The expression and fold down or up changes (log_2_ scaled) of selected day-28 miRNAs between ICH and Sham groups. N = 6 per group. The data were normalized using the “mean ± SEM”.

Similar to the reports from others^26^, quantification of circRNA, mRNA and miRNA by deep sequencing was highly correlated with the results derived from qRT-PCR analyses (Fig. 4, and Supplementary Table S6 for primers details), reflecting the accuracy and reliability of deep sequencing analyses.

### Expression signature of circRNAs, but not mRNAs or miRNAs, differentiated the early and chronic brain activities after ICH

**Figure 5:**
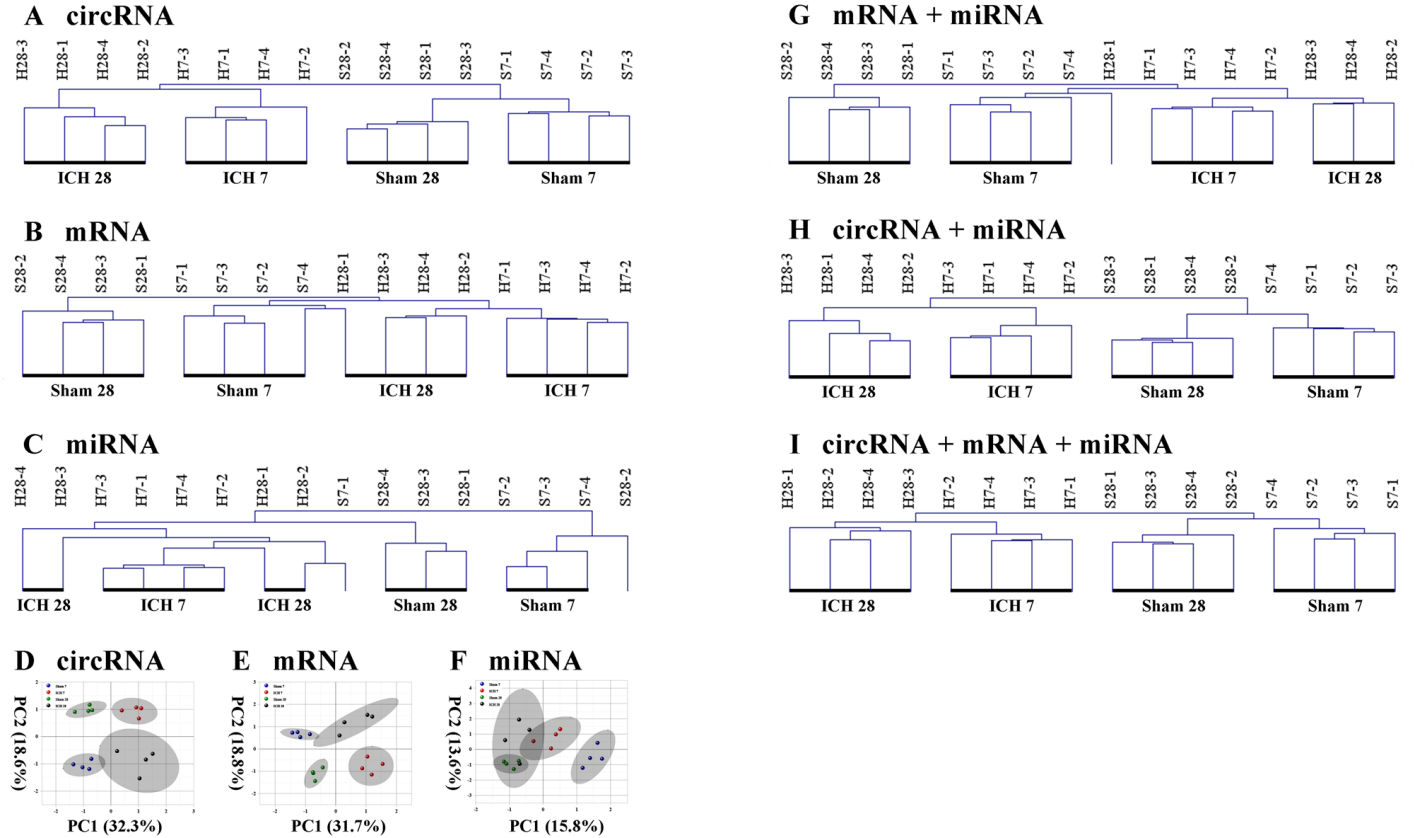
Expression signatures of circRNA, mRNA and miRNA expression profiles. (**A**) Unsupervised hierarchical clustering analysis of circRNA revealed that circRNA provided discriminatory power to precisely classify day-7 and day-28 samples. (**B**) Unsupervised hierarchical clustering analysis of mRNA revealed that mRNA provided discriminatory power to precisely classify day-7 and day-28 samples. (**C**) Unsupervised hierarchical clustering analysis of miRNA revealed that miRNA did not provide discriminatory power to precisely classify day-7 and day-28 samples. Principal component analyses of circRNA (**D**), mRNA (**E**) and miRNA (**F**) expression profiles showed similar findings. The variances explained by the principal components chosen were shown as PC1 and PC2. (**G**) Unsupervised hierarchical clustering analysis of mRNA/miRNA revealed that the mix provided a little stronger discriminatory power than miRNA itself to classify day-7 and day-28 samples. (**H**) Unsupervised hierarchical clustering analysis of circRNA/miRNA revealed that the mix provided a much stronger discriminatory power than miRNA itself to precisely classify day-7 and day-28 samples. (**I**) Unsupervised hierarchical clustering analysis of circRNA/mRNA/miRNA revealed that the mix provided a much stronger discriminatory power than mRNA/miRNA itself to precisely classify day-7 and day-28 samples.

Cluster analysis was used to determine the expression patterns of RNA species under different experimental conditions. By clustering RNAs with the same or similar expression patterns into clusters, the functions of unknown RNAs or unknown functions of annotated RNAs can be identified, since these similar RNAs may have similar functions or participate in the same metabolic process or cellular pathway. Unsupervised hierarchical clustering of the expression profiles of cerebral differentially expressed circRNAs, mRNAs and miRNAs revealed a distinct expression signature of all three RNA species in the ICH, compared to the Sham samples. It revealed that both the expression profile of differentially expressed circRNAs and that of mRNAs provided adequate power to distinguish hemorrhagic from non-hemorrhagic stroke as well as hemorrhage-inducing early (day 7) and chronic (day 28) brain activities, with 4 of the 4 Sham and 4 of the 4 ICH samples classified correctly in each time point (Fig. 5A,B). However, the miRNAs expression profile brought accurate discrimination neither between Sham and ICH groups, nor between day-7 and day-28 ICH samples. In the miRNA clustering, only 3 out of 4 day-7 Sham, 3 out of 4 day-28 Sham, 4 out of 4 day-7 ICH, and 2 out of 4 day-28 ICH individuals were adequately aligned (Fig. 5C). The clustering dendrograms suggested that the expression signatures of circRNA and mRNA in ICH groups played major roles in differentiating brain activities on different stages after cerebral hemorrhage, while miRNA was relatively at a disadvantage. Similarly, PCA plots also revealed the powerful discrimination of circRNA and mRNA expression profiles between day-7-Sham, day-28-Sham, day-7-ICH and day-28-ICH samples (Fig. 5D,E), and the overlap in miRNAs (Fig. 5F) in the day-7 and day-28 samples.

It has previously been reported that combining expression profiles of different RNA species can provide distinctions for cardiomyopathy of different etiology^27^. Similarly, to further explore the possibility that the marked difference in the discriminatory power among differentially expressed circRNAs, mRNAs and miRNAs, additional analyses were conducted. The identified sets of mRNA and miRNA, circRNA and miRNA, as well as circRNA, mRNA and miRNA, were subjected to cluster the Sham and ICH samples, on the 7th and 28th, respectively. The analyses revealed that the combination of mRNA or/and circRNA and miRNA was more powerful than miRNA itself to discriminate both day-7-Sham from day-28-Sham samples and day-7-ICH from day-28-ICH samples. For mRNA/miRNA combination, the mix provided adequate power to discriminate day-7 Sham from day-28 Sham, with 4 out of 4 day-7-Sham and 4 out of 4 day-28-Sham samples were classified correctly; and demonstrated sufficient ability to distinguish day-7-ICH from day-28-ICH samples, with 4 of the 4 day-7-ICH and 3 of the 4 day-28-ICH samples were correctly classified (Fig. 5G). The miRNA profile combined with circRNA delivered a relatively stronger power in discrimination than mRNA/miRNA mix, for the ability to distinguish between these four groups completely and accurately (Fig. 5H). What’s more, the circRNA/mRNA/miRNA combination demonstrated the same differentiating ability as circRNA/miRNA mix on account of the finding that the combination provided adequate discriminatory power among day-7-Sham, day-28-Sham, day-7-ICH, and day-28-ICH samples, with 4 in 4 samples were classified correctly (Fig. 5I).

Taken together, these data suggested that the circRNA expression profile was more sensitive than those of either mRNA or miRNA in discriminating the early (day 7) and chronic (day 28) brain activities after ICH. Considering the physiological functions of circRNA that transcriptional regulation of parent genes, binding to miRNAs as ceRNAs, promoting rolling-circle translation, and alternative splicing of mRNAs^28^, circRNA may provide an orthogonal and powerful marker for each disease state through these mechanisms.

### Functional enrichment analyses of differentially expressed mRNAs in ICH

**Figure 6:**
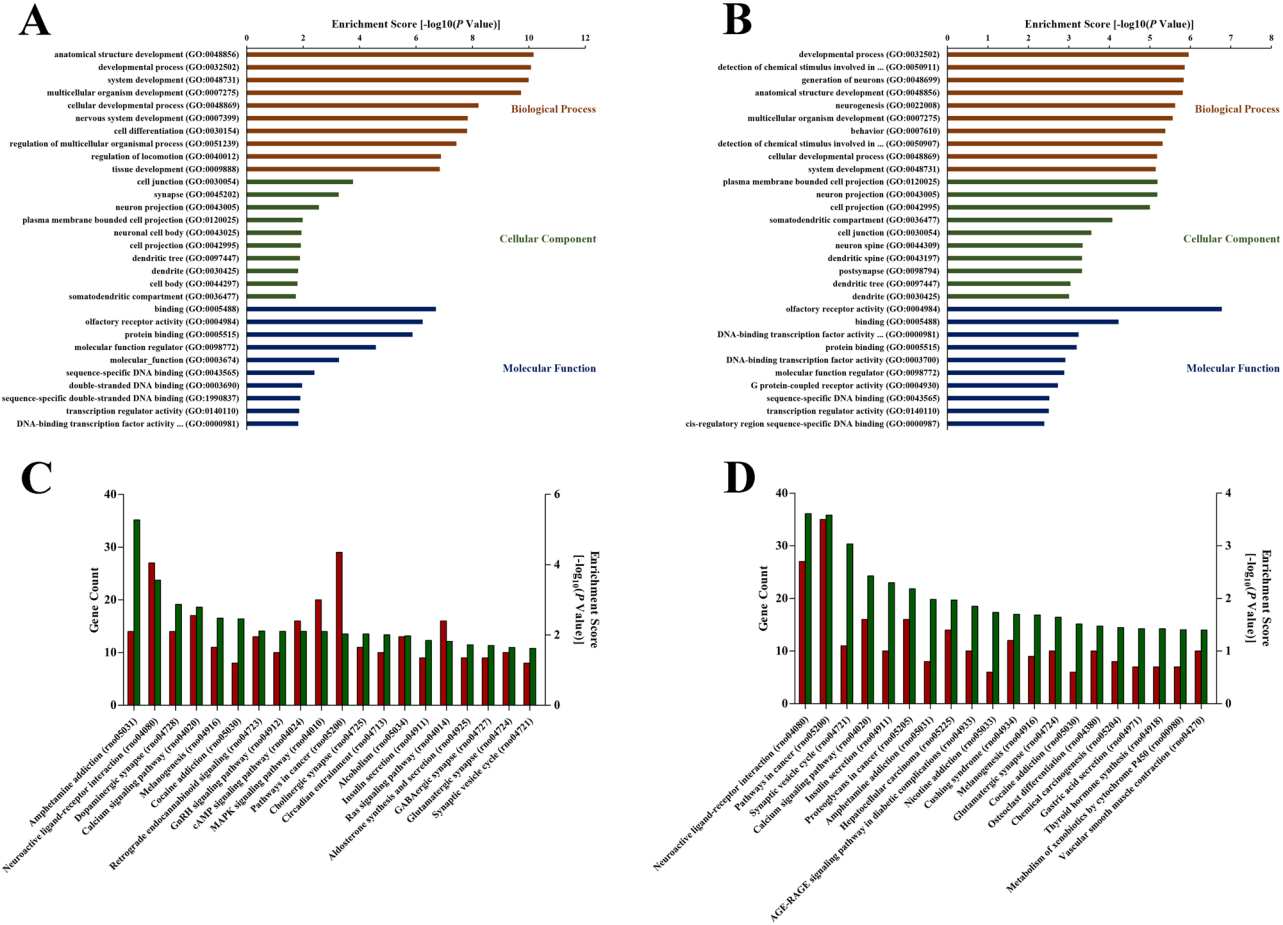
The GO and KEGG pathway annotations of differentially expressed mRNAs in ICH. (**A**) The top 10 most significant enrichment terms in biological process, cellular component, and molecular function, respectively, of differentially expressed mRNAs on day 7 in GO analysis. (**B**) The top 10 most significant enrichment terms in biological process, cellular component, and molecular function, respectively, of differentially expressed mRNAs on day 28 in GO analysis. Red bar represented the term of biological process; green bar represented the term of cellular component; blue bar represented the term of molecular function. The enrichment score (-log_10_ scale of corrected *P* value) expressed the regulational extent of the predicted functions by the differentially expressed mRNAs in ICH rats compared with Sham. (**C**) The top 20 most significant enrichment terms of differentially expressed mRNAs on day 7 in KEGG analysis. (**D**) The top 20 most significant enrichment terms of differentially expressed mRNAs on day 28 in KEGG analysis. Red bar represented the gene count enriched; green bar represented the enrichment score (-log_10_ scale of corrected *P* value).

Functional enrichment analyses of differentially expressed mRNAs were performed through the host genes of mRNAs annotated in GO and KEGG databases, illuminating the mechanisms involved in the development of ICH. GO enrichment analysis predicted the functional roles of target host genes based on three aspects, including biological processes, cellular components, and molecular functions. The results revealed that total 346 terms in the ontology of biological process, 14 terms in the ontology of cellular component, and 17 terms in the ontology of molecular function were significantly enriched (subject to the threshold of corrected *P* value no more than 0.05) with the differentially expressed mRNAs on day 7. The first 10 terms in each ontology were considered as the most important ones for the most significant *P* values they bearing, as shown in Fig. 6A, including anatomical structure development (ontology:biological process, GO:0048856), developmental process (ontology:biological process, GO:0032502), system development (ontology:biological process, GO:0048731), cell junction (ontology:cellular component, GO:0030054), synapse (ontology:cellular component, GO:0045202), neuron projection (ontology:cellular component, GO:0043005), binding (ontology:molecular function, GO:0005488), olfactory receptor activity (ontology:molecular function, GO:0004984), protein binding (ontology:molecular function, GO:0005515), and so forth. And likewise, 139 terms in the ontology of biological process, 18 terms in the ontology of cellular component, and 18 terms in the ontology of molecular function were significantly enriched with the differentially expressed mRNAs on day 28. The first 10 terms in each ontology were outlined in Fig. 6B, such as developmental process (ontology:biological process, GO:0032502), detection of chemical stimulus involved in sensory perception of smell (ontology:biological process, GO:0050911), generation of neurons (ontology:biological process, GO:0048699), plasma membrane bounded cell projection (ontology:cellular component, GO:0120025), neuron projection (ontology:cellular component, GO:0043005), DNA-binding transcription factor activity, RNA polymerase II-specific (ontology:molecular function, GO:0000981), etc..

KEGG analysis defines the pathways related to the functions of genes. In the KEGG analysis, total 261 and 266 terms were enriched with day-7 and day-28 mRNAs, respectively. As shown in Fig. 6C, the top 20 ones bearing the most significant *P* values on day 7 included Neuroactive ligand-receptor interaction (rno04080), Dopaminergic synapse (rno04728), Calcium signaling pathway (rno04020), GnRH signaling pathway (rno04912), Synaptic vesicle cycle (rno04721), and so on. Similarly, the top 20 terms such as Neuroactive ligand-receptor interaction (rno04080), Synaptic vesicle cycle (rno04721), Calcium signaling pathway (rno04020), Glutamatergic synapse (rno04724) and Vascular smooth muscle contraction (rno04270), enriched with day-28 mRNAs, were involved in the pathological development of chronic ICH (Fig. 6D).

### Construction of circRNA/miRNA/mRNA—associated ceRNA networks

CircRNA has miRNA binding sites and can act as a miRNA sponge to competitively bind to miRNA, inhibit the regulatory effect of miRNA on target genes, and thereby indirectly regulate gene expression^28^. In the present study, the miRNA target sites in exons of circRNA loci were identified using miRanda. The predicted miRNAs of circRNA-miRNA pairs were further filtered by matching the differentially expressed miRNAs selected previously, then the information of differentially expressed circRNA-miRNA pairs was obtained. Next, the target mRNAs of differentially expressed miRNAs were retrieved from miRanda and RNAhybrid. The predicted mRNAs of miRNA-mRNA pairs were further filtered by matching the differentially expressed mRNAs picked before, then the information of differentially expressed miRNA-mRNA pairs was obtained. Finally, based on the ceRNA theory, we screened circRNA-mRNA pairs with the same miRNA binding sites, then constructed circRNA-miRNA-mRNA pairs with circRNA as the decoy, miRNA as the core and mRNA as the target. For the whole transcriptome, the ceRNA regulatory network perhaps reveals a new mechanism for noncoding RNA to regulate gene expression.

**Figure 7:**
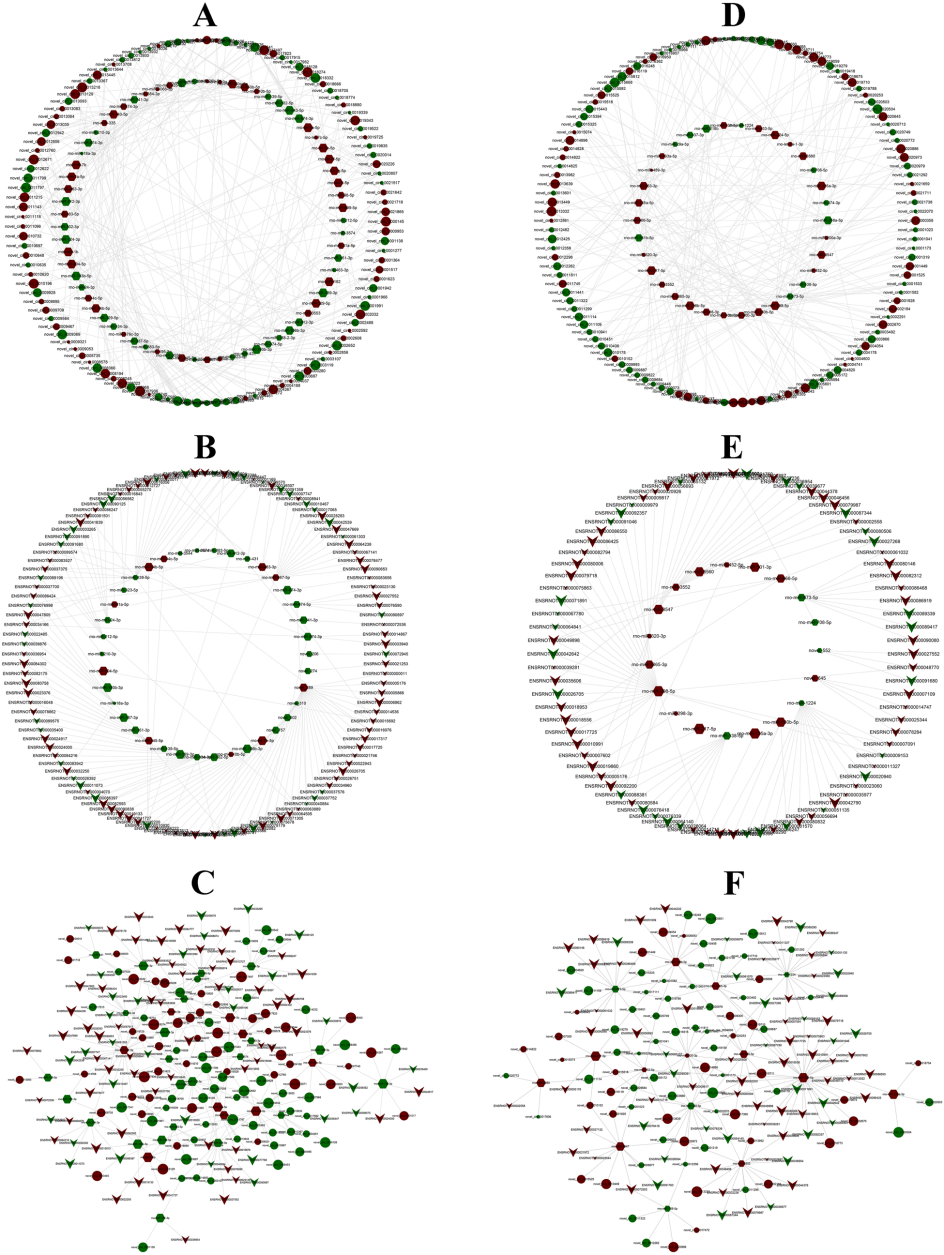
Analysis of circRNA/miRNA/mRNA networks. (A) The day-7 circRNA/miRNA network included 117 circRNAs, 69 miRNAs and 289 relationships. (**B**) The day-7 miRNA/mRNA network included 38 miRNAs, 116 mRNAs and 149 relationships. (**C**) The day-7 circRNA/miRNA/mRNA network was constructed with 97 circRNAs as the decoy, 32 miRNAs as the core and 89 mRNAs as the target. (**D**) The day-28 circRNA/miRNA network included 124 circRNAs, 34 miRNAs and 219 relationships. (**E**) The day-28 miRNA/mRNA network included 19 miRNAs, 84 mRNAs and 100 relationships. (**F**) The day-28 circRNA/miRNA/mRNA network was constructed with 81 circRNAs as the decoy, 15 miRNAs as the core and 77 mRNAs as the target. Ellipse, hexagon, and V-shape nodes represented circRNA, miRNA and mRNA, respectively. The node size represented the corrected *P* value (larger nodes for more significant *P* values). Green and red colors represented down‐ and up‐regulation, respectively.

On the 7th day, a circRNA-miRNA regulatory network, based on the sequencing results, was constructed containing 117 differentially expressed circRNAs (57 downregulated and 60 upregulated), 69 differentially expressed miRNAs (33 downregulated and 36 upregulated) and 289 relationships (Fig. 7A); the miRNA-mRNA co-expressed network included 38 differentially expressed miRNAs (28 downregulated and 10 upregulated), 116 differentially expressed mRNAs (38 downregulated and 78 upregulated) and 149 relationships (Fig. 7B); and the circRNA-miRNA-mRNA regulatory network was constructed with 97 circRNAs (50 downregulated and 47 upregulated) as the decoy, 32 miRNAs (23 downregulated and 9 upregulated) as the core and 89 mRNAs (31 downregulated and 58 upregulated) as the target (Fig. 7C). Likewise, a circRNA-miRNA interaction network containing 124 differentially expressed circRNAs (70 downregulated and 54 upregulated), 34 differentially expressed miRNAs (10 downregulated and 24 upregulated) and 219 relationships (Fig. 7D), a miRNA-mRNA interaction network containing 19 differentially expressed miRNAs (5 downregulated and 14 upregulated), 84 differentially expressed mRNAs (29 downregulated and 55 upregulated) and 100 relationships (Fig. 7E), and a circRNA-miRNA-mRNA regulatory network including 81 circRNAs (49 downregulated and 32 upregulated), 15 miRNAs (4 downregulated and 11 upregulated) and 77 mRNAs (27 downregulated and 50 upregulated) (Fig. 7F), were constructed on the 28th day.

**Figure 8:**
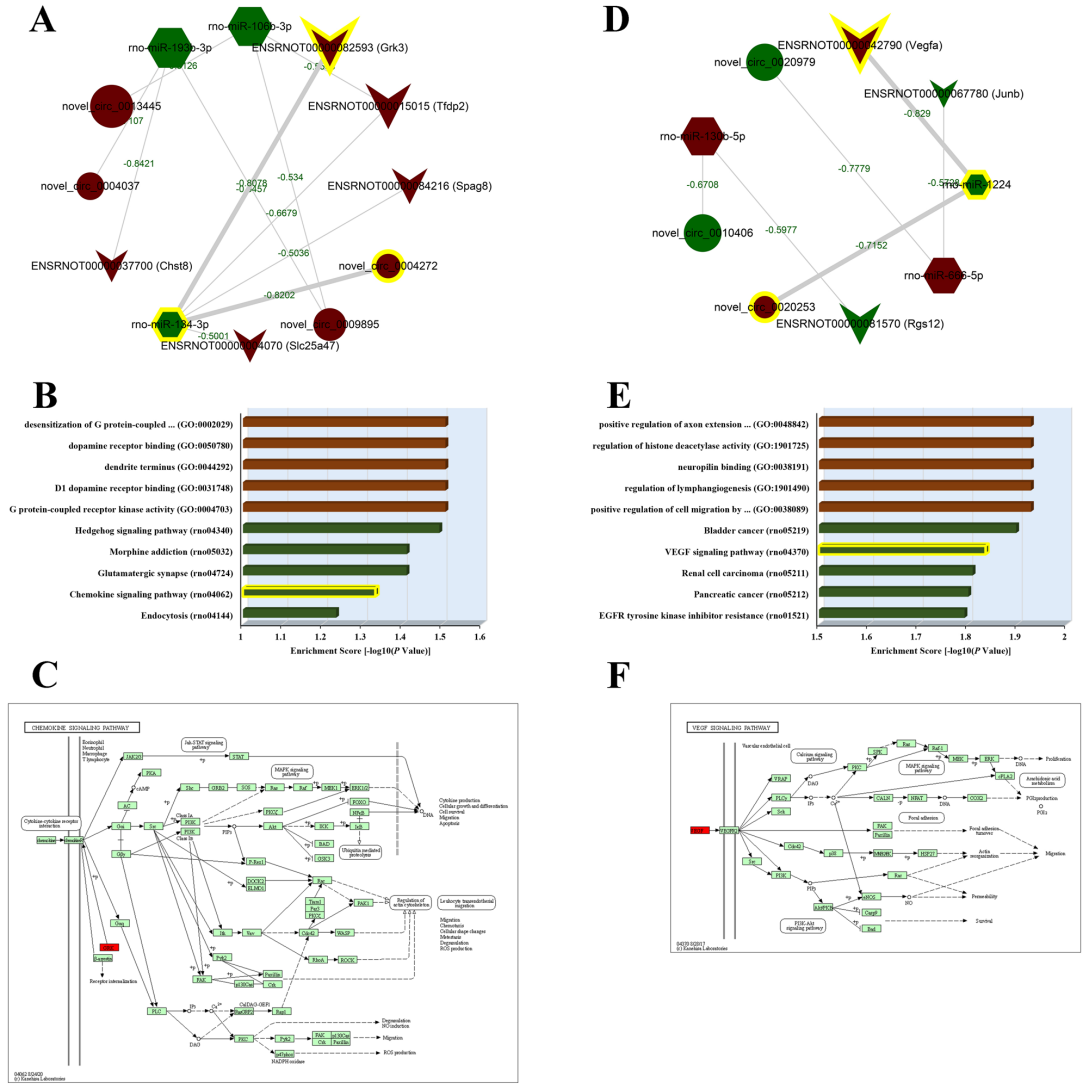
Analysis of hub ceRNA regulatory networks. (A) The 8 pairs of ceRNA networks constructed with 12 hub nodes on the 7th day. Ellipse, hexagon, and V-shape nodes represented circRNA, miRNA and mRNA, respectively. The node size represented the corrected *P* value (larger nodes for more significant *P* values). Green and red colors represented down‐ and up‐regulation, respectively. The green values on edges represented the negative *Pearson* correlation coefficients. The most qualified sub-network of novel_circ_0004272/rno-miR-134-3p/ENSRNOT00000082593(Grk3) was highlighted by yellow. (**B**) The top 5 most significantly enriched GO and KEGG terms of Grk3. Red bars represented the 5 GO terms; green bars represented the 5 KEGG terms. The enrichment score (-log_10_ scale of corrected *P* value) expressed the regulational extent of the predicted functions by Grk3 in ICH rats compared with Sham. The most qualified KEGG term of Chemokine signaling pathway was highlighted by yellow. (**C**) Detail of Chemokine signaling pathway (https://www.kegg.jp/kegg-bin/show_pathway?map04062). The target gene was highlighted by red. (**D**) The 3 pairs of ceRNA networks constructed with 9 hub nodes on the 28th day. The most qualified sub-network of novel_circ_0020253/rno-miR-1224/ENSRNOT00000042790(Vegfa) was highlighted by yellow. (**E**) The top 5 most significantly enriched GO and KEGG terms of Vegfa. The most qualified KEGG term of VEGF signaling pathway was highlighted by yellow. (**F**) Detail of VEGF signaling pathway (https://www.kegg.jp/kegg-bin/show_pathway?map04370). The target gene was highlighted by red.

The hub transcripts in the ceRNA network were recognized through the engagement of RNA co-expression analysis with correlation coefficients of circRNA-miRNA and miRNA-mRNA pairs less than -0.5 and relational *P* values no more than 0.05. A total of 12 transcripts, including novel_circ_0004037, novel_circ_0004272, novel_circ_0009895, novel_circ_0013445, rno-miR-193b-3p, rno-miR-134-3p, rno-miR-106b-3p, ENSRNOT00000037700, ENSRNOT00000004070, ENSRNOT00000015015, ENSRNOT00000082593 and ENSRNOT00000084216, were selected from the expression profile on day 7 as hub nodes. The sub-network was shown in Fig. 8A. Three nodes (novel_circ_0004272, rno-miR-134-3p, ENSRNOT00000082593) were found to have the most perfect interaction due to the closer correlation and well as the more relevant functional annotations of target gene. The top 5 GO and KEGG terms enriched with the hub gene Grk3 were detailed in Fig. 8B, which indicated that the novel_circ_0004272/rno-miR-134-3p/ENSRNOT00000082593 sub-network may play crucial roles in the early pathological development process of ICH via regulating G protein-coupled receptor kinase activity, dendrite terminus and dopamine receptor binding, through Hedgehog signaling pathway, Glutamatergic synapse and Chemokine signaling pathway. The specific role of Grk3 in the Chemokine signaling pathway was took as an example, as shown in Fig. 8C.

Similarly, a sub-network consisting of 9 transcripts including novel_circ_0010406, novel_circ_0020253, novel_circ_0020979, rno-miR-130b-5p, rno-miR-1224, rno-miR-666-5p, ENSRNOT00000081570, ENSRNOT00000042790 and ENSRNOT00000067780, stood out from the expression profile on day 28 (Fig. 8D). In particular, the novel_circ_0020253/rno-miR-1224/ENSRNOT00000042790 sub-network was authorized as the hub. The top 5 GO and KEGG terms enriched with the target gene Vegfa were detailed in Fig. 8E, which suggested the possible mechanism of novel_circ_0004272/rno-miR-134-3p/ENSRNOT00000082593 sub-network in the chronic brain activities of ICH, such as positive regulation of cell migration by vascular endothelial growth factor signaling pathway, neuropilin binding and positive regulation of axon extension involved in axon guidance. The specific role of Vegfa in the VEGF signaling pathway was took as an example, as shown in Fig. 8F.

## Discussion

The present study utilized next-generation sequencing to provide a quantitative and comprehensive analysis of the coding and noncoding transcriptome in hemorrhagic and non-hemorrhagic rat brain tissues, at the early (day 7) and the chronic (day 28) stages after insults, respectively. These analyses revealed significant differences in the patterns of circRNA, mRNA and miRNA expression in ICH samples compared with Sham, as well as dynamic changes in response to disease progression. Here we showed for the first time that the expression patterns of circRNA and mRNA discriminated between day-7 versus day-28 ICH, whereas the miRNA expression signature was at a disadvantage in distincting between ICH development periods. Moreover, this study showed that the changes in the expression levels of circRNAs were more dynamically regulated following ICH, depending on the observation that the expression pattern of circRNA in the cerebral hemorrhage was both temporal-specific as well as sensitive to brain activities.

The early brain activities after hemorrhage are mainly characterized by cell death and local inflammation, accompanied by BBB destruction and edema formation; as for the chronic, the inflammatory response pretty nearly disappears, and the BBB repair obviously takes the lead, including but not limited to neurogenesis, angiogenesis and tight junction assembly; however, it is sometimes likely to be beyond expectations that there may be a long-term BBB failure leading to neurodegeneration^29^. Accordingly, the fundamental aspect of treatment protocols for acute and chronic stroke is the nature of the molecules targeted for treatment. Understanding the timing of the expression of each of the agents to be regulated and their actions at each point of the activity cycle is prerequisite in planning the use of mediators. In the study, we have identified 162 and 179 circRNAs, 618 and 676 mRNAs, and 80 and 39 miRNAs with significantly differential expression at *P*_0.05_ threshold, on the 7th and 28th days after ICH, respectively, in ICH rats compared with Sham group. The findings suggested that these differentially expressed cerebral RNAs can be potential indicators for distinct brain activities in different stages of ICH.

Alternative splicing (AS) is a key process contributing to the diversity of mRNAs and proteins in multicellular eukaryotes. According to reports, there are approximately 95% of human multiexon genes undergoing AS^30^ and 2–12 different isoforms can be generated from genetic switches caused by AS events such as skipped exons, mutually exclusive exons, alternative 5’ or 3’ splicing sites and retained introns^31^. Of the classes of AS, intron retention (IR) has been demonstrated as a central component of gene expression programs during normal development as well as in response to stresses and diseases^32^. For example, IR coupled with nonsense mediated decay (NMD) are utilized to precisely modulate the levels and spatial expression patterns of Robo3 gene to control axon guidance in the spinal cord during embryonic development; while mouse embryos subjected to knockout of the NMD factor Upf2, showed excessive Robo3.2 expression and disrupted axonal trajectories^33^. AS is also prevalent in circRNA biogenesis, but not completely in the same pattern as in the corresponding mRNA^34^. For instance, Gao et al*.* revealed the existence of non-exonic circRNAs containing intronic or intergenic sequences that are not present in known mature mRNAs^35^, indicating a specific internal composition of circRNAs, upon which circRNAs are divided into three categories, including exonic circRNAs (ecircRNAs) synthesized from exons only, intronic circRNAs (ciRNAs) just containing intron sequences, and exon–intron circRNAs (EIciRNAs)^36^. In the present study, a total of 22,414 exons and 1,505 introns derived from 3,858 genes, as well as 2,565 intergenic fragments derived from 1,265 intergenic regions, were shown to be involved in the biogenesis of 11,620 candidate circRNAs. It is known that in eukaryotic cells, ecircRNAs are mainly enriched in the cytoplasm, while both ciRNAs and EIciRNAs are more nuclear^37^, therefore, the biogenesis feature suggests possible subcellular localizations of circRNAs. Take novel_circ_0004272 as an example, in the ceRNA network, the candidate novel_circ_0004272 was featured to be intergenic and might not be necessarily as a miRNA sponge if it was derived from intronic regions transcribed from the parental gene, which would contradict the network node proposed currently. In addition, intron-retaining circRNAs can promote the transcription of their parental genes by interacting with U1 small nuclear ribonucleoproteins^38^, which provides a basis for therapeutic modulation of IR in diseases such as cancers^36^. Therefore, a further evaluation on the AS events of corresponding circRNAs and their differences during the occurrence and development of diseases may be a promising understructure for ICH treatment. Unsupervised hierarchical cluster and principal component analyses on these RNAs revealed that no matter it is from hemorrhagic to non-hemorrhagic groups, or from early to chronic brain activities post cerebral hemorrhage, the expression profile of subsets of circRNAs provided adequate rights to distinguish them, whether through circRNAs themselves or by combination with miRNAs or mRNAs/miRNAs mix. However, the expression profile of mRNAs just committed to distinguish the hemorrhage from the non-hemorrhage groups as well as the difference between the early and chronic phases of ICH, when only themselves. In the mRNA/miRNA mix, mRNA did not provide miRNA with enough power to completely distinguish these time-specific brain activities. miRNA was found to be the most unbearable one for the matter that it was even not able to take on the responsibility of supporting the discrimination between ICH and Sham groups with the current restriction. The possible reason why mRNA and miRNA performed worse than circRNA may be that the number of miRNAs sequenced out was less-about 1,000, while circRNA was 10 times and mRNA 30 times more, so it was rather not unexpected that miRNA was a weak discriminator between various experimental groups. In addition, the temporal and spatial specificity may empower circRNA to perform as a distinguisher between different conditions, which is lacking in mRNA and miRNA. Moreover, adding circRNA to miRNA or mRNA/miRNA expression profiles for clustering reversed the indistinguishability of mRNA and miRNA. However, it may be not an inherent problem that sequencing depth did not reflect the true biological effect or specificity of miRNAs. It has been reported that increasing biological replications consistently increases the power of detecting differentially expressed genes significantly, regardless of sequencing depth, in the breast cancer cell samples^39^. Therefore, in the present research, if possible, additional studies conducted on larger numbers of well-annotated samples and exploiting improved sequencing technologies and analytical methods will be of considerable interest. Taken together, our results disclosed that cerebral circRNA expression profiles responded more sensitively to ICH progression than did mRNA and miRNA expression profiles and may serve, therefore, as a useful biomarker to assess brain activities in response to ICH at different stages.

Disruption of BBB plays a key role in the development of neurological dysfunction in acute and chronic cerebral hemorrhage. Basic research in animal models of ICH has provided insight into its complex pathology, in particular revealing the role of inflammation in driving neuronal death and neurological deficits resulting the barrier destroy after hemorrhage^29^. The regulation of BBB during inflammation is a complex process. In the hyperacute phase (within 6 h) of ICH, neuronal death and endothelial damage at the site of hematoma occur rapidly upon the onset of ICH, with which the potent inflammatory factors potentiating immune activation and proteases activating CNS-resident microglia are released or activated^40,41^. This initial cascade of cell death and subsequent localized immunopathology result in the degradation of BBB (progressively increased permeability of cerebral vasculature) observed at later time-points after ICH^42^. Moreover, the local inflammation precipitates recruitment of circulating inflammatory cells such as neutrophils and monocytes/macrophages that subsequently contributes to a secondary inflammatory response in a feed-forward loop for approximately the first 3 days after hemorrhage, whose resulting peak may be maintained until the 7th day^29^. In the chronic phase of ICH (3 to 4 weeks), the inflammation has almost receded and the engagement of tissue repair responses that promote BBB repair and restoration of neurological function has been vigorously induced^43^. Post-stroke angiogenesis is a key step for BBB recovery and provides the critical neurovascular substrates for neuronal remodeling after stroke^44^. The current functional analysis on mRNA expression profiles suggested that the functional annotations of mRNAs altered with ICH at the specific time points were almost consistent with the described. As an example, lymphatic endothelial cell differentiation was found to be one of the most significantly enriched and meaningful terms of biological processes on the 7th day. The lymphatic system is essential for maintaining tissue-fluid homeostasis, providing immune surveillance and mediating lipid absorption^45^. The exertion of these functions is closely related to lymphatic endothelial cells, which are of great importance in triggering inflammation and facilitating fibroblast migration for angiogenesis^46^. For another example, regulation of actin filament bundle assembly and neuron projection morphogenesis were two of the most classical biological processes enriched on the 28th day after ICH. The former is for actin formation mediating cell contraction and through which, endothelial cells can be guided to the site of peri-BBB to participate in the process of vascular remodeling and BBB restoration; and the latter is the extension of axons to the distant, which can be considered as an important process of neurological functional remodeling when cerebral hemorrhage occurs^47^.

Studies have found that circRNA can perform as the ceRNA to play miRNA sponge function. miRNA degrades or inhibits mRNA translation by matching genes targeting the 3' UTR region of mRNA, thereby regulating its gene expression. There are many miRNA response elements on circRNA, which can adsorb miRNA by covalently binding with these response elements and upregulate the downstream target gene expression of miRNA^48^. The interaction networks about differentially expressed circRNAs, miRNAs and target mRNAs were predicted in the present study, among which two specific circRNA-mediated ceRNA networks survived multiple iterations: novel_circ_0004272/rno-miR-134-3p/ENSRNOT00000082593(Grk3) on day 7 and novel_circ_0020253/rno-miR-1224/ENSRNOT00000042790(Vegfa) on day 28. GRK is a protein kinase specifically phosphorylating activated G protein-coupled receptors, thus decoupling the receptor from G protein and preventing it from coupling to G protein again, thereby effectively reducing the level of functional receptors on the cell membrane and making the signal transduction decreased or disappeared. For example, in the chemokine signaling pathway, GRK dissociates the chemokine receptors, and deactivates diverse downstream pathways such as Jak-STAT, MAPK and PI3K-Akt signaling pathways which contribute to neuroinflammation by guiding peripheral leukocyte transendotherial migration, lead to oxidative stress through NO and ROS production, and facilitate junctional formation during BBB creation^49,50^. VEGF is a heparin-binding growth factor specific for vascular endothelial cells to induce angiogenesis. The binding of VEGF to the corresponding receptors leads to a cascade activation of different signaling pathways, resulting in the upregulation of genes involved in mediating the proliferation and migration of endothelial cells and promoting their survival and vascular permeability. VEGF is the strongest angiogenic factor found so far and is related to many physiological and pathological processes. For example, in the VEGF signaling pathway, VEGF can facilitate the proliferation of vascular endothelial cells in a state of hypoxia through intracellular activation of MAPK signaling pathway, provoking focal adhesion to migrate endothelial cells to the extracellular matrix, contributing to the integration of BBB and even neurovascular units, and suppress the expression of caspase 9 through PI3K-Akt signaling pathway to promote cell survival^51^. Taken together, the findings highlighted that brain activities in the early and chronic stages of ICH were regulated by distinct stroke-responsive circRNA-mediated ceRNA nodes. Moreover, taking into account the species conservation and spatiotemporal specificity of circRNA, as well as its binding to RNA binding proteins (RBPs) for regulating the activity of RBPs and the role as a potential translation template for encoded proteins, circRNA is considered to be an ideal biomarker for ICH diagnosis and treatment.

Even though our study was the first to show that circRNAs were dynamically expressed with the progress of ICH and provided the power to discriminate ICH at early and chronic periods, the study has some limitations. First of all, the number of samples analyzed here is relatively small, and factors such as animal species, age, gender, modeling patterns, blood volume, etc., can have effects on disease progress and transcript expression that may, therefore, confound the results of the analyses completed and presented. Further investigation with more comprehensive considerations in larger cohorts of ICH samples needs to be carried out. Second, other technologies such as microarrays should be utilized to further verify the specific discrimination of circRNA to the development of ICH to ensure sufficient argumentation. Third, recent studies have shown that non-coding linear and circular transcripts can be transcribed from the same gene^37^. However, whether the linear RNAs such as long non-coding RNAs and other gene biotypes (e.g., pseudogenes, antisense transcripts and snoRNAs) are powerful in discriminating the samples and as sensitive as circRNAs described in the current study, are unknown, for the reason that these RNAs differ from each other in terms of their classifications, characteristics, functions and structures, despite of the identical parental origin^37^. Therefore, a deeper and more extensive research on the whole transcriptome is indispensable for the discovery of ICH biomarkers. Finally, in addition to ceRNA mechanism, intron-retaining circRNAs may also offer a tractable therapeutic target through mediating the nuclear translocation of their bound RBPs, therefore, further evaluations should be prudentially carried out in in vitro and in vivo models for a better understanding of the potential therapeutic targets.

## Conclusion

Taken together, the study presented here suggests, to our knowledge, for the first time, that circRNA plays an important functional role in the pathological progress of ICH and well as contributes to distinguish between early and chronic ICH. The results also reveal distinct relative abundance, expression pattern and genomic origin of cerebral circRNA, mRNA and miRNA, highlighting the different biological roles of the individual RNA classes during evolution. Despite its exploratory nature, the current study offers deep insights into many possible biomarkers for the early diagnosis and prognosis assessment of ICH, and well as therapeutic targets assisting the development of new drugs.

## Supplementary Information

Supplementary Information.

## Data Availability

The raw data supporting the conclusions of this manuscript will be made available by the authors, without undue reservation, to any qualified researcher.
