# Supplementary material for: Reconstruction of circRNA-miRNA-mRNA associated ceRNA networks reveal functional circRNAs in intracerebral hemorrhage
Source: Sci Rep. 2021 Jun 2;11:11584. doi: 10.1038/s41598-021-91059-9 (PMC8172571; doi:10.1038/s41598-021-91059-9)
Supplement: Supplementary file 1 — Supplementary Information. [file 41598_2021_91059_MOESM1_ESM.pdf]

Reconstruction of circRNA-miRNA-mRNA Associated ceRNA Networks Reveal Functional circRNAs in Intracerebral Hemorrhage

Zhen Liu, Xinran Wu, Zihan Yu, Xiaobo Tang*
Department of Biopharmaceutical Sciences (State-Province Key Laboratories of Biomedicine-Pharmaceutics of China), College of Pharmacy, Harbin Medical University, Harbin, Heilongjiang, China.

***Correspondence:**
Corresponding Author: Xiaobo Tang
Department of Biopharmaceutical Sciences (State-Province Key Laboratories of Biomedicine-Pharmaceutics of China), College of Pharmacy, Harbin Medical University, P. O. Box 19, 157 Baojian Road, Nangang District, Harbin, Heilongjiang 150081, China.
E-mail Address: ty6163@aliyun.com

Supplementary Table S1. Classification of mapped reads. Mt_rRNA: Mitochondrial ribosomal RNA. **Mt_tRNA:** Mitochondrial transfer RNA. **lincRNA:** Long intergenic non-coding RNA. **misc_RNA:** Miscellaneous RNA. **scaRNA:** Small Cajal body-specific RNA. **snRNA:** Small nuclear RNA. **snoRNA:** Small nucleolar RNA.

**Samples**	**ICH7-1**	**ICH7-2**	**ICH7-3**	**ICH7-4**	**Sham7-1**	**Sham7-2**	**Sham7-3**	**Sham7-4**
Mt_rRNA	64670 (0.16%)	68248 (0.18%)	84263 (0.22%)	55454 (0.17%)	76176 (0.20%)	47932 (0.13%)	44631 (0.12%)	8670 (0.02%)
Mt_tRNA	1161 (0.00%)	956 (0.00%)	1262 (0.00%)	1144 (0.00%)	1118 (0.00%)	905 (0.00%)	982 (0.00%)	942 (0.00%)
antisense	4731 (0.01%)	4636 (0.01%)	4518 (0.01%)	3493 (0.01%)	5848 (0.02%)	4941 (0.01%)	5033 (0.01%)	5215 (0.01%)
lincRNA	202858 (0.50%)	210047 (0.56%)	199708 (0.53%)	140426 (0.43%)	240738 (0.62%)	194815 (0.54%)	169671 (0.46%)	247675 (0.65%)
misc_RNA	338984 (0.83%)	160737 (0.43%)	229935 (0.61%)	85323 (0.26%)	299439 (0.77%)	64210 (0.18%)	354794 (0.96%)	381131 (1.00%)
protein_coding	29716503 (72.82%)	27309601 (72.69%)	27310986 (72.15%)	23946361 (72.63%)	27159716 (70.07%)	26217566 (72.98%)	26929920 (73.20%)	26930802 (70.68%)
pseudogene	80486 (0.20%)	68644 (0.18%)	69279 (0.18%)	60308 (0.18%)	59431 (0.15%)	67614 (0.19%)	69500 (0.19%)	56422 (0.15%)
scaRNA	4202 (0.01%)	3595 (0.01%)	4691 (0.01%)	3448 (0.01%)	3826 (0.01%)	2801 (0.01%)	3225 (0.01%)	2951 (0.01%)
snRNA	682 (0.00%)	614 (0.00%)	692 (0.00%)	649 (0.00%)	659 (0.00%)	557 (0.00%)	633 (0.00%)	646 (0.00%)
snoRNA	6703 (0.02%)	5518 (0.01%)	6427 (0.02%)	5242 (0.02%)	9001 (0.02%)	5462 (0.02%)	5945 (0.02%)	6659 (0.02%)
others	10097110 (24.74%)	9466496 (25.20%)	9649882 (25.49%)	8421871 (25.54%)	10621188 (27.40%)	9065966 (25.24%)	8957699 (24.35%)	10195784 (26.76%)
**Samples**	**ICH28-1**	**ICH28-2**	**ICH28-3**	**ICH28-4**	**Sham28-1**	**Sham28-2**	**Sham28-3**	**Sham28-4**
Mt_rRNA	174702 (0.39%)	145607 (0.41%)	185286 (0.37%)	25158 (0.06%)	176381 (0.39%)	115005 (0.32%)	130666 (0.29%)	209562 (0.43%)
Mt_tRNA	1569 (0.00%)	1313 (0.00%)	2057 (0.00%)	2190 (0.01%)	1900 (0.00%)	1229 (0.00%)	2081 (0.00%)	2133 (0.00%)
antisense	5635 (0.01%)	3955 (0.01%)	6382 (0.01%)	4577 (0.01%)	4909 (0.01%)	4719 (0.01%)	5170 (0.01%)	5543 (0.01%)
lincRNA	188071 (0.42%)	140938 (0.40%)	260864 (0.52%)	175281 (0.41%)	151226 (0.33%)	162040 (0.44%)	155338 (0.34%)	196774 (0.40%)
misc_RNA	419160 (0.93%)	180815 (0.51%)	167992 (0.33%)	211940 (0.50%)	194963 (0.43%)	182675 (0.50%)	319663 (0.71%)	603941 (1.24%)
protein_coding	32459560 (72.17%)	25795281 (73.11%)	36625891 (72.71%)	30984750 (73.02%)	33089101 (73.13%)	26247542 (71.98%)	32906461 (72.58%)	34777674 (71.48%)
pseudogene	86919 (0.19%)	64872 (0.18%)	91409 (0.18%)	79816 (0.19%)	88374 (0.20%)	60053 (0.16%)	85054 (0.19%)	87271 (0.18%)
scaRNA	5332 (0.01%)	5106 (0.01%)	5228 (0.01%)	5478 (0.01%)	5121 (0.01%)	3058 (0.01%)	5279 (0.01%)	5713 (0.01%)
snRNA	667 (0.00%)	638 (0.00%)	779 (0.00%)	733 (0.00%)	637 (0.00%)	597 (0.00%)	718 (0.00%)	805 (0.00%)
snoRNA	6372 (0.01%)	5284 (0.01%)	6486 (0.01%)	4472 (0.01%)	4966 (0.01%)	5295 (0.01%)	4515 (0.01%)	7783 (0.02%)
others	11281913 (25.08%)	8683645 (24.61%)	12651436 (25.12%)	10589500 (24.96%)	11192722 (24.74%)	9415410 (25.82%)	11376233 (25.09%)	12388742 (25.46%)

Supplementary Table S2. Details of differentially expressed circRNAs in day-7 and day-28 ICH samples when compared with Sham. Chr: Chromosome ID where circRNA is located. **Gene ID:** The parent gene of circRNA. **Start/End:** The full-length start or termination site of circRNA in the genome. **Strand:** The feature of the strand in the host gene forming circRNA; "+" indicates the sense strand, and "-" indicates the antisense strand. **Feature:** The splicing feature of genome forming circRNA (the total length of all splicing positions of the genome is the splice length of circRNA); "exonic" indicates circRNA spliced from exon, "intronic" indicates circRNA spliced from intron, and "intergenic" indicates circRNA spliced from intergenic region. **Count:** Numbers of corresponding exons, introns or intergenic regions. **log2(FC):** The log scale of fold change (the absolute ratio of normalized intensities between ICH and Sham groups). ***P* Value:** Significance index of statistical difference. ***Q* Value:** The corrected *P* Value. **ICH 1 to 4** and **Sham 1 to 4:** Read count after TPM normalization correction of each sample.

Day-7 circRNA	Chr	Gene ID	Start	End	Strand	Feature	Count	log2(FC)	*P* Value	*Q* Value	ICH1	ICH2	ICH3	ICH4	Sham1	Sham2	Sham3	Sham4
novel_circ_0000126	chr10	ENSRNOG00000010771	13931235	13933066	+	exonic	2	-1.1119	0.0008	0.0479	82.5645	0	125.1512	68.0596	157.2292	225.4678	118.3404	241.2953
novel_circ_0001138	chr10	ENSRNOG00000003905	91965973	91969538	-	exonic	3	-1.6217	0.0001	0.0143	0	0	41.7171	68.0596	89.8452	125.2599	260.3489	96.5181
novel_circ_0001277	chr10	ENSRNOG00000003800	97555576	97568711	-	exonic	5	-1.3604	0.0008	0.0466	0	143.0165	0	0	67.3839	225.4678	118.3404	120.6476
novel_circ_0001942	chr11	ENSRNOG00000029614	9799898	9800225	+	exonic	1	-1.0885	0.0001	0.0141	144.4878	114.4132	104.2927	181.4923	202.1518	375.7797	402.3573	289.5543
novel_circ_0001966	chr12	ENSRNOG00000025155	12448357	12463405	-	exonic	5	-1.3966	0.0007	0.0448	0	0	0	0	44.9226	125.2599	0	72.3886
novel_circ_0001986	chr12	ENSRNOG00000001101	13524226	13527329	+	exonic	1	-1.7151	0.0001	0.0166	0	0	0	0	112.3066	0	94.6723	96.5181
novel_circ_0001991	chr12	ENSRNOG00000024482	13879068	13884323	+	exonic	2	-2.0238	0.0000	0.0028	0	0	0	68.0596	179.6905	175.3639	142.0085	96.5181
novel_circ_0002488	chr13	ENSRNOG00000002653	107733301	107749528	-	exonic	3	-0.6049	0.0002	0.0204	1032.0556	886.7023	563.1805	499.1039	853.5298	1077.2353	1491.0890	1158.2173
novel_circ_0002537	chr13	ENSRNOG00000003841	111138405	111159973	+	exonic	3	-0.8097	0.0004	0.0330	309.6167	143.0165	312.8781	204.1789	471.6875	350.7278	307.6850	675.6268
novel_circ_0002652	chr13	ENSRNOG00000032490	31140420	31163102	+	exonic	3	-0.9536	0.0000	0.0021	350.8989	543.4627	250.3024	294.9250	763.6846	776.6115	520.6977	748.0153
novel_circ_0003107	chr13	ENSRNOG00000002544	82185979	82190210	+	exonic	4	-0.6097	0.0006	0.0423	495.3867	257.4297	542.3220	476.4173	471.6875	801.6635	710.0424	820.4039
novel_circ_0003119	chr13	n/a	83256697	83257043	-	intergenic	1	-1.9617	0.0000	0.0000	61.9233	286.0330	229.4439	0	404.3036	526.0916	781.0466	796.2744
novel_circ_0003267	chr13	ENSRNOG00000021497	95098505	95128782	-	exonic	3	-2.0819	0.0000	0.0029	41.2822	0	0	0	112.3066	150.3119	189.3446	72.3886
novel_circ_0003687	chr14	ENSRNOG00000030149	28448336	28465972	-	exonic	2	-2.0970	0.0000	0.0039	0	0	0	0	67.3839	100.2079	47.3362	144.7772
novel_circ_0003821	chr14	n/a	42007312	42015391	+	intergenic	3	-1.6186	0.0002	0.0228	0	0	0	0	44.9226	100.2079	0	144.7772
novel_circ_0004461	chr15	ENSRNOG00000013804	25905490	25913420	-	exonic	4	-1.4587	0.0006	0.0441	0	0	41.7171	0	67.3839	75.1559	94.6723	96.5181
novel_circ_0004464	chr15	ENSRNOG00000013804	25917998	25921639	-	exonic	1	-1.4121	0.0006	0.0433	0	0	0	0	44.9226	50.1040	47.3362	72.3886

novel_circ_0004985	chr16	ENSRNOG00000022781	14184371	14186953	-	exonic	1	-0.9222	0.0002	0.0202	247.6934	200.2231	312.8781	181.4923	247.0744	851.7674	497.0297	313.6839
novel_circ_0005300	chr16	ENSRNOG00000031287	5417617	5422411	-	exonic	3	-1.6316	0.0002	0.0238	0	57.2066	0	0	44.9226	250.5198	165.6766	0
novel_circ_0005365	chr16	ENSRNOG00000010392	63915077	63924142	+	exonic	2	-1.4792	0.0002	0.0224	41.2822	57.2066	41.7171	0	67.3839	175.3639	71.0042	265.4248
novel_circ_0005542	chr16	ENSRNOG00000012573	79918763	79924180	-	exonic	2	-1.8252	0.0001	0.0113	0	0	0	0	67.3839	200.4159	0	72.3886
novel_circ_0005582	chr16	ENSRNOG00000019978	8416755	8421217	+	exonic	3	-0.5250	0.0003	0.0315	763.7212	600.6693	730.0488	612.5366	988.2977	851.7674	923.0551	1182.3468
novel_circ_0006257	chr17	ENSRNOG00000049452	84296196	84299839	-	intronic	4	-0.6214	0.0007	0.0447	495.3867	286.0330	354.5951	431.0443	471.6875	776.6115	568.0339	675.6268
novel_circ_0006453	chr18	ENSRNOG00000027230	17182717	17196811	+	exonic	3	-1.8647	0.0000	0.0088	0	0	41.7171	0	89.8452	150.3119	47.3362	168.9067
novel_circ_0006458	chr18	ENSRNOG00000027230	17218298	17224348	+	exonic	2	-1.6080	0.0002	0.0237	0	0	0	0	89.8452	0	71.0042	120.6476
novel_circ_0006732	chr18	n/a	37284942	37308236	-	intergenic	2	-1.4723	0.0004	0.0359	0	0	0	0	67.3839	0	142.0085	48.2591
novel_circ_0006981	chr18	ENSRNOG00000033099	66581615	66632117	-	exonic	2	-1.7719	0.0001	0.0124	0	0	62.5756	0	179.6905	250.5198	118.3404	0
novel_circ_0007008	chr18	ENSRNOG00000051965	69647501	69657373	-	exonic	4	-0.8368	0.0002	0.0216	309.6167	257.4297	166.8683	226.8654	494.1488	375.7797	497.0297	410.2020
novel_circ_0007443	chr19	ENSRNOG00000013152	34292208	34312530	+	exonic	5	-1.9613	0.0000	0.0043	0	0	62.5756	0	134.7679	75.1559	118.3404	241.2953
novel_circ_0007466	chr19	ENSRNOG00000054264	38057556	38062193	+	exonic	1	-1.5934	0.0002	0.0243	0	0	0	68.0596	112.3066	100.2079	189.3446	48.2591
novel_circ_0007524	chr19	ENSRNOG00000022593	43119867	43124021	-	exonic	3	-0.6177	0.0006	0.0431	516.0278	514.8594	250.3024	612.5366	449.2262	851.7674	852.0508	796.2744
novel_circ_0007589	chr19	ENSRNOG00000019140	54803497	54822499	+	exonic	3	-1.5610	0.0001	0.0180	61.9233	57.2066	0	0	89.8452	150.3119	189.3446	120.6476
novel_circ_0007692	chr1	ENSRNOG00000011488	102636818	102660697	-	intronic	1	-1.5297	0.0003	0.0303	0	0	0	0	0	100.2079	71.0042	96.5181
novel_circ_0007968	chr1	ENSRNOG00000012716	134799595	134813696	-	exonic	3	-1.5178	0.0001	0.0113	103.2056	114.4132	0	0	179.6905	250.5198	94.6723	289.5543
novel_circ_0008366	chr1	ENSRNOG00000009812	175005899	175011736	-	exonic	2	-2.0403	0.0000	0.0049	0	0	0	0	0	175.3639	118.3404	96.5181
novel_circ_0009219	chr1	ENSRNOG00000022485	263867358	263876614	-	exonic	4	-0.7944	0.0008	0.0468	227.0522	143.0165	146.0098	249.5519	359.3810	325.6758	355.0212	361.9429
novel_circ_0009369	chr1	ENSRNOG00000017406	278580845	278594581	+	exonic	4	-0.5225	0.0000	0.0021	2559.4980	2574.2971	1960.7025	2404.7732	2785.2025	3657.5895	3029.5141	4174.4082
novel_circ_0009584	chr1	ENSRNOG00000018158	47193253	47202318	+	exonic	2	-1.4671	0.0005	0.0364	0	0	0	0	44.9226	0	47.3362	168.9067
novel_circ_0009928	chr20	ENSRNOG00000053288	20268026	20276110	+	exonic	2	-1.9011	0.0000	0.0086	0	0	0	0	67.3839	125.2599	71.0042	48.2591
novel_circ_0010635	chr2	ENSRNOG00000029209	148003673	148025257	+	exonic	2	-1.2521	0.0005	0.0373	123.8467	0	104.2927	0	269.5357	200.4159	94.6723	168.9067
novel_circ_0010697	chr2	ENSRNOG00000031247	157552304	157581187	+	exonic	3	-1.5160	0.0003	0.0315	0	0	0	0	44.9226	125.2599	0	96.5181
novel_circ_0011299	chr2	n/a	224356778	224363213	-	intergenic	4	-0.7107	0.0007	0.0454	227.0522	286.0330	229.4439	362.9846	449.2262	375.7797	639.0381	386.0724
novel_circ_0011797	chr2	ENSRNOG00000013282	3809212	3839676	+	exonic	4	-1.1399	0.0000	0.0003	247.6934	429.0495	271.1610	272.2385	651.3780	776.6115	781.0466	530.8496
novel_circ_0011799	chr2	ENSRNOG00000013282	3809212	3860857	+	exonic	5	-0.9492	0.0000	0.0057	227.0522	343.2396	271.1610	204.1789	583.9941	526.0916	473.3616	482.5905
novel_circ_0011802	chr2	ENSRNOG00000039717	38096881	38097500	-	exonic	2	-2.3923	0.0000	0.0010	0	0	0	0	202.1518	0	189.3446	96.5181

novel_circ_0012622	chr3	ENSRNOG00000004951	136874644	136893637	-	exonic	4	-1.6795	0.0001	0.0187	0	0	0	0	157.2292	0	47.3362	96.5181
novel_circ_0012942	chr3	ENSRNOG00000051498	177140836	177145025	+	exonic	3	-1.7635	0.0001	0.0142	0	0	0	0	112.3066	50.1040	71.0042	48.2591
novel_circ_0013093	chr3	n/a	25350826	25414590	-	intergenic	3	-1.5643	0.0001	0.0194	61.9233	57.2066	0	0	134.7679	100.2079	47.3362	289.5543
novel_circ_0013367	chr3	ENSRNOG00000008616	56199665	56213623	+	exonic	2	-1.5045	0.0004	0.0327	0	0	0	0	89.8452	75.1559	94.6723	0
novel_circ_0013644	chr3	ENSRNOG00000011132	94142095	94161613	-	exonic	4	-1.3467	0.0006	0.0437	41.2822	0	0	90.7462	134.7679	225.4678	118.3404	48.2591
novel_circ_0013812	chr4	ENSRNOG00000017838	118500899	118509011	-	exonic	4	-1.4976	0.0004	0.0333	0	0	0	0	67.3839	0	94.6723	96.5181
novel_circ_0013930	chr4	ENSRNOG00000010301	132075418	132084031	-	exonic	4	-1.5133	0.0003	0.0318	0	0	0	0	67.3839	100.2079	94.6723	0
novel_circ_0013932	chr4	n/a	132075418	132113888	-	intergenic	6	-0.6756	0.0008	0.0482	330.2578	371.8429	458.8878	204.1789	718.7619	701.4555	473.3616	337.8134
novel_circ_0014036	chr4	ENSRNOG00000010473	147692060	147692909	+	exonic	2	-1.3884	0.0007	0.0458	0	0	0	0	44.9226	75.1559	0	120.6476
novel_circ_0014203	chr4	ENSRNOG00000007047	171526040	171527537	-	exonic	1	-1.6327	0.0002	0.0215	0	0	0	0	247.0744	0	94.6723	0
novel_circ_0014232	chr4	ENSRNOG00000014382	177269462	177281107	-	exonic	4	-1.6210	0.0002	0.0242	0	0	0	45.3731	67.3839	150.3119	71.0042	96.5181
novel_circ_0014527	chr4	ENSRNOG00000030151	38838811	38873762	-	exonic	2	-1.6358	0.0002	0.0216	0	0	0	0	67.3839	125.2599	0	96.5181
novel_circ_0014647	chr4	ENSRNOG00000011014	58143323	58147448	-	exonic	3	-1.9746	0.0000	0.0064	0	0	0	0	134.7679	50.1040	0	193.0362
novel_circ_0014650	chr4	n/a	58367536	58408977	-	intergenic	2	-1.3944	0.0007	0.0444	0	0	0	0	179.6905	100.2079	0	0
novel_circ_0015600	chr5	ENSRNOG00000030862	14282911	14287930	-	exonic	2	-1.6307	0.0002	0.0220	0	0	0	0	0	150.3119	47.3362	96.5181
novel_circ_0015761	chr5	ENSRNOG00000013515	149960185	149965456	-	exonic	1	-1.4193	0.0006	0.0428	0	0	0	90.7462	112.3066	100.2079	71.0042	168.9067
novel_circ_0016126	chr5	n/a	37742707	37757012	+	intergenic	3	-0.7951	0.0000	0.0045	701.7978	886.7023	333.7366	362.9846	1055.6816	876.8194	946.7232	1085.8287
novel_circ_0016428	chr5	ENSRNOG00000005391	7763384	7764306	-	exonic	2	-1.4100	0.0005	0.0368	61.9233	57.2066	0	0	112.3066	50.1040	165.6766	168.9067
novel_circ_0016926	chr6	n/a	126864142	126894194	+	intergenic	7	-0.2697	0.0007	0.0445	4272.7104	3718.4291	4255.1416	4015.5176	4806.7204	4910.1886	5206.9773	4681.1283
novel_circ_0017341	chr6	ENSRNOG00000011914	28022405	28051704	+	exonic	4	-0.5106	0.0000	0.0065	1733.8535	2231.0575	1898.1269	1451.9386	2156.2858	2104.3666	2840.1695	3281.6157
novel_circ_0017915	chr6	ENSRNOG00000000618	88462487	88478487	-	exonic	2	-1.4772	0.0004	0.0354	0	0	0	0	44.9226	0	142.0085	72.3886
novel_circ_0017982	chr6	ENSRNOG00000014720	9405092	9434931	-	exonic	7	-0.4031	0.0008	0.0477	1444.8779	1258.5452	980.3513	1588.0578	1482.4465	1828.7947	1585.7613	2099.2689
novel_circ_0018128	chr7	ENSRNOG00000005159	112745540	112761127	-	exonic	4	-1.3320	0.0001	0.0131	41.2822	85.8099	41.7171	113.4327	202.1518	225.4678	213.0127	193.0362
novel_circ_0018332	chr7	ENSRNOG00000017057	127178723	127182545	+	intronic	3	-2.1346	0.0000	0.0033	0	0	0	0	44.9226	50.1040	71.0042	217.1657
novel_circ_0018705	chr7	ENSRNOG00000004026	41167311	41167751	+	exonic	1	-0.6454	0.0003	0.0313	412.8223	543.4627	271.1610	453.7308	673.8393	526.0916	733.7104	699.7563
novel_circ_0018774	chr7	ENSRNOG00000052157	52310712	52318397	-	exonic	3	-0.5855	0.0006	0.0441	392.1811	858.0990	396.3122	521.7904	741.2232	801.6635	591.7020	1085.8287
novel_circ_0019339	chr8	ENSRNOG00000015389	109243252	109288160	+	exonic	5	-0.9613	0.0009	0.0494	123.8467	85.8099	62.5756	158.8058	224.6131	275.5718	236.6808	193.0362
novel_circ_0019522	chr8	ENSRNOG00000008919	120442502	120446457	-	exonic	2	-0.9581	0.0004	0.0354	123.8467	85.8099	166.8683	136.1192	269.5357	225.4678	260.3489	337.8134

novel_circ_0019538	chr8	ENSRNOG00000009161	122129370	122142063	+	exonic	1	-2.2223	0.0000	0.0022	0	0	0	0	179.6905	50.1040	118.3404	48.2591
novel_circ_0019835	chr8	ENSRNOG00000032180	39790058	39807952	-	exonic	2	-1.4849	0.0004	0.0346	0	0	0	0	112.3066	0	71.0042	72.3886
novel_circ_0019857	chr8	ENSRNOG00000029876	456818	509712	+	exonic	1	-1.0644	0.0000	0.0102	206.4111	171.6198	146.0098	136.1192	291.9970	450.9357	260.3489	482.5905
novel_circ_0020014	chr8	ENSRNOG00000009170	59022286	59025874	-	exonic	2	-0.6209	0.0003	0.0293	557.3101	486.2561	479.7464	476.4173	583.9941	776.6115	639.0381	1134.0878
novel_circ_0020807	chr9	ENSRNOG00000015439	112406659	112412113	+	exonic	4	-1.0675	0.0007	0.0446	61.9233	228.8264	41.7171	68.0596	112.3066	350.7278	284.0169	168.9067
novel_circ_0021517	chr9	ENSRNOG00000045587	7651928	7660794	+	intronic	2	-1.3751	0.0008	0.0469	0	0	0	0	202.1518	75.1559	0	0
novel_circ_0000145	chr10	ENSRNOG00000020488	15544775	15555433	+	exonic	3	2.3281	0.0000	0.0013	61.9233	228.8264	62.5756	90.7462	0	0	0	0
novel_circ_0000953	chr10	ENSRNOG00000060561	74343129	74360926	-	exonic	1	1.7811	0.0001	0.0133	61.9233	0	125.1512	136.1192	0	0	0	0
novel_circ_0001364	chr11	ENSRNOG00000006997	24547989	24550752	-	exonic	2	0.6090	0.0003	0.0277	701.7978	972.5122	667.4732	839.4020	381.8423	425.8837	615.3701	603.2382
novel_circ_0001517	chr11	ENSRNOG00000027992	37020528	37053225	-	exonic	1	1.6875	0.0001	0.0183	61.9233	0	62.5756	181.4923	0	0	0	0
novel_circ_0001623	chr11	ENSRNOG00000056716	62059610	62067655	-	exonic	4	1.3256	0.0003	0.0295	123.8467	114.4132	187.7268	204.1789	44.9226	50.1040	94.6723	0
novel_circ_0002032	chr12	ENSRNOG00000029986	1793317	1793869	-	exonic	1	2.1199	0.0000	0.0035	123.8467	0	166.8683	113.4327	0	0	0	0
novel_circ_0002592	chr13	ENSRNOG00000032206	22121991	22151171	+	exonic	1	1.3721	0.0008	0.0483	61.9233	0	104.2927	68.0596	0	0	0	0
novel_circ_0002608	chr13	n/a	25113372	25115431	-	intergenic	4	1.5473	0.0003	0.0326	185.7700	171.6198	0	68.0596	0	50.1040	0	0
novel_circ_0002856	chr13	ENSRNOG00000011063	56114437	56183125	+	exonic	6	0.6126	0.0008	0.0467	433.4634	1029.7188	625.7561	612.5366	449.2262	350.7278	402.3573	482.5905
novel_circ_0003047	chr13	ENSRNOG00000042492	76979426	77004245	+	exonic	5	1.2600	0.0002	0.0209	206.4111	114.4132	229.4439	340.2981	0	125.2599	94.6723	96.5181
novel_circ_0003280	chr13	ENSRNOG00000021497	95120751	95208329	-	exonic	3	1.6780	0.0001	0.0164	61.9233	143.0165	166.8683	113.4327	67.3839	0	0	0
novel_circ_0003721	chr14	ENSRNOG00000002039	2980818	2988823	+	exonic	2	1.3399	0.0006	0.0413	144.4878	228.8264	83.4341	90.7462	67.3839	75.1559	0	0
novel_circ_0004037	chr14	ENSRNOG00000006400	79407475	79421836	+	exonic	3	1.3789	0.0008	0.0474	82.5645	0	83.4341	68.0596	0	0	0	0
novel_circ_0004188	chr14	ENSRNOG00000059479	87328007	87357732	+	exonic	4	0.5444	0.0005	0.0373	1135.2612	1229.9419	1147.2196	2518.2059	763.6846	1177.4432	852.0508	1278.8649
novel_circ_0004267	chr15	ENSRNOG00000037886	104974439	104976461	+	intronic	2	2.0217	0.0000	0.0053	82.5645	114.4132	104.2927	45.3731	0	0	0	0
novel_circ_0004272	chr15	n/a	106432817	106434369	+	intergenic	2	1.5215	0.0003	0.0319	165.1289	143.0165	41.7171	90.7462	0	0	71.0042	0
novel_circ_0004615	chr15	ENSRNOG00000014509	41493484	41507648	+	exonic	4	1.4472	0.0005	0.0390	0	85.8099	104.2927	68.0596	0	0	0	0
novel_circ_0007281	chr19	ENSRNOG00000014658	20282664	20292462	+	exonic	1	1.3614	0.0006	0.0429	82.5645	143.0165	146.0098	113.4327	67.3839	50.1040	0	0
novel_circ_0007599	chr19	ENSRNOG00000015150	55882754	55886776	+	exonic	3	1.1650	0.0007	0.0446	185.7700	171.6198	271.1610	272.2385	247.0744	0	0	72.3886
novel_circ_0007740	chr1	ENSRNOG00000010803	112926909	112929030	-	exonic	3	1.2426	0.0009	0.0499	123.8467	85.8099	146.0098	181.4923	0	50.1040	47.3362	72.3886
novel_circ_0007936	chr1	ENSRNOG00000014187	128978103	128978649	+	exonic	1	3.2563	0.0000	0.0000	268.3345	143.0165	208.5854	158.8058	0	0	0	0
novel_circ_0008023	chr1	ENSRNOG00000017120	141053113	141055207	+	exonic	2	1.2535	0.0000	0.0030	371.5400	286.0330	604.8976	340.2981	157.2292	125.2599	236.6808	96.5181

novel_circ_0008045	chr1	ENSRNOG00000019005	143143054	143143524	+	exonic	2	1.5446	0.0004	0.0329	82.5645	0	83.4341	249.5519	0	50.1040	0	0
novel_circ_0008194	chr1	ENSRNOG00000024194	162639899	162643789	+	exonic	1	1.8040	0.0000	0.0047	309.6167	171.6198	187.7268	113.4327	134.7679	0	0	0
novel_circ_0008578	chr1	n/a	195009268	195011851	-	intergenic	5	0.9295	0.0009	0.0499	309.6167	514.8594	187.7268	317.6116	67.3839	350.7278	165.6766	48.2591
novel_circ_0008667	chr1	ENSRNOG00000016374	200648163	200672411	-	exonic	3	1.6677	0.0001	0.0198	41.2822	114.4132	125.1512	136.1192	0	50.1040	0	0
novel_circ_0008668	chr1	ENSRNOG00000016374	200671012	200672411	-	exonic	2	1.9646	0.0000	0.0044	41.2822	57.2066	208.5854	340.2981	0	75.1559	0	0
novel_circ_0008735	chr1	ENSRNOG00000016227	209778385	209797179	+	exonic	3	1.5979	0.0002	0.0260	82.5645	85.8099	83.4341	136.1192	0	50.1040	0	0
novel_circ_0009053	chr1	n/a	250612596	250630729	-	intergenic	3	1.3791	0.0008	0.0474	61.9233	114.4132	0	68.0596	0	0	0	0
novel_circ_0009321	chr1	ENSRNOG00000011313	270300441	270364139	-	intronic	3	1.3849	0.0009	0.0499	123.8467	114.4132	229.4439	0	44.9226	0	47.3362	0
novel_circ_0009467	chr1	n/a	31118289	31122188	-	intergenic	1	1.6062	0.0002	0.0239	0	143.0165	62.5756	90.7462	0	0	0	0
novel_circ_0009709	chr1	ENSRNOG00000032699	73746710	73747791	-	exonic	2	1.4749	0.0002	0.0220	123.8467	114.4132	208.5854	136.1192	67.3839	0	71.0042	0
novel_circ_0009895	chr20	ENSRNOG00000001303	14335984	14342412	-	exonic	3	1.4892	0.0004	0.0344	82.5645	0	41.7171	136.1192	0	0	0	0
novel_circ_0010038	chr20	n/a	3149070	3169368	+	intergenic	1	1.7402	0.0001	0.0153	123.8467	0	146.0098	45.3731	0	0	0	0
novel_circ_0010044	chr20	n/a	31723857	31736111	-	intergenic	1	1.8959	0.0000	0.0078	1052.6968	1258.5452	0	1088.9539	0	0	0	0
novel_circ_0010196	chr20	ENSRNOG00000000327	50659750	50687868	+	exonic	5	0.8657	0.0000	0.0017	1919.6235	1029.7188	771.7659	1315.8193	583.9941	551.1436	757.3785	820.4039
novel_circ_0010281	chr20	ENSRNOG00000038883	7363739	7372069	-	exonic	2	0.4631	0.0002	0.0262	1919.6235	1630.3881	1501.8147	1792.2367	1347.6786	977.0273	970.3912	1616.6783
novel_circ_0010541	chr2	ENSRNOG00000012385	140524187	140529795	+	exonic	4	1.7516	0.0001	0.0117	227.0522	143.0165	62.5756	90.7462	0	75.1559	0	0
novel_circ_0010620	chr2	n/a	145485715	145513090	-	intergenic	4	0.8845	0.0005	0.0394	412.8223	257.4297	333.7366	340.2981	291.9970	75.1559	165.6766	144.7772
novel_circ_0010648	chr2	ENSRNOG00000010680	149304804	149315205	+	exonic	2	1.5854	0.0002	0.0259	82.5645	57.2066	41.7171	68.0596	0	0	0	0
novel_circ_0010732	chr2	n/a	170837749	170842114	+	intergenic	2	1.2309	0.0001	0.0115	185.7700	228.8264	208.5854	589.8500	89.8452	100.2079	142.0085	120.6476
novel_circ_0010988	chr2	ENSRNOG00000015553	189723523	189726207	+	exonic	1	1.7611	0.0001	0.0133	103.2056	114.4132	62.5756	158.8058	0	50.1040	0	0
novel_circ_0010989	chr2	ENSRNOG00000015553	189723523	189729005	+	exonic	2	0.8049	0.0007	0.0462	227.0522	486.2561	354.5951	362.9846	179.6905	200.4159	165.6766	217.1657
novel_circ_0011096	chr2	ENSRNOG00000018946	205814397	205815848	+	exonic	3	1.3239	0.0005	0.0405	206.4111	143.0165	166.8683	158.8058	0	0	189.3446	0
novel_circ_0011118	chr2	ENSRNOG00000019885	206563071	206570833	-	exonic	3	1.1542	0.0009	0.0496	185.7700	171.6198	187.7268	113.4327	89.8452	100.2079	0	48.2591
novel_circ_0011143	chr2	ENSRNOG00000014002	207356977	207360278	+	exonic	3	1.8704	0.0000	0.0096	123.8467	0	125.1512	90.7462	0	0	0	0
novel_circ_0011215	chr2	n/a	213064652	213065395	-	intergenic	1	2.0829	0.0000	0.0038	0	600.6693	354.5951	657.9097	0	0	0	0
novel_circ_0012671	chr3	ENSRNOG00000025141	141105391	141107311	+	exonic	1	2.4049	0.0000	0.0009	103.2056	200.2231	208.5854	0	0	0	0	0
novel_circ_0012760	chr3	ENSRNOG00000019339	15225635	15229198	-	intronic	4	1.4475	0.0005	0.0389	61.9233	0	146.0098	45.3731	0	0	0	0
novel_circ_0012856	chr3	ENSRNOG00000017824	161474538	161487731	-	exonic	2	1.8666	0.0000	0.0097	0	114.4132	83.4341	158.8058	0	0	0	0

novel_circ_0013035	chr3	ENSRNOG00000010150	21878520	21904269	-	exonic	7	0.7280	0.0000	0.0103	722.4390	915.3056	667.4732	794.0289	404.3036	475.9877	331.3531	603.2382
novel_circ_0013064	chr3	ENSRNOG00000014493	23315950	23329228	-	exonic	2	1.4910	0.0004	0.0347	41.2822	85.8099	62.5756	45.3731	0	0	0	0
novel_circ_0013083	chr3	n/a	25268500	25316608	-	intergenic	3	1.4884	0.0004	0.0345	61.9233	0	104.2927	90.7462	0	0	0	0
novel_circ_0013129	chr3	ENSRNOG00000031038	29584277	29597359	-	intronic	2	2.0280	0.0000	0.0019	268.3345	114.4132	187.7268	136.1192	89.8452	0	0	0
novel_circ_0013218	chr3	ENSRNOG00000057740	45033237	45088995	-	exonic	3	1.5928	0.0000	0.0018	227.0522	228.8264	271.1610	499.1039	89.8452	150.3119	0	96.5181
novel_circ_0013445	chr3	ENSRNOG00000024457	63010096	63058267	-	exonic	4	1.9266	0.0000	0.0078	61.9233	114.4132	104.2927	45.3731	0	0	0	0
novel_circ_0013708	chr4	ENSRNOG00000013431	10775671	10777395	+	intronic	3	0.9796	0.0007	0.0452	371.5400	371.8429	146.0098	317.6116	224.6131	175.3639	0	144.7772
novel_circ_0014210	chr4	ENSRNOG00000008747	174700909	174704764	+	exonic	4	1.4008	0.0007	0.0445	103.2056	57.2066	83.4341	0	0	0	0	0
novel_circ_0014784	chr4	ENSRNOG00000012826	64894604	64899587	-	exonic	1	1.4903	0.0004	0.0361	41.2822	143.0165	187.7268	68.0596	0	0	0	72.3886
novel_circ_0015528	chr5	ENSRNOG00000008560	138375786	138395761	-	exonic	3	1.1617	0.0006	0.0437	227.0522	171.6198	208.5854	113.4327	0	100.2079	47.3362	120.6476
novel_circ_0015544	chr5	ENSRNOG00000061851	138622349	138622674	+	exonic	1	0.8701	0.0001	0.0129	598.5923	457.6528	542.3220	431.0443	359.3810	275.5718	260.3489	168.9067
novel_circ_0015668	chr5	ENSRNOG00000012397	144931943	144942704	-	exonic	1	1.3043	0.0006	0.0442	123.8467	200.2231	146.0098	204.1789	0	0	189.3446	0
novel_circ_0015981	chr5	ENSRNOG00000022694	169281335	169285425	+	exonic	3	1.4279	0.0006	0.0412	0	57.2066	62.5756	136.1192	0	0	0	0
novel_circ_0017063	chr6	ENSRNOG00000010330	136059671	136081622	+	exonic	3	1.4933	0.0004	0.0344	61.9233	85.8099	41.7171	45.3731	0	0	0	0
novel_circ_0017497	chr6	ENSRNOG00000008479	4298604	4315682	-	exonic	6	0.7635	0.0000	0.0022	1135.2612	1344.3551	1042.9269	1270.4462	920.9137	651.3516	781.0466	386.0724
novel_circ_0017823	chr6	ENSRNOG00000046256	76491314	76492176	-	exonic	1	1.6660	0.0001	0.0194	103.2056	0	250.3024	0	0	0	0	0
novel_circ_0018274	chr7	n/a	123600186	123617087	-	intergenic	2	2.8244	0.0000	0.0000	144.4878	686.4792	292.0195	249.5519	0	0	0	96.5181
novel_circ_0018334	chr7	ENSRNOG00000017057	127189205	127223231	+	exonic	4	1.4093	0.0008	0.0480	165.1289	85.8099	83.4341	68.0596	0	75.1559	0	0
novel_circ_0018666	chr7	n/a	32955132	32982696	-	intergenic	5	1.0784	0.0003	0.0288	165.1289	228.8264	396.3122	226.8654	157.2292	125.2599	71.0042	72.3886
novel_circ_0018801	chr7	ENSRNOG00000004077	55148729	55151796	+	exonic	1	2.4181	0.0000	0.0009	0	200.2231	208.5854	113.4327	0	0	0	0
novel_circ_0018890	chr7	ENSRNOG00000004013	64707677	64722547	+	exonic	5	0.9164	0.0005	0.0376	330.2578	457.6528	500.6049	272.2385	202.1518	275.5718	284.0169	0
novel_circ_0019066	chr7	ENSRNOG00000004201	78347305	78415345	+	intronic	2	1.9222	0.0000	0.0079	41.2822	85.8099	83.4341	113.4327	0	0	0	0
novel_circ_0019343	chr8	ENSRNOG00000015389	109302671	109306182	+	exonic	3	2.1230	0.0000	0.0035	41.2822	57.2066	166.8683	113.4327	0	0	0	0
novel_circ_0019725	chr8	ENSRNOG00000015189	23851918	23884848	+	exonic	5	1.2070	0.0007	0.0447	123.8467	143.0165	333.7366	90.7462	112.3066	0	47.3362	72.3886
novel_circ_0020226	chr8	ENSRNOG00000010176	69139839	69143732	-	exonic	3	1.7434	0.0001	0.0153	41.2822	143.0165	62.5756	45.3731	0	0	0	0
novel_circ_0021014	chr9	ENSRNOG00000011000	28529456	28552133	-	exonic	3	0.7067	0.0007	0.0444	392.1811	543.4627	375.4537	521.7904	224.6131	300.6238	284.0169	265.4248
novel_circ_0021355	chr9	ENSRNOG00000017258	66796279	66804771	-	exonic	2	1.6631	0.0001	0.0199	0	85.8099	83.4341	136.1192	0	0	0	0
novel_circ_0021480	chr9	ENSRNOG00000011841	73429544	73434017	+	exonic	1	2.0686	0.0000	0.0044	144.4878	57.2066	62.5756	90.7462	0	0	0	0

novel_circ_0021642	chr9	ENSRNOG00000023577	94463646	94481167	+	exonic	7	0.2774	0.0003	0.0301	10650.8143	12156.4028	12056.2346	13679.9837	11006.0421	9619.9614	10153.6058	8927.9251
novel_circ_0021718	chrX	ENSRNOG00000003261	10567594	10606963	-	exonic	2	1.4986	0.0004	0.0339	41.2822	85.8099	41.7171	68.0596	0	0	0	0
novel_circ_0021721	chrX	n/a	105805716	105810447	-	intergenic	1	1.7127	0.0000	0.0084	144.4878	257.4297	62.5756	181.4923	44.9226	0	71.0042	0
novel_circ_0021722	chrX	n/a	105805716	105821741	-	intergenic	2	0.7985	0.0005	0.0398	227.0522	457.6528	792.6244	567.1635	291.9970	225.4678	449.6935	144.7772
novel_circ_0021865	chrX	n/a	13905155	13917538	+	intergenic	4	1.1006	0.0001	0.0116	350.8989	486.2561	625.7561	158.8058	202.1518	175.3639	236.6808	72.3886

**Day-28 circRNA**	**Chr**	**Gene ID**	**Start**	**End**	**Strand**	**Feature**	**Count**	**log2(FC)**	***P* Value**	***Q* Value**	**ICH1**	**ICH2**	**ICH3**	**ICH4**	**Sham1**	**Sham2**	**Sham3**	**Sham4**
novel_circ_0000323	chr10	ENSRNOG00000004563	37167399	37172772	-	exonic	4	-1.2175	0.0001	0.0151	0	46.6190	0	0	80.2414	127.2912	89.7562	62.2675
novel_circ_0001023	chr10	ENSRNOG00000002749	81798930	81799587	+	exonic	2	-1.0477	0.0005	0.0365	0	46.6190	0	45.5512	80.2414	76.3747	128.2232	108.9681
novel_circ_0001041	chr10	ENSRNOG00000009275	85109545	85112742	-	exonic	2	-0.9274	0.0005	0.0379	0	0	0	0	48.1448	76.3747	51.2893	31.1337
novel_circ_0001173	chr10	ENSRNOG00000006285	93451476	93465700	+	exonic	3	-0.9507	0.0008	0.0483	50.1362	46.6190	0	60.7349	176.5310	101.8330	89.7562	124.5349
novel_circ_0001319	chr11	ENSRNOG00000001573	15457478	15474826	+	exonic	3	-1.0842	0.0002	0.0212	0	46.6190	83.8153	91.1023	192.5793	254.5825	128.2232	77.8343
novel_circ_0001533	chr11	n/a	42050183	42050847	+	intergenic	1	-0.6828	0.0006	0.0423	267.3931	349.6422	0	349.2256	353.0620	432.7902	538.5375	451.4392
novel_circ_0001582	chr11	ENSRNOG00000001982	51068883	51088607	-	exonic	2	-0.8784	0.0007	0.0453	0	0	0	0	80.2414	76.3747	64.1116	0
novel_circ_0002291	chr12	ENSRNOG00000001120	43501059	43510832	-	exonic	3	-0.9913	0.0008	0.0485	0	0	125.7229	0	160.4827	76.3747	64.1116	62.2675
novel_circ_0002890	chr13	ENSRNOG00000013312	57409195	57411487	+	exonic	2	-1.0539	0.0004	0.0352	50.1362	0	0	0	112.3379	101.8330	64.1116	62.2675
novel_circ_0003140	chr13	ENSRNOG00000003078	83634262	83638054	-	exonic	2	-1.2363	0.0000	0.0095	0	0	0	0	96.2896	101.8330	25.6446	62.2675
novel_circ_0003492	chr14	n/a	113552475	113565546	-	intergenic	3	-0.9269	0.0005	0.0379	0	0	0	0	32.0965	76.3747	51.2893	46.7006
novel_circ_0003563	chr14	ENSRNOG00000002053	14455619	14467922	-	exonic	1	-1.2575	0.0000	0.0086	0	0	0	0	80.2414	76.3747	76.9339	46.7006
novel_circ_0003866	chr14	ENSRNOG00000025494	43558567	43581074	-	exonic	4	-1.1482	0.0001	0.0142	0	0	0	0	0	178.2077	76.9339	46.7006
novel_circ_0004178	chr14	ENSRNOG00000005130	86431782	86438230	+	intronic	3	-1.0727	0.0002	0.0263	116.9845	0	0	45.5512	48.1448	178.2077	166.6902	217.9361
novel_circ_0004335	chr15	n/a	110036613	110053440	-	intergenic	2	-0.9094	0.0006	0.0400	0	0	0	0	64.1931	101.8330	64.1116	0
novel_circ_0004820	chr15	ENSRNOG00000047106	61665893	61683055	+	exonic	3	-1.1115	0.0001	0.0152	0	69.9284	83.8153	136.6535	272.8206	127.2912	333.3803	93.4012
novel_circ_0005172	chr16	n/a	39367334	39375826	-	intergenic	4	-1.0576	0.0001	0.0197	167.1207	46.6190	0	30.3674	160.4827	178.2077	243.6241	171.2355
novel_circ_0005323	chr16	ENSRNOG00000013061	56313380	56350456	+	exonic	3	-1.2397	0.0000	0.0093	0	0	0	0	48.1448	50.9165	115.4009	62.2675
novel_circ_0005594	chr16	ENSRNOG00000016483	84806783	84842321	-	exonic	3	-0.9941	0.0006	0.0409	0	0	0	30.3674	144.4345	0	51.2893	108.9681
novel_circ_0005601	chr16	n/a	85581043	85595037	+	intergenic	1	-1.8085	0.0000	0.0003	0	0	0	0	80.2414	178.2077	102.5786	77.8343
novel_circ_0005867	chr17	n/a	51972800	52027612	+	intergenic	4	-0.8207	0.0004	0.0343	150.4086	116.5474	209.5382	91.1023	240.7241	203.6660	256.4464	326.9042
novel_circ_0006133	chr17	ENSRNOG00000017505	718533	739440	+	exonic	4	-0.5938	0.0003	0.0298	484.6500	326.3327	586.7069	318.8582	577.7378	687.3727	512.8928	747.2096

novel_circ_0006352	chr17	ENSRNOG00000039807	9613029	9614622	+	exonic	1	-1.6867	0.0000	0.0007	0	0	0	0	0	127.2912	102.5786	233.5030
novel_circ_0006461	chr18	ENSRNOG00000027230	17224243	17264715	+	exonic	4	-0.7772	0.0006	0.0397	284.1052	93.2379	167.6305	121.4698	224.6758	483.7067	243.6241	342.4711
novel_circ_0006677	chr18	ENSRNOG00000013920	32069616	32094303	-	exonic	5	-1.0647	0.0004	0.0341	0	0	0	60.7349	48.1448	152.7495	102.5786	77.8343
novel_circ_0007444	chr19	ENSRNOG00000013152	34292208	34346224	+	exonic	5	-0.9801	0.0005	0.0376	66.8483	69.9284	0	45.5512	128.3862	178.2077	153.8679	93.4012
novel_circ_0007447	chr19	ENSRNOG00000013152	34298522	34312530	+	exonic	4	-0.8830	0.0002	0.0261	200.5448	93.2379	125.7229	75.9186	320.9655	229.1242	205.1571	280.2036
novel_circ_0007851	chr1	n/a	122605869	122678378	-	intergenic	3	-1.0451	0.0001	0.0199	0	0	0	0	192.5793	381.8737	0	326.9042
novel_circ_0008137	chr1	ENSRNOG00000009664	157422217	157440238	-	exonic	4	-0.6382	0.0007	0.0443	434.5138	233.0948	335.2611	273.3070	513.5447	229.1242	653.9384	591.5410
novel_circ_0008495	chr1	ENSRNOG00000027405	188436735	188437895	+	exonic	3	-0.8798	0.0008	0.0457	0	0	0	0	32.0965	50.9165	64.1116	46.7006
novel_circ_0008933	chr1	ENSRNOG00000025539	235759863	235764346	-	exonic	4	-0.8138	0.0002	0.0213	150.4086	279.7138	167.6305	167.0210	272.8206	280.0407	384.6696	467.0060
novel_circ_0009073	chr1	ENSRNOG00000012122	25216048	25284394	+	exonic	3	-1.1073	0.0003	0.0272	50.1362	0	0	0	80.2414	76.3747	115.4009	77.8343
novel_circ_0009393	chr1	ENSRNOG00000017406	278679021	278749829	+	exonic	7	-0.5212	0.0003	0.0275	1420.5258	792.5223	1424.8596	501.0629	1364.1032	1782.0774	1371.9884	1214.2157
novel_circ_0009401	chr1	ENSRNOG00000017406	278698311	278749829	+	exonic	5	-0.5700	0.0000	0.0034	1353.6775	1375.2593	2095.3818	1138.7792	1797.4066	2163.9511	1589.9678	2833.1699
novel_circ_0009446	chr1	ENSRNOG00000013323	28362166	28375861	+	exonic	2	-1.0468	0.0005	0.0369	0	69.9284	0	0	64.1931	101.8330	64.1116	124.5349
novel_circ_0009684	chr1	n/a	66934988	66944229	-	intergenic	2	-0.9924	0.0002	0.0260	0	0	0	0	304.9172	76.3747	0	171.2355
novel_circ_0009822	chr1	ENSRNOG00000052814	91873486	91879026	+	exonic	5	-0.9452	0.0004	0.0324	100.2724	139.8569	0	45.5512	192.5793	229.1242	179.5125	140.1018
novel_circ_0009887	chr20	ENSRNOG00000001237	13906483	13912191	+	exonic	1	-1.0310	0.0002	0.0245	0	0	0	0	112.3379	50.9165	25.6446	46.7006
novel_circ_0009993	chr20	ENSRNOG00000042916	27535431	27544812	-	exonic	4	-0.7212	0.0002	0.0248	250.6810	396.2612	125.7229	273.3070	513.5447	356.4155	589.8268	373.6048
novel_circ_0010178	chr20	ENSRNOG00000000316	47860619	47896656	-	exonic	3	-0.5350	0.0000	0.0086	869.0275	885.7602	712.4298	1093.2281	1299.9101	1145.6212	1320.6991	1432.1518
novel_circ_0010406	chr2	ENSRNOG00000033134	11729893	11747274	+	exonic	2	-0.5680	0.0002	0.0223	768.7551	489.4991	628.6145	440.3280	834.5102	1171.0794	705.2277	778.3434
novel_circ_0010451	chr2	ENSRNOG00000038436	123576588	123600232	+	exonic	5	-1.0700	0.0003	0.0270	100.2724	0	0	45.5512	128.3862	76.3747	166.6902	171.2355
novel_circ_0010818	chr2	ENSRNOG00000054204	179624045	179638761	-	exonic	3	-1.0289	0.0006	0.0402	0	69.9284	0	0	64.1931	152.7495	76.9339	62.2675
novel_circ_0010941	chr2	ENSRNOG00000020386	188281554	188301124	+	exonic	1	-1.0804	0.0003	0.0291	33.4241	0	0	0	64.1931	76.3747	102.5786	62.2675
novel_circ_0011109	chr2	ENSRNOG00000019785	206472094	206477723	+	exonic	3	-1.2234	0.0000	0.0101	0	0	0	0	64.1931	50.9165	76.9339	77.8343
novel_circ_0011114	chr2	ENSRNOG00000019885	206534103	206536360	-	exonic	2	-0.6614	0.0000	0.0106	635.0586	652.6654	335.2611	349.2256	690.0757	789.2057	884.7402	825.0440
novel_circ_0011120	chr2	ENSRNOG00000019885	206567017	206570833	-	exonic	2	-1.5546	0.0000	0.0017	0	0	0	0	96.2896	101.8330	64.1116	93.4012
novel_circ_0011299	chr2	n/a	224356778	224363213	-	intergenic	4	-1.0200	0.0003	0.0296	367.6655	93.2379	0	258.1233	561.6896	381.8737	769.3393	420.3054
novel_circ_0011322	chr2	ENSRNOG00000015353	227687640	227702229	+	exonic	3	-1.1679	0.0001	0.0198	83.5603	0	0	0	80.2414	254.5825	76.9339	77.8343
novel_circ_0011441	chr2	ENSRNOG00000009882	242126333	242151045	+	exonic	3	-1.1726	0.0001	0.0130	0	0	0	0	48.1448	101.8330	51.2893	62.2675

novel_circ_0011811	chr2	n/a	39042495	39042852	-	intergenic	1	-1.0492	0.0003	0.0286	116.9845	69.9284	0	0	256.7724	152.7495	89.7562	124.5349
novel_circ_0012262	chr3	ENSRNOG00000005183	111513947	111523935	+	exonic	3	-0.9085	0.0001	0.0140	116.9845	139.8569	125.7229	212.5721	385.1586	356.4155	243.6241	295.7705
novel_circ_0012325	chr3	ENSRNOG00000012798	113149411	113149914	+	exonic	2	-0.9975	0.0003	0.0278	0	0	0	0	0	127.2912	64.1116	62.2675
novel_circ_0012356	chr3	ENSRNOG00000013442	11394658	11396393	+	exonic	2	-0.9347	0.0006	0.0413	0	116.5474	167.6305	60.7349	96.2896	127.2912	179.5125	311.3373
novel_circ_0012425	chr3	ENSRNOG00000026277	121519926	121533453	+	exonic	1	-1.2441	0.0000	0.0092	0	0	0	0	64.1931	76.3747	89.7562	46.7006
novel_circ_0012482	chr3	ENSRNOG00000025286	123819851	123833222	+	exonic	2	-0.9834	0.0004	0.0341	33.4241	93.2379	83.8153	30.3674	128.3862	203.6660	102.5786	155.6687
novel_circ_0013601	chr3	ENSRNOG00000015396	8587700	8589420	+	exonic	2	-0.9484	0.0005	0.0376	150.4086	46.6190	0	60.7349	112.3379	127.2912	166.6902	311.3373
novel_circ_0014083	chr4	ENSRNOG00000009956	152526402	152529177	-	exonic	2	-1.0499	0.0004	0.0354	0	0	0	45.5512	80.2414	76.3747	128.2232	46.7006
novel_circ_0014625	chr4	ENSRNOG00000031855	5644286	5652694	+	exonic	4	-1.0295	0.0006	0.0402	0	46.6190	0	45.5512	32.0965	152.7495	128.2232	93.4012
novel_circ_0015325	chr5	ENSRNOG00000005730	12156801	12172003	-	exonic	2	-0.4958	0.0002	0.0251	718.6189	699.2844	670.5222	789.5536	962.8964	941.9552	1218.1205	902.8783
novel_circ_0015394	chr5	n/a	127620258	127635191	-	intergenic	3	-0.9094	0.0001	0.0180	167.1207	116.5474	209.5382	75.9186	337.0137	330.9572	217.9795	249.0699
novel_circ_0015443	chr5	ENSRNOG00000010299	129003128	129009597	+	exonic	3	-1.1638	0.0001	0.0132	0	0	0	0	160.4827	0	51.2893	77.8343
novel_circ_0015592	chr5	n/a	142152200	142154418	+	intergenic	1	-0.3822	0.0001	0.0144	3542.9584	2517.4238	3310.7032	2338.2933	3193.6064	4327.9022	3231.2249	4062.9524
novel_circ_0015668	chr5	ENSRNOG00000012397	144931943	144942704	-	exonic	1	-1.6915	0.0000	0.0007	0	0	0	0	0	101.8330	115.4009	249.0699
novel_circ_0015812	chr5	ENSRNOG00000022372	154919348	154931756	-	exonic	5	-1.3363	0.0000	0.0057	0	0	0	0	96.2896	76.3747	64.1116	62.2675
novel_circ_0016248	chr5	ENSRNOG00000013078	60390690	60393196	+	exonic	3	-1.0334	0.0001	0.0145	150.4086	116.5474	0	75.9186	144.4345	330.9572	192.3348	295.7705
novel_circ_0016957	chr6	ENSRNOG00000004827	129629702	129632684	+	exonic	1	-0.8933	0.0006	0.0425	0	0	0	0	32.0965	76.3747	0	124.5349
novel_circ_0016966	chr6	ENSRNOG00000005274	129864988	129890111	+	exonic	4	-0.8355	0.0008	0.0468	150.4086	0	167.6305	151.8372	240.7241	178.2077	166.6902	373.6048
novel_circ_0017108	chr6	ENSRNOG00000004968	144343081	144347969	+	exonic	3	-0.8199	0.0001	0.0149	150.4086	163.1664	293.3534	303.6745	417.2551	560.0815	435.9589	280.2036
novel_circ_0017111	chr6	ENSRNOG00000050220	14450091	14490352	-	exonic	4	-0.3387	0.0007	0.0434	1704.6309	1701.5920	1424.8596	1715.7607	1813.4549	1934.8269	2064.3937	2350.5970
novel_circ_0017236	chr6	ENSRNOG00000006340	22373411	22409230	+	exonic	3	-1.0362	0.0005	0.0392	33.4241	0	0	30.3674	112.3379	50.9165	89.7562	93.4012
novel_circ_0017318	chr6	n/a	27858537	27881618	+	intergenic	3	-0.9384	0.0006	0.0398	0	116.5474	83.8153	91.1023	144.4345	280.0407	166.6902	108.9681
novel_circ_0017606	chr6	ENSRNOG00000004186	54570722	54585265	+	exonic	4	-0.8123	0.0009	0.0495	150.4086	116.5474	0	121.4698	176.5310	254.5825	230.8018	217.9361
novel_circ_0017710	chr6	ENSRNOG00000004067	64847536	64861750	+	exonic	4	-0.8505	0.0005	0.0360	66.8483	116.5474	209.5382	106.2861	160.4827	330.9572	192.3348	264.6367
novel_circ_0017764	chr6	ENSRNOG00000004841	73658708	73659072	+	exonic	1	-0.8934	0.0005	0.0387	100.2724	93.2379	0	136.6535	272.8206	127.2912	141.0455	280.2036
novel_circ_0018159	chr7	ENSRNOG00000007916	114490397	114499158	-	exonic	5	-0.6275	0.0003	0.0276	417.8017	489.4991	293.3534	258.1233	593.7861	509.1650	525.7152	653.8084
novel_circ_0018270	chr7	ENSRNOG00000007003	123336675	123349326	+	exonic	3	-1.2803	0.0000	0.0077	0	0	0	0	80.2414	76.3747	51.2893	77.8343
novel_circ_0018315	chr7	ENSRNOG00000014637	126256292	126283984	+	exonic	3	-1.1501	0.0001	0.0170	50.1362	116.5474	0	0	144.4345	178.2077	76.9339	202.3693

novel_circ_0019279	chr8	ENSRNOG00000011241	104157474	104168030	+	exonic	3	-1.1318	0.0001	0.0156	0	0	0	0	48.1448	76.3747	25.6446	108.9681
novel_circ_0019418	chr8	ENSRNOG00000014576	115811799	115835862	-	exonic	2	-0.9093	0.0006	0.0407	0	0	0	0	64.1931	50.9165	25.6446	62.2675
novel_circ_0019788	chr8	ENSRNOG00000008709	33279133	33335423	+	exonic	7	-0.3750	0.0003	0.0310	2105.7205	2097.8532	2137.2894	1320.9839	2343.0479	2316.7006	2820.9106	2008.1259
novel_circ_0020499	chr8	ENSRNOG00000005934	84434783	84496011	-	exonic	5	-0.6023	0.0000	0.0013	2540.2343	1841.4489	1676.3054	1609.4746	2294.9031	4073.3198	2667.0428	2661.9343
novel_circ_0020500	chr8	ENSRNOG00000005934	84434783	84496086	-	exonic	4	-0.8168	0.0000	0.0064	451.2258	396.2612	377.1687	151.8372	625.8827	814.6640	538.5375	498.1398
novel_circ_0020503	chr8	ENSRNOG00000005934	84443615	84496011	-	exonic	6	-0.4909	0.0001	0.0185	1069.5723	885.7602	754.3374	728.8187	1139.4274	1451.1202	1141.1866	1136.3813
novel_circ_0020504	chr8	ENSRNOG00000005934	84443615	84496086	-	exonic	5	-1.0254	0.0000	0.0011	300.8172	163.1664	377.1687	167.0210	465.3999	585.5397	551.3598	467.0060
novel_circ_0020713	chr9	n/a	110314145	110314527	-	intergenic	2	-0.9694	0.0007	0.0455	33.4241	0	0	0	128.3862	50.9165	0	140.1018
novel_circ_0020749	chr9	ENSRNOG00000015898	111607927	111653491	+	exonic	3	-0.9464	0.0003	0.0265	183.8327	139.8569	0	75.9186	272.8206	330.9572	89.7562	342.4711
novel_circ_0020772	chr9	ENSRNOG00000045605	11194238	11209094	-	exonic	3	-1.0286	0.0005	0.0365	0	0	0	30.3674	48.1448	76.3747	102.5786	62.2675
novel_circ_0020979	chr9	ENSRNOG00000012942	2205171	2246426	+	exonic	2	-0.5721	0.0002	0.0233	835.6034	419.5706	880.0603	409.9605	866.6068	712.8310	1000.1410	1027.4133
novel_circ_0021067	chr9	ENSRNOG00000012045	31604267	31693992	-	exonic	6	-0.5078	0.0001	0.0128	1270.1172	885.7602	670.5222	1032.4932	1267.8136	1502.0367	1474.5669	1385.4512
novel_circ_0021146	chr9	ENSRNOG00000015699	43004143	43004499	-	exonic	2	-1.0453	0.0003	0.0268	100.2724	0	0	75.9186	176.5310	101.8330	141.0455	186.8024
novel_circ_0021161	chr9	ENSRNOG00000058681	44382120	44390535	-	exonic	3	-0.9757	0.0006	0.0401	100.2724	0	0	75.9186	112.3379	203.6660	102.5786	171.2355
novel_circ_0021292	chr9	ENSRNOG00000032659	62291941	62311800	+	exonic	2	-1.0793	0.0003	0.0309	0	69.9284	0	30.3674	64.1931	203.6660	64.1116	108.9681
novel_circ_0021738	chrX	n/a	107663670	107679677	-	intergenic	4	-0.9731	0.0003	0.0308	0	0	0	0	64.1931	76.3747	0	108.9681
novel_circ_0022070	chrX	ENSRNOG00000007014	39830727	39885483	+	exonic	6	-0.7817	0.0008	0.0459	267.3931	139.8569	167.6305	91.1023	401.2068	101.8330	435.9589	280.2036
novel_circ_0000358	chr10	ENSRNOG00000026745	39683305	39705408	+	exonic	5	1.1350	0.0000	0.0074	284.1052	186.4758	83.8153	379.5931	128.3862	101.8330	64.1116	62.2675
novel_circ_0001449	chr11	ENSRNOG00000001682	34611538	34631134	+	intronic	3	1.2601	0.0001	0.0120	50.1362	116.5474	125.7229	106.2861	0	0	0	46.7006
novel_circ_0001525	chr11	ENSRNOG00000061472	37459698	37475255	-	exonic	2	0.5577	0.0001	0.0176	852.3155	2121.1627	1089.5985	1761.3119	802.4137	1094.7047	974.4964	918.4452
novel_circ_0001928	chr11	ENSRNOG00000037851	89108653	89110961	-	exonic	2	1.0278	0.0002	0.0222	100.2724	209.7853	209.5382	242.9396	128.3862	0	51.2893	77.8343
novel_circ_0002170	chr12	ENSRNOG00000001004	36980979	36993990	+	exonic	1	0.9342	0.0004	0.0334	50.1362	0	125.7229	60.7349	0	0	0	0
novel_circ_0002184	chr12	ENSRNOG00000001053	37474836	37478321	-	exonic	2	1.1443	0.0001	0.0132	83.5603	116.5474	0	60.7349	0	0	0	0
novel_circ_0002670	chr13	n/a	35579756	35608096	-	intergenic	1	1.2544	0.0000	0.0083	116.9845	163.1664	167.6305	167.0210	32.0965	0	76.9339	31.1337
novel_circ_0003298	chr13	ENSRNOG00000021497	95193008	95230772	-	exonic	3	1.2665	0.0000	0.0107	167.1207	93.2379	167.6305	60.7349	32.0965	0	25.6446	31.1337
novel_circ_0003883	chr14	ENSRNOG00000002643	44496990	44498906	+	exonic	2	0.9750	0.0007	0.0443	83.5603	46.6190	83.8153	60.7349	32.0965	0	0	0
novel_circ_0004054	chr14	ENSRNOG00000011073	81163988	81182711	-	exonic	5	1.1673	0.0000	0.0106	83.5603	139.8569	335.2611	288.4907	0	76.3747	64.1116	108.9681
novel_circ_0004600	chr15	ENSRNOG00000013707	41008528	41011344	+	exonic	1	0.9655	0.0009	0.0500	133.6965	93.2379	125.7229	91.1023	0	76.3747	38.4670	31.1337

novel_circ_0004741	chr15	ENSRNOG00000016029	55100385	55104181	-	exonic	3	0.9108	0.0008	0.0461	100.2724	163.1664	251.4458	136.6535	32.0965	127.2912	38.4670	62.2675
novel_circ_0005465	chr16	ENSRNOG00000017231	71834318	71862207	+	exonic	3	0.9312	0.0004	0.0337	66.8483	0	83.8153	75.9186	0	0	0	0
novel_circ_0005771	chr17	ENSRNOG00000032215	29730436	29731094	-	exonic	1	0.4283	0.0000	0.0054	2540.2343	2470.8049	3939.3177	3249.3167	2118.3721	2189.4094	1936.1705	2303.8964
novel_circ_0006043	chr17	ENSRNOG00000014670	63849304	63879166	-	exonic	3	0.5591	0.0006	0.0416	534.7862	955.6887	670.5222	576.9815	577.7378	280.0407	435.9589	389.1717
novel_circ_0006305	chr17	ENSRNOG00000008659	87738588	87739346	-	exonic	2	1.0878	0.0001	0.0171	83.5603	69.9284	0	91.1023	0	0	0	0
novel_circ_0006593	chr18	ENSRNOG00000024241	27445383	27449360	-	exonic	4	0.8439	0.0008	0.0481	150.4086	279.7138	167.6305	167.0210	80.2414	76.3747	102.5786	77.8343
novel_circ_0007005	chr18	ENSRNOG00000051965	69634005	69657373	-	exonic	1	0.7338	0.0001	0.0115	367.6655	652.6654	754.3374	576.9815	304.9172	280.0407	359.0250	295.7705
novel_circ_0007080	chr18	ENSRNOG00000052894	74317435	74332122	+	exonic	4	1.2742	0.0000	0.0070	0	93.2379	125.7229	106.2861	0	0	0	0
novel_circ_0007300	chr19	ENSRNOG00000024101	22158620	22179584	-	exonic	5	0.7380	0.0002	0.0212	501.3620	372.9517	963.8756	303.6745	256.7724	254.5825	333.3803	217.9361
novel_circ_0007352	chr19	ENSRNOG00000018382	29642343	29671928	-	exonic	3	1.6876	0.0000	0.0007	33.4241	116.5474	125.7229	121.4698	0	0	0	0
novel_circ_0007513	chr19	n/a	42856165	42920390	-	intergenic	1	1.6489	0.0000	0.0007	100.2724	279.7138	125.7229	167.0210	48.1448	0	0	46.7006
novel_circ_0007589	chr19	ENSRNOG00000019140	54803497	54822499	+	exonic	3	1.3041	0.0000	0.0071	66.8483	209.7853	125.7229	182.2047	0	50.9165	51.2893	31.1337
novel_circ_0007900	chr1	ENSRNOG00000011526	126819844	126824250	+	exonic	4	1.0072	0.0007	0.0432	100.2724	46.6190	251.4458	91.1023	48.1448	0	64.1116	0
novel_circ_0007936	chr1	ENSRNOG00000014187	128978103	128978649	+	exonic	1	0.9887	0.0002	0.0247	167.1207	93.2379	251.4458	379.5931	96.2896	0	89.7562	140.1018
novel_circ_0008336	chr1	ENSRNOG00000019613	171715868	171720495	+	exonic	3	1.1062	0.0000	0.0111	116.9845	349.6422	167.6305	227.7558	128.3862	76.3747	51.2893	46.7006
novel_circ_0008525	chr1	ENSRNOG00000025940	190980863	190991049	+	exonic	1	0.6101	0.0002	0.0241	434.5138	978.9982	880.0603	789.5536	513.5447	534.6232	307.7357	513.7066
novel_circ_0008698	chr1	n/a	204959172	204961691	+	intergenic	2	0.2843	0.0001	0.0200	4562.3945	4941.6098	5573.7155	5177.6496	4204.6476	4124.2363	4167.2544	3424.7108
novel_circ_0008891	chr1	ENSRNOG00000014183	233464786	233469856	+	intronic	2	0.9959	0.0005	0.0379	0	116.5474	83.8153	106.2861	0	0	25.6446	0
novel_circ_0009463	chr1	ENSRNOG00000014240	29436595	29471715	+	exonic	3	0.8546	0.0008	0.0460	33.4241	69.9284	125.7229	0	0	0	0	0
novel_circ_0010152	chr20	ENSRNOG00000000583	45551507	45553536	+	exonic	2	1.1194	0.0002	0.0256	66.8483	93.2379	125.7229	91.1023	0	0	51.2893	0
novel_circ_0010492	chr2	ENSRNOG00000014139	128562841	128585482	-	exonic	2	0.8618	0.0007	0.0446	50.1362	69.9284	0	75.9186	0	0	0	0
novel_circ_0010536	chr2	ENSRNOG00000010815	140357773	140387503	-	intronic	3	0.8030	0.0001	0.0122	350.9534	512.8086	419.0764	971.7583	353.0620	229.1242	282.0911	326.9042
novel_circ_0011346	chr2	n/a	23042185	23044366	-	intergenic	1	1.4847	0.0000	0.0023	0	116.5474	167.6305	106.2861	0	0	0	0
novel_circ_0011745	chr2	n/a	33950933	33955969	-	intergenic	3	0.3773	0.0001	0.0148	2690.6429	3333.2556	4232.6712	3613.7261	2407.2410	2494.9084	2077.2160	3113.3735
novel_circ_0012252	chr3	ENSRNOG00000014483	111241746	111250504	-	exonic	3	1.3218	0.0000	0.0056	83.5603	46.6190	83.8153	75.9186	0	0	0	0
novel_circ_0012298	chr3	ENSRNOG00000011059	112713523	112752649	-	exonic	6	0.7550	0.0002	0.0248	334.2414	512.8086	293.3534	409.9605	224.6758	229.1242	243.6241	140.1018
novel_circ_0012861	chr3	ENSRNOG00000019154	162506116	162514248	-	exonic	2	0.9907	0.0007	0.0442	100.2724	116.5474	83.8153	212.5721	48.1448	0	0	108.9681
novel_circ_0013042	chr3	ENSRNOG00000010526	22198807	22239368	-	exonic	3	0.6016	0.0008	0.0457	451.2258	466.1896	377.1687	652.9001	256.7724	356.4155	282.0911	342.4711

novel_circ_0013175	chr3	ENSRNOG00000018666	3785952	3786880	+	exonic	2	1.5793	0.0000	0.0008	100.2724	163.1664	209.5382	303.6745	80.2414	0	25.6446	31.1337
novel_circ_0013194	chr3	n/a	40004820	40038575	+	intergenic	3	0.6570	0.0004	0.0317	635.0586	372.9517	377.1687	546.6140	304.9172	356.4155	230.8018	295.7705
novel_circ_0013332	chr3	ENSRNOG00000024808	54547245	54554495	-	exonic	3	0.9870	0.0000	0.0004	584.9224	629.3560	1173.4138	1078.0443	288.8689	534.6232	269.2687	498.1398
novel_circ_0013449	chr3	ENSRNOG00000010812	63416256	63433816	+	exonic	4	1.2202	0.0000	0.0043	116.9845	279.7138	251.4458	288.4907	64.1931	50.9165	64.1116	108.9681
novel_circ_0013639	chr3	ENSRNOG00000009152	93705173	93733686	-	exonic	4	1.3045	0.0000	0.0036	167.1207	186.4758	251.4458	258.1233	80.2414	50.9165	0	108.9681
novel_circ_0013982	chr4	ENSRNOG00000006813	140176028	140185951	-	exonic	4	0.8926	0.0003	0.0281	150.4086	349.6422	251.4458	227.7558	144.4345	101.8330	115.4009	62.2675
novel_circ_0014194	chr4	n/a	170518671	170526305	+	intergenic	1	1.1916	0.0001	0.0178	83.5603	69.9284	125.7229	106.2861	32.0965	0	25.6446	0
novel_circ_0014205	chr4	ENSRNOG00000007570	171770055	171787877	+	exonic	2	0.9692	0.0003	0.0289	50.1362	46.6190	167.6305	0	0	0	0	0
novel_circ_0014273	chr4	ENSRNOG00000001804	180711144	180722357	-	exonic	3	0.9895	0.0002	0.0265	0	69.9284	125.7229	60.7349	0	0	0	0
novel_circ_0014316	chr4	ENSRNOG00000049378	183486984	183491414	-	exonic	1	1.1629	0.0001	0.0123	100.2724	46.6190	83.8153	30.3674	0	0	0	0
novel_circ_0014822	chr4	ENSRNOG00000005564	66172554	66200637	+	exonic	4	0.5365	0.0003	0.0304	685.1948	1048.9266	796.2451	819.9210	706.1240	534.6232	384.6696	560.4072
novel_circ_0014828	chr4	ENSRNOG00000005564	66172554	66232512	+	exonic	2	0.8323	0.0004	0.0338	284.1052	279.7138	377.1687	197.3884	128.3862	254.5825	51.2893	124.5349
novel_circ_0014896	chr4	ENSRNOG00000010957	67463556	67483807	-	exonic	2	1.3410	0.0000	0.0066	116.9845	69.9284	167.6305	151.8372	32.0965	0	25.6446	31.1337
novel_circ_0015074	chr4	n/a	99489314	99494425	+	intergenic	2	0.9814	0.0007	0.0446	116.9845	69.9284	125.7229	167.0210	64.1931	50.9165	0	46.7006
novel_circ_0015235	chr5	ENSRNOG00000006853	109530785	109563853	-	exonic	3	0.5863	0.0005	0.0371	534.7862	675.9749	460.9840	652.9001	433.3034	483.7067	307.7357	280.2036
novel_circ_0015518	chr5	ENSRNOG00000019381	136437824	136447778	+	exonic	4	0.4754	0.0004	0.0328	818.8913	1095.5456	1131.5062	880.6559	738.2206	534.6232	641.1161	684.9422
novel_circ_0015525	chr5	ENSRNOG00000019977	137096508	137105017	-	exonic	1	1.1177	0.0001	0.0150	83.5603	116.5474	83.8153	0	0	0	0	0
novel_circ_0015676	chr5	ENSRNOG00000014302	145257716	145264951	+	exonic	2	0.9748	0.0006	0.0411	167.1207	46.6190	167.6305	227.7558	80.2414	127.2912	25.6446	0
novel_circ_0016119	chr5	ENSRNOG00000009974	36040324	36054615	+	exonic	3	0.7979	0.0001	0.0172	217.2569	512.8086	586.7069	728.8187	240.7241	178.2077	230.8018	373.6048
novel_circ_0016362	chr5	ENSRNOG00000025076	75968776	75975620	-	exonic	2	1.0106	0.0005	0.0366	150.4086	139.8569	125.7229	91.1023	64.1931	0	25.6446	62.2675
novel_circ_0016430	chr5	ENSRNOG00000005391	7763384	7798747	-	exonic	3	1.1447	0.0001	0.0134	33.4241	46.6190	83.8153	91.1023	0	0	0	0
novel_circ_0016619	chr6	ENSRNOG00000029871	105078167	105080571	-	intronic	1	0.9440	0.0000	0.0063	250.6810	536.1180	293.3534	622.5326	208.6276	229.1242	192.3348	171.2355
novel_circ_0016844	chr6	ENSRNOG00000004207	122848758	122863499	-	exonic	4	0.8569	0.0009	0.0493	167.1207	116.5474	335.2611	212.5721	80.2414	0	102.5786	140.1018
novel_circ_0016950	chr6	ENSRNOG00000005287	128643587	128644617	+	intronic	3	1.0930	0.0001	0.0168	50.1362	46.6190	209.5382	0	0	0	0	0
novel_circ_0017155	chr6	ENSRNOG00000050220	15083616	15085804	-	exonic	1	1.1161	0.0002	0.0250	66.8483	93.2379	125.7229	60.7349	0	0	38.4670	0
novel_circ_0017360	chr6	ENSRNOG00000026649	28235695	28272697	+	exonic	3	0.7432	0.0000	0.0038	735.3310	815.8318	712.4298	941.3908	561.6896	585.5397	346.2027	358.0380
novel_circ_0017472	chr6	ENSRNOG00000024503	38745003	38762910	+	exonic	1	0.9414	0.0005	0.0363	150.4086	209.7853	167.6305	121.4698	48.1448	76.3747	51.2893	77.8343
novel_circ_0018264	chr7	n/a	122878044	122882820	+	intergenic	2	0.9918	0.0007	0.0448	33.4241	93.2379	83.8153	106.2861	0	0	0	46.7006

novel_circ_0018660	chr7	n/a	32922003	32982696	-	intergenic	5	0.3857	0.0000	0.0006	5982.9203	7762.0568	7669.0973	6954.1452	4958.9164	5448.0652	5359.7302	4965.8307
novel_circ_0018665	chr7	n/a	32949651	32982696	-	intergenic	4	0.6468	0.0001	0.0156	601.6344	1095.5456	670.5222	1260.2490	770.3171	381.8737	500.0705	529.2735
novel_circ_0018711	chr7	ENSRNOG00000024450	41322520	41326655	+	exonic	2	1.2735	0.0000	0.0071	0	46.6190	209.5382	91.1023	0	0	0	0
novel_circ_0018738	chr7	ENSRNOG00000004585	47425323	47425894	-	exonic	1	0.9984	0.0004	0.0319	150.4086	233.0948	167.6305	182.2047	0	0	141.0455	93.4012
novel_circ_0018754	chr7	ENSRNOG00000006426	50173692	50232687	-	exonic	2	1.0680	0.0004	0.0334	116.9845	186.4758	125.7229	0	32.0965	0	38.4670	0
novel_circ_0018773	chr7	ENSRNOG00000052157	52258463	52258952	-	exonic	1	1.1305	0.0000	0.0049	150.4086	559.4275	125.7229	592.1652	112.3379	203.6660	102.5786	140.1018
novel_circ_0019059	chr7	ENSRNOG00000004201	78347123	78375555	+	exonic	5	0.9416	0.0000	0.0071	267.3931	419.5706	502.8916	364.4094	128.3862	127.2912	217.9795	186.8024
novel_circ_0019675	chr8	ENSRNOG00000006957	1652852	1654237	-	exonic	2	1.0530	0.0002	0.0203	33.4241	93.2379	83.8153	30.3674	0	0	0	0
novel_circ_0019710	chr8	ENSRNOG00000013752	23035954	23048640	-	exonic	5	0.4512	0.0000	0.0071	2406.5378	3892.6832	3268.7956	3644.0935	2599.8203	1731.1609	2231.0839	2584.1000
novel_circ_0019719	chr8	ENSRNOG00000015189	23508991	23534941	+	exonic	3	0.9071	0.0002	0.0260	233.9689	209.7853	251.4458	242.9396	80.2414	76.3747	76.9339	171.2355
novel_circ_0020253	chr8	ENSRNOG00000011739	70339050	70347005	+	exonic	3	0.7332	0.0005	0.0363	417.8017	256.4043	293.3534	227.7558	593.7861	432.7902	500.0705	389.1717
novel_circ_0020645	chr8	n/a	95867336	95894135	-	intergenic	1	1.3432	0.0000	0.0074	33.4241	139.8569	125.7229	106.2861	0	50.9165	0	0
novel_circ_0020886	chr9	ENSRNOG00000037227	121831707	121862897	+	exonic	3	1.2050	0.0000	0.0038	250.6810	233.0948	125.7229	728.8187	112.3379	76.3747	192.3348	77.8343
novel_circ_0020973	chr9	ENSRNOG00000012708	21275899	21276380	-	exonic	1	1.3296	0.0000	0.0073	200.5448	186.4758	0	151.8372	64.1931	0	38.4670	0
novel_circ_0021659	chr9	ENSRNOG00000019476	96746439	96775060	+	exonic	3	0.9265	0.0007	0.0433	133.6965	139.8569	209.5382	212.5721	144.4345	76.3747	51.2893	0
novel_circ_0021711	chrX	ENSRNOG00000003929	104429942	104487794	-	exonic	2	1.0469	0.0004	0.0311	50.1362	163.1664	0	91.1023	0	0	0	31.1337
novel_circ_0022246	chrX	ENSRNOG00000037799	82932967	82946086	-	exonic	1	0.9633	0.0003	0.0294	100.2724	93.2379	0	30.3674	0	0	0	0

Supplementary Table S3. Feature details of differentially expressed circRNAs.

Day-7 circRNA	Feature Detail
novel_circ_0000126	exon:13931236-13931307,exon:13931422-13931493
novel_circ_0001138	exon:91965974-91966129,exon:91968439-91968514,exon:91969431-91969538
novel_circ_0001277	exon:97555577-97555658,exon:97559328-97559404,exon:97561392-97561450,exon:97561690-97561741,exon:97568615-97568711
novel_circ_0001942	exon:9799899-9800225
novel_circ_0001966	exon:12448358-12448445,exon:12449032-12449150,exon:12450245-12450318,exon:12458467-12458611,exon:12463281-12463405
novel_circ_0001986	exon:13524227-13524357
novel_circ_0001991	exon:13879069-13879344,exon:13884069-13884323

novel_circ_0002488	exon:107733302-107733441,exon:107745993-107746179,exon:107749368-107749528
novel_circ_0002537	exon:111138406-111138605,exon:111144026-111144278,exon:111159777-111159973
novel_circ_0002652	exon:31140421-31140715,exon:31152740-31152907,exon:31162915-31163102
novel_circ_0003107	exon:82185980-82186169,exon:82187107-82187180,exon:82187333-82187408,exon:82190111-82190210
novel_circ_0003119	intergenic_region:83256698-83257043
novel_circ_0003267	exon:95098506-95098593,exon:95120752-95120880,exon:95128660-95128782
novel_circ_0003687	exon:28448337-28448428,exon:28465763-28465972
novel_circ_0003821	intergenic_region:42007313-42007427,intergenic_region:42008911-42009010,intergenic_region:42015346-42015391
novel_circ_0004461	exon:25905491-25905638,exon:25907368-25907577,exon:25909586-25909630,exon:25910259-25910368
novel_circ_0004464	exon:25917999-25918193
novel_circ_0004985	exon:14184372-14184568
novel_circ_0005300	exon:5417618-5417768,exon:5417860-5417920,exon:5422280-5422411
novel_circ_0005365	exon:63915078-63915255,exon:63924021-63924142
novel_circ_0005542	exon:79918764-79918983,exon:79924033-79924180
novel_circ_0005582	exon:8416756-8416831,exon:8418463-8418598,exon:8421070-8421217
novel_circ_0006257	intron:84296197-84296277,intron:84298114-84298191,intron:84298278-84298603,intron:84299732-84299839
novel_circ_0006453	exon:17182718-17182812,exon:17190112-17190255,exon:17196570-17196811
novel_circ_0006458	exon:17218299-17218655,exon:17224244-17224348
novel_circ_0006732	intergenic_region:37284943-37284973,intergenic_region:37292527-37292606
novel_circ_0006981	exon:66581616-66581732,exon:66632010-66632117
novel_circ_0007008	exon:69647502-69647714,exon:69654505-69654534,exon:69655142-69655316,exon:69656997-69657373
novel_circ_0007443	exon:34292209-34292283,exon:34298523-34298610,exon:34305791-34305849,exon:34310750-34310855,exon:34312371-34312530
novel_circ_0007466	exon:38062031-38062193
novel_circ_0007524	exon:43119868-43120001,exon:43120530-43120685,exon:43123897-43124021
novel_circ_0007589	exon:54803498-54803673,exon:54820626-54820756,exon:54822461-54822499
novel_circ_0007692	intron:102636819-102637016
novel_circ_0007968	exon:134799596-134799771,exon:134812982-134813134,exon:134813534-134813696
novel_circ_0008366	exon:175005900-175006045,exon:175011541-175011736

novel_circ_0009219	exon:263867359-263867553,exon:263873869-263873958,exon:263874851-263874939,exon:263876545-263876614
novel_circ_0009369	exon:278580846-278580929,exon:278582003-278582116,exon:278590619-278590747,exon:278594352-278594581
novel_circ_0009584	exon:47193254-47193353,exon:47202143-47202318
novel_circ_0009928	exon:20268027-20268125,exon:20276012-20276110
novel_circ_0010635	exon:148003674-148003764,exon:148009483-148009588
novel_circ_0010697	exon:157552305-157552386,exon:157562992-157563195,exon:157581094-157581187
novel_circ_0011299	intergenic_region:224356779-224356865,intergenic_region:224357412-224357546,intergenic_region:224359896-224360097,intergenic_region:224363077-224363213
novel_circ_0011797	exon:3809213-3809383,exon:3821216-3821346,exon:3834636-3834822,exon:3839583-3839676
novel_circ_0011799	exon:3809213-3809383,exon:3821216-3821346,exon:3858236-3858337,exon:3858804-3858878,exon:3860738-3860857
novel_circ_0011802	exon:38096882-38097085,exon:38097428-38097500
novel_circ_0012622	exon:136874645-136874704,exon:136878106-136878171,exon:136892807-136892916,exon:136893540-136893637
novel_circ_0012942	exon:177140837-177140955,exon:177141608-177141826,exon:177144903-177145025
novel_circ_0013093	intergenic_region:25350827-25350933,intergenic_region:25414210-25414329,intergenic_region:25414474-25414590
novel_circ_0013367	exon:56199666-56199762,exon:56213450-56213623
novel_circ_0013644	exon:94142096-94142259,exon:94152523-94152694,exon:94155970-94156121,exon:94161554-94161613
novel_circ_0013812	exon:118500900-118500987,exon:118503790-118503935,exon:118507627-118507702,exon:118508942-118509011
novel_circ_0013930	exon:132075419-132075574,exon:132079305-132079371,exon:132082496-132082556,exon:132083937-132084031
novel_circ_0013932	intergenic_region:132075419-132075574,intergenic_region:132079305-132079371,intergenic_region:132082496-132082556,intergenic_region:132083937-132084031,intergenic_region:132094678-132094750,intergenic_region:132113754-132113888
novel_circ_0014036	exon:147692061-147692204,exon:147692755-147692909
novel_circ_0014203	exon:171527470-171527537
novel_circ_0014232	exon:177269463-177269627,exon:177272028-177272214,exon:177277503-177277598,exon:177280993-177281107
novel_circ_0014527	exon:38838812-38839024,exon:38873514-38873762
novel_circ_0014647	exon:58143324-58143481,exon:58146336-58146428,exon:58147373-58147448
novel_circ_0014650	intergenic_region:58367537-58367703,intergenic_region:58372935-58373171
novel_circ_0015600	exon:14282912-14283025,exon:14287829-14287930
novel_circ_0015761	exon:149965148-149965456
novel_circ_0016126	intergenic_region:37742708-37743005,intergenic_region:37746751-37746901,intergenic_region:37756985-37757012
novel_circ_0016428	exon:7763385-7763592,exon:7764098-7764306

novel_circ_0016926	intergenic_region:126864143-126864263,intergenic_region:126870603-126870907,intergenic_region:126874110-126874280,intergenic_region:126883895-126883987,intergenic_region:126888691-126888782,intergenic_region:126893337-126893392,intergenic_region:126894065-126894194
novel_circ_0017341	exon:28022406-28022491,exon:28041574-28041728,exon:28048712-28048817,exon:28051538-28051704
novel_circ_0017915	exon:88462488-88462618,exon:88478332-88478487
novel_circ_0017982	exon:9405093-9405200,exon:9405801-9405891,exon:9421744-9421901,exon:9425144-9425251,exon:9429437-9429563,exon:9433838-9433973,exon:9434835-9434931
novel_circ_0018128	exon:112745541-112745667,exon:112751626-112751799,exon:112755242-112755308,exon:112760984-112761127
novel_circ_0018332	intron:127178724-127178852,intron:127180623-127180685,intron:127182349-127182545
novel_circ_0018705	exon:41167312-41167751
novel_circ_0018774	exon:52310713-52310832,exon:52317280-52317663,exon:52318289-52318397
novel_circ_0019339	exon:109243253-109243370,exon:109250465-109250568,exon:109254694-109254764,exon:109263072-109263159,exon:109288080-109288160
novel_circ_0019522	exon:120442503-120442588,exon:120446281-120446457
novel_circ_0019538	exon:122131052-122131159
novel_circ_0019835	exon:39790059-39790162,exon:39807839-39807952
novel_circ_0019857	exon:456819-456940
novel_circ_0020014	exon:59022287-59022408,exon:59025635-59025874
novel_circ_0020807	exon:112406660-112406825,exon:112408205-112408426,exon:112409488-112409606,exon:112411999-112412113
novel_circ_0021517	intron:7651929-7652086,intron:7658816-7658922
novel_circ_0000145	exon:15544776-15544874,exon:15554042-15554152,exon:15555290-15555433
novel_circ_0000953	exon:74343130-74343263
novel_circ_0001364	exon:24547990-24548183,exon:24550640-24550752
novel_circ_0001517	exon:37020529-37020831
novel_circ_0001623	exon:62059611-62059798,exon:62060164-62060327,exon:62062326-62062460,exon:62067556-62067655
novel_circ_0002032	exon:1793318-1793869
novel_circ_0002592	exon:22151000-22151171
novel_circ_0002608	intergenic_region:25113373-25113565,intergenic_region:25113789-25113924,intergenic_region:25114626-25114734,intergenic_region:25115322-25115431
novel_circ_0002856	exon:56114438-56114507,exon:56115261-56115341,exon:56121585-56121638,exon:56161619-56161720,exon:56173047-56173172,exon:56183024-56183125
novel_circ_0003047	exon:76979427-76979486,exon:76980791-76980867,exon:76989280-76989337,exon:77000105-77000219,exon:77004110-77004245
novel_circ_0003280	exon:95120752-95120880,exon:95196258-95196402,exon:95208218-95208329
novel_circ_0003721	exon:2980819-2980977,exon:2988677-2988823

novel_circ_0004037	exon:79407476-79407594,exon:79419997-79420077,exon:79421742-79421836
novel_circ_0004188	exon:87328008-87328157,exon:87331763-87331877,exon:87350088-87350206,exon:87357621-87357732
novel_circ_0004267	intron:104974440-104974615,intron:104976308-104976461
novel_circ_0004272	intergenic_region:106432818-106432918,intergenic_region:106434176-106434369
novel_circ_0004615	exon:41493485-41493569,exon:41495139-41495224,exon:41498106-41498217,exon:41507557-41507648
novel_circ_0007281	exon:20292378-20292462
novel_circ_0007599	exon:55882755-55882857,exon:55885922-55886071,exon:55886687-55886776
novel_circ_0007740	exon:112926910-112927130,exon:112927235-112927302,exon:112928909-112929030
novel_circ_0007936	exon:128978104-128978649
novel_circ_0008023	exon:141053114-141053289,exon:141055040-141055207
novel_circ_0008045	exon:143143055-143143190,exon:143143363-143143524
novel_circ_0008194	exon:162643583-162643789
novel_circ_0008578	intergenic_region:195009269-195009379,intergenic_region:195010089-195010200,intergenic_region:195010746-195010944,intergenic_region:195011082-195011154,intergenic_region:195011716-195011851
novel_circ_0008667	exon:200648164-200648287,exon:200671013-200671090,exon:200672145-200672411
novel_circ_0008668	exon:200671013-200671090,exon:200672145-200672411
novel_circ_0008735	exon:209778386-209778494,exon:209785479-209785567,exon:209797087-209797179
novel_circ_0009053	intergenic_region:250612597-250612674,intergenic_region:250613890-250614017,intergenic_region:250630614-250630729
novel_circ_0009321	intron:270300442-270300509,intron:270363787-270363837,intron:270363967-270364139
novel_circ_0009467	intergenic_region:31118290-31118448
novel_circ_0009709	exon:73746711-73746931,exon:73747680-73747791
novel_circ_0009895	exon:14335985-14336075,exon:14340849-14340940,exon:14342249-14342412
novel_circ_0010038	intergenic_region:3149071-3149187
novel_circ_0010044	intergenic_region:31735983-31736111
novel_circ_0010196	exon:50659751-50659805,exon:50660031-50660120,exon:50661539-50661643,exon:50676394-50676469,exon:50687737-50687868
novel_circ_0010281	exon:7363740-7363937,exon:7371915-7372069
novel_circ_0010541	exon:140524188-140524296,exon:140526783-140526881,exon:140528275-140528418,exon:140529698-140529795
novel_circ_0010620	intergenic_region:145485716-145485849,intergenic_region:145486403-145486600,intergenic_region:145509128-145509228,intergenic_region:145512859-145513090
novel_circ_0010648	exon:149314177-149314281,exon:149315029-149315205

novel_circ_0010732	intergenic_region:170837750-170838157,intergenic_region:170842070-170842114
novel_circ_0010988	exon:189723524-189723655
novel_circ_0010989	exon:189723524-189723655,exon:189728803-189729005
novel_circ_0011096	exon:205814398-205814516,exon:205815014-205815158,exon:205815716-205815848
novel_circ_0011118	exon:206563072-206563151,exon:206567018-206567195,exon:206570624-206570833
novel_circ_0011143	exon:207356978-207357143,exon:207358742-207358796,exon:207360163-207360278
novel_circ_0011215	intergenic_region:213064653-213065395
novel_circ_0012671	exon:141107008-141107311
novel_circ_0012760	intron:15225636-15225751,intron:15226686-15226781,intron:15228025-15228093,intron:15229027-15229198
novel_circ_0012856	exon:161474539-161474665,exon:161487665-161487731
novel_circ_0013035	exon:21878521-21878612,exon:21879730-21879836,exon:21886616-21886722,exon:21888304-21888448,exon:21894168-21894333,exon:21898276-21898496,exon:21904100-21904269
novel_circ_0013064	exon:23327420-23327515,exon:23329133-23329228
novel_circ_0013083	intergenic_region:25268501-25268665,intergenic_region:25314148-25314285,intergenic_region:25316483-25316608
novel_circ_0013129	intron:29584278-29584549,intron:29597192-29597359
novel_circ_0013218	exon:45033238-45033378,exon:45086410-45086616,exon:45088857-45088995
novel_circ_0013445	exon:63010097-63010229,exon:63038652-63038716,exon:63053909-63054049,exon:63058178-63058267
novel_circ_0013708	intron:10775672-10775777,intron:10776758-10776948,intron:10777039-10777395
novel_circ_0014210	exon:174700910-174700993,exon:174702092-174702212,exon:174702703-174702807,exon:174704692-174704764
novel_circ_0014784	exon:64899412-64899587
novel_circ_0015528	exon:138375787-138375928,exon:138381415-138381527,exon:138395607-138395761
novel_circ_0015544	exon:138622350-138622674
novel_circ_0015668	exon:144931944-144932199
novel_circ_0015981	exon:169281336-169281473,exon:169281591-169281710,exon:169285293-169285425
novel_circ_0017063	exon:136059672-136059863,exon:136071831-136071884,exon:136081574-136081622
novel_circ_0017497	exon:4298605-4298704,exon:4302978-4303108,exon:4307775-4307843,exon:4312304-4312324,exon:4314959-4315062,exon:4315576-4315682
novel_circ_0017823	exon:76491315-76491673
novel_circ_0018274	intergenic_region:123600187-123600374,intergenic_region:123600596-123600737
novel_circ_0018334	exon:127189206-127189320,exon:127192981-127193134,exon:127221964-127222046,exon:127223122-127223231

novel_circ_0018666	intergenic_region:32955133-32955235,intergenic_region:32976786-32976910,intergenic_region:32979163-32979288,intergenic_region:32981667-32981808,intergenic_region:32982568-32982696
novel_circ_0018801	exon:55151632-55151796
novel_circ_0018890	exon:64707678-64707812,exon:64709209-64709354,exon:64716367-64716450,exon:64720268-64720343,exon:64722402-64722547
novel_circ_0019066	intron:78413918-78414074,intron:78415235-78415345
novel_circ_0019343	exon:109302672-109302764,exon:109304309-109304483,exon:109306043-109306182
novel_circ_0019725	exon:23851919-23852021,exon:23854410-23854514,exon:23864610-23864750,exon:23878265-23878437,exon:23884696-23884848
novel_circ_0020226	exon:69139840-69139904,exon:69141576-69141777,exon:69143608-69143732
novel_circ_0021014	exon:28529457-28529623,exon:28534875-28534987,exon:28552021-28552133
novel_circ_0021355	exon:66796280-66796534,exon:66804728-66804771
novel_circ_0021480	exon:73433883-73434017
novel_circ_0021642	exon:94463647-94463776,exon:94464704-94464799,exon:94474695-94474809,exon:94479170-94479277,exon:94479354-94479444,exon:94480129-94480240,exon:94481127-94481167
novel_circ_0021718	exon:10567595-10567806,exon:10606710-10606963
novel_circ_0021721	intergenic_region:105810348-105810447
novel_circ_0021722	intergenic_region:105819979-105820080,intergenic_region:105821635-105821741
novel_circ_0021865	intergenic_region:13905156-13905232,intergenic_region:13913065-13913131,intergenic_region:13915027-13915112,intergenic_region:13917431-13917538

**Day-28 circRNA**	**Feature Detail**
novel_circ_0000323	exon:37167400-37167606,exon:37168239-37168311,exon:37168668-37168723,exon:37172654-37172772
novel_circ_0001023	exon:81798931-81799149,exon:81799392-81799587
novel_circ_0001041	exon:85109546-85109770,exon:85112641-85112742
novel_circ_0001173	exon:93451477-93451644,exon:93464374-93464469,exon:93465608-93465700
novel_circ_0001319	exon:15457479-15457556,exon:15461115-15461262,exon:15474664-15474826
novel_circ_0001533	intergenic_region:42050184-42050847
novel_circ_0001582	exon:51068884-51068978,exon:51088404-51088607
novel_circ_0002291	exon:43501060-43501143,exon:43503310-43503463,exon:43510748-43510832
novel_circ_0002890	exon:57409196-57409409,exon:57411382-57411487
novel_circ_0003140	exon:83634263-83634520,exon:83637961-83638054
novel_circ_0003492	intergenic_region:113552476-113552574,intergenic_region:113560035-113560101,intergenic_region:113565354-113565546
novel_circ_0003563	exon:14455620-14455786

novel_circ_0003866	exon:43558568-43558751,exon:43559086-43559238,exon:43580429-43580559,exon:43581016-43581074
novel_circ_0004178	intron:86431783-86431896,intron:86434307-86434498,intron:86438128-86438230
novel_circ_0004335	intergenic_region:110036614-110036742,intergenic_region:110053276-110053440
novel_circ_0004820	exon:61674930-61675047,exon:61678464-61678600,exon:61682957-61683055
novel_circ_0005172	intergenic_region:39367335-39367424,intergenic_region:39371721-39371786,intergenic_region:39372328-39372404,intergenic_region:39375673-39375826
novel_circ_0005323	exon:56313381-56313498,exon:56326666-56326806,exon:56350367-56350456
novel_circ_0005594	exon:84806784-84806931,exon:84835779-84835889,exon:84842171-84842321
novel_circ_0005601	intergenic_region:85581044-85581247
novel_circ_0005867	intergenic_region:51972801-51972948,intergenic_region:51973809-51973941,intergenic_region:51980918-51981081,intergenic_region:52027511-52027612
novel_circ_0006133	exon:718534-718636,exon:719170-719277,exon:728286-728329,exon:739380-739440
novel_circ_0006352	exon:9613030-9613261
novel_circ_0006461	exon:17224244-17224348,exon:17254724-17254858,exon:17258815-17258865,exon:17264504-17264715
novel_circ_0006677	exon:32069617-32069717,exon:32071367-32071496,exon:32088892-32088963,exon:32092380-32092441,exon:32094208-32094303
novel_circ_0007444	exon:34292209-34292283,exon:34298523-34298610,exon:34305791-34305849,exon:34312371-34312530,exon:34346074-34346224
novel_circ_0007447	exon:34298523-34298610,exon:34305791-34305849,exon:34310750-34310855,exon:34312371-34312530
novel_circ_0007851	intergenic_region:122605870-122605974,intergenic_region:122613645-122613766,intergenic_region:122678291-122678378
novel_circ_0008137	exon:157422218-157422362,exon:157425551-157425677,exon:157438483-157438608,exon:157440038-157440238
novel_circ_0008495	exon:188436736-188436837,exon:188437069-188437185,exon:188437822-188437895
novel_circ_0008933	exon:235759864-235759926,exon:235761648-235761819,exon:235763930-235764036,exon:235764219-235764346
novel_circ_0009073	exon:25216049-25216223,exon:25274525-25274635,exon:25284174-25284394
novel_circ_0009393	exon:278679022-278679115,exon:278687271-278687323,exon:278698312-278698406,exon:278700185-278700263,exon:278701709-278701866,exon:278731694-278731755,exon:278749751-278749829
novel_circ_0009401	exon:278698312-278698406,exon:278700185-278700263,exon:278701687-278701866,exon:278731694-278731755,exon:278749751-278749829
novel_circ_0009446	exon:28362167-28362400,exon:28375697-28375861
novel_circ_0009684	intergenic_region:66934989-66935127,intergenic_region:66943974-66944229
novel_circ_0009822	exon:91873487-91873641,exon:91874508-91874567,exon:91876496-91876617,exon:91877688-91877766,exon:91878894-91879026
novel_circ_0009887	exon:13911860-13912191
novel_circ_0009993	exon:27535432-27535614,exon:27538479-27538541,exon:27541657-27541787,exon:27544747-27544812
novel_circ_0010178	exon:47860620-47860771,exon:47893649-47893834,exon:47896518-47896656

novel_circ_0010406	exon:11729894-11730086,exon:11747071-11747274
novel_circ_0010451	exon:123576589-123576775,exon:123582304-123582385,exon:123597469-123597547,exon:123598623-123598714,exon:123600115-123600232
novel_circ_0010818	exon:179624046-179624207,exon:179628733-179628786,exon:179638565-179638761
novel_circ_0010941	exon:188301023-188301124
novel_circ_0011109	exon:206472095-206472251,exon:206477353-206477526,exon:206477634-206477723
novel_circ_0011114	exon:206534104-206534135,exon:206535755-206536360
novel_circ_0011120	exon:206567018-206567195,exon:206570624-206570833
novel_circ_0011299	intergenic_region:224356779-224356865,intergenic_region:224357412-224357546,intergenic_region:224359896-224360097,intergenic_region:224363077-224363213
novel_circ_0011322	exon:227687641-227687819,exon:227700162-227700240,exon:227702107-227702229
novel_circ_0011441	exon:242126334-242126473,exon:242133712-242133789,exon:242150971-242151045
novel_circ_0011811	intergenic_region:39042496-39042852
novel_circ_0012262	exon:111513948-111514059,exon:111522866-111523001,exon:111523758-111523935
novel_circ_0012325	exon:113149412-113149462,exon:113149809-113149914
novel_circ_0012356	exon:11394659-11394730,exon:11396164-11396393
novel_circ_0012425	exon:121533317-121533453
novel_circ_0012482	exon:123829471-123829647,exon:123833099-123833222
novel_circ_0013601	exon:8587701-8587909,exon:8589300-8589420
novel_circ_0014083	exon:152526403-152526560,exon:152528957-152529177
novel_circ_0014625	exon:5644287-5644382,exon:5644997-5645104,exon:5649254-5649302,exon:5652602-5652694
novel_circ_0015325	exon:12156802-12156904,exon:12171585-12172003
novel_circ_0015394	intergenic_region:127620259-127620451,intergenic_region:127632645-127632705,intergenic_region:127635076-127635191
novel_circ_0015443	exon:129003129-129003245,exon:129007261-129007397,exon:129009531-129009597
novel_circ_0015592	intergenic_region:142152201-142152430
novel_circ_0015668	exon:144931944-144932199
novel_circ_0015812	exon:154919349-154919491,exon:154926879-154926973,exon:154928923-154929004,exon:154930842-154930948,exon:154931664-154931756
novel_circ_0016248	exon:60390691-60390734,exon:60392077-60392202,exon:60393026-60393196
novel_circ_0016957	exon:129632546-129632684
novel_circ_0016966	exon:129864989-129865153,exon:129871169-129871224,exon:129878417-129878486,exon:129889933-129890111

novel_circ_0017108	exon:144343082-144343185,exon:144346023-144346123,exon:144347870-144347969
novel_circ_0017111	exon:14450092-14450211,exon:14451134-14451160,exon:14457691-14457864,exon:14490230-14490352
novel_circ_0017236	exon:22373412-22373524,exon:22376624-22376735,exon:22408964-22409230
novel_circ_0017318	intergenic_region:27858538-27858646,intergenic_region:27862332-27862425,intergenic_region:27881498-27881618
novel_circ_0017606	exon:54570723-54570830,exon:54572217-54572327,exon:54583308-54583376,exon:54585153-54585265
novel_circ_0017710	exon:64847537-64847689,exon:64852693-64852824,exon:64854315-64854393,exon:64861596-64861750
novel_circ_0017764	exon:73658709-73659072
novel_circ_0018159	exon:114490398-114490487,exon:114492856-114492956,exon:114494327-114494442,exon:114496180-114496280,exon:114499074-114499158
novel_circ_0018270	exon:123336676-123336736,exon:123338325-123338422,exon:123349222-123349326
novel_circ_0018315	exon:126256293-126256375,exon:126265888-126265984,exon:126283815-126283984
novel_circ_0019279	exon:104157475-104157543,exon:104163899-104164050,exon:104167864-104168030
novel_circ_0019418	exon:115811800-115811954,exon:115812519-115812560
novel_circ_0019788	exon:33279134-33279218,exon:33296459-33296545,exon:33312321-33312458,exon:33314876-33314968,exon:33316117-33316239,exon:33317632-33317709,exon:33335342-33335423
novel_circ_0020499	exon:84434784-84434954,exon:84443616-84443660,exon:84453284-84453328,exon:84461132-84461191,exon:84461481-84461546
novel_circ_0020500	exon:84434784-84434912,exon:84443616-84443660,exon:84453284-84453328,exon:84461132-84461191
novel_circ_0020503	exon:84443616-84443660,exon:84453284-84453328,exon:84461132-84461191,exon:84461316-84461399,exon:84461481-84461546,exon:84482629-84482700
novel_circ_0020504	exon:84443616-84443660,exon:84453284-84453328,exon:84461132-84461191,exon:84461481-84461546,exon:84482629-84482700
novel_circ_0020713	intergenic_region:110314146-110314305,intergenic_region:110314406-110314527
novel_circ_0020749	exon:111607928-111608101,exon:111646207-111646329,exon:111653302-111653491
novel_circ_0020772	exon:11194239-11194343,exon:11202671-11202851,exon:11209034-11209094
novel_circ_0020979	exon:2205172-2205416,exon:2246271-2246426
novel_circ_0021067	exon:31604268-31604311,exon:31620318-31620420,exon:31631500-31631575,exon:31632461-31632610,exon:31660796-31660864,exon:31693798-31693992
novel_circ_0021146	exon:43004144-43004258,exon:43004338-43004499
novel_circ_0021161	exon:44382121-44382306,exon:44387014-44387182,exon:44390480-44390535
novel_circ_0021292	exon:62309706-62309781,exon:62311691-62311800
novel_circ_0021738	intergenic_region:107663671-107663739,intergenic_region:107675975-107676028,intergenic_region:107677388-107677467,intergenic_region:107679528-107679677
novel_circ_0022070	exon:39830728-39830787,exon:39835574-39835642,exon:39850822-39850968,exon:39860825-39860958,exon:39883718-39883807,exon:39885269-39885483
novel_circ_0000358	exon:39683306-39683357,exon:39688649-39688722,exon:39692192-39692326,exon:39700262-39700378,exon:39705265-39705408

novel_circ_0001449	intron:34611539-34611724,intron:34621441-34621503,intron:34631080-34631134
novel_circ_0001525	exon:37459699-37459845,exon:37474938-37475255
novel_circ_0001928	exon:89108654-89108904,exon:89110772-89110961
novel_circ_0002170	exon:36993848-36993990
novel_circ_0002184	exon:37474837-37474944,exon:37478129-37478321
novel_circ_0002670	intergenic_region:35607933-35608096
novel_circ_0003298	exon:95193009-95193140,exon:95208218-95208329,exon:95230647-95230772
novel_circ_0003883	exon:44498588-44498679,exon:44498796-44498906
novel_circ_0004054	exon:81163989-81164150,exon:81164278-81164356,exon:81170080-81170190,exon:81180966-81181093,exon:81182585-81182711
novel_circ_0004600	exon:41011200-41011344
novel_circ_0004741	exon:55100386-55100531,exon:55101680-55101825,exon:55104066-55104181
novel_circ_0005465	exon:71834319-71834488,exon:71835231-71835312,exon:71862024-71862207
novel_circ_0005771	exon:29730437-29731094
novel_circ_0006043	exon:63849305-63849466,exon:63860368-63860527,exon:63879032-63879166
novel_circ_0006305	exon:87738589-87738731,exon:87739210-87739346
novel_circ_0006593	exon:27445384-27445516,exon:27445615-27445720,exon:27446208-27446250,exon:27449223-27449360
novel_circ_0007005	exon:69634006-69634174
novel_circ_0007080	exon:74317436-74317572,exon:74318040-74318147,exon:74329214-74329368,exon:74331982-74332122
novel_circ_0007300	exon:22158621-22158701,exon:22162545-22162652,exon:22172781-22172880,exon:22177001-22177139,exon:22179495-22179584
novel_circ_0007352	exon:29642344-29642498,exon:29645232-29645344,exon:29671756-29671928
novel_circ_0007513	intergenic_region:42856166-42856397
novel_circ_0007589	exon:54803498-54803673,exon:54820626-54820756,exon:54822461-54822499
novel_circ_0007900	exon:126819845-126819949,exon:126820488-126820631,exon:126821843-126821919,exon:126824162-126824250
novel_circ_0007936	exon:128978104-128978649
novel_circ_0008336	exon:171715869-171715989,exon:171718017-171718188,exon:171720366-171720495
novel_circ_0008525	exon:190980864-190981054
novel_circ_0008698	intergenic_region:204959173-204959337,intergenic_region:204960841-204961691
novel_circ_0008891	intron:233464787-233464855,intron:233469672-233469856

novel_circ_0009463	exon:29436596-29436655,exon:29462625-29462756,exon:29471595-29471715
novel_circ_0010152	exon:45551508-45551618,exon:45553396-45553536
novel_circ_0010492	exon:128562842-128563038,exon:128585384-128585482
novel_circ_0010536	intron:140357774-140357876,intron:140384029-140384250,intron:140387418-140387503
novel_circ_0011346	intergenic_region:23042186-23042496
novel_circ_0011745	intergenic_region:33950934-33951051,intergenic_region:33952609-33952806,intergenic_region:33955922-33955969
novel_circ_0012252	exon:111241747-111241864,exon:111242713-111242797,exon:111250424-111250504
novel_circ_0012298	exon:112713524-112713649,exon:112714614-112714706,exon:112716589-112716654,exon:112722217-112722321,exon:112728900-112728973,exon:112752502-112752649
novel_circ_0012861	exon:162508835-162508987,exon:162514102-162514248
novel_circ_0013042	exon:22198808-22198933,exon:22217774-22217875,exon:22239323-22239368
novel_circ_0013175	exon:3785953-3786023,exon:3786701-3786880
novel_circ_0013194	intergenic_region:40004821-40004891,intergenic_region:40024657-40024755,intergenic_region:40038336-40038575
novel_circ_0013332	exon:54547246-54547301,exon:54548417-54548578,exon:54554387-54554495
novel_circ_0013449	exon:63416257-63416331,exon:63421092-63421257,exon:63431713-63431820,exon:63433679-63433816
novel_circ_0013639	exon:93705174-93705237,exon:93705513-93705613,exon:93706191-93706385,exon:93733477-93733686
novel_circ_0013982	exon:140176029-140176142,exon:140183019-140183133,exon:140183917-140184039,exon:140185869-140185951
novel_circ_0014194	intergenic_region:170526168-170526305
novel_circ_0014205	exon:171770056-171770153,exon:171787749-171787877
novel_circ_0014273	exon:180711145-180711240,exon:180713294-180713440,exon:180721313-180721399
novel_circ_0014316	exon:183491269-183491414
novel_circ_0014822	exon:66172555-66172647,exon:66180863-66180964,exon:66193731-66193868,exon:66200567-66200637
novel_circ_0014828	exon:66172555-66172647,exon:66232317-66232512
novel_circ_0014896	exon:67463557-67463820,exon:67483706-67483807
novel_circ_0015074	intergenic_region:99489315-99489436,intergenic_region:99494234-99494425
novel_circ_0015235	exon:109530786-109530889,exon:109560566-109560809,exon:109563738-109563853
novel_circ_0015518	exon:136437825-136437941,exon:136442933-136443128,exon:136444187-136444246,exon:136447687-136447778
novel_circ_0015525	exon:137104883-137105017
novel_circ_0015676	exon:145257717-145257799,exon:145264879-145264951

novel_circ_0016119	exon:36040325-36040451,exon:36043415-36043564,exon:36054456-36054615
novel_circ_0016362	exon:75968777-75968911,exon:75975497-75975620
novel_circ_0016430	exon:7763385-7763592,exon:7793484-7793620,exon:7798662-7798747
novel_circ_0016619	intron:105080483-105080571
novel_circ_0016844	exon:122848759-122848877,exon:122849273-122849379,exon:122861892-122862093,exon:122863364-122863499
novel_circ_0016950	intron:128643588-128643744,intron:128643869-128643936,intron:128644430-128644617
novel_circ_0017155	exon:15083617-15083646
novel_circ_0017360	exon:28235696-28235928,exon:28272106-28272230,exon:28272551-28272697
novel_circ_0017472	exon:38745004-38745143
novel_circ_0018264	intergenic_region:122878045-122878195,intergenic_region:122882659-122882820
novel_circ_0018660	intergenic_region:32922004-32922239,intergenic_region:32922343-32922502,intergenic_region:32980944-32981166,intergenic_region:32981667-32981808,intergenic_region:32982568-32982696
novel_circ_0018665	intergenic_region:32949652-32949856,intergenic_region:32955133-32955235,intergenic_region:32981667-32981808,intergenic_region:32982568-32982696
novel_circ_0018711	exon:41322521-41322692,exon:41326476-41326655
novel_circ_0018738	exon:47425324-47425894
novel_circ_0018754	exon:50173693-50173810,exon:50232503-50232687
novel_circ_0018773	exon:52258464-52258952
novel_circ_0019059	exon:78347124-78347191,exon:78349080-78349199,exon:78351047-78351146,exon:78373036-78373207,exon:78375402-78375555
novel_circ_0019675	exon:1652853-1652957,exon:1654070-1654237
novel_circ_0019710	exon:23035955-23035993,exon:23037219-23037444,exon:23037543-23037696,exon:23048110-23048213,exon:23048421-23048640
novel_circ_0019719	exon:23508992-23509114,exon:23524797-23524947,exon:23534877-23534941
novel_circ_0020253	exon:70339051-70339117,exon:70343121-70343296,exon:70346905-70347005
novel_circ_0020645	intergenic_region:95867337-95867470
novel_circ_0020886	exon:121831708-121831980,exon:121856478-121856631,exon:121862766-121862897
novel_circ_0020973	exon:21275900-21276380
novel_circ_0021659	exon:96746440-96746498,exon:96771733-96771874,exon:96774926-96775060
novel_circ_0021711	exon:104429943-104430115,exon:104437571-104437629
novel_circ_0022246	exon:82932968-82933021

Supplementary Table S4. Details of differentially expressed mRNAs in day-7 and day-28 ICH samples when compared with Sham. Chr: Chromosome ID where mRNA is located. **Gene ID and Gene Symbol:** The parent gene of mRNA and gene name. **Start/End:** The full-length start or termination site of mRNA in the genome. **Strand:** The feature of the strand in the host gene forming mRNA; "+" indicates the sense strand, and "-" indicates the antisense strand. **log2(FC):** The log scale of fold change (the absolute ratio of normalized intensities between ICH and Sham groups). ***P* Value:** Significance index of statistical difference. ***Q* Value:** The corrected *P* Value. **ICH 1 to 4** and **Sham 1 to 4:** Read count after FPKM normalization correction of each sample.

Day-7 mRNA	Chr	Gene ID	Gene Symbol	Start	End	Strand	log2(FC)	*P* Value	*Q* Value	ICH1	ICH2	ICH3	ICH4	Sham1	Sham2	Sham3	Sham4
ENSRNOT00000001759	chr20	ENSRNOG00000001302	Adora2a	14265252	14282873	-	-1.0403	0.0007	0.0399	20.4711	29.7152	14.2578	2.5264	30.4280	39.2949	33.2895	34.7205
ENSRNOT00000002310	chr11	ENSRNOG00000001701	Cbr3	33909439	33917674	+	-1.0223	0.0010	0.0477	4.4974	5.7199	3.5993	1.9692	6.2478	9.1812	8.9894	7.6447
ENSRNOT00000003024	chr14	ENSRNOG00000002224	Yipf7	62567745	62595854	-	-Inf	0.0000	0.0034	0	0	0	0	0.1502	0.1077	0.1420	0
ENSRNOT00000003125	chr14	ENSRNOG00000002278	Tec	37919233	38027925	+	-2.0947	0.0008	0.0434	0.4107	2.9491	0.0729	0.0056	3.0263	4.4561	4.4101	2.7940
ENSRNOT00000003687	chr10	ENSRNOG00000002669	Cluh	61432819	61453530	+	-1.6428	0.0004	0.0266	3.8935	4.9654	4.3963	4.2939	26.2633	24.8539	3.0106	0.6729
ENSRNOT00000004183	chr13	ENSRNOG00000003136	Fcrla	89296573	89306219	-	-Inf	0.0007	0.0400	0	0	0	0	0	0	0.5858	0
ENSRNOT00000004347	chr13	ENSRNOG00000003209	Pcp4l1	89542378	89565813	-	-0.8887	0.0003	0.0247	18.9179	24.6773	22.8630	8.5755	24.5785	34.4856	51.8732	27.9921
ENSRNOT00000005016	chr10	ENSRNOG00000003495	Prpf8	63635251	63658360	+	-Inf	0.0000	0.0000	0	0	0	0	0	31.2920	38.9593	0
ENSRNOT00000005988	chr6	ENSRNOG00000004498	Scin	59976003	60054279	-	-3.8995	0.0004	0.0288	0	0.0281	0	0	0	0.1069	0.2740	0.0389
ENSRNOT00000006741	chrX	ENSRNOG00000004322	Sh3kbp1	37791483	38196060	-	-Inf	0.0001	0.0116	0	0	0	0	0	0	0.7263	0
ENSRNOT00000006832	chr6	ENSRNOG00000005124	Plekhh2	7793855	7893519	+	-Inf	0.0002	0.0166	0	0	0	0	0	0	0.0190	0.1262
ENSRNOT00000007398	chr7	ENSRNOG00000004860	Myh9l1	118741110	118792625	-	-Inf	0.0000	0.0001	0	0	0	0	0	0.6029	0	1.0387
ENSRNOT00000007602	chr6	ENSRNOG00000005776	Bcl11b	131836674	131914028	-	-3.1807	0.0002	0.0182	0.0137	0.3832	0.0330	0.0222	0.0796	0.0484	0.2522	3.7182
ENSRNOT00000007747	chr7	ENSRNOG00000005825	Lyz2	60335969	60341264	-	-1.1244	0.0001	0.0123	36.5017	46.4234	29.0118	34.6105	37.4560	94.6489	155.1567	32.2222
ENSRNOT00000008673	chr7	ENSRNOG00000028713	Acvrl1	142776252	142787335	+	-1.9154	0.0004	0.0266	0.5591	0.3410	0.0109	0.3835	1.5622	1.5808	1.1638	0.5760
ENSRNOT00000009228	chr5	ENSRNOG00000006487	Casp8ap2	47853818	47891127	+	-3.0782	0.0000	0.0051	0.0844	0.0425	0.0392	0.0102	0.2688	1.1267	0.0292	0.0632
ENSRNOT00000009370	chr2	ENSRNOG00000028348	Lhx8	260574190	260596777	-	-1.4274	0.0009	0.0445	0.7989	0.9829	0.7273	0.2579	1.8188	1.8149	3.0078	0.8004
ENSRNOT00000009515	chr4	ENSRNOG00000007178	Cd8a	99239115	99243351	+	-2.3589	0.0005	0.0310	1.6044	1.0473	0.1270	0.2068	0.3087	12.2836	2.3006	0.4224
ENSRNOT00000009823	chr7	ENSRNOG00000007489	Zfp41	116655145	116655741	+	-3.5490	0.0001	0.0115	0.0822	0.0976	0.0954	0.0980	4.0919	0.1029	0.0786	0.0943
ENSRNOT00000010467	chr7	ENSRNOG00000007133	Plxnb2	130135484	130151414	-	-1.6437	0.0001	0.0138	1.7659	2.4657	0.2570	1.8599	7.5186	2.2849	8.8534	1.1800
ENSRNOT00000010930	chr13	ENSRNOG00000008054	Igsf9	90815562	90832138	-	-Inf	0.0000	0.0002	0	0	0	0	0	0.6074	0	0.5942

ENSRNOT00000010991	chr8	ENSRNOG00000007926	Map2k5	68055977	68282590	-	-Inf	0.0000	0.0000	0	0	0	0	0.7797	0.7817	0.8725	0.6357
ENSRNOT00000011073	chr10	ENSRNOG00000008134	Mefv	12046701	12056222	+	-Inf	0.0000	0.0038	0	0	0	0	0	0.0550	0.0419	0.0636
ENSRNOT00000011727	chr8	ENSRNOG00000030187	Mmp12	5606592	5616493	+	-2.6196	0.0002	0.0173	0.2476	0.2267	0.0852	0	0.2727	0.8432	1.9991	0.3239
ENSRNOT00000011945	chr4	ENSRNOG00000008816	Gpnmb	78694447	78715683	+	-1.6305	0.0000	0.0017	17.1791	28.7111	7.6976	9.6145	20.3921	80.8490	74.4751	19.9669
ENSRNOT00000012568	chr3	ENSRNOG00000009399	Fads2l1	72854475	72895740	-	-Inf	0.0002	0.0179	0	0	0	0	0.0224	0.0225	0.0216	0
ENSRNOT00000013408	chr15	ENSRNOG00000010063	Htr2a	56666012	56735382	+	-0.8307	0.0002	0.0194	11.9114	6.3385	8.2058	5.2371	11.6951	12.1600	13.7227	18.7881
ENSRNOT00000013836	chr10	ENSRNOG00000047940	LOC103694864	70660691	70681672	-	-Inf	0.0007	0.0389	0	0	0	0	0	0.4648	0	0
ENSRNOT00000013936	chr8	ENSRNOG00000009907	Mmp8	5768811	5778702	+	-2.8452	0.0002	0.0158	0.1974	0.1688	0	0.0340	0.2712	0.3967	2.0346	0.1736
ENSRNOT00000014273	chr15	ENSRNOG00000010516	Plau	3644769	3650819	-	-1.9825	0.0000	0.0058	1.0033	1.4756	0.5198	0.7053	1.2825	5.0962	7.1075	1.1510
ENSRNOT00000015127	chr16	ENSRNOG00000011016	Slc7a2	54460067	54513349	-	-2.3808	0.0005	0.0332	0.0097	0.0373	0.0277	0.0538	0.4621	0.0830	0.0713	0.0535
ENSRNOT00000015325	chr4	ENSRNOG00000011175	Hnrnpa2b1	81237496	81241152	-	-1.3111	0.0003	0.0224	2.1553	4.6493	4.4887	5.9739	5.4308	8.0336	10.0446	19.3363
ENSRNOT00000015778	chr15	ENSRNOG00000011704	Fbxo34	24267323	24334885	+	-5.2034	0.0000	0.0000	0.0233	0	0.0022	0.0390	0.5842	0.2552	1.1798	0.3594
ENSRNOT00000016352	chr5	ENSRNOG00000012274	Ddi2	160236952	160282810	-	-0.9746	0.0000	0.0062	6.8591	5.8503	5.5063	6.9845	6.9554	13.5391	14.1932	14.8328
ENSRNOT00000017065	chr5	ENSRNOG00000012804	C1qc	155255005	155258392	-	-1.0507	0.0004	0.0288	55.8619	46.7551	18.5485	29.4443	48.9714	161.7356	68.5688	32.7248
ENSRNOT00000017358	chr19	ENSRNOG00000013007	Il17c	55246926	55248205	+	-Inf	0.0000	0.0057	0	0	0	0	0.0549	0.1891	0.0370	0.1561
ENSRNOT00000017385	chr5	ENSRNOG00000012807	C1qa	155261250	155264143	-	-0.8825	0.0005	0.0330	86.4360	87.8471	35.3907	54.4757	77.9639	222.2794	121.4079	65.3101
ENSRNOT00000017601	chr1	ENSRNOG00000013190	Rnaset2	53174879	53192048	+	-0.8600	0.0003	0.0214	41.1918	35.8370	22.3538	33.0337	38.8096	91.4077	69.3357	40.7890
ENSRNOT00000017828	chr2	ENSRNOG00000013282	Mctp1	3662763	4088106	+	-1.7483	0.0006	0.0361	0.3443	0.1434	0.2497	0.0833	1.1840	0.4718	0.7295	0.3721
ENSRNOT00000018351	chr9	ENSRNOG00000013578	Trem2	14611561	14618013	-	-0.9384	0.0006	0.0346	9.6856	12.7583	5.9812	9.5482	11.1417	26.6791	22.7882	12.1610
ENSRNOT00000018630	chr16	ENSRNOG00000013330	Cdhr1	14328160	14348046	-	-1.1908	0.0001	0.0091	2.0037	2.9866	2.0869	0.9887	4.9449	3.4000	6.5510	3.5164
ENSRNOT00000018854	chr16	ENSRNOG00000014055	Nat1	23970743	23991573	-	-Inf	0.0001	0.0077	0	0	0	0	1.1558	0	0	0
ENSRNOT00000019929	chr2	ENSRNOG00000014793	Gpr149	152839117	152911553	-	-1.1179	0.0004	0.0264	1.6286	1.6099	1.6217	0.6530	3.1335	2.4716	4.0001	2.3602
ENSRNOT00000020017	chr19	ENSRNOG00000014761	Rasd2	14653198	14663914	+	-0.7401	0.0004	0.0292	81.5041	103.9925	45.1202	32.9002	98.5607	115.4898	100.5592	125.5454
ENSRNOT00000021938	chr2	ENSRNOG00000016164	Fcrl2	186595051	186606172	-	-1.3787	0.0007	0.0372	7.1760	5.6725	1.3409	10.3359	13.4597	23.7182	24.1051	2.4900
ENSRNOT00000021972	chr18	ENSRNOG00000026136	Tnfaip8	44737154	44779914	+	-2.3355	0.0007	0.0394	0.1682	0.0866	0.1474	0.2171	0.1234	0.1386	2.7585	0.1056
ENSRNOT00000022002	chr5	ENSRNOG00000016364	Gba2	59068366	59079719	-	-3.4210	0.0000	0.0016	0.4133	0.2120	0.4724	0.4872	0.3725	0.3001	0.6110	15.6915
ENSRNOT00000022292	chr2	ENSRNOG00000016550	Dclk2	186119729	186245361	-	-1.1049	0.0002	0.0152	5.0352	6.7528	4.4590	3.6881	13.2316	3.5622	14.0581	12.0248
ENSRNOT00000022485	chr9	ENSRNOG00000016653	Ngef	94569286	94601852	-	-0.6629	0.0003	0.0225	118.0221	82.7852	67.7157	57.3634	109.0847	129.9241	121.4392	155.5109

ENSRNOT00000023079	chr2	ENSRNOG00000017187	LOC108348173	213173403	213178632	-	-Inf	0.0000	0.0003	0	0	0	0	0.1106	0	0	1.9686
ENSRNOT00000026225	chr16	ENSRNOG00000019387	Ifi30	20426566	20430752	+	-0.9376	0.0008	0.0427	11.4340	16.3383	7.5190	9.5507	10.8965	35.9213	26.8271	12.2413
ENSRNOT00000026392	chr16	ENSRNOG00000062013	Adprhl1	81616604	81632030	+	-1.6079	0.0005	0.0335	0.2651	0.3205	0.5103	0.3179	0.7012	0.7165	1.3082	1.5832
ENSRNOT00000026451	chr3	ENSRNOG00000019467	Fer1l4	151564604	151600140	-	-Inf	0.0001	0.0112	0	0	0	0	0	0.0239	0.0063	0.0266
ENSRNOT00000026657	chr3	ENSRNOG00000050864	LOC100910990	151616037	151625644	-	-Inf	0.0000	0.0005	0	0	0	0	0	0	5.8713	0
ENSRNOT00000026893	chr7	ENSRNOG00000019857	Gng7	11582984	11628929	+	-0.8394	0.0000	0.0064	54.7331	79.8187	46.4323	27.9470	84.5777	96.0824	94.3897	98.7985
ENSRNOT00000028557	chr10	ENSRNOG00000021031	Grn	90376933	90383205	+	-0.9037	0.0001	0.0137	54.2122	50.4259	23.7330	34.6698	52.9208	123.1242	81.9900	46.9904
ENSRNOT00000028688	chr1	ENSRNOG00000021129	RGD1308428	90098387	90151405	-	-7.0169	0.0000	0.0000	0.0043	0.0028	0.0593	0.0011	0.2539	0.0419	3.4063	5.0458
ENSRNOT00000028732	chr2	ENSRNOG00000021157	Ctss	197655786	197679458	+	-0.6403	0.0002	0.0197	117.5104	120.8541	84.6637	110.8432	146.8310	218.7656	167.7899	142.8488
ENSRNOT00000029208	chr7	ENSRNOG00000027468	Slc6a15	45328105	45381920	+	-3.4739	0.0000	0.0028	0.0737	0.0170	0.0068	0.0038	0.2702	0.0273	0.3648	0.4626
ENSRNOT00000030036	chr15	ENSRNOG00000027839	Ptk2b	42827846	42898150	-	-1.4729	0.0000	0.0027	2.5497	2.2440	1.9581	2.1055	4.2167	3.7913	3.9622	12.6150
ENSRNOT00000030327	chr1	ENSRNOG00000021478	Tpd52l1	28455051	28576553	+	-3.8129	0.0000	0.0020	1.1542	0.2453	0.0379	0	0.8228	9.5415	8.7148	1.1226
ENSRNOT00000030893	chr17	ENSRNOG00000023688	Drd1	11101306	11103541	+	-1.0094	0.0003	0.0243	23.0482	28.4097	13.0767	4.4478	29.3475	37.5142	41.2206	30.7855
ENSRNOT00000032189	chr18	ENSRNOG00000025502	Arhgef37	56817988	56870839	-	-3.6874	0.0000	0.0000	0.0337	0.1306	0.0423	0.0654	1.0088	0.1195	1.1109	1.2636
ENSRNOT00000032783	chr2	ENSRNOG00000023588	Dmgdh	23289391	23370353	+	-Inf	0.0000	0.0053	0	0	0	0	0	0	0	0.9488
ENSRNOT00000033048	chr15	ENSRNOG00000024061	Rarb	10120206	10262599	+	-0.8726	0.0006	0.0350	5.0294	6.1556	3.7637	2.0521	6.9172	8.8966	7.9060	7.4081
ENSRNOT00000033265	chr4	ENSRNOG00000026306	Clec5a	68810891	68819872	-	-4.2116	0.0001	0.0089	0	0	0.0191	0.0623	0.0210	0.0387	0.9436	0.5052
ENSRNOT00000035400	chr13	ENSRNOG00000046452	Fcgr2b	89329304	89343899	-	-1.2663	0.0005	0.0328	2.7231	3.3364	1.2926	2.7228	1.9712	7.4963	8.3462	6.4200
ENSRNOT00000035531	chr1	ENSRNOG00000025651	Ikzf5	201963204	201981250	-	-0.9721	0.0002	0.0152	5.2644	5.1687	4.7955	4.7744	11.9331	12.0815	4.6266	10.6000
ENSRNOT00000036040	chr13	ENSRNOG00000003486	Mnda	92073668	92089980	-	-2.7026	0.0006	0.0341	0.1623	0.0488	0.0467	0	0.1547	1.2325	0.2414	0.0494
ENSRNOT00000036447	chr10	ENSRNOG00000027852	RGD1311343	15947253	15960673	-	-Inf	0.0002	0.0187	0	0	0	0	0.0472	0	0.0401	0.0210
ENSRNOT00000036791	chr1	ENSRNOG00000042679	AABR07006860.1	260798239	260804901	+	-4.3511	0.0000	0.0001	0.1528	0.2092	0.1968	0.1929	0.1689	0.1866	7.5789	7.4070
ENSRNOT00000037199	chr8	ENSRNOG00000008919	Arpp21	120439994	120446507	-	-0.5989	0.0006	0.0347	129.6202	112.2594	97.7140	61.5652	129.7668	166.2930	158.7451	152.7826
ENSRNOT00000037576	chr3	ENSRNOG00000026036	Pdyn	122194329	122206671	-	-0.7020	0.0005	0.0326	19.9357	15.8794	16.3957	13.2240	25.7217	28.7509	34.7571	17.2204
ENSRNOT00000037752	chr10	ENSRNOG00000028404	Ppp1r1b	86303727	86312762	+	-0.8727	0.0003	0.0216	233.9733	285.8908	145.6498	58.5182	279.7371	360.2778	341.8056	343.9315
ENSRNOT00000038006	chr13	ENSRNOG00000023209	Slamf7	90058127	90074952	-	-5.3224	0.0001	0.0096	0	0	0	0.0158	0	0.3823	0.1603	0.0883
ENSRNOT00000038589	chr2	ENSRNOG00000027894	Iqgap3	187447501	187489630	+	-1.0472	0.0011	0.0499	0.7686	1.1959	0.7750	0.3246	1.2523	1.6984	1.8898	1.4914
ENSRNOT00000038798	chr1	ENSRNOG00000021130	Abcc8	102110708	102191287	-	-Inf	0.0000	0.0046	0	0	0	0	0	0.6773	0	0

ENSRNOT00000038994	chrX	ENSRNOG00000003622	Cybb	14578264	14612547	+	-1.1034	0.0010	0.0479	4.1149	3.0218	1.4963	1.7908	3.0676	10.7421	6.8249	1.7616
ENSRNOT00000039876	chr11	ENSRNOG00000001959	Mx1	37891156	37914983	-	-1.2470	0.0005	0.0322	9.9727	7.1992	1.6550	4.1539	17.8074	21.7665	11.8701	3.0999
ENSRNOT00000040202	chr11	ENSRNOG00000030930	Samsn1	14254047	14304603	-	-Inf	0.0000	0.0000	0	0	0	0	0.3238	0.2525	0.3723	0.0956
ENSRNOT00000040759	chr16	ENSRNOG00000013147	Cacna1d	6113168	6245644	-	-6.2321	0.0004	0.0285	0.0354	0	0	0	2.6627	0	0	0
ENSRNOT00000040884	chr11	ENSRNOG00000038540	NEWGENE_2724	74014983	74018075	+	-Inf	0.0008	0.0430	0	0	0	0	0	0	0.2973	0
ENSRNOT00000041186	chr2	ENSRNOG00000015425	Ssx2ip	252263487	252296031	+	-6.9243	0.0002	0.0205	0	0.0072	0	0	0	0	0	0.8803
ENSRNOT00000041994	chr16	ENSRNOG00000013884	Psd3	23553647	23781604	+	-10.8919	0.0000	0.0006	0.0051	0	0.0039	0.0037	0	0	0	24.0636
ENSRNOT00000042539	chr20	ENSRNOG00000000503	Ppard	7818289	7883482	+	-Inf	0.0000	0.0004	0	0	0	0	0	0	0	7.0906
ENSRNOT00000043661	chr3	ENSRNOG00000009371	Abl1	9410661	9447813	-	-4.6594	0.0007	0.0375	0	0.0037	0.0027	0	0.0029	0.0041	0.0026	0.1517
ENSRNOT00000043852	chr17	ENSRNOG00000031716	LOC100910978	15533972	15555919	-	-3.4327	0.0007	0.0394	0.0061	0.0305	0.0181	0.0091	0.6528	0.0133	0.0235	0
ENSRNOT00000043917	chr11	ENSRNOG00000002046	Cd200r1l	60804311	60819249	-	-Inf	0.0008	0.0428	0	0	0	0	0	0.0800	0.1004	0.0050
ENSRNOT00000046246	chr6	ENSRNOG00000008479	Slc8a1	4258991	4520604	-	-Inf	0.0000	0.0000	0	0	0	0	0	5.7213	1.8173	0
ENSRNOT00000046456	chr6	ENSRNOG00000004711	Mta1	137924568	137950066	+	-Inf	0.0000	0.0000	0	0	0	0	0	5.6374	6.8809	2.0856
ENSRNOT00000047200	chr5	ENSRNOG00000033110	Svep1	75143867	75319189	-	-7.4012	0.0001	0.0076	0.0012	0.0026	0	0	0	0.6373	0	0
ENSRNOT00000047241	chr7	ENSRNOG00000031916	Timeless	2643288	2667321	+	-Inf	0.0006	0.0346	0	0	0	0	0	0	0	0.1919
ENSRNOT00000049180	chr11	ENSRNOG00000002229	Adcy5	68695839	68842320	-	-0.5972	0.0007	0.0380	73.3825	88.6819	60.1823	37.3160	91.4645	101.4018	96.9418	102.8459
ENSRNOT00000049574	chr1	ENSRNOG00000030689	Ms4a6bl	227757497	227769322	+	-2.9713	0.0003	0.0238	0	0.3607	0	0.6002	1.0170	2.5356	3.6894	0.2932
ENSRNOT00000050393	chr1	ENSRNOG00000016063	Vps35l	188448604	188552265	+	-Inf	0.0000	0.0007	0	0	0	0	0	0	0	4.7945
ENSRNOT00000051352	chr19	ENSRNOG00000017905	Map1lc3b	53629779	53646055	+	-4.8336	0.0000	0.0000	0.3784	0.0768	0.1647	0.1931	7.9725	8.0583	0.2995	6.8528
ENSRNOT00000051589	chr1	ENSRNOG00000028982	Mrgprb3	103897041	103909926	-	-Inf	0.0006	0.0367	0	0	0	0	0.0284	0	0.0106	0.0090
ENSRNOT00000052017	chr4	ENSRNOG00000007090	Cacna1c	150642534	150829741	-	-1.6075	0.0007	0.0403	1.0015	0.2173	0.1059	0.0831	1.3692	1.1538	1.0707	0.6960
ENSRNOT00000055027	chr1	ENSRNOG00000018311	Cd19	197815413	197821936	-	-Inf	0.0006	0.0366	0	0	0	0	0	0.0322	0.0154	0.0154
ENSRNOT00000055473	chr4	ENSRNOG00000001830	Arntl2	181103774	181158200	+	-Inf	0.0009	0.0444	0	0	0	0	0	0	0	0.3850
ENSRNOT00000055829	chr1	ENSRNOG00000012716	Chd2	134760842	134870255	-	-6.9056	0.0000	0.0000	0.0097	0.0388	0.0175	0.0179	0.0393	0.0505	0.0158	9.9459
ENSRNOT00000056450	chr3	ENSRNOG00000042446	Ankrd63	110491226	110492398	-	-0.7600	0.0008	0.0410	14.3663	15.6516	13.9325	8.1850	20.5477	21.8413	25.4013	20.4993
ENSRNOT00000056562	chr8	ENSRNOG00000014871	Zic4	98755104	98760737	+	-5.1436	0.0000	0.0017	0	0.0162	0.0077	0.0054	0.4024	0.6005	0.0109	0.0180
ENSRNOT00000056652	chr2	ENSRNOG00000020657	Shc1	188745503	188757066	+	-1.1235	0.0006	0.0342	0.8650	1.4118	0.9143	0.9942	1.5206	2.7703	2.6689	2.1587
ENSRNOT00000056918	chr10	ENSRNOG00000037563	Cd68	56268720	56270640	-	-1.3517	0.0010	0.0482	9.0476	8.5177	2.4046	2.9329	4.7742	33.8930	15.8532	3.9307

ENSRNOT00000057378	chrX	ENSRNOG00000037799	Hdx	82875476	83151511	-	-5.3473	0.0000	0.0019	0	0.0187	0	0	0.5972	0.0103	0.0465	0.1057
ENSRNOT00000057724	chr2	ENSRNOG00000009955	Plch1	154256392	154334356	-	-Inf	0.0000	0.0000	0	0	0	0	0	0.7819	0.5693	0.0025
ENSRNOT00000058402	chr11	ENSRNOG00000038355	Crygs	81796891	81802172	+	-3.7604	0.0005	0.0324	0	0.0146	0.0128	0	0.0954	0.1176	0.0940	0.0651
ENSRNOT00000058414	chr14	ENSRNOG00000006400	Tbc1d14	79339258	79438764	+	-2.3594	0.0011	0.0499	0.0392	0.1032	0.7104	0.0135	0.0128	0.3512	2.0466	2.0350
ENSRNOT00000059078	chr8	ENSRNOG00000025624	Arhgap20	56179816	56261194	+	-2.5613	0.0001	0.0077	0.0327	0.0579	0.0197	0.0454	0.4227	0.0406	0.2022	0.2533
ENSRNOT00000060515	chr14	ENSRNOG00000050800	LOC100911576	88543630	88574195	+	-2.1392	0.0001	0.0106	0.7839	0.9380	0.9596	0.6131	1.3198	1.2016	0.7893	11.2025
ENSRNOT00000061303	chr3	ENSRNOG00000008810	Nsmf	2262321	2270743	+	-3.3450	0.0008	0.0410	0	0.2749	0.1372	0.1026	0.0711	0	5.0252	0.1336
ENSRNOT00000061858	chr4	ENSRNOG00000021441	Reln	9347779	9773217	+	-2.6971	0.0004	0.0279	0.0951	0.1101	0.1361	0.1110	0.0971	0.0976	2.7375	0.0006
ENSRNOT00000063967	chr8	ENSRNOG00000006545	7-Sep	26413233	26476611	+	-2.1212	0.0000	0.0041	6.0644	37.9277	6.3915	3.9260	80.5372	5.0873	52.2934	98.3662
ENSRNOT00000064447	chr9	ENSRNOG00000042181	Dytn	70320443	70374553	-	-Inf	0.0009	0.0435	0	0	0	0	0.0296	0.0205	0.0393	0
ENSRNOT00000065079	chr14	ENSRNOG00000042714	RGD1559459	22937421	22952822	+	-3.0160	0.0003	0.0219	0.0434	0	0	0.0262	0.0970	0.0994	0.1215	0.2458
ENSRNOT00000065386	chr1	ENSRNOG00000016728	Tiam2	44311513	44433193	+	-0.6089	0.0008	0.0431	9.9085	7.7354	7.9036	6.8484	10.1167	11.8553	13.2742	14.1614
ENSRNOT00000065644	chr15	ENSRNOG00000009783	Camk2g	3938075	3995915	+	-1.9445	0.0010	0.0480	0.0553	0.0489	0.0670	0.0505	0.3673	0.0350	0.4220	0.0292
ENSRNOT00000066048	chr18	ENSRNOG00000017503	Ppargc1b	56626736	56728185	-	-7.6323	0.0000	0.0012	0.0018	0.0010	0.0032	0.0025	0.0042	0.0038	1.6809	0.0028
ENSRNOT00000066233	chr7	ENSRNOG00000043439	Zfp707l1	117001084	117007662	+	-4.1920	0.0001	0.0089	0.0194	0.0157	0.0423	0.0093	0.2218	1.3321	0.0112	0.0196
ENSRNOT00000066279	chr3	ENSRNOG00000005281	Stx16	172155496	172182855	+	-5.7235	0.0000	0.0049	0.0016	0.0018	0.0094	0.0017	0.6546	0.1060	0.0011	0.0013
ENSRNOT00000067070	chr1	ENSRNOG00000031700	Map3k5	15412603	15613746	+	-Inf	0.0001	0.0078	0	0	0	0	0	0	0.5292	0
ENSRNOT00000067188	chr10	ENSRNOG00000003399	Hnrnph1	35870682	35879910	+	-1.4552	0.0007	0.0403	0.8073	3.2166	1.5478	1.6146	1.4320	9.9128	7.0120	1.3473
ENSRNOT00000067616	chr5	ENSRNOG00000014241	Ece1	156215417	156318671	+	-1.7130	0.0002	0.0161	1.5966	0.6707	1.2539	1.4362	1.2744	12.0622	1.5517	1.3637
ENSRNOT00000067689	chr15	ENSRNOG00000009074	AABR07017902.1	32811135	32856430	+	-Inf	0.0005	0.0320	0	0	0	0	0	0.8728	0	0
ENSRNOT00000067875	chr14	ENSRNOG00000043451	Spp1	6673686	6679901	-	-1.9206	0.0000	0.0012	18.0903	26.5390	21.0072	16.1174	27.0863	54.6371	209.8884	17.8840
ENSRNOT00000068134	chr14	ENSRNOG00000002045	Anxa3	14371928	14426437	-	-0.6607	0.0009	0.0443	22.4534	28.2923	13.4816	18.3556	23.6528	42.9096	37.4311	26.5574
ENSRNOT00000070821	chr2	ENSRNOG00000031576	Zfp455	86996798	87011171	+	-Inf	0.0000	0.0000	0	0	0	0	1.1107	0	0.9066	0.6481
ENSRNOT00000070855	chr7	ENSRNOG00000048982	LOC100912282	144308345	144319804	-	-Inf	0.0000	0.0017	0	0	0	0	3.5578	0	0	0
ENSRNOT00000071399	chr20	ENSRNOG00000046758	LOC102547645	8202355	8202924	-	-Inf	0.0009	0.0443	0	0	0	0	0.1090	0.0355	0	0.0712
ENSRNOT00000071532	chr8	ENSRNOG00000045905	LOC108348070	13511805	13513337	-	-2.3530	0.0004	0.0259	0.0324	0.1383	0.0796	0.0545	0.6969	0.0382	0.5060	0.3163
ENSRNOT00000071615	chr10	ENSRNOG00000045816	Gria1	42618487	42760200	+	-Inf	0.0000	0.0003	0	0	0	0	0	0	14.1087	0
ENSRNOT00000071663	chr20	ENSRNOG00000049507	10-Sep	28722580	28783589	-	-Inf	0.0003	0.0214	0	0	0	0	0	0	0.8249	0

ENSRNOT00000071885	chr7	ENSRNOG00000049033	Racgap1	141277312	141307233	-	-1.6171	0.0001	0.0131	0.4201	0.6186	0.3537	0.2117	1.1071	1.4088	0.8616	1.5434
ENSRNOT00000071977	chr6	ENSRNOG00000050553	Atp6v1c2	42585997	42630983	-	-1.9216	0.0003	0.0227	0.1364	0.1301	0.2607	0.1380	0.4824	0.3923	0.7882	0.8571
ENSRNOT00000072054	chr9	ENSRNOG00000058883	NEWGENE_1589866	90787787	90806126	-	-Inf	0.0010	0.0490	0	0	0	0	0.2006	0	0	0.0113
ENSRNOT00000072069	chr3	ENSRNOG00000050697	Ctsz	172527107	172537877	-	-0.8722	0.0010	0.0478	30.4431	30.0551	12.9460	17.3655	24.2395	70.8407	49.6847	21.4580
ENSRNOT00000072945	chr3	ENSRNOG00000045686	Nfs1	151665814	151688149	-	-1.6724	0.0002	0.0167	1.1016	2.1848	0.3829	7.8886	9.0372	10.0243	8.5565	9.2222
ENSRNOT00000073192	chr5	ENSRNOG00000050760	LOC103690035	64566804	64584309	+	-6.7680	0.0002	0.0170	0	0	0	0.0833	0	0	0	9.0829
ENSRNOT00000073276	chr20	ENSRNOG00000049580	Gpr6	45813169	45815940	-	-0.9998	0.0009	0.0453	7.5963	9.2845	5.2590	1.3435	10.8270	14.0289	12.1159	9.9871
ENSRNOT00000073534	chr17	ENSRNOG00000046647	Impad1	90188043	90217786	-	-4.5565	0.0010	0.0470	0	0.0496	0.0252	0.0203	2.2396	0	0	0
ENSRNOT00000073677	chrX	ENSRNOG00000050465	AABR07041109.1	119532513	119532965	-	-1.1127	0.0005	0.0307	5.2677	8.7188	7.4145	7.6911	15.9524	14.0930	17.1808	15.6852
ENSRNOT00000073853	chr15	ENSRNOG00000009074	AABR07017902.1	32817343	32856421	+	-Inf	0.0000	0.0035	0	0	0	0	0.3204	0.0231	0	0.3903
ENSRNOT00000074074	chr5	ENSRNOG00000050636	Arhgef10l	159018893	159119171	-	-12.5017	0.0000	0.0000	0.0013	0.0014	0.0009	0.0011	0.0016	13.6561	12.9789	0.0010
ENSRNOT00000074082	chr10	ENSRNOG00000048771	RGD1559482	104952458	104957149	+	-2.2608	0.0002	0.0171	0.4099	0.8407	0.2017	0.2578	0.3747	4.1860	2.6143	1.0203
ENSRNOT00000074108	chr7	ENSRNOG00000047194	Arl13b	1122567	1188209	-	-1.9584	0.0001	0.0108	0.3103	0.4935	1.0958	0.8306	4.8812	4.5053	0.7463	0.4771
ENSRNOT00000074915	chr9	ENSRNOG00000049797	NEWGENE_1311658	101307764	101319845	-	-Inf	0.0003	0.0250	0	0	0	0	0	0.1256	0.3853	0
ENSRNOT00000075678	chr7	ENSRNOG00000048636	Il2rb	119701367	119716238	-	-4.6779	0.0008	0.0433	0	0.0137	0.0065	0	0	0.5113	0	0.0055
ENSRNOT00000075906	chr4	ENSRNOG00000010609	Abcf2	7129932	7134361	+	-Inf	0.0000	0.0018	0	0	0	0	1.0404	0	0	1.3708
ENSRNOT00000075987	chr12	ENSRNOG00000000989	Bud31	11232964	11240399	-	-3.7514	0.0008	0.0417	0	0	0	0.0526	0.1198	0.4464	0.1429	0
ENSRNOT00000076274	chr2	ENSRNOG00000009955	Plch1	154256380	154418629	-	-Inf	0.0000	0.0031	0	0	0	0	0.0511	0	0	0.1885
ENSRNOT00000076671	chrX	ENSRNOG00000002904	Arr3	70438617	70452067	+	-Inf	0.0004	0.0258	0	0	0	0	0.0039	0.0331	0.0433	0.0419
ENSRNOT00000076699	chrX	ENSRNOG00000006642	Yipf6	68627313	68637027	+	-5.0721	0.0001	0.0074	0	0	0.0330	0.0373	0	2.2808	0.0160	0.0693
ENSRNOT00000076730	chr14	ENSRNOG00000051169	Clnk	76657311	76833161	+	-Inf	0.0005	0.0319	0	0	0	0	0	0.0350	0.0238	0.0707
ENSRNOT00000076737	chr16	ENSRNOG00000014464	Tnfsf13b	85275678	85305839	-	-2.8310	0.0001	0.0125	0	0.0183	0.2704	0.1183	0.4203	1.3848	0.6973	0.3937
ENSRNOT00000076998	chr7	ENSRNOG00000043465	Arc	115907097	115910522	-	-0.6568	0.0005	0.0314	18.4855	17.0026	22.4314	31.0473	37.6610	37.9045	31.3806	33.3210
ENSRNOT00000077091	chr3	ENSRNOG00000004730	Meis2	107559997	107760550	-	-0.8521	0.0000	0.0062	25.0221	30.7899	22.1217	12.7683	40.3022	39.2549	48.1717	35.9972
ENSRNOT00000077788	chr3	ENSRNOG00000030763	Dpp4	48292739	48372609	-	-Inf	0.0000	0.0004	0	0	0	0	0.4904	0.6368	0	0
ENSRNOT00000077953	chr17	ENSRNOG00000015585	Suv39h2	78764506	78779545	+	-2.9336	0.0008	0.0407	0.0331	0.0388	0	0.0381	0.0059	0.4511	0.3034	0.0795
ENSRNOT00000078017	chr2	ENSRNOG00000052613	Casp6	235341365	235353967	+	-Inf	0.0004	0.0265	0	0	0	0	0	0.5190	0	0
ENSRNOT00000078871	chr20	ENSRNOG00000047657	C4a	2651599	2652952	-	-5.3458	0.0000	0.0058	0.1605	0	0	0	5.5218	0	0.6712	0.3320

ENSRNOT00000078891	chr8	ENSRNOG00000000204	Syncrip	96107240	96266342	-	-2.6586	0.0000	0.0058	0.1253	0.1189	0.9695	0.1061	2.5516	1.8971	3.5085	0.3754
ENSRNOT00000079189	chr1	ENSRNOG00000020543	Nosip	101055622	101072938	+	-3.3662	0.0003	0.0252	0.0782	0.2665	0.1156	0.1094	0.1609	0.0475	5.6663	0
ENSRNOT00000079501	chr7	ENSRNOG00000029762	Acr	130542202	130548251	+	-Inf	0.0001	0.0087	0	0	0	0	0.0671	0.0964	0.0747	0
ENSRNOT00000079684	chr9	ENSRNOG00000011000	Rims1	28442813	28972835	-	-3.4989	0.0000	0.0038	0.0104	0.0227	0	0.0305	0.2271	0.0106	0.3270	0.1536
ENSRNOT00000080101	chr17	ENSRNOG00000016767	Ggps1	53962444	53971443	+	-6.1071	0.0000	0.0002	0.0154	0.0184	0.0162	0.0089	0.0204	4.0064	0.0147	0.0214
ENSRNOT00000080724	chr7	ENSRNOG00000011781	Oplah	117353786	117364322	-	-2.5775	0.0002	0.0189	0.3095	0.5398	0.4250	0.3012	0.3214	8.6756	0.0407	0.3669
ENSRNOT00000080820	chr1	ENSRNOG00000053260	Lilrb3a	63842382	63849065	+	-8.4441	0.0000	0.0017	0	0.0052	0	0	1.6210	0	0	0.1817
ENSRNOT00000081085	chr20	ENSRNOG00000000467	Ring1	3812287	3813673	-	-4.1819	0.0000	0.0014	0	0	0.1526	0	0.7964	0.7854	0.5728	0.6148
ENSRNOT00000081113	chr20	ENSRNOG00000000632	Cdk1	20577561	20591549	+	-5.4914	0.0000	0.0012	0.0198	0.0090	0	0.0182	0.7868	1.2604	0.0144	0.0514
ENSRNOT00000081321	chr16	ENSRNOG00000016256	Myo9b	19686630	19753133	+	-3.4760	0.0000	0.0024	0.0098	0.3369	0.0563	0.6178	0.3010	1.2415	9.4879	0.3271
ENSRNOT00000081440	chr10	ENSRNOG00000053047	Top2a	86901467	86930947	-	-Inf	0.0000	0.0011	0	0	0	0	0	0	2.0827	0
ENSRNOT00000081594	chr8	ENSRNOG00000022845	Cep70	107508351	107547643	+	-Inf	0.0004	0.0269	0	0	0	0	0.1304	0.0980	0	0
ENSRNOT00000081599	chr7	ENSRNOG00000005362	Rab3ip	59928867	59957228	-	-5.5050	0.0000	0.0013	0.0146	0	0.0100	0.0317	0.0751	2.3694	0.0373	0.0710
ENSRNOT00000081709	chr10	ENSRNOG00000030170	Krt10	87296452	87300736	-	-3.9222	0.0001	0.0073	0.0167	0.0476	0.0431	0.0418	0.9852	0	0.0250	1.2506
ENSRNOT00000081854	chr4	ENSRNOG00000061895	Ly49si1	163736132	163762434	-	-3.7851	0.0005	0.0312	0.0427	0.0113	0.0131	0	0	0.5443	0.3479	0.0344
ENSRNOT00000081939	chr4	ENSRNOG00000013135	Ptpn12	10631073	10688211	+	-3.3564	0.0002	0.0161	0.0627	0.3015	0.0222	0.3073	0.0585	0.0596	0.2265	6.7604
ENSRNOT00000082082	chr10	ENSRNOG00000003326	Rrn3	3179346	3214647	+	-2.9276	0.0000	0.0029	0.6940	0.3336	0	0.5018	3.5939	0.5792	4.4004	3.0633
ENSRNOT00000082136	chr20	ENSRNOG00000032708	RT1-Bb	4039413	4047093	+	-Inf	0.0000	0.0021	0	0	0	0	0.2011	0	3.1111	0
ENSRNOT00000082304	chr15	ENSRNOG00000010645	Lgals3	24141651	24165535	+	-7.7466	0.0006	0.0338	0	0	0	0.0062	0	0	1.3270	0
ENSRNOT00000082561	chr3	ENSRNOG00000010897	Nek6	22829817	22888598	+	-4.9051	0.0002	0.0158	0.0046	0.0054	0	0.0054	0.0049	0.4393	0.0091	0.0061
ENSRNOT00000082717	chr5	ENSRNOG00000007590	Eya1	4393472	4513142	+	-3.0512	0.0010	0.0472	0.0018	0	0.0032	0.0319	0.0607	0.1391	0.0803	0.0252
ENSRNOT00000083028	chr5	ENSRNOG00000011054	Laptm5	149056078	149068932	+	-3.6164	0.0002	0.0182	0.0056	0.0402	0	0	0.0160	0.0768	0.0702	0.3984
ENSRNOT00000083245	chr9	ENSRNOG00000049330	NEWGENE_1308196	44483875	44495676	+	-Inf	0.0000	0.0017	0	0	0	0	3.4131	0	0	0
ENSRNOT00000083585	chr1	ENSRNOG00000060849	LOC102552640	222519615	222524779	+	-Inf	0.0000	0.0000	0	0	0	0	2.9522	3.5622	0.2356	3.6771
ENSRNOT00000083701	chr14	ENSRNOG00000003098	Prom1	71542059	71637417	+	-5.6454	0.0000	0.0024	0.0078	0.0403	0.0021	0.0018	0.0151	0.0093	2.5737	0.0012
ENSRNOT00000083732	chr6	ENSRNOG00000004827	Papola	129609375	129660107	+	-1.9262	0.0008	0.0417	0.0901	0.0967	0.0743	0.0842	0.6221	0.4307	0.0734	0.1859
ENSRNOT00000083942	chr1	ENSRNOG00000017703	Unc93b1	219144205	219183963	+	-0.9846	0.0004	0.0274	5.6111	7.0561	2.9860	3.6719	6.4106	14.5768	10.9506	6.3019
ENSRNOT00000083959	chr7	ENSRNOG00000052289	Ptdss1	71293388	71355417	+	-5.3234	0.0006	0.0353	0	0.0107	0	0	0.0177	0.4125	0	0

ENSRNOT00000084567	chr3	ENSRNOG00000018666	Gpsm1	3788583	3794359	+	-1.3502	0.0005	0.0314	1.7425	2.8614	1.5939	0.5031	4.8848	4.9781	5.5111	1.7095
ENSRNOT00000084611	chr6	ENSRNOG00000004657	Sec23a	80062703	80102635	-	-1.1260	0.0009	0.0439	0.7920	1.0080	0.9804	1.1885	0.9581	2.5131	3.4698	1.7212
ENSRNOT00000084633	chr14	ENSRNOG00000014098	Pold2	86111294	86116363	-	-2.3186	0.0008	0.0432	0.1053	0.0025	0.1409	0.1604	0.7913	0.0878	0.0843	1.0773
ENSRNOT00000084756	chr2	ENSRNOG00000011076	Ank2	231232925	231521052	-	-5.4913	0.0000	0.0010	0.0097	0.0051	0.0038	0	0.0088	0.7089	0.1083	0.0099
ENSRNOT00000084796	chr4	ENSRNOG00000060241	LOC497796	164184870	164211819	-	-2.9659	0.0005	0.0325	0.0413	0.0514	0	0.0338	0.0316	0.6508	0.2567	0.0489
ENSRNOT00000084902	chr18	ENSRNOG00000016740	Fam210a	64045432	64084556	-	-2.7480	0.0000	0.0001	0.8053	0.6158	0.6497	0.5838	8.6063	0.6385	0.7079	7.8809
ENSRNOT00000085110	chrX	ENSRNOG00000006967	Xiap	128416809	128452969	+	-0.9591	0.0004	0.0295	19.5593	6.5817	9.7442	9.1350	25.0744	26.9688	9.4512	26.0259
ENSRNOT00000085262	chr10	ENSRNOG00000057058	Cd300a	103438303	103450914	+	-3.5070	0.0000	0.0007	0.3716	0.1937	0.1099	0.1359	0.3899	4.9643	3.3737	0.4936
ENSRNOT00000085581	chr17	ENSRNOG00000014524	S1pr3	13799384	13812704	-	-5.7546	0.0000	0.0000	0.0093	0.0071	0.0065	0.0105	0.4495	0.6846	0.2698	0.3943
ENSRNOT00000085613	chr6	ENSRNOG00000010800	Hadhb	27555689	27582985	-	-8.7593	0.0000	0.0002	0.0019	0.0088	0.0049	0.0047	0.0179	0.0044	0.0029	8.7647
ENSRNOT00000085632	chr2	ENSRNOG00000042717	Ciart	197987742	197991574	-	-4.9907	0.0004	0.0280	0.0208	0	0.0228	0	0.0123	0	1.3720	0
ENSRNOT00000085680	chr7	ENSRNOG00000004517	Igf1	28414350	28486609	+	-6.2934	0.0000	0.0007	0.0021	0	0.0037	0.0036	0.0530	0.3470	0.2618	0.0786
ENSRNOT00000085797	chr6	ENSRNOG00000006865	Laptm4a	34028936	34059961	+	-5.1758	0.0006	0.0343	0	0.0049	0.0037	0.0032	0.0040	0.0326	0.0044	0.3849
ENSRNOT00000085857	chr2	ENSRNOG00000029304	Aggf1	25072877	25095106	-	-2.7580	0.0002	0.0195	0	0.0781	0.0297	0.0043	0.0586	0.3068	0.1169	0.2756
ENSRNOT00000086027	chr20	ENSRNOG00000000443	LOC103689965	4302347	4508197	-	-Inf	0.0000	0.0000	0	0	0	0	0	0	2.8177	1.9565
ENSRNOT00000086161	chr8	ENSRNOG00000011348	Snx14	96023445	96088364	-	-5.2845	0.0006	0.0364	0.0116	0	0.0106	0	0	0.0124	0.8533	0
ENSRNOT00000086242	chr3	ENSRNOG00000015285	Lrp4	80362865	80412815	+	-6.5992	0.0001	0.0077	0.0033	0.0012	0.0012	0	0	0.0027	0.5447	0.0022
ENSRNOT00000086261	chr10	ENSRNOG00000042912	Mycbpap	82232107	82252963	-	-2.9842	0.0004	0.0287	0.0500	0.0163	0.0502	0.0535	0.0149	1.2669	0.0492	0.0140
ENSRNOT00000086305	chr10	ENSRNOG00000004146	Coro7	11090314	11143683	+	-1.7140	0.0008	0.0413	8.3609	0.7173	0.8255	0.5587	11.3660	13.6198	1.4012	7.9372
ENSRNOT00000086355	chr7	ENSRNOG00000019383	Tef	123043584	123055783	+	-Inf	0.0000	0.0000	0	0	0	0	1.0914	0.7040	0.6615	0
ENSRNOT00000086390	chr4	ENSRNOG00000056219	Olr1	163239849	163261958	-	-1.1742	0.0010	0.0478	1.3331	2.2154	0.6152	1.1707	1.5684	4.0233	5.1021	1.3437
ENSRNOT00000086397	chr12	ENSRNOG00000059456	Grk3	49665282	49746162	+	-Inf	0.0000	0.0000	0	0	0	0	10.9430	8.8764	10.1291	0
ENSRNOT00000086525	chr4	ENSRNOG00000059227	Akap3	159403501	159425511	+	-Inf	0.0007	0.0371	0	0	0	0	0	0	0.2583	0
ENSRNOT00000086967	chr9	ENSRNOG00000011000	Rims1	28442813	28972835	-	-3.3923	0.0000	0.0051	1.1039	0.8248	1.2399	0.6475	38.6875	0.0683	0.4010	0.9126
ENSRNOT00000087025	chr13	ENSRNOG00000060544	Kdm5b	51384389	51455357	+	-Inf	0.0000	0.0016	0	0	0	0	0	0	0	1.2564
ENSRNOT00000087038	chr7	ENSRNOG00000051563	Giot1	13104481	13108630	-	-Inf	0.0006	0.0350	0	0	0	0	0	0.7182	0	0
ENSRNOT00000087122	chr3	ENSRNOG00000024808	Stk39	54360420	54593306	-	-3.2513	0.0009	0.0442	0.0229	0.0201	0	0.0146	0.0420	0.0062	0.4849	0.0154
ENSRNOT00000087376	chr7	ENSRNOG00000006235	Nell2	136527135	136853154	-	-Inf	0.0002	0.0152	0	0	0	0	0	0	0.6058	0

ENSRNOT00000087792	chr1	ENSRNOG00000019687	Art2b	166667169	166670867	-	-Inf	0.0008	0.0412	0	0	0	0	0	0.0777	0.0247	0.0260
ENSRNOT00000088065	chr1	ENSRNOG00000060994	Tctn3	259682249	259691742	-	-Inf	0.0000	0.0000	0	0	0	0	3.1559	2.9165	3.9004	0
ENSRNOT00000088078	chr14	ENSRNOG00000002054	Glmn	3204390	3247695	+	-5.1396	0.0001	0.0107	0.5117	0	0	0	0	7.9629	9.6322	0.4414
ENSRNOT00000088304	chr8	ENSRNOG00000013610	Chrna5	59561817	59590172	+	-Inf	0.0000	0.0000	0	0	0	0	1.3338	2.7860	0	0
ENSRNOT00000088327	chr2	ENSRNOG00000020373	Dap3	188226110	188252551	-	-3.4693	0.0001	0.0087	0.0221	0.0132	0.0509	0.0014	0.5092	0.0804	0.0299	0.3503
ENSRNOT00000088603	chr12	ENSRNOG00000000989	Bud31	11232964	11239521	-	-2.7022	0.0000	0.0068	0.0704	0.1004	0.1403	0.1557	1.0739	1.2592	0.6007	0.1032
ENSRNOT00000088674	chr14	ENSRNOG00000002855	Rfc1	44580216	44689001	+	-Inf	0.0004	0.0259	0	0	0	0	0	0	0.4190	0
ENSRNOT00000088969	chr6	ENSRNOG00000005410	Cdca7l	145770662	145784403	+	-Inf	0.0010	0.0470	0	0	0	0	0	0.4906	0	0
ENSRNOT00000088981	chr15	ENSRNOG00000051706	Tep1	27751180	27798408	-	-5.0081	0.0002	0.0151	0	0.0060	0	0.0047	0.0017	0	0.0027	0.3411
ENSRNOT00000089196	chr1	ENSRNOG00000015036	Ctgf	21851660	21854763	-	-2.0562	0.0004	0.0257	1.1257	0.9098	1.1270	0.1403	1.5199	10.9739	0.7815	0.4608
ENSRNOT00000089290	chr7	ENSRNOG00000048982	LOC100912282	144307509	144322240	-	-5.9913	0.0007	0.0368	0.1430	0	0	0	9.0987	0	0	0
ENSRNOT00000089339	chr2	ENSRNOG00000016550	Dclk2	186117778	186245163	-	-Inf	0.0000	0.0000	0	0	0	0	1.6158	0	1.1295	1.2899
ENSRNOT00000089575	chr8	ENSRNOG00000030654	Man2c1	61805677	61816800	+	-2.2092	0.0004	0.0259	0.0537	0.0225	0.0231	0.0408	0.1864	0.0524	0.0831	0.3264
ENSRNOT00000089714	chr2	ENSRNOG00000057089	LOC103691744	264746485	264765085	-	-Inf	0.0004	0.0281	0	0	0	0	0	0.5532	0	0
ENSRNOT00000089891	chr6	ENSRNOG00000004826	Sos2	91883425	92007957	-	-1.9964	0.0001	0.0142	0.9483	0.3230	0.3053	0.3483	4.9459	0.6964	0.3175	1.7206
ENSRNOT00000090047	chr8	ENSRNOG00000055631	Zfp280d	78872102	78949447	+	-1.0930	0.0001	0.0068	7.0844	6.3386	5.7179	4.6237	17.9552	18.7701	7.0086	6.9585
ENSRNOT00000090125	chr20	ENSRNOG00000030630	Ehmt2	4576514	4592973	+	-1.6710	0.0000	0.0028	2.4585	1.8028	4.0227	2.2194	20.6077	4.3920	3.9337	4.5122
ENSRNOT00000090897	chr1	ENSRNOG00000017120	Abhd2	140998260	141087084	+	-1.4275	0.0003	0.0222	1.9366	0.7274	1.7860	0.6433	2.6221	3.5953	4.2067	3.2762
ENSRNOT00000090942	chrX	ENSRNOG00000006642	Yipf6	68626185	68637416	+	-2.6321	0.0000	0.0000	1.2386	0.6110	1.1196	0.8390	9.0668	2.8245	2.1682	9.5486
ENSRNOT00000091242	chr10	ENSRNOG00000047933	LOC103690164	37032056	37051519	+	-Inf	0.0004	0.0258	0	0	0	0	0.6333	0	0	0
ENSRNOT00000091359	chr4	ENSRNOG00000008369	Gimap4	78320241	78327026	+	-Inf	0.0001	0.0075	0	0	0	0	0	0.4606	0.2176	0
ENSRNOT00000091389	chr13	ENSRNOG00000003510	Fmo2	80755107	80775230	-	-Inf	0.0000	0.0004	0	0	0	0	0	0	1.0105	0.9348
ENSRNOT00000091493	chr3	ENSRNOG00000021243	Siglec1	123612257	123631109	-	-2.3146	0.0006	0.0361	2.6128	0.6439	0.0548	0.1183	2.2062	12.2846	2.4987	0.0727
ENSRNOT00000091552	chr4	ENSRNOG00000054251	Clec7a	163216163	163227242	-	-1.5777	0.0007	0.0383	1.4884	2.3837	0.4798	0.5371	1.1641	7.1742	5.3918	0.8633
ENSRNOT00000091565	chr9	ENSRNOG00000042581	Ptcra	16530074	16539472	+	-Inf	0.0008	0.0413	0	0	0	0	0.0401	0.0392	0.0375	0
ENSRNOT00000091680	chr1	ENSRNOG00000018708	Ppp1ca	219440192	219443564	+	-2.4146	0.0002	0.0207	0.0406	0.0418	4.9354	2.4513	9.3252	13.9320	11.9812	4.5845
ENSRNOT00000091890	chr4	ENSRNOG00000061739	Klrk1	163393435	163402561	-	-4.3040	0.0001	0.0137	0	0.0765	0	0	0.0527	1.0864	0.3471	0.0252
ENSRNOT00000092365	chr2	ENSRNOG00000018285	Kcna2	209838869	209843588	+	-6.1560	0.0004	0.0270	0	0	0.0138	0	0	0	0	0.9814

ENSRNOT00000092976	chr1	ENSRNOG00000017030	Arid1b	45923222	46230523	+	-Inf	0.0000	0.0047	0	0	0	0	1.5966	0	0	0
ENSRNOT00000093107	chr8	ENSRNOG00000013610	Chrna5	59561721	59589144	+	-5.1327	0.0001	0.0146	0.0099	0.0016	0	0.0340	1.5739	0	0.0100	0.0116
ENSRNOT00000093260	chr15	ENSRNOG00000010479	Mycbp2	87506959	87540378	-	-4.8211	0.0000	0.0023	0.1207	0.1583	0.1163	0.1299	14.4482	0.1914	0.0805	0.1281
ENSRNOT00000093630	chr9	ENSRNOG00000060984	Dnah7	60271917	60330125	-	-Inf	0.0000	0.0020	0	0	0	0	0.2900	0	0.3045	0
ENSRNOT00000000011	chr19	ENSRNOG00000000010	Cbln1	20607507	20611316	+	1.0962	0.0007	0.0385	5.2058	9.7883	11.2930	6.1193	8.4758	2.0621	3.3612	1.2585
ENSRNOT00000001169	chrX	ENSRNOG00000000875	Fhl1	159158194	159172518	+	0.9036	0.0010	0.0485	4.9121	3.4894	5.7232	9.1206	4.2481	2.1891	3.5003	2.4885
ENSRNOT00000001449	chr12	ENSRNOG00000001092	Kl	943006	987551	+	1.4825	0.0010	0.0488	0.8279	17.5258	12.6297	13.4104	2.8533	5.7377	6.9863	0.3094
ENSRNOT00000001686	chr12	ENSRNOG00000001251	Grifin	16170162	16172129	+	2.6771	0.0004	0.0269	0.0603	0.7654	2.8465	1.1942	0.2086	0.4171	0.1352	0
ENSRNOT00000002452	chr11	ENSRNOG00000001796	Dgkg	81972219	82163165	+	0.8372	0.0001	0.0084	16.9868	17.4138	27.9840	35.1857	15.7577	11.0001	17.2199	10.6352
ENSRNOT00000002492	chr11	ENSRNOG00000001821	Adipoq	81330845	81344488	-	3.1746	0.0008	0.0413	0	0.8206	0.3834	0.7208	0	0	0.1859	0.0273
ENSRNOT00000002498	chr11	ENSRNOG00000001825	AABR07034767.1	88912599	88972176	-	1.2986	0.0006	0.0343	0.7027	1.5342	2.2038	3.4871	0.9380	0.6452	0.9925	0.6471
ENSRNOT00000002640	chr11	ENSRNOG00000001926	Cldn1	77815181	77830416	+	1.5529	0.0006	0.0352	0.1777	1.3494	1.5003	1.6357	0.4351	0.3649	0.6161	0.1732
ENSRNOT00000003050	chr14	ENSRNOG00000002227	Kit	35072133	35149608	-	0.7907	0.0001	0.0145	9.1478	8.8572	15.2875	21.6327	8.7545	7.3445	7.7821	7.8680
ENSRNOT00000003314	chr13	ENSRNOG00000002417	Serpinb10	27465930	27483799	+	Inf	0.0004	0.0267	0.0228	0	0.0476	0.0821	0	0	0	0
ENSRNOT00000003441	chr13	ENSRNOG00000002549	Htr5b	37479758	37492680	-	2.8533	0.0000	0.0050	0.1404	0.9030	0.5676	2.2245	0.1587	0.0131	0.3049	0.0541
ENSRNOT00000003784	chr10	ENSRNOG00000002831	Wfikkn2	81936444	81942188	-	1.3077	0.0002	0.0176	0.9786	2.7607	3.1501	3.1785	1.0705	1.4436	1.0022	0.5508
ENSRNOT00000003859	chr10	ENSRNOG00000024730	Ppm1e	74548270	74679858	-	0.4841	0.0008	0.0426	43.4526	61.9604	51.3019	70.8931	38.4115	37.7835	45.2914	41.2357
ENSRNOT00000004070	chr6	ENSRNOG00000003020	Slc25a47	132762280	132768379	+	2.9693	0.0005	0.0327	0.0857	0.0608	0.0757	0.0617	0	0	0.0362	0
ENSRNOT00000004385	chr14	ENSRNOG00000003253	Qdpr	70164650	70178284	+	0.5107	0.0003	0.0228	111.0335	162.0227	149.6088	138.0499	96.2499	102.1614	103.6729	91.4623
ENSRNOT00000004400	chr10	ENSRNOG00000003284	Epn3	82220762	82229140	-	1.1020	0.0008	0.0418	0.8639	2.5047	1.7812	1.9262	0.6736	1.1057	0.7893	0.7280
ENSRNOT00000004780	chr13	ENSRNOG00000003515	Ephx1	99271390	99287887	-	6.4587	0.0001	0.0082	0.0220	0.0164	1.0414	0	0	0	0	0.0123
ENSRNOT00000004905	chr13	ENSRNOG00000003694	Prox1	108382399	108443294	+	1.1548	0.0000	0.0017	4.6777	15.0988	6.7098	13.9741	4.8296	5.1051	4.6954	3.5418
ENSRNOT00000005031	chr10	ENSRNOG00000003773	Hmox2	10990034	11005952	-	3.6889	0.0002	0.0163	1.3926	0.0303	0.0122	0.0186	0.0474	0.0276	0.0118	0.0259
ENSRNOT00000005176	chr10	ENSRNOG00000003845	Wnt3	91830654	91874793	+	1.6671	0.0003	0.0254	0.2350	2.2728	0.9397	1.4344	0.3752	0.5851	0.3923	0.1846
ENSRNOT00000005866	chr3	ENSRNOG00000004372	Cbln4	170033166	170040953	-	0.9775	0.0002	0.0156	3.7099	5.5567	7.6174	6.9516	3.5530	2.2845	3.6922	2.5757
ENSRNOT00000006070	chr7	ENSRNOG00000004554	Dcn	38742051	38782323	+	0.7932	0.0010	0.0491	9.5449	5.8725	11.8402	12.2804	4.5997	6.3038	8.0487	3.8644
ENSRNOT00000006218	chr10	ENSRNOG00000004093	Rhot1	67559420	67640555	+	Inf	0.0000	0.0000	5.3838	1.7743	0	0	0	0	0	0
ENSRNOT00000006264	chr15	ENSRNOG00000004516	Itgbl1	110114148	110378969	+	1.1864	0.0003	0.0222	2.4219	2.0592	3.7912	5.9365	2.0140	1.4331	1.3458	1.4505

ENSRNOT00000006339	chr6	ENSRNOG00000019584	Dlk1	133552821	133583498	+	7.2799	0.0001	0.0115	0	0	2.3365	0	0.0150	0	0	0
ENSRNOT00000006689	chr3	ENSRNOG00000005037	Kif18a	100366168	100426687	+	Inf	0.0001	0.0120	0	0.1003	0.1200	0	0	0	0	0
ENSRNOT00000006789	chr7	ENSRNOG00000004890	Adcy8	105353913	105592804	-	0.8048	0.0002	0.0178	5.6924	6.7951	8.1607	8.0050	5.8729	3.9260	3.9856	2.6177
ENSRNOT00000006862	chr13	ENSRNOG00000005166	Nhlh1	90437956	90443157	-	5.4422	0.0000	0.0001	0.0422	0.6218	0.1250	1.6208	0.0169	0	0	0.0385
ENSRNOT00000007103	chr3	ENSRNOG00000005258	Myef2	117354581	117389456	-	2.5560	0.0005	0.0300	2.0070	3.1181	30.3730	0	2.1044	2.2545	0.8386	0.8390
ENSRNOT00000007309	chr7	ENSRNOG00000005491	Best3	60015998	60057152	+	1.9433	0.0006	0.0350	0.0818	0.6454	0.3458	0.4245	0.0280	0.1286	0.1928	0.0400
ENSRNOT00000007662	chr3	ENSRNOG00000005609	Neurod1	66414308	66417741	-	0.8379	0.0008	0.0434	4.8813	5.3838	6.7173	8.6736	4.1849	3.3266	3.7071	3.1348
ENSRNOT00000008387	chr3	ENSRNOG00000006397	Chrm5	103966451	104018861	-	1.3881	0.0009	0.0437	1.2784	1.1120	3.2688	2.5329	1.5442	0.5383	0.8776	0.1698
ENSRNOT00000008608	chr20	ENSRNOG00000006589	Mif	13732198	13732859	+	0.7335	0.0004	0.0261	107.8171	93.9312	224.3013	133.0977	88.9064	85.8093	93.7522	67.8244
ENSRNOT00000008679	chr10	ENSRNOG00000006612	Map2k3	47182433	47203753	+	Inf	0.0000	0.0000	1.5356	1.3432	1.5453	0	0	0	0	0
ENSRNOT00000008752	chr15	ENSRNOG00000006649	Thrb	8890578	8914501	-	4.4097	0.0000	0.0002	14.9167	0.2093	0.2036	0.2721	0.2251	0.1807	0.0591	0.2691
ENSRNOT00000008759	chr15	ENSRNOG00000005957	Slc4a7	11836407	11912916	-	Inf	0.0000	0.0006	0	0.3435	0	0.4259	0	0	0	0
ENSRNOT00000008941	chr7	ENSRNOG00000006789	Ddit3	70580252	70585074	+	2.4945	0.0006	0.0352	1.3825	1.6526	2.3438	1.2613	0.7137	0.4646	0	0
ENSRNOT00000008972	chr4	ENSRNOG00000006197	Calu	56625611	56653111	+	8.5328	0.0000	0.0001	0.0047	21.8154	0.0132	0.0052	0.0047	0.0102	0.0180	0.0260
ENSRNOT00000009101	chr14	ENSRNOG00000006876	Msx1	77712240	77716059	+	1.1504	0.0006	0.0346	2.0465	6.3819	4.2181	6.8213	2.1133	2.9426	2.6389	1.0757
ENSRNOT00000009428	chr7	ENSRNOG00000007003	Mei1	123308041	123361391	+	1.6304	0.0002	0.0154	0.6397	1.1234	0.6485	3.2313	0.4750	0.3599	0.2810	0.7068
ENSRNOT00000009879	chr5	ENSRNOG00000007404	Svbp	138245822	138251353	+	4.8879	0.0001	0.0147	0	0	1.2043	1.0325	0.0096	0.0405	0.0254	0
ENSRNOT00000009940	chr6	ENSRNOG00000009284	Foxa1	78545804	78549669	-	Inf	0.0000	0.0009	0	0.0508	0.4647	0.2063	0	0	0	0
ENSRNOT00000010358	chr10	ENSRNOG00000007869	Wscd1	58342393	58371196	+	0.8931	0.0002	0.0154	36.7522	59.0731	18.0525	18.8360	19.2808	21.6360	17.8297	12.7137
ENSRNOT00000010366	chr6	ENSRNOG00000007172	Map4k3	3276738	3444519	-	0.5738	0.0011	0.0498	21.3372	11.7655	14.2304	23.6874	11.3343	13.7543	11.0808	11.5451
ENSRNOT00000010454	chr5	ENSRNOG00000007886	Orm1	79179417	79182820	+	Inf	0.0000	0.0043	0	0.2191	0.0375	0.3057	0	0	0	0
ENSRNOT00000010846	chr5	ENSRNOG00000008184	Esrp1	24576991	24631679	-	3.4913	0.0000	0.0046	0.0929	0.0214	0.0390	0.0727	0	0	0.0100	0.0101
ENSRNOT00000011604	chr14	ENSRNOG00000008716	Nefh	85181572	85191557	-	0.5253	0.0006	0.0356	33.6444	38.4201	46.9174	38.6106	20.5301	31.2507	24.3346	33.3838
ENSRNOT00000011651	chr4	ENSRNOG00000008628	Ica1	34635506	34780193	-	1.1193	0.0000	0.0045	10.5454	6.4399	13.3563	17.1772	6.7274	4.1372	5.2103	5.7986
ENSRNOT00000011711	chr15	ENSRNOG00000008855	Gjb2	37377316	37383277	-	0.8937	0.0008	0.0421	4.9901	3.4176	3.4540	4.2320	2.2329	2.4669	2.4081	1.5540
ENSRNOT00000011963	chr8	ENSRNOG00000047799	Gnb5	82257849	82285184	+	Inf	0.0000	0.0005	0	18.8375	0	0	0	0	0	0
ENSRNOT00000011983	chr1	ENSRNOG00000008890	Slc18a2	280423079	280457148	+	1.9461	0.0003	0.0221	0.7601	1.2236	8.6320	0.5734	1.0809	0.7313	0.9579	0.1337
ENSRNOT00000012162	chr8	ENSRNOG00000009005	Slco2a1	111495443	111576697	+	Inf	0.0001	0.0148	0.4817	0	0	0	0	0	0	0

ENSRNOT00000012385	chr5	ENSRNOG00000009269	Cga	50381244	50393367	+	7.4436	0.0001	0.0071	10.0759	0	0	0	0	0	0.0579	0
ENSRNOT00000012727	chr3	ENSRNOG00000009588	Snph	147103562	147143576	-	3.1121	0.0004	0.0259	0.2007	0.0633	0.1209	0.2019	0	0	0	0.0679
ENSRNOT00000013355	chr4	ENSRNOG00000009956	Wnk1	152452848	152578403	-	0.7178	0.0002	0.0199	9.7229	10.3624	8.2370	5.6851	5.8193	7.2721	3.9953	3.5912
ENSRNOT00000013694	chr2	ENSRNOG00000010121	Lef1	236233239	236345056	+	0.8452	0.0010	0.0485	3.2129	9.9032	4.9816	7.6982	3.4148	4.0759	3.9190	2.9488
ENSRNOT00000013991	chr16	ENSRNOG00000010392	Nrg1	63958645	64057434	+	3.1793	0.0000	0.0018	0.2393	0.7927	0.0849	0.1249	0.0532	0.0539	0.0168	0.0132
ENSRNOT00000014034	chr2	ENSRNOG00000010325	Ptger3	263895290	263975834	+	1.4215	0.0005	0.0312	1.6376	2.7952	1.7629	2.5206	0.8749	0.4845	1.1981	0.6966
ENSRNOT00000014129	chr3	ENSRNOG00000010528	LOC100911166	112519808	112525575	+	6.3505	0.0000	0.0000	0	1.5917	2.0975	1.6689	0	0.0438	0	0.0218
ENSRNOT00000014152	chr1	ENSRNOG00000010308	Nr2f2	131448737	131454689	-	1.3151	0.0000	0.0047	16.3997	16.3424	24.7811	18.8560	13.1476	4.7979	10.3927	2.3594
ENSRNOT00000014218	chr13	ENSRNOG00000010357	Lhx9	55861015	55878094	-	2.8591	0.0000	0.0018	0.5512	4.0851	1.6838	2.5793	0.6893	0.2246	0.2829	0.0297
ENSRNOT00000014354	chr5	ENSRNOG00000010514	Cachd1	119727839	119831634	+	0.8710	0.0000	0.0066	7.6749	9.3761	12.7910	10.6592	6.5410	5.4596	5.9580	4.1856
ENSRNOT00000014536	chr5	ENSRNOG00000010893	Hes3	169464989	169467153	-	2.9786	0.0003	0.0236	0.0566	0.1860	0.1588	0.3570	0	0.0647	0.0316	0
ENSRNOT00000014731	chr6	ENSRNOG00000011091	Ferd3l	53371706	53372584	+	Inf	0.0009	0.0448	0	0.1789	0.0218	0.0434	0	0	0	0
ENSRNOT00000014747	chr15	ENSRNOG00000010997	Ednrb	88006977	88036354	-	0.7941	0.0001	0.0139	29.6351	45.0253	36.2036	30.3201	29.1453	20.2580	21.5455	10.4714
ENSRNOT00000014867	chr10	ENSRNOG00000010929	Tcam1	94466523	94478594	+	2.1514	0.0002	0.0168	0.0885	0.1300	0.1914	0.3493	0.0303	0.0444	0.0423	0.0539
ENSRNOT00000015015	chr8	ENSRNOG00000011241	Tfdp2	104106740	104176446	+	4.3042	0.0000	0.0009	5.6659	7.1240	0.0207	6.8316	0.0300	0.0211	0.9044	0.0388
ENSRNOT00000015272	chr19	ENSRNOG00000011180	Irx5	16417817	16420511	+	2.8474	0.0008	0.0433	0.2091	0.7291	0.7502	0.2179	0.2649	0	0	0
ENSRNOT00000015576	chr5	ENSRNOG00000011504	Akap2	74941802	74982401	+	0.5523	0.0004	0.0274	26.0164	39.0142	29.2292	35.7611	24.3775	21.7549	25.6954	16.8405
ENSRNOT00000015583	chr19	ENSRNOG00000011533	Irx3	15838714	15840990	-	2.9646	0.0000	0.0016	0.2878	0.7734	1.0492	0.4631	0.1190	0.0409	0.1439	0.0259
ENSRNOT00000015692	chr4	ENSRNOG00000011648	Aqp1	85551503	85563683	+	1.4136	0.0003	0.0235	1.2707	7.8024	3.8037	5.0932	1.6794	2.1403	2.5250	0.4007
ENSRNOT00000016048	chr1	ENSRNOG00000012057	Olig3	14797766	14799835	+	2.8206	0.0008	0.0411	0.3419	0.0901	0.1913	0.2065	0.1175	0	0	0
ENSRNOT00000016056	chr8	ENSRNOG00000011936	Abhd14a	115141580	115149419	-	3.8867	0.0000	0.0015	1.7298	0.0936	0.0839	1.6252	0.0313	0.0214	0.1464	0.0397
ENSRNOT00000016175	chr4	ENSRNOG00000012098	Adcyap1r1	85662892	85711696	+	4.2632	0.0000	0.0035	0.0088	0.0400	0.8554	0.0088	0.0206	0.0040	0.0095	0.0135
ENSRNOT00000016452	chr16	ENSRNOG00000012283	Chrna6	68860018	68866718	+	3.1237	0.0005	0.0327	0	0.3249	2.3316	0.1612	0.0967	0.1440	0.0826	0
ENSRNOT00000016485	chr2	ENSRNOG00000012278	Fgf10	51672722	51750369	+	1.4987	0.0005	0.0301	0.4028	1.6123	0.7755	1.2317	0.3431	0.4382	0.5466	0.0955
ENSRNOT00000016554	chr10	ENSRNOG00000012390	Npw	14021187	14022452	-	Inf	0.0003	0.0237	0.0437	0	0.1117	0.2049	0	0	0	0
ENSRNOT00000016656	chr16	ENSRNOG00000012448	Chrnb3	68875709	68913628	-	2.4481	0.0000	0.0008	0.5623	0.9982	2.2050	0.8663	0.3304	0.1022	0.2980	0.1182
ENSRNOT00000016843	chr2	ENSRNOG00000012478	Shox2	164118191	164126783	-	2.0307	0.0007	0.0370	0.3171	14.5795	3.9085	6.3658	0.4785	3.5823	2.0210	0.0784
ENSRNOT00000016976	chr6	ENSRNOG00000012686	Pomc	28382962	28388967	+	1.9470	0.0003	0.0245	12.7140	0.8626	2.9152	0.8950	2.3943	0.6190	0.8469	0.6492

ENSRNOT00000017019	chr1	ENSRNOG00000012742	Irx2	33271977	33275540	-	2.8528	0.0001	0.0089	0.1949	1.8238	1.3074	0.8330	0.3669	0.0840	0.1248	0
ENSRNOT00000017137	chr1	ENSRNOG00000012847	Scgb1c1	213595240	213596459	+	1.7451	0.0010	0.0472	0.5820	3.0916	4.1225	4.6884	0.7054	1.8117	0.9421	0.2651
ENSRNOT00000017317	chr19	ENSRNOG00000012789	Mlnr	55176258	55183557	-	1.1383	0.0000	0.0068	3.1266	6.4463	4.0966	4.7436	1.7715	2.7477	1.9279	1.9178
ENSRNOT00000017472	chr4	ENSRNOG00000012999	Phb2	157230895	157235051	+	3.7752	0.0001	0.0111	3.4444	0.8434	8.2566	0.0031	0.9097	0.0024	0.0010	0.0033
ENSRNOT00000017725	chr3	ENSRNOG00000013209	Barhl1	7491234	7498555	-	3.1418	0.0001	0.0134	0.0280	0.0777	0.6740	0.1346	0.0336	0.0538	0.0161	0
ENSRNOT00000017878	chr2	ENSRNOG00000012892	Abca4	225645568	225783287	+	1.4191	0.0006	0.0339	0.1499	0.9291	0.8781	0.9777	0.2975	0.2872	0.3963	0.1164
ENSRNOT00000018427	chr16	ENSRNOG00000013768	Defb1	75294376	75309176	-	2.3104	0.0004	0.0266	1.1307	0.1368	0.4408	0.9184	0.1818	0.1776	0.1702	0
ENSRNOT00000018435	chr2	ENSRNOG00000013771	Clca2	250954397	250981623	-	Inf	0.0006	0.0339	0	0.0100	0.0092	0.0201	0	0	0	0
ENSRNOT00000018780	chr2	ENSRNOG00000014046	Sertm1	144303703	144323254	-	0.7725	0.0002	0.0158	10.8023	8.5073	14.7248	16.3140	8.2029	6.2960	7.6167	7.3574
ENSRNOT00000018967	chr10	ENSRNOG00000014081	Nmur2	41392666	41408518	-	1.7849	0.0010	0.0480	0.6885	0.3279	0.1567	0.2268	0.1129	0.2107	0.0704	0.0123
ENSRNOT00000019133	chr10	ENSRNOG00000014233	Krt19	88055841	88060561	-	Inf	0.0000	0.0034	0.0986	0.0459	0.0274	0.0436	0	0	0	0
ENSRNOT00000019307	chr8	ENSRNOG00000013829	Chrna3	59592403	59607275	-	1.7836	0.0001	0.0073	0.6951	5.6551	1.0203	2.0369	0.6980	0.7902	0.6643	0.5800
ENSRNOT00000019681	chr1	ENSRNOG00000049232	Tcf7l2	276659542	276730514	+	1.2497	0.0001	0.0070	4.3063	27.6672	13.3982	14.0685	5.5454	8.1359	8.0210	3.2950
ENSRNOT00000019862	chr17	ENSRNOG00000014616	Iars	15356016	15402631	-	5.5919	0.0008	0.0429	0	0	0	3.5889	0.0744	0	0	0
ENSRNOT00000019931	chr2	ENSRNOG00000014867	Synpo2	227255902	227411964	-	1.1198	0.0003	0.0235	2.3964	11.1803	2.8512	6.0519	2.2973	3.5296	3.0342	1.4831
ENSRNOT00000019997	chr2	ENSRNOG00000014686	Kcnd3	207923775	208140727	+	0.7245	0.0002	0.0169	11.3191	13.2275	15.4393	13.4589	12.0013	5.7964	9.3128	5.2347
ENSRNOT00000020100	chr1	ENSRNOG00000014971	Mas1	48077033	48108216	+	1.0258	0.0010	0.0469	2.0812	3.3744	3.2301	5.5293	2.4117	1.5654	1.3618	1.6427
ENSRNOT00000020926	chr3	ENSRNOG00000015550	Ptgds	2686123	2689084	-	1.1120	0.0000	0.0001	555.5302	340.3972	383.8003	544.1069	221.1895	207.2356	279.1810	136.1798
ENSRNOT00000021253	chr18	ENSRNOG00000015867	Chst9	6834365	7081356	-	1.7502	0.0001	0.0144	0.4625	2.3772	1.9580	3.4843	0.7708	0.4977	0.8824	0.3109
ENSRNOT00000021270	chr9	ENSRNOG00000015567	Slc9a2	47386626	47491907	+	1.1419	0.0004	0.0276	2.5015	1.9690	3.6948	4.4099	2.5755	0.6579	1.5897	0.8753
ENSRNOT00000021471	chr1	ENSRNOG00000016011	Plekhg1	40389638	40520447	+	1.0395	0.0000	0.0060	6.3067	24.8290	10.7518	18.9145	6.1556	9.5983	8.0173	5.8095
ENSRNOT00000021502	chr2	ENSRNOG00000015553	Gatad2b	189655702	189732559	+	2.4478	0.0002	0.0175	2.0945	0.1136	0.3332	0.1142	0.0728	0.1762	0.0964	0.1413
ENSRNOT00000021702	chr1	ENSRNOG00000016102	Ebf3	209523157	209641123	-	2.1786	0.0002	0.0153	0.2619	1.1306	2.4705	0.3701	0.5869	0.0636	0.2452	0.0394
ENSRNOT00000021746	chr1	ENSRNOG00000015724	Gucy2g	276187242	276228574	-	3.7437	0.0006	0.0364	0.0202	0	0.0497	0.0839	0	0.0115	0	0
ENSRNOT00000022091	chr19	ENSRNOG00000016422	Klhl36	52515022	52536048	+	Inf	0.0000	0.0042	0	0	0	1.5784	0	0	0	0
ENSRNOT00000022200	chr2	ENSRNOG00000016571	Ngf	204886202	204939523	+	Inf	0.0001	0.0111	0	0	0	1.6122	0	0	0	0
ENSRNOT00000022383	chr1	ENSRNOG00000016147	Slc17a6	106998623	107038704	+	1.5871	0.0000	0.0003	12.5368	42.9184	30.4455	24.1883	15.6763	8.0243	9.7693	3.1722
ENSRNOT00000022943	chr19	ENSRNOG00000016977	Calb2	41482728	41509658	+	1.1625	0.0003	0.0230	15.5557	39.8141	45.6632	19.6419	26.6715	8.4763	15.2677	3.4964

ENSRNOT00000022953	chr3	ENSRNOG00000017019	Lmx1b	12609574	12686869	-	4.8475	0.0000	0.0051	0.0292	0.0484	0.7111	0.1280	0.0318	0	0	0
ENSRNOT00000023068	chr9	ENSRNOG00000016957	Igfbp2	80118029	80144789	+	1.1727	0.0000	0.0015	20.9717	46.5059	31.8349	48.1447	12.9111	19.1861	22.7449	10.5680
ENSRNOT00000023116	chr1	ENSRNOG00000016625	Slc22a2	48317995	48360261	-	2.3735	0.0004	0.0285	0.1925	0.2770	0.0163	0.2602	0.0187	0.0635	0.0436	0.0183
ENSRNOT00000023130	chr19	ENSRNOG00000017084	Hsd11b2	37476096	37481307	+	2.7462	0.0006	0.0351	0.1137	0.0796	0.0339	0.0685	0	0.0441	0	0
ENSRNOT00000023376	chr1	ENSRNOG00000017299	Phrf1	214216047	214248901	+	8.3138	0.0000	0.0000	0.0004	1.8370	1.2332	0.0238	0.0068	0.0009	0.0014	0.0006
ENSRNOT00000023984	chr16	ENSRNOG00000017689	Itih3	6992171	7007051	-	Inf	0.0000	0.0006	0	0	6.0049	0	0	0	0	0
ENSRNOT00000024000	chr1	ENSRNOG00000017619	Aldh1a1	238222521	238264330	+	1.1659	0.0000	0.0056	8.5234	6.1650	19.0026	7.5969	4.3752	3.8003	4.7889	5.4367
ENSRNOT00000024917	chr19	ENSRNOG00000018445	Agt	57321640	57333433	-	0.9356	0.0000	0.0025	75.6267	103.3707	123.8192	86.3650	84.3116	40.8583	48.4217	29.8767
ENSRNOT00000024978	chr15	ENSRNOG00000018399	Cpne6	34187223	34194118	+	0.5550	0.0011	0.0496	38.5531	37.3695	51.6375	70.8678	38.3672	28.9519	40.0850	27.6586
ENSRNOT00000025548	chr19	ENSRNOG00000018884	Ttc13	57429980	57484566	-	1.6280	0.0000	0.0053	7.0565	3.7425	3.4063	1.7228	1.6833	0.6158	0.6287	2.2256
ENSRNOT00000026009	chr1	ENSRNOG00000019161	Cpeb1	143171870	143278485	-	2.0896	0.0003	0.0238	0.7056	0.8720	0.8279	0.8750	0.0573	0.0267	0.6138	0.0730
ENSRNOT00000026187	chr17	ENSRNOG00000019336	Gata3	72429618	72450681	+	4.7541	0.0000	0.0007	0.0261	0.3379	1.2198	0.1909	0	0.0226	0.0432	0
ENSRNOT00000026705	chr2	ENSRNOG00000019638	Lmna	187842885	187863516	-	9.4573	0.0000	0.0002	0.0023	0.0023	5.0657	0.0008	0.0006	0.0031	0.0008	0.0027
ENSRNOT00000026751	chr16	ENSRNOG00000019760	Oxnad1	8207223	8236292	+	3.5256	0.0004	0.0292	0.0332	3.4939	0.1732	0	0.1204	0.1589	0.0420	0
ENSRNOT00000027063	chr1	ENSRNOG00000019959	Kcnc3	100593680	100607874	+	0.5238	0.0006	0.0351	18.2237	15.6611	18.8831	18.6264	11.0895	14.5622	11.0685	12.9361
ENSRNOT00000027552	chr8	ENSRNOG00000020284	Prkar2a	117486083	117545564	+	3.0243	0.0000	0.0053	0.3147	10.7363	0.3539	0.3531	0.3635	0.3500	0.3056	0.4261
ENSRNOT00000027682	chr1	ENSRNOG00000020410	Th	216073031	216080287	-	3.3497	0.0000	0.0009	2.0587	0.3295	22.2194	1.4734	1.4431	0.2978	0.4139	0.4036
ENSRNOT00000028283	chr1	ENSRNOG00000050994	Cttn	215255386	215290838	-	Inf	0.0000	0.0002	0	0	0	15.6949	0	0	0	0
ENSRNOT00000028409	chr8	ENSRNOG00000020915	Setd2	118821729	118888224	+	Inf	0.0000	0.0000	0.8732	0.8568	0	0	0	0	0	0
ENSRNOT00000028508	chr1	ENSRNOG00000020985	Atp4a	89162639	89175811	+	2.4591	0.0009	0.0453	0.0071	0.1228	0.1009	0.1362	0.0378	0.0178	0.0112	0
ENSRNOT00000029577	chr8	ENSRNOG00000027736	Cnn1	23113048	23121761	+	1.9131	0.0009	0.0444	0.7587	0.2477	0.1591	0.6490	0.0823	0.0270	0.2649	0.1076
ENSRNOT00000030329	chr1	ENSRNOG00000025587	Plagl1	7252349	7259035	+	1.3108	0.0002	0.0195	3.6697	1.7283	3.7334	2.8204	2.4195	0.6111	1.4136	0.3734
ENSRNOT00000030912	chr9	ENSRNOG00000011823	Tfap2b	25410669	25440384	+	Inf	0.0001	0.0115	0	0	0.4699	0	0	0	0	0
ENSRNOT00000031243	chr19	ENSRNOG00000021517	Tmem231	44137423	44158621	-	1.7170	0.0002	0.0190	4.0753	0.6919	0.7309	0.7496	0.5687	0.7029	0.4090	0.2198
ENSRNOT00000031789	chr13	ENSRNOG00000028194	Serpinb13	27032048	27062620	+	Inf	0.0002	0.0160	0.0648	0.0761	0	0.0260	0	0	0	0
ENSRNOT00000032250	chr12	ENSRNOG00000022483	Trim50	24348321	24365324	-	Inf	0.0001	0.0072	0.0252	0.0200	0.0371	0.0202	0	0	0	0
ENSRNOT00000032280	chr8	ENSRNOG00000008095	Onecut1	81766041	81793577	+	1.9616	0.0005	0.0314	0.2840	0.2921	0.1829	0.0894	0.1327	0.0173	0.0331	0.0347
ENSRNOT00000032772	chr4	ENSRNOG00000028219	Tmem209	57796338	57823283	-	1.4747	0.0006	0.0345	1.9386	3.7957	3.3629	0.5169	0.5233	1.5077	0.5833	0.8450

ENSRNOT00000033940	chr2	ENSRNOG00000021745	Bhlhe22	102685513	102688624	+	0.9289	0.0000	0.0023	18.6281	13.9357	22.3004	29.5792	12.2184	10.1386	10.2168	11.7819
ENSRNOT00000034166	chr3	ENSRNOG00000024923	Nnat	154043873	154046330	+	0.8860	0.0002	0.0207	108.3518	110.4139	183.8837	163.5499	128.8571	51.7163	99.5671	26.2308
ENSRNOT00000034277	chr8	ENSRNOG00000022084	Sox14	108107865	108109801	-	2.7665	0.0004	0.0290	0.2292	0.1023	0.8953	0.2032	0.1684	0.0418	0	0
ENSRNOT00000034426	chr1	ENSRNOG00000027240	Kndc1	212230259	212277694	+	0.4925	0.0005	0.0332	25.2749	37.5735	33.7556	38.4564	22.6989	26.8901	22.7373	23.6717
ENSRNOT00000034854	chr1	ENSRNOG00000022129	Galp	71352419	71373605	-	Inf	0.0006	0.0359	0.5069	0	0.0963	0	0	0	0	0
ENSRNOT00000034907	chr17	ENSRNOG00000024874	Prtfdc1	87927058	88037040	-	2.3001	0.0002	0.0186	1.4101	22.8586	1.7879	1.5062	1.6684	1.7141	0.1165	2.0977
ENSRNOT00000034960	chr2	ENSRNOG00000024119	Ghsr	113066885	113070261	+	1.9685	0.0003	0.0219	1.0058	0.4184	0.9997	1.2120	0.5652	0.1569	0.1319	0.0750
ENSRNOT00000035285	chr10	ENSRNOG00000022244	Olr1462	45922873	45923829	-	2.4035	0.0009	0.0453	0.0726	0.1561	0.3208	0.9014	0	0.0826	0.1540	0.0376
ENSRNOT00000035567	chr10	ENSRNOG00000027006	LOC100911361	80953006	80954332	+	1.4124	0.0004	0.0287	2.1599	1.3440	4.7135	4.9957	1.4373	1.0599	1.0274	1.4394
ENSRNOT00000036168	chr1	ENSRNOG00000026432	Syt7	226436139	226493179	+	Inf	0.0000	0.0006	0	0.8182	0	0.9933	0	0	0	0
ENSRNOT00000036954	chr10	ENSRNOG00000028075	LOC360479	10795229	10808585	-	3.8153	0.0005	0.0329	0.0787	0.1280	0	0.2030	0.0055	0.0236	0	0
ENSRNOT00000037375	chr8	ENSRNOG00000021548	Rassf1	116307141	116318289	+	2.1329	0.0002	0.0197	0.7473	0.5822	0.4934	0.0808	0.1551	0.0840	0.1229	0.0720
ENSRNOT00000037700	chr1	ENSRNOG00000022919	Chst8	90542096	90685257	-	0.9148	0.0006	0.0352	3.7426	4.8873	6.6292	7.3061	3.4655	2.8532	3.1386	2.5117
ENSRNOT00000038222	chr9	ENSRNOG00000025691	Pla2g7	19935768	19978013	-	0.7467	0.0002	0.0205	19.1662	22.3701	17.3197	23.2763	15.4309	7.7954	13.3413	12.3807
ENSRNOT00000038549	chr16	ENSRNOG00000010392	Nrg1	63837216	64057434	+	2.4066	0.0009	0.0449	0.0738	1.3772	0.0598	0.4397	0	0.1531	0.1451	0.0696
ENSRNOT00000038703	chr2	ENSRNOG00000028335	Fat4	125752130	125879387	+	1.0766	0.0000	0.0008	3.7919	4.7171	5.5543	8.4252	3.8038	2.2008	2.4539	2.2040
ENSRNOT00000038983	chr3	ENSRNOG00000025394	Tanc1	45683993	45917745	+	0.9509	0.0000	0.0040	5.8549	13.7696	9.4648	17.5397	6.2789	6.0767	6.5360	5.2295
ENSRNOT00000039271	chr8	ENSRNOG00000023622	LOC100910021	106863494	106870994	-	2.4576	0.0000	0.0011	2.5326	3.0464	1.1226	3.6823	0.4851	0.4803	0.3964	0.5286
ENSRNOT00000039337	chr6	ENSRNOG00000047365	LOC100360647	86131242	86131643	+	0.8816	0.0005	0.0326	104.2175	41.8105	96.2361	80.0808	34.4432	21.3292	69.0460	50.1386
ENSRNOT00000039677	chr1	ENSRNOG00000020857	Ppfia1	217644259	217720221	-	6.7021	0.0000	0.0013	0	0.4703	0.2052	0.0081	0	0	0	0.0066
ENSRNOT00000040132	chr10	ENSRNOG00000050441	AABR07028989.1	1622573	1674701	+	Inf	0.0000	0.0062	0	0	0	1.2772	0	0	0	0
ENSRNOT00000040215	chr14	ENSRNOG00000030568	Rgs12	80975322	81053906	-	1.5004	0.0009	0.0435	0.2646	2.4108	0.3719	0.5362	0.3839	0.3114	0.3043	0.2670
ENSRNOT00000040249	chr3	ENSRNOG00000049848	Rpl21	91870208	91870690	+	4.4896	0.0000	0.0002	0.4411	0.4703	27.0312	5.7949	0.2015	0.3007	0.6952	0.3044
ENSRNOT00000040291	chr1	ENSRNOG00000017302	Slc6a3	32323011	32363983	-	Inf	0.0000	0.0004	0.0271	0	1.8992	0.0027	0	0	0	0
ENSRNOT00000041241	chrMT	ENSRNOG00000033615	Mt-nd3	9451	9798	+	0.4690	0.0007	0.0390	563.4276	577.7128	739.6663	562.4886	549.3760	443.5878	390.9995	381.2720
ENSRNOT00000041310	chr14	ENSRNOG00000017074	Rab28	73889213	73963202	+	1.2668	0.0001	0.0096	8.2004	8.5501	5.8091	28.4473	6.3630	5.1802	4.8941	4.7607
ENSRNOT00000041569	chr3	ENSRNOG00000010262	Hdc	119057528	119075606	-	Inf	0.0000	0.0020	0	0	2.3599	0	0	0	0	0
ENSRNOT00000041639	chr14	ENSRNOG00000005633	Zbtb49	77322040	77343734	+	Inf	0.0000	0.0000	1.3393	0	1.2969	0.8549	0	0	0	0

ENSRNOT00000041664	chr19	ENSRNOG00000045837	AABR07042611.1	320906	323636	+	Inf	0.0003	0.0220	0.2657	0	0	0.4463	0	0	0	0
ENSRNOT00000041727	chr3	ENSRNOG00000026378	LOC100911204	112531703	112560634	+	Inf	0.0000	0.0015	0	0.2111	0	0.1398	0	0	0	0
ENSRNOT00000042082	chr9	ENSRNOG00000019953	Ube2f	98398128	98428128	+	Inf	0.0004	0.0260	0.3424	0.4866	0	0	0	0	0	0
ENSRNOT00000042316	chr11	ENSRNOG00000028774	Vgll3	3119885	3164751	+	1.8392	0.0002	0.0187	0.9323	0.6491	2.8760	1.9078	0.8437	0.3611	0.2631	0.3109
ENSRNOT00000042721	chr15	ENSRNOG00000048413	AABR07017809.1	31243097	31243530	+	Inf	0.0010	0.0490	0.2039	0	0	0.6759	0	0	0	0
ENSRNOT00000043508	chr6	ENSRNOG00000032348	LOC108349682	135139292	135139621	+	2.2192	0.0001	0.0148	24.2913	1.4402	7.7593	1.8367	2.3614	2.3380	2.1156	0.7724
ENSRNOT00000043544	chr18	ENSRNOG00000027103	Gpr151	36892403	36893797	-	3.0846	0.0007	0.0381	0.0015	3.0811	0.0548	1.0987	0.3507	0.0105	0.1381	0
ENSRNOT00000043693	chrMT	ENSRNOG00000030371	Mt-co2	7006	7689	+	0.4936	0.0007	0.0373	3795.9082	3607.5603	4117.6797	3664.0779	3494.2705	2972.3127	2638.2842	1680.4373
ENSRNOT00000043854	chr1	ENSRNOG00000032798	Slco3a1	135799801	136073483	-	1.4214	0.0001	0.0133	5.8636	6.5046	7.3965	6.8963	6.0547	1.4786	1.7069	0.7135
ENSRNOT00000044232	chr2	ENSRNOG00000010681	Pitx2	233602732	233621051	+	5.8865	0.0001	0.0082	0	0	1.7242	0.2383	0	0	0.0332	0
ENSRNOT00000045647	chr7	ENSRNOG00000029762	Acr	130542203	130548356	+	4.2030	0.0003	0.0253	0.0789	0.0433	0.1161	0	0	0	0	0.0129
ENSRNOT00000045763	chr9	ENSRNOG00000019963	Spats1	17920677	17946797	+	Inf	0.0001	0.0108	0.1702	0	0.0405	0.1987	0	0	0	0
ENSRNOT00000045827	chr3	ENSRNOG00000015396	Sptan1	8534440	8599258	+	0.7245	0.0003	0.0254	28.8262	25.1061	34.2938	28.7725	24.3645	18.7068	21.3549	6.3797
ENSRNOT00000045922	chr5	ENSRNOG00000030155	Bmp8a	141007462	141033511	-	1.7811	0.0007	0.0394	0.0724	0.5863	0.3451	0.5139	0.1381	0.1032	0.1412	0.0591
ENSRNOT00000046013	chr17	ENSRNOG00000017863	Zeb1	54658463	54678710	-	2.9531	0.0001	0.0146	0.2033	0.4892	0.3782	0.5938	0.0003	0	0.1986	0.0160
ENSRNOT00000046854	chr13	ENSRNOG00000003336	Mybph	51034297	51041879	+	Inf	0.0010	0.0479	0	0	0	0.3422	0	0	0	0
ENSRNOT00000047669	chr1	ENSRNOG00000050994	Cttn	215255381	215290845	-	9.2308	0.0000	0.0005	8.5422	0	0.0092	0.0044	0.0142	0	0	0
ENSRNOT00000047755	chr18	ENSRNOG00000030486	Prdm6	48434652	48538023	+	2.4343	0.0001	0.0100	0.9016	0.1561	0.2013	0.6017	0.0489	0.1381	0.0895	0.0677
ENSRNOT00000047805	chr5	ENSRNOG00000007445	Asph	22745751	22769907	-	Inf	0.0000	0.0004	0	1.0049	0	1.2586	0	0	0	0
ENSRNOT00000048353	chr3	ENSRNOG00000008810	Nsmf	2262454	2269902	+	Inf	0.0000	0.0042	0	0	0	2.1418	0	0	0	0
ENSRNOT00000048754	chr1	ENSRNOG00000015473	Phactr2	7355154	7480825	-	1.0730	0.0002	0.0193	4.0011	8.8143	6.9551	6.6808	3.8513	2.9545	4.0547	1.7121
ENSRNOT00000048765	chr7	ENSRNOG00000007286	Mdm1	61174369	61200863	+	3.8269	0.0000	0.0055	2.9035	0.1018	0.0238	0.0115	0.0460	0.0322	0.0658	0.0703
ENSRNOT00000049862	chr11	ENSRNOG00000001747	Pak2	71959228	72017463	+	1.4947	0.0002	0.0189	12.2921	3.0822	1.5024	4.1782	1.8134	2.9349	1.7059	1.0170
ENSRNOT00000051139	chr7	ENSRNOG00000004089	Enpp2	94480396	94563001	-	0.7190	0.0003	0.0210	33.0642	97.2164	76.2922	72.2235	35.4803	51.4194	50.9394	31.5380
ENSRNOT00000051422	chr1	ENSRNOG00000020830	LOC108348128	90952337	90956544	-	Inf	0.0003	0.0240	0	0.2314	0	0.1582	0	0	0	0
ENSRNOT00000051612	chr8	ENSRNOG00000029055	Ttk	91368430	91405993	+	3.7271	0.0006	0.0349	0.1236	0.5195	0	0.4023	0	0.0789	0	0
ENSRNOT00000052018	chr19	ENSRNOG00000034007	Nr3c2	34448289	34752695	-	0.6496	0.0006	0.0353	11.5755	16.4008	14.3598	22.4027	11.2405	9.8456	11.6728	8.5096
ENSRNOT00000054858	chr1	ENSRNOG00000048049	AABR07006049.2	215298161	215310013	-	Inf	0.0000	0.0000	3.5209	1.9226	0	3.5164	0	0	0	0

ENSRNOT00000055073	chr10	ENSRNOG00000003491	Prkca	96191133	96584947	-	Inf	0.0000	0.0000	0.5152	0	0.7584	0.9880	0	0	0	0
ENSRNOT00000055270	chr7	ENSRNOG00000036826	Glycam1	145172493	145174771	-	2.4284	0.0003	0.0237	0.1730	10.1842	3.7674	11.4255	3.0938	0.1680	1.3497	0.1348
ENSRNOT00000056030	chr1	ENSRNOG00000015675	Nell1	105349069	106218958	+	0.5199	0.0008	0.0419	22.5409	21.4962	25.6523	26.7100	19.6678	15.2768	15.6666	16.6217
ENSRNOT00000057055	chr5	ENSRNOG00000010835	Dmbx1	134767436	134776247	-	4.2425	0.0002	0.0190	0	0.0453	0.3025	0.0358	0.0203	0	0	0
ENSRNOT00000057509	chr10	ENSRNOG00000029012	Shisa6	52749846	53037816	-	1.7676	0.0000	0.0011	2.7742	7.5222	5.8816	12.1982	2.1742	2.1227	2.6920	1.3451
ENSRNOT00000058833	chrX	ENSRNOG00000032472	Adgrg2	36930186	37003642	-	Inf	0.0000	0.0002	0.3271	0	0.9529	0	0	0	0	0
ENSRNOT00000058956	chr1	ENSRNOG00000038625	Sbk2	72425707	72433488	+	3.9493	0.0001	0.0090	0.0295	0.0353	0.1380	0.0516	0	0	0.0165	0
ENSRNOT00000059627	chr5	ENSRNOG00000011425	Ptpn3	74250453	74368186	-	0.6949	0.0004	0.0293	10.3996	21.3513	9.1384	16.0644	6.4827	10.9296	8.5807	9.1907
ENSRNOT00000059763	chr14	ENSRNOG00000002216	Srd5a3	34556220	34561696	-	5.3821	0.0000	0.0015	0.0138	0.0427	0.1489	5.8264	0.0426	0.0584	0.0375	0.0061
ENSRNOT00000059892	chr4	ENSRNOG00000011184	Slc13a4	62797677	62840357	-	1.4940	0.0000	0.0015	6.3324	7.6623	5.8435	9.1225	2.5747	3.0753	3.5641	1.0679
ENSRNOT00000060020	chr12	ENSRNOG00000039214	RGD1305455	19577897	19582185	-	Inf	0.0000	0.0001	0.1481	0.8441	0.1856	0	0	0	0	0
ENSRNOT00000060309	chr4	ENSRNOG00000007100	Ccdc136	56674832	56704549	+	0.6421	0.0003	0.0238	10.4207	19.7230	15.8566	16.4192	10.9343	10.1070	9.9509	9.0045
ENSRNOT00000060767	chr1	ENSRNOG00000019549	Akap12	40816107	40906581	+	0.8302	0.0000	0.0024	10.2777	15.9807	15.8739	13.5701	9.4512	7.6468	7.6352	6.5966
ENSRNOT00000061157	chr1	ENSRNOG00000011815	Sgk1	24185456	24191908	-	1.5173	0.0005	0.0308	1.3363	2.4810	5.4762	2.1249	0.5649	0.1910	1.2813	1.9518
ENSRNOT00000063783	chr2	ENSRNOG00000018166	Prkab2	199831990	199844649	+	Inf	0.0002	0.0193	0	0.0368	0	0.2734	0	0	0	0
ENSRNOT00000063889	chr10	ENSRNOG00000013588	Glra1	40855559	40953651	-	1.6510	0.0008	0.0423	0.9784	2.7281	5.5589	1.2438	1.9404	0.4983	0.8252	0.0824
ENSRNOT00000064203	chr3	ENSRNOG00000060572	Tlk1	57013025	57104030	-	5.7668	0.0000	0.0004	0.0267	0.0138	2.8726	9.6064	0.0330	0	0.0197	0.1773
ENSRNOT00000064238	chr2	ENSRNOG00000021656	RGD1359334	196411964	196415530	-	Inf	0.0000	0.0002	0.1436	0.1721	0.0603	0.1220	0	0	0	0
ENSRNOT00000064316	chr16	ENSRNOG00000012757	Nek3	74785281	74806149	+	5.6052	0.0001	0.0146	0.0104	0	0	1.7688	0.0120	0.0080	0.0166	0
ENSRNOT00000064448	chr7	ENSRNOG00000004013	Grip1	64672722	64864155	+	Inf	0.0004	0.0261	0.2715	0	0	0	0	0	0	0
ENSRNOT00000064505	chr10	ENSRNOG00000003039	Wnt3a	45598912	45638035	-	Inf	0.0003	0.0220	0	0.0237	0	0.1727	0	0	0	0
ENSRNOT00000064551	chr8	ENSRNOG00000010652	Dock6	22822412	22874637	-	1.8166	0.0004	0.0276	1.0580	0.6831	0.7213	0.5466	0.0463	0.1143	0.6551	0.0385
ENSRNOT00000064731	chr19	ENSRNOG00000014336	Mcm5	14523570	14560830	+	Inf	0.0000	0.0044	0.2756	0	0.1572	0	0	0	0	0
ENSRNOT00000065234	chr9	ENSRNOG00000015306	Slc9a4	47281961	47328839	+	2.1972	0.0000	0.0005	1.3914	2.1816	4.5075	5.7771	1.3861	0.4284	0.8793	0.3281
ENSRNOT00000065288	chr1	ENSRNOG00000013290	Nrip3	174385424	174411141	-	0.5516	0.0003	0.0234	41.7052	58.0588	54.9779	76.0114	34.6630	38.0569	39.8912	44.8279
ENSRNOT00000065693	chr18	ENSRNOG00000038784	Piezo2	58354648	58499836	-	1.1437	0.0009	0.0466	0.3504	0.5689	0.4551	0.8159	0.2732	0.2318	0.3341	0.1522
ENSRNOT00000065729	chr3	ENSRNOG00000049814	LOC100910882	152226710	152259156	-	1.3350	0.0001	0.0095	29.8581	6.9787	31.7487	24.9555	4.2517	19.8812	6.8689	6.0773
ENSRNOT00000066001	chr5	ENSRNOG00000008922	Trim14	62128941	62153762	-	7.6963	0.0000	0.0000	0.3976	0.3445	0.3182	0	0	0	0	0.0051

ENSRNOT00000066218	chr2	ENSRNOG00000031136	Ntng1	212696624	212852870	-	1.2259	0.0000	0.0009	5.9091	16.5648	10.2651	13.3897	4.7728	6.0522	5.6995	3.1970
ENSRNOT00000066222	chr8	ENSRNOG00000009014	Slc35f2	58347757	58391460	+	Inf	0.0009	0.0464	0	0	0.2394	0	0	0	0	0
ENSRNOT00000066917	chr8	ENSRNOG00000015389	Stag1	108958099	109342731	+	Inf	0.0000	0.0008	0	2.2973	0	0	0	0	0	0
ENSRNOT00000067141	chr19	ENSRNOG00000017905	Map1lc3b	53635688	53643964	+	0.6545	0.0005	0.0335	80.9235	86.9438	88.2648	90.2793	39.6963	46.9107	90.7629	42.7050
ENSRNOT00000067262	chr18	ENSRNOG00000016675	Kcnn2	39335377	39479257	+	3.5050	0.0002	0.0181	0.0119	2.4386	0.0987	0.0124	0.1214	0.0172	0.0719	0.0151
ENSRNOT00000067332	chr12	ENSRNOG00000001079	Daglb	13114003	13157570	-	Inf	0.0001	0.0076	0	0	1.0371	0	0	0	0	0
ENSRNOT00000067389	chr5	ENSRNOG00000006553	Bnc2	102415847	102743417	-	1.4839	0.0009	0.0447	0.4588	0.2561	0.9039	0.3579	0.2428	0.2758	0.0974	0.0907
ENSRNOT00000067396	chr4	ENSRNOG00000023077	Cpne9	145238947	145262442	+	0.7932	0.0005	0.0316	9.4649	15.6311	9.5806	9.8902	4.5969	6.8042	5.9259	8.3909
ENSRNOT00000067655	chr5	ENSRNOG00000010381	Mknk1	134691913	134730459	+	Inf	0.0000	0.0001	0	1.5360	2.3142	0	0	0	0	0
ENSRNOT00000067776	chr15	ENSRNOG00000012273	Dmtn	52292765	52320385	-	9.8922	0.0000	0.0001	0.0259	0.0117	0	48.6807	0.0214	0	0.0079	0.0220
ENSRNOT00000068177	chr16	ENSRNOG00000013884	Psd3	23475351	23781604	+	0.6983	0.0003	0.0247	13.4689	13.0032	17.8734	17.2185	13.4879	8.6535	6.6242	9.1756
ENSRNOT00000070827	chr9	ENSRNOG00000048239	Cxcr1	81466513	81469274	-	Inf	0.0001	0.0072	0	0.0755	0.0084	0.5023	0	0	0	0
ENSRNOT00000070876	chr17	ENSRNOG00000048358	LOC102553010	32009809	32015071	-	4.3751	0.0000	0.0026	0.1935	0.3711	0.6417	0.1716	0	0.0664	0	0
ENSRNOT00000070964	chr3	ENSRNOG00000050035	Sall4	165520392	165537940	-	Inf	0.0003	0.0214	0.0155	0.0747	0.0382	0.0697	0	0	0	0
ENSRNOT00000071248	chr20	ENSRNOG00000047175	Cryaa	10438444	10442187	+	Inf	0.0001	0.0112	0	0.0375	0.0694	0.2086	0	0	0	0
ENSRNOT00000071305	chr13	ENSRNOG00000046863	C1ql2	36378356	36380302	+	2.4633	0.0000	0.0001	4.3398	12.7195	12.0788	24.1666	4.2651	1.3957	3.1293	0.8758
ENSRNOT00000071603	chr2	ENSRNOG00000047158	LOC103689947	195416469	195423787	-	Inf	0.0000	0.0031	0	0	2.2252	0	0	0	0	0
ENSRNOT00000071891	chr1	ENSRNOG00000050201	Slc29a2	220307394	220314035	+	Inf	0.0000	0.0001	0	0	2.4166	1.9617	0	0	0	0
ENSRNOT00000072179	chr12	ENSRNOG00000049070	Rack1	35905358	35906382	-	Inf	0.0001	0.0129	0	0.2364	0.3882	0	0	0	0	0
ENSRNOT00000072206	chr1	ENSRNOG00000049394	LOC100912599	32573228	32581781	+	1.4873	0.0003	0.0235	20.3624	23.8048	14.5900	29.5909	0.6665	9.8277	11.3960	9.6207
ENSRNOT00000072536	chr16	ENSRNOG00000048472	Comp	20798437	20807070	-	1.8794	0.0010	0.0484	0.0642	0.0782	0.2698	0.0968	0.0421	0.0160	0.0634	0.0168
ENSRNOT00000072565	chr16	ENSRNOG00000049949	Hapln4	21081377	21089508	-	0.5308	0.0009	0.0453	30.0364	27.2909	28.0566	42.7830	17.1827	25.1695	19.3701	26.9921
ENSRNOT00000073220	chr1	ENSRNOG00000045795	LOC100911365	213951759	213957535	-	Inf	0.0000	0.0000	6.7233	0	6.3510	7.0734	0	0	0	0
ENSRNOT00000073259	chr20	ENSRNOG00000047102	Popdc3	50394673	50422551	+	Inf	0.0002	0.0151	0.2397	0	0.1818	0	0	0	0	0
ENSRNOT00000073788	chr3	ENSRNOG00000048433	Tshz2	166743544	166746597	+	0.7342	0.0009	0.0461	12.2328	10.9384	22.3592	25.4385	17.1808	7.6458	10.9195	6.9176
ENSRNOT00000073951	chr7	ENSRNOG00000047393	Krt18	143629455	143633131	+	1.4343	0.0008	0.0425	0.5665	1.9509	2.2351	2.0906	0.7327	0.5410	0.8879	0.3705
ENSRNOT00000074041	chr17	ENSRNOG00000050431	Aspn	15495801	15511972	-	Inf	0.0000	0.0003	0	0.2775	0.1798	0.1608	0	0	0	0
ENSRNOT00000074238	chr8	ENSRNOG00000009878	Crtapl1	122398276	122402127	-	2.5236	0.0006	0.0341	0.1088	1.0575	1.1269	0.0764	0.1133	0.0848	0.0845	0.1295

ENSRNOT00000074354	chr10	ENSRNOG00000046962	Pde6g	109620980	109622745	-	3.2892	0.0010	0.0488	0	1.0311	0.6052	0.6410	0.0935	0.1395	0	0
ENSRNOT00000074936	chr12	ENSRNOG00000045574	Morn3	38965063	38975400	+	1.9867	0.0002	0.0204	0.4067	1.3170	0.6200	1.0644	0.3204	0.1787	0.2508	0.1100
ENSRNOT00000074985	chr1	ENSRNOG00000037931	Plaur	81328183	81344705	+	Inf	0.0009	0.0464	0	0	0.5948	0	0	0	0	0
ENSRNOT00000075026	chr16	ENSRNOG00000049792	Glra3	37676962	37908173	-	Inf	0.0000	0.0032	0.2782	0	0.6670	0	0	0	0	0
ENSRNOT00000075513	chr6	ENSRNOG00000047455	Cdhr3	52345240	52401853	-	2.7128	0.0001	0.0097	0.1323	0.3264	0.3973	0.1553	0.0967	0.0286	0.0290	0
ENSRNOT00000075564	chr8	ENSRNOG00000049115	Ccr5	133197032	133214257	+	Inf	0.0001	0.0081	1.7346	0	0	0	0	0	0	0
ENSRNOT00000075606	chr11	ENSRNOG00000046177	LOC100912498	83896873	83905889	-	Inf	0.0000	0.0001	21.1607	0	0	0	0	0	0	0
ENSRNOT00000075624	chr5	ENSRNOG00000000138	Glis1	127160495	127233078	+	Inf	0.0005	0.0336	0	0	0	0.4592	0	0	0	0
ENSRNOT00000075786	chr15	ENSRNOG00000050996	Kctd4	58016238	58019442	+	0.7937	0.0001	0.0146	8.0535	10.6944	11.8085	16.5102	7.3080	6.5531	7.7403	5.5492
ENSRNOT00000076010	chr20	ENSRNOG00000047102	Popdc3	50394650	50422541	+	9.1014	0.0001	0.0080	0.1123	0.0931	0.0090	0.0740	0.0005	0	0	0
ENSRNOT00000076079	chrX	ENSRNOG00000002767	Dlg3	70596580	70648532	+	0.9435	0.0002	0.0200	9.7344	11.1104	9.3026	37.1320	9.8997	8.4178	8.4583	8.2077
ENSRNOT00000076160	chr5	ENSRNOG00000012274	Ddi2	160230955	160282810	-	1.4140	0.0001	0.0146	13.7656	12.7781	12.0208	12.9046	13.1626	1.8872	0.7016	3.5640
ENSRNOT00000076230	chr2	ENSRNOG00000033134	Mef2c	11658568	11820093	+	2.3059	0.0008	0.0427	0.3660	3.8768	0.2368	0.2914	0.2591	0.4222	0.2836	0
ENSRNOT00000076590	chrX	ENSRNOG00000004036	Snx12	71117528	71121034	-	2.1205	0.0003	0.0219	1.7008	0.7136	0.9531	13.4559	0.8823	1.2759	0.6870	1.0238
ENSRNOT00000076610	chr13	ENSRNOG00000003120	Prelp	50749542	50761306	-	10.4146	0.0000	0.0002	0.0065	5.6595	0.0079	0	0.0001	0.0001	0.0020	0.0019
ENSRNOT00000077428	chr14	ENSRNOG00000054513	LOC103693776	86652365	86661441	+	Inf	0.0000	0.0002	0	0	0	23.3889	0	0	0	0
ENSRNOT00000077701	chr19	ENSRNOG00000015397	Cpne7	55929664	55946250	+	1.3015	0.0000	0.0019	10.1153	11.9170	24.9609	25.5642	11.0761	5.9690	8.7423	3.6489
ENSRNOT00000077861	chr14	ENSRNOG00000022631	Fgf5	12975252	12995584	-	Inf	0.0002	0.0158	0.0714	0.0953	0	0.8721	0	0	0	0
ENSRNOT00000078179	chr14	ENSRNOG00000057703	Cabp7	84968130	84978255	-	1.7356	0.0000	0.0000	7.2143	23.6161	22.8018	40.7351	8.5799	6.5157	7.0653	6.1764
ENSRNOT00000078477	chr1	ENSRNOG00000014530	Nav2	104635989	104940171	+	1.3634	0.0000	0.0067	1.2472	3.3930	0.8125	4.8765	1.0149	0.9792	0.9962	1.0243
ENSRNOT00000078495	chr4	ENSRNOG00000023561	Ano2	158224000	158576978	+	7.2124	0.0003	0.0214	0.1531	0	0	0.1227	0	0.0019	0	0
ENSRNOT00000078733	chr4	ENSRNOG00000052731	AABR07059243.1	10992342	10995792	-	Inf	0.0000	0.0057	0	0	0	10.5413	0	0	0	0
ENSRNOT00000078760	chr8	ENSRNOG00000043085	Clstn2	105323837	105462168	-	0.5120	0.0009	0.0456	33.6556	32.8666	29.5003	46.2359	23.6624	28.3813	22.6033	25.1148
ENSRNOT00000078838	chr14	ENSRNOG00000007120	Mtmr3	84703263	84751886	-	1.4060	0.0002	0.0195	3.4757	2.4407	2.3927	21.0728	2.9469	3.2225	2.3078	2.6106
ENSRNOT00000078862	chr5	ENSRNOG00000022309	Frem1	101023247	101166651	-	1.7814	0.0002	0.0194	0.1326	0.3340	0.2654	0.6103	0.1400	0.0310	0.1351	0.0843
ENSRNOT00000079235	chr7	ENSRNOG00000056493	Mybpc1	29086156	29171783	-	1.5190	0.0004	0.0279	0.4763	3.1253	0.8327	1.2459	0.2636	0.6500	0.7888	0.2797
ENSRNOT00000079412	chr16	ENSRNOG00000014149	Npy1r	24779481	24788456	-	Inf	0.0003	0.0208	0	0.6362	0	0	0	0	0	0
ENSRNOT00000079466	chr16	ENSRNOG00000022482	Trappc11	47875091	47920823	+	7.3486	0.0000	0.0000	0.0046	1.2249	0.5932	0.8447	0.0029	0.0031	0.0053	0.0052

ENSRNOT00000079606	chr3	ENSRNOG00000048310	AABR07053518.1	112370057	112371664	-	Inf	0.0001	0.0068	0	0	3.5762	0	0	0	0	0
ENSRNOT00000079651	chr19	ENSRNOG00000042274	Fbxo31	53487826	53625665	-	2.5738	0.0002	0.0169	2.1020	6.4240	1.8340	31.2579	0.0084	0.4921	4.2510	2.2385
ENSRNOT00000079696	chr10	ENSRNOG00000004093	Rhot1	67559566	67639679	+	1.0550	0.0003	0.0214	15.8945	23.3394	6.7697	5.3559	5.3775	7.6164	6.4721	5.2535
ENSRNOT00000079719	chr6	ENSRNOG00000009079	Prkar2b	51267096	51316943	-	3.2324	0.0007	0.0370	0.0166	0.3990	0.3957	0	0.0146	0.0275	0.0113	0.0329
ENSRNOT00000079744	chr4	ENSRNOG00000012876	Slc6a13	153874852	153912155	+	1.0826	0.0010	0.0470	3.1087	1.4245	1.8973	3.5614	1.4314	1.0834	1.3863	0.8168
ENSRNOT00000079838	chr20	ENSRNOG00000040052	RT1-M6-2	2076583	2081885	+	Inf	0.0004	0.0269	0	0	0	1.0340	0	0	0	0
ENSRNOT00000079844	chr1	ENSRNOG00000019751	Cyb5r2	171971896	171979757	-	3.4444	0.0001	0.0114	0.0166	0.8623	0.1977	0.1292	0.0114	0.0707	0.0174	0.0114
ENSRNOT00000079987	chr1	ENSRNOG00000017302	Slc6a3	32321580	32362359	-	5.9284	0.0000	0.0004	0.0908	0.0369	14.0404	0.1222	0.1645	0	0.0401	0.0301
ENSRNOT00000080068	chr12	ENSRNOG00000001422	Col26a1	22965025	22980603	+	Inf	0.0006	0.0348	0	0	0	0.3622	0	0	0	0
ENSRNOT00000080170	chr13	ENSRNOG00000059202	AABR07021988.1	101727935	101748098	-	3.1748	0.0000	0.0012	0.8941	19.8422	0.6872	0.6838	0.7556	0.5935	0.3269	0.7721
ENSRNOT00000080246	chr1	ENSRNOG00000020369	Igf2	215828102	215836641	-	0.9447	0.0002	0.0199	2.9025	7.6559	5.4216	7.1165	2.5561	3.2528	4.4008	1.7900
ENSRNOT00000080283	chr20	ENSRNOG00000000322	Atg5	49318308	49391940	+	6.3465	0.0001	0.0121	7.1463	0	0	0	0.0589	0	0.0104	0.0185
ENSRNOT00000080288	chr8	ENSRNOG00000053239	Clmp	44847157	44975457	+	0.7043	0.0003	0.0231	7.1066	7.1234	6.8612	8.4534	5.2651	4.6702	4.3058	3.8923
ENSRNOT00000080470	chr2	ENSRNOG00000011076	Ank2	231232925	231521052	-	16.2304	0.0000	0.0006	0	0	1.5377	0	0	0	0	0
ENSRNOT00000080604	chr14	ENSRNOG00000005094	C1qtnf7	72011518	72025137	-	Inf	0.0000	0.0050	0.2307	0.0319	0.1125	0	0	0	0	0
ENSRNOT00000080758	chr14	ENSRNOG00000056944	Arhgap24	8383237	8510138	-	6.8800	0.0000	0.0000	1.3618	1.5751	1.4186	0.5685	0.0129	0.0211	0.0078	0
ENSRNOT00000080832	chr16	ENSRNOG00000046973	Tmco3	80904125	81072145	+	Inf	0.0000	0.0028	1.3188	0	0	0	0	0	0	0
ENSRNOT00000081106	chr9	ENSRNOG00000014806	Pnkd	81615251	81632360	+	2.3080	0.0004	0.0270	0.6693	0.1686	1.2007	0.7921	0.0487	0.0650	0.0392	0.4187
ENSRNOT00000081247	chr10	ENSRNOG00000019075	Stat5b	88686208	88712309	-	Inf	0.0000	0.0033	0	0	0	1.5837	0	0	0	0
ENSRNOT00000081433	chr6	ENSRNOG00000050553	Atp6v1c2	42585934	42616548	-	1.8691	0.0011	0.0499	0.3048	0.5102	0.8907	0.4329	0.3925	0.1209	0.0297	0.0423
ENSRNOT00000081501	chr2	ENSRNOG00000018937	Gstm7	210720704	210782856	-	2.4932	0.0005	0.0300	0.5370	0.1057	0.1133	0.0976	0.0676	0.0372	0.0329	0.0139
ENSRNOT00000081784	chr7	ENSRNOG00000060020	C1ql4	140767737	140770647	-	Inf	0.0000	0.0004	0.0720	0.1637	0.8828	0.1453	0	0	0	0
ENSRNOT00000081813	chr10	ENSRNOG00000003495	Prpf8	63635219	63658361	+	0.6393	0.0008	0.0426	56.8721	56.4926	57.7257	54.6096	55.0556	24.2666	16.9534	48.6315
ENSRNOT00000081893	chr5	ENSRNOG00000013166	Wnt4	155660553	155672579	+	0.9386	0.0001	0.0101	2.7472	4.9703	3.8744	6.7283	2.5841	2.1459	2.6553	2.1731
ENSRNOT00000082084	chr2	ENSRNOG00000009730	Cyp7b1	102702689	102838745	-	4.8096	0.0000	0.0006	1.4037	0.0157	0.0413	1.1816	0.0109	0.0375	0.0171	0.0288
ENSRNOT00000082164	chr1	ENSRNOG00000015086	Plin1	141458181	141471010	-	3.3498	0.0008	0.0417	0.0356	0.0142	0.0604	0.0250	0	0	0.0133	0
ENSRNOT00000082175	chr4	ENSRNOG00000008775	Lrguk	61420009	61544446	+	7.9861	0.0002	0.0191	0	0	0.7173	0	0	0	0	0.0028
ENSRNOT00000082271	chr7	ENSRNOG00000023781	Plec	117230319	117267803	-	9.4999	0.0000	0.0001	0.0073	0	0.0055	7.6357	0.0021	0.0054	0.0030	0

ENSRNOT00000082401	chr8	ENSRNOG00000010634	Megf11	70112925	70178561	+	3.6152	0.0008	0.0432	0.0035	0.0069	0.3320	0.0059	0.0162	0.0070	0.0038	0.0014
ENSRNOT00000082537	chr4	ENSRNOG00000057335	Clec1b	163162211	163170466	+	2.7151	0.0007	0.0372	0.0342	0.4772	0.1995	0.2606	0.0847	0	0	0.0632
ENSRNOT00000082586	chr5	ENSRNOG00000022694	Plekhg5	169274785	169287448	+	2.6071	0.0007	0.0377	0.0777	0.5146	0.2642	0.0188	0.0201	0.0004	0.1071	0.0160
ENSRNOT00000082593	chr12	ENSRNOG00000059456	Grk3	49626871	49746272	+	Inf	0.0000	0.0000	11.6408	10.0825	14.9183	0	0	0	0	0
ENSRNOT00000082602	chr5	ENSRNOG00000016183	Ipp	135351816	135387648	+	1.9650	0.0005	0.0300	0.2156	0.1529	0.2717	1.1570	0.1054	0.0715	0.1806	0.1028
ENSRNOT00000082688	chr15	ENSRNOG00000024237	Scel	87722221	87820779	+	1.5600	0.0006	0.0341	0.5306	0.4058	0.7490	0.4463	0.3438	0.1389	0.1392	0.1010
ENSRNOT00000083007	chr13	ENSRNOG00000056580	En1	36532758	36537093	+	3.3280	0.0005	0.0307	0.0289	0.0690	1.2563	0.1043	0.0862	0	0.0591	0
ENSRNOT00000083302	chr10	ENSRNOG00000053055	Otop3	103891369	103898331	+	1.9410	0.0006	0.0365	0.1276	0.4301	0.6390	1.4090	0.2408	0.1682	0.2506	0.0189
ENSRNOT00000083527	chr3	ENSRNOG00000009401	Lmo2	93920013	93931871	+	2.2955	0.0006	0.0349	0.8718	4.2807	2.4590	4.4655	0	1.7826	0.0838	0.5935
ENSRNOT00000083566	chrX	ENSRNOG00000054495	Cldn2	111122552	111133191	+	2.0883	0.0002	0.0173	0.1625	5.9036	4.2565	4.0764	1.0007	1.3477	0.9853	0.0524
ENSRNOT00000083642	chr18	ENSRNOG00000016675	Kcnn2	39335377	39479257	+	6.9716	0.0000	0.0007	0.0691	2.8942	0.0033	0.0019	0.0054	0.0058	0.0069	0.0055
ENSRNOT00000083656	chr1	ENSRNOG00000027564	Tsga10ip	220772411	220787238	-	2.9500	0.0010	0.0466	0.0863	0.0469	0.0088	0.0171	0	0	0.0071	0.0135
ENSRNOT00000083684	chr19	ENSRNOG00000016311	Slc6a2	15391581	15421404	-	Inf	0.0003	0.0230	0.0197	0.0079	0	0.0079	0	0	0	0
ENSRNOT00000083850	chr3	ENSRNOG00000011732	Psmb7	22871105	22952063	-	5.4022	0.0000	0.0020	0.5446	0	0.5343	0.5627	0.0388	0	0	0
ENSRNOT00000084216	chr5	ENSRNOG00000032539	Spag8	59147337	59149699	-	2.0867	0.0004	0.0265	0.4697	0.5129	0.2807	0.9809	0.2108	0.0286	0.0312	0.2576
ENSRNOT00000084302	chr2	ENSRNOG00000014002	St7l	207351051	207473857	+	5.8330	0.0000	0.0010	0.9526	0.9325	0	0.0685	0.0311	0.0032	0	0
ENSRNOT00000084350	chr15	ENSRNOG00000009042	Sec24c	4035201	4057104	-	3.0839	0.0006	0.0360	0.0043	5.4137	0.2008	0.0023	0.2396	0.2859	0.1253	0.0121
ENSRNOT00000084640	chr5	ENSRNOG00000007890	Usp1	117586103	117594335	+	Inf	0.0000	0.0039	0	0	1.3328	0	0	0	0	0
ENSRNOT00000084736	chr4	ENSRNOG00000056243	ST7	45000973	45247788	+	4.9079	0.0000	0.0049	0.0098	1.4236	0.0202	0.0008	0.0082	0.0131	0.0105	0.0166
ENSRNOT00000084777	chr4	ENSRNOG00000012764	Agap3	7203003	7252950	-	2.4881	0.0001	0.0102	0.1891	0.3614	0.1514	3.6087	0.1706	0.1584	0.2106	0.2287
ENSRNOT00000085747	chr12	ENSRNOG00000057769	Oas1h	41214587	41224254	-	3.8992	0.0001	0.0084	0.0630	0.1330	0.0123	0.1463	0	0	0	0.0238
ENSRNOT00000085754	chr11	ENSRNOG00000042289	Plcxd2	57207679	57260568	+	2.6918	0.0005	0.0318	0.0299	0.5383	0.0572	0.0298	0.0463	0.0172	0.0197	0.0182
ENSRNOT00000086097	chr11	ENSRNOG00000002265	Casr	67221359	67258771	+	4.3886	0.0004	0.0266	0	0.0105	0.0051	0.4411	0	0.0107	0.0111	0
ENSRNOT00000086247	chr6	ENSRNOG00000014879	Ttc7a	10912488	11013048	+	Inf	0.0005	0.0305	0.2788	0	0	0	0	0	0	0
ENSRNOT00000086279	chr2	ENSRNOG00000016394	Wdr77	208420309	208587260	+	6.4244	0.0000	0.0010	5.2461	0.0032	0.0071	0.0059	0.0216	0.0056	0.0165	0.0176
ENSRNOT00000086424	chr16	ENSRNOG00000011912	Tmem38a	18959356	18974729	-	Inf	0.0003	0.0226	0.5251	0	0	0	0	0	0	0
ENSRNOT00000086425	chr3	ENSRNOG00000020086	Aar2	152626757	152648244	+	4.3113	0.0000	0.0033	0.0368	0.0468	2.3246	0.0579	0.0554	0.0457	0	0.0232
ENSRNOT00000086646	chr14	ENSRNOG00000056826	Arap2	48726045	48922807	+	1.7460	0.0004	0.0263	0.2737	0.1640	1.6771	0.2531	0.1922	0.1542	0.1121	0.2475

ENSRNOT00000086886	chr7	ENSRNOG00000060185	LOC103690317	140197530	140213045	-	Inf	0.0000	0.0015	0	0	0	5.2547	0	0	0	0
ENSRNOT00000087037	chr16	ENSRNOG00000051993	Gdf10	10250404	10262383	+	1.1466	0.0005	0.0302	1.6495	2.5299	2.3580	3.2125	1.2709	0.9076	1.3635	0.8620
ENSRNOT00000087115	chr4	ENSRNOG00000019716	Ntf3	158636884	158705885	-	Inf	0.0003	0.0241	0	0	0.6827	0	0	0	0	0
ENSRNOT00000087289	chr3	ENSRNOG00000057817	LOC100911769	152571121	152599512	+	Inf	0.0000	0.0033	0.3036	0.2232	0	0	0	0	0	0
ENSRNOT00000087408	chr18	ENSRNOG00000013867	Fgf1	32273770	32359824	+	1.0439	0.0004	0.0257	1.9467	6.4254	6.4980	3.7617	2.8957	1.9942	3.0496	1.0973
ENSRNOT00000087613	chr20	ENSRNOG00000000804	Mrps18b	3344915	3350780	+	2.6633	0.0009	0.0455	0.1648	0.9492	0.4116	0.2881	0	0	0.2266	0.0596
ENSRNOT00000087873	chr16	ENSRNOG00000014363	Arhgef3	2743823	3025019	+	2.1436	0.0010	0.0469	0.1240	0.3950	0.0992	0.4623	0.1387	0.0381	0.0677	0
ENSRNOT00000087907	chr3	ENSRNOG00000055809	Epb41l1	153848242	153876938	-	1.9641	0.0000	0.0000	6.0274	6.4949	2.9649	5.6230	1.6114	1.2398	1.2090	1.3504
ENSRNOT00000088148	chr1	ENSRNOG00000047104	LOC100911660	257766691	257949180	+	10.1281	0.0000	0.0040	0	0.9504	0.0557	0	0.0009	0	0	0
ENSRNOT00000088287	chr20	ENSRNOG00000000824	Dse	27703732	27757149	-	2.4647	0.0007	0.0406	0.1226	0.1991	0.0055	0.5209	0.0016	0.0459	0.0992	0.0069
ENSRNOT00000088441	chr14	ENSRNOG00000002278	Tec	37918652	38027920	+	6.6517	0.0000	0.0000	2.0009	0.0037	1.9067	2.2432	0.0036	0.0205	0.0017	0.0355
ENSRNOT00000088710	chr5	ENSRNOG00000056358	Ccdc27	171312793	171327450	-	Inf	0.0000	0.0002	0.0344	0.0920	0.0584	0.2192	0	0	0	0
ENSRNOT00000088945	chr7	ENSRNOG00000023781	Plec	117230319	117267402	-	4.4601	0.0008	0.0429	0	0.0016	0	0.1469	0.0029	0	0	0.0038
ENSRNOT00000089138	chr17	ENSRNOG00000019232	RGD1311345	6684621	6690723	+	Inf	0.0003	0.0254	0	0.1707	0.0679	0	0	0	0	0
ENSRNOT00000089177	chr4	ENSRNOG00000005433	Shq1	133037014	133131953	-	3.5194	0.0007	0.0389	0.0265	0.0616	0.0202	0.0099	0	0	0.0103	0
ENSRNOT00000089257	chr20	ENSRNOG00000000818	Nrm	3397834	3401273	-	Inf	0.0000	0.0005	0	0.1558	0.2308	0.6616	0	0	0	0
ENSRNOT00000089482	chr4	ENSRNOG00000013547	Slc6a12	153921900	153940963	+	Inf	0.0001	0.0137	0.1749	0	0	0.1495	0	0	0	0
ENSRNOT00000089574	chr5	ENSRNOG00000012531	Ephb2	155022493	155204456	-	1.2391	0.0008	0.0421	0.8442	2.8034	0.6878	0.6582	0.5775	0.4391	0.6085	0.4904
ENSRNOT00000089975	chr19	ENSRNOG00000011180	Irx5	16415636	16421191	+	4.6183	0.0000	0.0031	0.0130	0.1174	0.2674	0.2117	0	0	0.0180	0.0068
ENSRNOT00000090017	chr6	ENSRNOG00000008299	Allc	47799082	47848075	-	Inf	0.0002	0.0198	0	0.0177	0.3938	0	0	0	0	0
ENSRNOT00000090375	chr1	ENSRNOG00000050994	Cttn	215256629	215284548	-	Inf	0.0000	0.0000	6.6701	0	0	2.8410	0	0	0	0
ENSRNOT00000090383	chr9	ENSRNOG00000014806	Pnkd	81566074	81634534	+	2.5926	0.0000	0.0057	0.2777	5.3005	0.2811	0.4460	0.2442	0.2619	0.2459	0.2933
ENSRNOT00000090431	chr4	ENSRNOG00000030522	LOC100910725	162077188	162168221	+	5.4705	0.0005	0.0307	0	0.0778	0.0473	0.1800	0.0069	0	0	0
ENSRNOT00000090502	KL568417.1	ENSRNOG00000053118	LOC103694867	15968	35620	+	Inf	0.0000	0.0000	3.0012	3.7937	4.1351	0.0021	0	0	0	0
ENSRNOT00000090653	chr1	ENSRNOG00000020865	Ano1	217754336	217844957	-	Inf	0.0000	0.0001	1.0577	0	0	1.0893	0	0	0	0
ENSRNOT00000090730	chr7	ENSRNOG00000060185	LOC103690317	140196953	140213050	-	Inf	0.0000	0.0010	0	0	0	5.5837	0	0	0	0
ENSRNOT00000090802	chr3	ENSRNOG00000004812	Sema6d	116899878	116956169	+	0.8148	0.0001	0.0069	8.3415	17.6813	16.7710	15.6239	8.1258	8.6878	8.5847	7.8124
ENSRNOT00000090838	chr1	ENSRNOG00000020695	Tead2	101213788	101230477	+	Inf	0.0000	0.0016	0.3290	0.5715	0	0	0	0	0	0

ENSRNOT00000090848	chr13	ENSRNOG00000004018	Tdrd5	73872621	73921969	-	Inf	0.0000	0.0001	0.0446	0.0156	0.1640	0.0938	0	0	0	0
ENSRNOT00000091610	chr10	ENSRNOG00000046271	LOC100911685	4312863	4446200	+	1.2622	0.0001	0.0132	9.0674	3.4816	3.1495	17.4564	3.0268	4.2959	3.3604	3.1391
ENSRNOT00000092505	chrX	ENSRNOG00000002451	Fndc3c1	77431005	77570825	-	4.6240	0.0006	0.0362	0.3485	0	0.0613	0	0	0	0.0166	0
ENSRNOT00000092639	chrX	ENSRNOG00000052226	LOC108348144	123803103	123806736	-	2.5540	0.0001	0.0070	2.0333	3.3057	0.1754	0.1232	0.2286	0.2806	0.1765	0.2742
ENSRNOT00000092757	chr1	ENSRNOG00000012609	Trdn	25716396	25839279	-	Inf	0.0000	0.0040	0.0421	0.0640	0.0801	0.0548	0	0	0	0
ENSRNOT00000092802	chr13	ENSRNOG00000025701	Nifk	34267614	34273463	+	4.0373	0.0000	0.0002	0.9892	1.9466	1.5882	0.1844	0.0781	0.1281	0.0284	0.0522
ENSRNOT00000093186	chr5	ENSRNOG00000006096	Slc26a7	27987414	28130803	-	4.5537	0.0001	0.0148	0.0009	0.0065	0.4287	0.0491	0.0207	0	0	0
ENSRNOT00000093201	chr11	ENSRNOG00000002171	Phldb2	57430166	57481589	+	Inf	0.0000	0.0001	0	0	1.3385	1.8043	0	0	0	0
ENSRNOT00000093462	chr5	ENSRNOG00000006956	AABR07049085.1	104394119	104714197	+	6.9233	0.0000	0.0027	0	0.5790	0	1.4302	0	0.0166	0	0

**Day-28 mRNA**	**Chr**	**Gene ID**	**Gene Symbol**	**Start**	**End**	**Strand**	**log2(FC)**	***P* Value**	***Q* Value**	**ICH1**	**ICH2**	**ICH3**	**ICH4**	**Sham1**	**Sham2**	**Sham3**	**Sham4**
ENSRNOT00000002557	chr11	ENSRNOG00000001872	Lrrc74b	87418205	87434482	-	-3.9056	0.0000	0.0017	0.0268	0	0	0.0253	0.2447	0.2399	0.1984	0.0970
ENSRNOT00000003198	chr13	ENSRNOG00000002339	Mark1	102809549	102942863	-	-1.0822	0.0001	0.0104	7.0259	5.9187	5.3139	4.5165	16.9528	19.6700	5.6920	5.9049
ENSRNOT00000003405	chr13	ENSRNOG00000002517	Pdc	67545430	67558600	+	-Inf	0.0012	0.0499	0	0	0	0	0.0227	0.0613	0	0.0226
ENSRNOT00000003567	chr13	ENSRNOG00000002525	Ptgs2	67351087	67359335	+	-0.6336	0.0008	0.0394	8.1456	4.9633	6.7523	4.4870	7.2367	9.7960	10.6427	10.0993
ENSRNOT00000003741	chrX	ENSRNOG00000002767	Dlg3	70596577	70647298	+	-0.5480	0.0008	0.0390	17.5511	11.5022	10.9527	14.3908	20.2583	21.0223	17.6145	20.6335
ENSRNOT00000003928	chr13	ENSRNOG00000002863	Cacna1e	71907171	72367980	-	-0.5696	0.0003	0.0216	23.5227	25.0914	17.2008	14.6225	25.7669	32.9345	29.8345	30.8417
ENSRNOT00000004408	chr13	ENSRNOG00000003300	Btg2	50913180	50916982	-	-0.8633	0.0004	0.0273	20.5390	14.8932	16.5298	3.9347	21.4999	28.6416	25.9962	25.5523
ENSRNOT00000004703	chr7	ENSRNOG00000016156	Nptxr	121011680	121029754	-	-0.4605	0.0009	0.0431	301.9932	304.9721	198.6460	197.0907	344.4196	326.5628	351.8714	356.8502
ENSRNOT00000005067	chr10	ENSRNOG00000003741	Nptx1	108682638	108691367	-	-0.4905	0.0008	0.0401	96.8446	71.1952	57.2275	61.3438	94.5764	115.9208	100.2802	91.8946
ENSRNOT00000005129	chr10	ENSRNOG00000003865	Tmigd1	63259427	63274640	-	-2.4934	0.0003	0.0199	0.0134	0.0084	0	0.0173	0.0679	0.0174	0.0603	0.0745
ENSRNOT00000005324	chr13	ENSRNOG00000003959	Rgs18	61565483	61591139	-	-1.4198	0.0007	0.0386	1.2469	0.5175	0.8643	1.3824	2.8304	2.8282	2.2147	2.8582
ENSRNOT00000005663	chrX	ENSRNOG00000004197	Asb12	64714350	64800435	-	-Inf	0.0006	0.0340	0	0	0	0	0.0436	0.0296	0.0034	0.0036
ENSRNOT00000006067	chr10	ENSRNOG00000004146	Coro7	11100917	11143881	+	-1.0699	0.0003	0.0210	4.6454	6.0549	2.5423	6.1447	3.6869	16.3224	14.5062	6.1843
ENSRNOT00000006083	chr7	ENSRNOG00000004527	Lin7a	49385705	49627836	+	-5.4224	0.0000	0.0024	0	0.1666	0	0	2.7882	2.6167	1.7394	0
ENSRNOT00000006339	chr6	ENSRNOG00000019584	Dlk1	133552821	133583498	+	-Inf	0.0000	0.0027	0	0	0	0	0.3330	0	0	0.5939
ENSRNOT00000006389	chr3	ENSRNOG00000004810	Plcb1	128155117	128419545	+	-0.5198	0.0011	0.0490	46.6068	42.2560	35.4609	25.3128	45.8176	69.4682	41.0799	58.1816
ENSRNOT00000007103	chr3	ENSRNOG00000005258	Myef2	117354581	117389456	-	-3.6951	0.0000	0.0000	2.0645	0.5759	1.6589	0.5818	29.9796	29.2217	2.4723	1.5479
ENSRNOT00000007321	chr13	ENSRNOG00000005229	Sec16b	75177965	75216933	+	-Inf	0.0000	0.0001	0	0	0	0	0.5944	0.7412	0	0

ENSRNOT00000007795	chr15	ENSRNOG00000005883	Nek10	11635932	11812485	-	-Inf	0.0000	0.0007	0	0	0	0	0	0.3846	0.2841	0
ENSRNOT00000008302	chr5	ENSRNOG00000005964	Nr4a3	63781801	63821637	+	-0.7281	0.0003	0.0201	5.1472	7.6398	9.7768	6.4003	7.5676	11.0852	15.1098	14.2148
ENSRNOT00000008350	chr7	ENSRNOG00000006118	Klf10	77156006	77162148	-	-0.6950	0.0005	0.0286	9.9032	6.5778	8.2710	5.1036	9.6264	12.1788	13.4147	13.1144
ENSRNOT00000008898	chr5	ENSRNOG00000006735	Cdkn2b	107852129	107857320	-	-Inf	0.0000	0.0056	0	0	0	0	0.3532	0.1252	0	0
ENSRNOT00000009153	chr5	ENSRNOG00000006979	Hpca	147294820	147303346	-	-0.5744	0.0007	0.0362	212.5038	97.3113	109.6563	117.3789	195.0912	192.8259	231.5340	179.9715
ENSRNOT00000009248	chr5	ENSRNOG00000006889	Ambp	78975678	78985990	-	-Inf	0.0009	0.0435	0	0	0	0	0.0463	0	0.0456	0.0233
ENSRNOT00000009421	chr14	ENSRNOG00000007049	Bcl11a	108826939	108913078	+	-0.5363	0.0011	0.0471	21.2620	12.5198	15.7900	16.6906	20.1131	24.6123	26.3599	25.0122
ENSRNOT00000009979	chr7	ENSRNOG00000007346	Grasp	142869752	142878012	+	-0.5996	0.0008	0.0396	18.0452	12.3397	12.2036	11.2531	18.8572	20.0200	20.9541	21.7533
ENSRNOT00000010171	chr7	ENSRNOG00000007607	Nr4a1	142912316	142920216	+	-1.0025	0.0002	0.0176	23.7568	14.8297	34.5938	5.1275	26.3613	47.7440	40.6161	42.1629
ENSRNOT00000010289	chr4	ENSRNOG00000007830	Apold1	168752133	168755023	+	-1.4294	0.0000	0.0044	8.3282	11.2343	14.9223	1.9313	10.3916	20.5839	24.7410	42.3631
ENSRNOT00000010851	chr3	ENSRNOG00000008165	Tpx2	148327965	148370187	+	-4.7325	0.0000	0.0002	0.0376	0.0360	0.0501	0.0114	0.2288	1.1866	2.1777	0.0002
ENSRNOT00000011904	chr7	ENSRNOG00000008697	Nov	94375020	94383024	+	-0.7904	0.0000	0.0023	66.7297	45.1927	36.2742	54.7688	75.1517	86.4514	89.7205	99.7268
ENSRNOT00000013459	chr5	ENSRNOG00000009519	LOC108348114	129052043	129082208	+	-Inf	0.0006	0.0335	0	0	0	0	0	0	0.0326	0.1221
ENSRNOT00000013646	chr5	ENSRNOG00000010270	Espn	169293904	169301728	-	-Inf	0.0002	0.0144	0	0	0	0	0	0.1106	0.0564	0.0330
ENSRNOT00000014129	chr3	ENSRNOG00000010528	LOC100911166	112519808	112525575	+	-6.5167	0.0001	0.0077	0	0	0	0.0208	0	1.8832	0	0.0234
ENSRNOT00000015279	chr2	ENSRNOG00000010889	Fbxw7	184231830	184263564	-	-0.8289	0.0007	0.0369	38.3353	16.6457	23.2816	18.0076	25.9024	84.9414	31.8443	28.3236
ENSRNOT00000015366	chr8	ENSRNOG00000011543	Fam118b	36416997	36438730	-	-1.6139	0.0001	0.0112	1.4037	1.2600	1.2719	1.7662	1.3135	6.5840	1.7296	7.8251
ENSRNOT00000018137	chr1	ENSRNOG00000013413	Rorb	234363994	234435478	+	-5.8105	0.0003	0.0234	0	0.0045	0.0055	0	0	0.3417	0	0.2170
ENSRNOT00000019501	chr2	ENSRNOG00000014350	Cyr61	251529354	251532312	-	-1.1698	0.0010	0.0466	6.1231	3.4606	4.9117	0.4127	5.6011	7.0749	11.1378	9.7270
ENSRNOT00000019829	chr3	ENSRNOG00000028908	Eppin	161041727	161050557	-	-2.9449	0.0007	0.0371	0	0	0.0250	0	0.0319	0.0624	0.0490	0.0491
ENSRNOT00000020940	chr1	ENSRNOG00000015267	Aldh18a1	259057413	259089632	-	-7.1615	0.0000	0.0035	0.0022	0.0012	0.0025	0	0.0011	0	0.8492	0.0016
ENSRNOT00000021596	chr17	ENSRNOG00000015616	Rgs14	9777925	9792007	-	-0.6081	0.0005	0.0307	16.4929	15.3347	9.4143	11.9260	18.0250	22.4806	20.8887	19.6452
ENSRNOT00000022292	chr2	ENSRNOG00000016550	Dclk2	186119729	186245361	-	-1.2753	0.0000	0.0018	3.4587	3.7957	4.6123	3.8194	11.0159	3.5725	11.1277	12.2526
ENSRNOT00000022583	chr8	ENSRNOG00000029826	Cep164	50070579	50126413	-	-Inf	0.0000	0.0011	0	0	0	0	0	0	0	1.7325
ENSRNOT00000022625	chr2	ENSRNOG00000016872	Plppr4	220298245	220341866	+	-0.4433	0.0010	0.0452	61.9589	59.5167	42.5397	51.1023	69.4485	67.5530	82.0594	73.4399
ENSRNOT00000023584	chr2	ENSRNOG00000017462	Spata5	124149872	124341124	+	-2.9312	0.0010	0.0462	0.0100	0.0602	0	0	0.0042	0.2095	0.2383	0.0840
ENSRNOT00000023843	chr7	ENSRNOG00000017553	Mief1	121463039	121475346	+	-4.5022	0.0007	0.0363	0.0837	0	0	0.3804	0	0.3259	10.1920	0
ENSRNOT00000025137	chr18	ENSRNOG00000018525	Slc4a9	29352749	29365595	+	-Inf	0.0000	0.0052	0	0	0	0	0.0058	0.0173	0.0105	0.0313

ENSRNOT00000025365	chr2	ENSRNOG00000018716	Dennd2c	205617220	205660611	+	-Inf	0.0000	0.0024	0	0	0	0	0.7009	0	0	0
ENSRNOT00000025826	chr1	ENSRNOG00000047551	Pnpla2	213997709	214002815	+	-Inf	0.0000	0.0008	0	0	0	0	0	0	4.0188	0
ENSRNOT00000026303	chr18	ENSRNOG00000019422	Egr1	27657628	27661429	+	-0.6844	0.0006	0.0338	56.7663	34.2536	62.7898	18.8870	61.2485	83.2316	66.2627	66.7833
ENSRNOT00000026428	chr1	ENSRNOG00000019491	Stard10	166433109	166457505	+	-2.9240	0.0003	0.0220	0	0.2450	0.0968	0.1229	0.1817	0.2007	2.9862	0.1584
ENSRNOT00000026657	chr3	ENSRNOG00000050864	LOC100910990	151616037	151625644	-	-Inf	0.0000	0.0003	0	0	0	0	5.2617	0	0	0
ENSRNOT00000026705	chr2	ENSRNOG00000019638	Lmna	187842885	187863516	-	-7.4620	0.0000	0.0008	0.0009	0.0018	0.0011	0.0007	0.0025	0.4145	0.0014	0.3902
ENSRNOT00000027268	chr16	ENSRNOG00000020134	Upf1	20825044	20845213	+	-Inf	0.0000	0.0007	0	0	0	0	0	3.3842	0	0
ENSRNOT00000028064	chr1	ENSRNOG00000020650	Slc17a7	101161252	101173174	+	-0.5790	0.0003	0.0194	444.1902	251.9264	263.3002	264.1367	391.0326	495.3023	483.7171	457.7623
ENSRNOT00000028096	chr8	ENSRNOG00000020694	Icam5	22050222	22057209	+	-0.5399	0.0005	0.0282	61.3518	62.2766	37.1836	47.4052	74.0106	63.3298	85.0576	80.3249
ENSRNOT00000028265	chr1	ENSRNOG00000049277	Syne4	88772904	88776999	+	-Inf	0.0000	0.0004	0	0	0	0	0	0.2100	0.2941	0.4113
ENSRNOT00000028283	chr1	ENSRNOG00000050994	Cttn	215255386	215290838	-	-Inf	0.0000	0.0002	0	0	0	0	0	0	14.0447	0
ENSRNOT00000030007	chr15	ENSRNOG00000027839	Ptk2b	42827310	42947656	-	-0.4699	0.0005	0.0311	116.8495	98.2592	83.4590	81.8526	131.1102	130.4887	138.4925	126.8083
ENSRNOT00000032904	chr1	ENSRNOG00000018061	Tmem80	214317682	214326291	+	-1.1653	0.0009	0.0442	2.6754	3.3705	2.5233	3.6871	11.3222	1.9620	2.3001	11.9047
ENSRNOT00000033940	chr2	ENSRNOG00000021745	Bhlhe22	102685513	102688624	+	-0.6477	0.0009	0.0436	11.1154	21.9681	10.3869	16.9753	24.2325	14.1499	32.1371	24.1781
ENSRNOT00000035415	chr1	ENSRNOG00000026065	Slit1	260843678	260992291	-	-1.2600	0.0000	0.0044	1.3173	1.9055	1.6683	2.4067	2.0497	2.8962	6.8230	5.7086
ENSRNOT00000035678	chr7	ENSRNOG00000025725	Ly6k	116034104	116106368	-	-Inf	0.0003	0.0203	0	0	0	0	0.0271	0.1455	0	0.0777
ENSRNOT00000036954	chr10	ENSRNOG00000028075	LOC360479	10795229	10808585	-	-3.8418	0.0000	0.0032	0	0	0.0691	0.0029	0.2063	0.0857	0.3323	0.4077
ENSRNOT00000038748	chr3	ENSRNOG00000026061	Sirpb3	121978126	122057632	-	-Inf	0.0009	0.0423	0	0	0	0	0	0.0233	0.1012	0
ENSRNOT00000039677	chr1	ENSRNOG00000020857	Ppfia1	217644259	217720221	-	-5.2836	0.0003	0.0201	0	0.0017	0	0.0038	0.0057	0	0.0048	0.2035
ENSRNOT00000041394	chr3	ENSRNOG00000005600	Nr4a2	43111240	43119159	-	-1.0703	0.0000	0.0006	8.7872	7.2833	9.2246	15.4362	16.4244	22.4582	26.3831	20.2639
ENSRNOT00000041533	chr18	ENSRNOG00000018712	Camk2a	56193978	56295869	+	-0.4912	0.0007	0.0380	538.2367	335.5749	374.2184	306.0003	509.6637	575.1530	581.1025	518.4207
ENSRNOT00000042331	chr12	ENSRNOG00000001120	Med13l	43423905	43576754	-	-1.0752	0.0010	0.0458	0.3680	0.3112	1.7439	1.6058	2.0920	2.1425	2.2848	1.9694
ENSRNOT00000042642	chr7	ENSRNOG00000023781	Plec	117230321	117289961	-	-0.9791	0.0000	0.0007	6.9940	4.8113	3.9551	8.0702	13.9152	13.5212	9.4403	10.0986
ENSRNOT00000042900	chr6	ENSRNOG00000030812	AABR07065781.1	141146719	141147264	-	-Inf	0.0003	0.0226	0	0	0	0	0	1.8847	0	0
ENSRNOT00000043508	chr6	ENSRNOG00000032348	LOC108349682	135139292	135139621	+	-2.7492	0.0000	0.0042	1.6902	1.3815	0.8923	1.2199	28.4213	1.9292	2.3519	2.1509
ENSRNOT00000046013	chr17	ENSRNOG00000017863	Zeb1	54658463	54678710	-	-3.9100	0.0004	0.0238	0.0089	0	0.0049	0.0082	0.0009	0.0017	0.0249	0.3035
ENSRNOT00000046700	chr2	ENSRNOG00000016070	Trpc3	123330041	123396386	-	-Inf	0.0008	0.0398	0	0	0	0	0	0	0.1467	0
ENSRNOT00000047547	chr7	ENSRNOG00000026976	AABR07056369.1	22927796	22943159	-	-Inf	0.0000	0.0062	0	0	0	0	0.0178	0.0140	0.0115	0.0310

ENSRNOT00000047593	chr2	ENSRNOG00000034093	AABR07011951.1	177651241	177653288	+	-0.9704	0.0006	0.0335	31.6371	59.4345	33.4911	6.0435	99.9758	50.6662	51.8463	53.4175
ENSRNOT00000049573	chr20	ENSRNOG00000000729	Tmlhe	248410	284832	+	-Inf	0.0004	0.0241	0	0	0	0	0.5343	0	0	0
ENSRNOT00000050764	chr2	ENSRNOG00000012729	Mfsd8	127708375	127781003	-	-4.0491	0.0000	0.0047	0.0196	0.0443	0.0393	0.0230	1.9828	0.0465	0.0173	0.0409
ENSRNOT00000050914	chr2	ENSRNOG00000021101	Sema6c	196334626	196343987	+	-Inf	0.0000	0.0009	0	0	0	0	0	0.2457	0.3643	0
ENSRNOT00000051135	chrX	ENSRNOG00000003749	Xk	14498119	14534473	+	-2.1325	0.0007	0.0365	0.1547	0.1972	0.1184	0.1715	0.0506	0.2298	0.1249	2.4089
ENSRNOT00000057641	chr8	ENSRNOG00000018184	Tpm1	72814740	72841496	-	-1.1105	0.0005	0.0310	1.6172	1.1545	2.5381	1.3058	2.5340	4.3896	3.5197	3.8414
ENSRNOT00000057724	chr2	ENSRNOG00000009955	Plch1	154256392	154334356	-	-Inf	0.0000	0.0016	0	0	0	0	0.8189	0	0	0
ENSRNOT00000057845	chr16	ENSRNOG00000012573	Dlgap2	79872514	79973735	-	-1.0491	0.0000	0.0038	13.7553	6.5867	7.4514	12.5244	10.4442	26.6911	24.7209	21.5704
ENSRNOT00000061417	chr18	ENSRNOG00000039969	Dsc2	11826706	11858744	-	-Inf	0.0004	0.0241	0	0	0	0	0	0	0	0.1579
ENSRNOT00000064140	chr8	ENSRNOG00000020804	Smarcc1	118206703	118324188	+	-5.2321	0.0000	0.0000	0.1254	0.1804	0.1529	0.1685	0.1799	6.9356	0.1869	16.2675
ENSRNOT00000064184	chr1	ENSRNOG00000020650	Slc17a7	101161375	101172131	+	-1.8433	0.0011	0.0483	0.0625	0.0421	0.1114	0.0666	0.0743	0.5382	0.1417	0.2597
ENSRNOT00000064507	chr3	ENSRNOG00000010551	Lhx2	22640545	22658446	+	-1.2409	0.0000	0.0009	7.4235	5.0092	14.0889	6.7740	18.9477	13.7042	25.5949	20.4478
ENSRNOT00000064755	chr5	ENSRNOG00000008237	Unc13b	58667885	58714247	+	-1.4575	0.0006	0.0323	0.0370	0.3986	0.9929	0.7279	1.5495	1.9491	1.1826	1.2412
ENSRNOT00000064841	chr5	ENSRNOG00000022694	Plekhg5	169244778	169288309	+	-0.6336	0.0005	0.0284	13.4977	9.2705	7.3180	11.0757	13.0018	14.6401	19.2345	16.9853
ENSRNOT00000065294	chr10	ENSRNOG00000005360	Dhrs7b	47081834	47111101	+	-2.3643	0.0001	0.0066	0.6191	0.7062	0.9272	0.5997	0.8442	11.8647	1.1390	0.8375
ENSRNOT00000065373	chr10	ENSRNOG00000003207	Fmnl1	91254088	91281416	+	-0.5913	0.0005	0.0290	15.3226	9.0221	12.9505	10.7683	15.8952	19.6873	18.8503	17.9820
ENSRNOT00000066119	chr13	ENSRNOG00000002947	Dpt	83073544	83102396	+	-9.3854	0.0008	0.0399	0.0002	0	0.0001	0	0.2258	0.0002	0	0.0001
ENSRNOT00000066986	chr3	ENSRNOG00000008746	Dtd1	138770615	138931542	+	-Inf	0.0006	0.0319	0	0	0	0	0	0	0.5537	0
ENSRNOT00000067262	chr18	ENSRNOG00000016675	Kcnn2	39335377	39479257	+	-4.3085	0.0001	0.0087	0.0056	0.0055	0.0545	0.0068	0.0291	0.0133	0.0033	1.3895
ENSRNOT00000067331	chr5	ENSRNOG00000006096	Slc26a7	27989298	28131133	-	-Inf	0.0008	0.0393	0	0	0	0	0.0150	0	0.0932	0
ENSRNOT00000067694	chr14	ENSRNOG00000018144	Depdc5	83089000	83219464	-	-1.0331	0.0007	0.0367	0.4134	0.7919	0.4044	0.7701	0.7322	2.0038	1.3076	0.8265
ENSRNOT00000067780	chr19	ENSRNOG00000042838	Junb	26092974	26094756	-	-0.6664	0.0010	0.0446	43.3974	28.3028	44.3342	13.5088	44.4303	54.3316	56.2168	50.6210
ENSRNOT00000068496	chr9	ENSRNOG00000018366	RGD1310819	44194325	44237117	-	-0.5005	0.0009	0.0432	48.5578	33.7867	31.6182	26.7711	46.1418	54.5740	49.3488	49.0286
ENSRNOT00000070855	chr7	ENSRNOG00000048982	LOC100912282	144308345	144319804	-	-Inf	0.0004	0.0265	0	0	0	0	0	0	0	0.3256
ENSRNOT00000071435	chr10	ENSRNOG00000028845	Ebf1	23914894	24049832	+	-3.7868	0.0000	0.0028	0	0.0101	0.0626	0.0362	0.0333	0.3172	0.2392	0.9129
ENSRNOT00000071634	chrX	ENSRNOG00000052909	AABR07036746.1	1543244	1543849	+	-2.0603	0.0008	0.0403	0.2442	0.4244	0.2092	0.4015	0.6856	0.1268	0.9950	3.5280
ENSRNOT00000071728	chr13	ENSRNOG00000046663	LOC498276	91146876	91228901	-	-2.4796	0.0001	0.0096	0.1373	0.2409	0.1926	0.1969	0.1766	1.8025	0.2107	2.0925
ENSRNOT00000071741	chr1	ENSRNOG00000050206	Shank2	217345545	217589035	+	-9.2136	0.0000	0.0004	0.0010	0.0003	0.0004	0	0.0004	0	0.4288	0.5499

ENSRNOT00000071829	chr17	ENSRNOG00000047594	Heatr1	66579319	66619250	+	-4.5962	0.0000	0.0047	0.0447	0.0230	0.0058	0	0.0094	0	0.7733	0.9936
ENSRNOT00000071891	chr1	ENSRNOG00000050201	Slc29a2	220307394	220314035	+	-Inf	0.0000	0.0001	0	0	0	0	0	0	1.9643	1.9051
ENSRNOT00000072024	chr12	ENSRNOG00000049282	Oas2	41341417	41363161	+	-Inf	0.0007	0.0380	0	0	0	0	0.0299	0.0200	0	0.0069
ENSRNOT00000072402	chr11	ENSRNOG00000046850	Thpo	83868655	83873910	+	-Inf	0.0000	0.0002	0	0	0	0	0	0.4944	0.0582	0.6428
ENSRNOT00000072675	chr2	ENSRNOG00000010556	Intu	127459089	127525396	+	-11.2144	0.0001	0.0106	0	0	0.0003	0	0.7342	0	0	0
ENSRNOT00000073526	chr11	ENSRNOG00000045653	Fam131a	82875627	82884660	-	-0.6614	0.0001	0.0123	27.8912	18.7668	16.5272	18.3126	31.6357	26.2358	36.8721	34.1570
ENSRNOT00000073534	chr17	ENSRNOG00000046647	Impad1	90188043	90217786	-	-5.2034	0.0000	0.0055	0.0199	0.0146	0.0277	0.0333	3.4966	0	0.0079	0.0159
ENSRNOT00000074087	chr17	ENSRNOG00000017941	Optn	77195715	77218374	+	-Inf	0.0001	0.0067	0	0	0	0	0	0.6250	0	0
ENSRNOT00000074469	chr9	ENSRNOG00000047247	Ptprs	10603813	10645939	+	-Inf	0.0000	0.0005	0	0	0	0	2.7724	0	0	0
ENSRNOT00000074490	chr16	ENSRNOG00000045697	Ccdc110	47151520	47163898	-	-Inf	0.0010	0.0450	0	0	0	0	0.1546	0	0	0
ENSRNOT00000074526	chr4	ENSRNOG00000045978	Hist2h4	170144951	170145262	-	-1.3786	0.0001	0.0122	9.6618	11.5115	9.6304	9.5617	14.8395	40.4087	39.4943	10.2139
ENSRNOT00000075906	chr4	ENSRNOG00000010609	Abcf2	7129932	7134361	+	-Inf	0.0003	0.0221	0	0	0	0	0	0	1.0126	0
ENSRNOT00000075998	chr13	ENSRNOG00000026542	Suco	79737985	79801368	-	-Inf	0.0003	0.0235	0	0	0	0	0	0.2905	0	0
ENSRNOT00000076213	chr8	ENSRNOG00000005934	Mlip	84434784	84522588	-	-0.5962	0.0006	0.0338	22.9558	13.7535	16.6805	16.9107	22.3416	31.5748	29.3740	22.9813
ENSRNOT00000076248	chr9	ENSRNOG00000022812	Ercc5	50925619	50955882	+	-1.0720	0.0008	0.0406	1.0449	1.3872	1.8993	1.0632	4.9157	2.5879	1.4023	2.4353
ENSRNOT00000076266	chr15	ENSRNOG00000051196	AABR07018038.1	36898195	36911165	+	-3.3176	0.0000	0.0029	0.1283	0.1058	0.0824	0.0630	1.4848	0.0253	0.3250	1.9493
ENSRNOT00000076339	chr4	ENSRNOG00000010609	Abcf2	7122890	7135669	+	-0.7990	0.0004	0.0271	8.8420	8.1495	10.2910	4.7551	20.5912	9.5320	16.0920	9.5276
ENSRNOT00000076418	chr4	ENSRNOG00000010609	Abcf2	7122906	7135606	+	-1.2343	0.0000	0.0048	5.8933	5.1843	3.9442	6.0583	11.3152	21.7881	12.5240	3.9679
ENSRNOT00000076467	chr5	ENSRNOG00000033527	Pappa1	80920568	81150929	+	-Inf	0.0000	0.0062	0	0	0	0	0.3441	0	0	0
ENSRNOT00000076618	chr10	ENSRNOG00000021412	Slfn13	70326157	70339578	-	-6.7834	0.0000	0.0009	0.0018	0	0.0033	0.0007	0.3549	0.2891	0.0007	0.0007
ENSRNOT00000076998	chr7	ENSRNOG00000043465	Arc	115907097	115910522	-	-0.9914	0.0006	0.0340	40.2234	10.3098	52.9902	7.3922	45.1361	63.6150	55.8809	55.8740
ENSRNOT00000077136	KL568097.1	ENSRNOG00000061540	LOC100909715	52128	52865	-	-3.4814	0.0000	0.0005	0.6296	0.4308	0.1602	0.2234	0.3292	5.8529	1.2114	8.7335
ENSRNOT00000077741	chr3	ENSRNOG00000057376	AABR07051548.2	16753703	16754189	+	-5.8483	0.0004	0.0271	0.2571	0	0	0	0	0	0	14.8130
ENSRNOT00000077758	chr15	ENSRNOG00000038483	AABR07018323.1	51434096	51463158	+	-Inf	0.0005	0.0311	0	0	0	0	0.0048	0.0401	0	0.1179
ENSRNOT00000077912	chr7	ENSRNOG00000029651	LOC100365958	70980422	71072072	+	-Inf	0.0008	0.0414	0	0	0	0	0	0	0.4166	0
ENSRNOT00000078032	chr18	ENSRNOG00000002697	Mtmr1	291780	325374	-	-0.6222	0.0004	0.0248	11.7939	8.0688	10.3090	7.9581	15.2723	12.7845	15.3186	15.3132
ENSRNOT00000078477	chr1	ENSRNOG00000014530	Nav2	104635989	104940171	+	-1.0152	0.0001	0.0110	0.9187	1.2977	0.8626	0.6393	1.7545	2.5448	1.9948	1.2212
ENSRNOT00000078530	chr12	ENSRNOG00000001247	Clip1	38375416	38451226	+	-9.0327	0.0001	0.0111	0	0.0005	0.0004	0	0	0.4946	0.0003	0

ENSRNOT00000078548	chr6	ENSRNOG00000006199	Ispd	55881387	56055489	+	-4.7445	0.0000	0.0000	0.0673	0.0612	0.1009	0.0697	2.6751	0.1831	2.3756	2.7837
ENSRNOT00000078640	chr1	ENSRNOG00000054459	Mboat7	64101018	64113792	+	-Inf	0.0000	0.0000	0	0	0	0	25.0837	25.2992	19.9668	20.4576
ENSRNOT00000078683	chr12	ENSRNOG00000001274	Vps29	39690397	39697465	-	-2.9132	0.0001	0.0132	0.1401	0.1232	0.1664	0.1086	0.0998	0.2623	3.5598	0.1332
ENSRNOT00000079116	chr13	ENSRNOG00000003120	Prelp	50749514	50755046	-	-4.3104	0.0000	0.0002	0.1249	0.0472	0.2448	0.0267	3.9476	4.6282	0.0719	0.1539
ENSRNOT00000079219	chr1	ENSRNOG00000014276	Plce1	257157312	257466064	+	-1.7875	0.0009	0.0439	0.0637	0.0973	0.1048	0.0601	0.0465	0.0871	0.8874	0.1040
ENSRNOT00000079732	chr5	ENSRNOG00000013865	Zmym6	145145983	145186357	+	-2.1617	0.0012	0.0497	0.0727	0.0285	0.0661	0.0270	0.0560	0.7311	0.0461	0.0360
ENSRNOT00000079838	chr20	ENSRNOG00000040052	RT1-M6-2	2076583	2081885	+	-Inf	0.0002	0.0164	0	0	0	0	0	0	0	1.0005
ENSRNOT00000080108	chr5	ENSRNOG00000061876	Aldh4a1	158069725	158128523	+	-2.7772	0.0005	0.0311	0.2265	0	0	0.3940	0.6390	0.4367	0.4866	2.6917
ENSRNOT00000080188	chr10	ENSRNOG00000036698	Nploc4	109555241	109604899	-	-8.9976	0.0000	0.0000	0	0.0203	0.0434	0.0485	0.0347	28.9715	28.3464	0
ENSRNOT00000080190	chr3	ENSRNOG00000047466	Bdnf	100786862	100819210	+	-1.0349	0.0003	0.0198	1.4361	1.2822	1.9068	0.7852	2.6198	3.5527	2.1296	2.7829
ENSRNOT00000080466	chr1	ENSRNOG00000059741	Tmc4	64114721	64126415	+	-3.2121	0.0010	0.0453	0	0	0.0179	0	0.0046	0.0620	0.0502	0.0494
ENSRNOT00000080506	chr1	ENSRNOG00000028288	Clrn3	207750223	207811008	-	-Inf	0.0003	0.0228	0	0	0	0	0	0.0321	0.0244	0.0237
ENSRNOT00000080512	chrX	ENSRNOG00000007014	Cnksr2	39711201	39942544	+	-0.5618	0.0009	0.0441	31.8947	14.8735	18.4005	18.0264	25.9166	34.7604	33.0331	29.0970
ENSRNOT00000080872	chr1	ENSRNOG00000020039	Slc5a2	199682693	199688573	+	-Inf	0.0000	0.0011	0	0	0	0	0.1353	0	0.0555	0.1321
ENSRNOT00000080966	chr2	ENSRNOG00000021024	Pi4kb	196145384	196171573	+	-1.0718	0.0001	0.0106	5.5877	2.3813	3.4805	5.5878	11.6941	5.7279	13.0122	5.3787
ENSRNOT00000081280	chr10	ENSRNOG00000006557	Cyfip2	31278764	31359699	-	-0.9843	0.0000	0.0049	65.0844	73.9014	223.6262	210.1341	279.6830	315.8693	274.4416	263.1125
ENSRNOT00000081542	AABR07046231.1	ENSRNOG00000059312	Sfxn2	24692	31220	+	-4.6016	0.0000	0.0001	0.0278	0	0	0.0064	0.1353	0.2751	0.1827	0.2372
ENSRNOT00000081570	chr14	ENSRNOG00000030568	Rgs12	80982162	81023682	-	-0.7833	0.0001	0.0133	10.4668	8.9159	5.6791	6.5905	13.1306	18.0523	11.0185	12.2748
ENSRNOT00000081838	chr16	ENSRNOG00000055604	LOC100911229	68633720	68635522	+	-Inf	0.0002	0.0140	0	0	0	0	0.0080	0.0939	0.0329	0
ENSRNOT00000081986	chrX	ENSRNOG00000052455	LOC100910245	29157470	29176539	+	-Inf	0.0000	0.0002	0	0	0	0	2.6935	0	2.7636	0
ENSRNOT00000082017	chr6	ENSRNOG00000040287	Cyp1b1	2310988	2316717	-	-7.8102	0.0007	0.0377	0	0	0.0003	0.0004	0.0992	0.0239	0	0.0311
ENSRNOT00000082286	chr6	ENSRNOG00000051077	Brms1l	76675437	76753950	+	-1.8581	0.0007	0.0362	0.1078	0.1158	0.2251	0.0980	1.4697	0.1740	0.1148	0.2233
ENSRNOT00000082310	chr1	ENSRNOG00000060599	Gabrb3	113033098	113033228	+	-2.3774	0.0011	0.0478	0.0875	0.1197	0.2326	0.1002	0.7468	0.7614	0.7429	0.5547
ENSRNOT00000082401	chr8	ENSRNOG00000010634	Megf11	70112925	70178561	+	-4.4364	0.0001	0.0121	0.0030	0.0091	0.0011	0.0113	0.0102	0.5028	0.0087	0.0104
ENSRNOT00000082489	chr3	ENSRNOG00000013694	Ntng2	7742723	7796385	-	-0.5952	0.0008	0.0401	13.9695	16.1728	10.2452	16.2497	19.4939	25.6686	23.2465	17.1489
ENSRNOT00000083383	chr6	ENSRNOG00000004908	Smc6	36941596	36995138	+	-2.0526	0.0000	0.0000	2.9435	2.1959	1.8700	2.3922	11.8178	12.3791	2.6716	12.1344
ENSRNOT00000083943	chr16	ENSRNOG00000056947	AABR07025272.1	31257230	31301880	-	-Inf	0.0009	0.0428	0	0	0	0	0.0083	0.0645	0	0.0246
ENSRNOT00000084454	chr1	ENSRNOG00000017030	Arid1b	45923222	46232119	+	-1.1086	0.0009	0.0431	1.0786	1.1766	1.0361	1.0965	1.3498	1.1684	1.1899	5.7538

ENSRNOT00000084471	chr15	ENSRNOG00000060775	Lmo7	86243148	86457739	+	-0.4866	0.0010	0.0456	17.0791	15.2114	11.8348	11.8795	19.4436	20.5088	20.0656	18.4502
ENSRNOT00000084588	chr4	ENSRNOG00000010253	Cd163	156752082	156784869	+	-6.8924	0.0000	0.0016	0.0015	0.0019	0.0023	0.0013	0.3210	0	0	0.5227
ENSRNOT00000084720	chr20	ENSRNOG00000059663	Rufy2	27181195	27208041	-	-10.4450	0.0000	0.0000	0	0	0	0.0057	4.9016	0	0.0637	3.0225
ENSRNOT00000084875	chr7	ENSRNOG00000005233	Lrrc6	107189782	107223047	-	-Inf	0.0002	0.0139	0	0	0	0	0.1797	0	0.2986	0
ENSRNOT00000084881	KL568483.1	ENSRNOG00000058500	LOC103694908	1101	9649	-	-Inf	0.0001	0.0118	0	0	0	0	0.2394	0	0.0158	0.1136
ENSRNOT00000085132	chr3	ENSRNOG00000008316	Vps39	112087796	112125318	-	-1.1195	0.0004	0.0273	2.6545	3.2113	3.4288	12.2765	3.6606	14.5299	14.2662	14.4110
ENSRNOT00000085195	chr9	ENSRNOG00000013484	LOC108348061	27368272	27447877	-	-Inf	0.0000	0.0000	0	0	0	0	11.0123	6.4508	0	9.9056
ENSRNOT00000085380	chr20	ENSRNOG00000057125	Ddr1	3555135	3574959	+	-6.3911	0.0004	0.0269	0	0.0026	0.0017	0.0005	0	0.0067	0.3993	0
ENSRNOT00000085632	chr2	ENSRNOG00000042717	Ciart	197987742	197991574	-	-5.6169	0.0001	0.0067	0.0169	0	0	0.0121	0	0.0478	1.3636	0.0110
ENSRNOT00000085754	chr11	ENSRNOG00000042289	Plcxd2	57207679	57260568	+	-2.6666	0.0010	0.0451	0	0.0487	0.0425	0.0319	0.6618	0.0313	0.0422	0.0464
ENSRNOT00000085808	chr8	ENSRNOG00000030183	Plod2	99977334	100059728	+	-2.3681	0.0001	0.0108	0.1230	0.3338	0	0.2886	0.9480	0.8101	1.0184	1.0720
ENSRNOT00000085894	chr8	ENSRNOG00000018184	Tpm1	72814740	72841496	-	-1.0091	0.0004	0.0244	3.5969	1.7556	2.8553	4.1237	6.4006	6.8711	4.6402	6.9070
ENSRNOT00000085987	chr20	ENSRNOG00000000596	Fyn	44521279	44630294	+	-Inf	0.0000	0.0001	0	0	0	0	0	0	0	15.0701
ENSRNOT00000086078	chr12	ENSRNOG00000022893	Rimbp2	31536951	31620760	+	-0.8366	0.0001	0.0133	3.2826	2.6816	2.5682	3.3348	2.9336	4.1595	6.5725	7.5279
ENSRNOT00000086185	chr8	ENSRNOG00000057676	Polr2m	77986094	78096302	-	-Inf	0.0012	0.0494	0	0	0	0	0	0.0690	0.0251	0
ENSRNOT00000086279	chr2	ENSRNOG00000016394	Wdr77	208420309	208587260	+	-6.4772	0.0000	0.0002	0.0350	0	0.0295	0.0129	0.0489	0.0337	0.0356	6.7686
ENSRNOT00000086397	chr12	ENSRNOG00000059456	Grk3	49665282	49746162	+	-Inf	0.0000	0.0002	0	0	0	0	0	0	0	13.8197
ENSRNOT00000086745	chr3	ENSRNOG00000008663	Nat10	93658657	93693690	-	-1.5838	0.0001	0.0098	0.7824	0.7963	1.2722	0.6856	0.7815	1.2000	4.0691	4.5500
ENSRNOT00000087174	KL568024.1	ENSRNOG00000052587	Fam43a	22	1354	-	-1.7530	0.0005	0.0301	0.0675	0.3835	0.1966	0.1270	0.7260	0.6583	0.6870	0.5393
ENSRNOT00000087177	chr7	ENSRNOG00000037687	Rspo2	81924050	82059247	-	-0.8865	0.0005	0.0300	6.9717	3.9608	2.4086	4.5227	5.7990	6.2365	10.1358	10.8528
ENSRNOT00000087289	chr3	ENSRNOG00000057817	LOC100911769	152571121	152599512	+	-Inf	0.0003	0.0201	0	0	0	0	0	0	0	0.3287
ENSRNOT00000087344	chr7	ENSRNOG00000012295	Pla2g6	120519467	120558805	-	-7.6692	0.0000	0.0005	0.0043	0.0042	0.0007	0.0029	0.0033	0.0011	0.0025	2.4438
ENSRNOT00000087504	chr13	ENSRNOG00000007887	Elk4	48790767	48804922	+	-4.4049	0.0002	0.0151	0.7342	0	0.0177	0	0.6377	7.2029	0	8.0874
ENSRNOT00000087753	chr8	ENSRNOG00000029910	Golga4	127171636	127248538	+	-1.1608	0.0006	0.0352	0.4479	1.0043	1.1857	1.2263	1.3919	5.1304	1.1468	0.9702
ENSRNOT00000088065	chr1	ENSRNOG00000060994	Tctn3	259682249	259691742	-	-Inf	0.0000	0.0001	0	0	0	0	0	3.2741	0.6010	0
ENSRNOT00000088148	chr1	ENSRNOG00000047104	LOC100911660	257766691	257949180	+	-Inf	0.0000	0.0002	0	0	0	0	0	0	0.7286	0.7146
ENSRNOT00000088297	chr19	ENSRNOG00000021517	Tmem231	44137444	44158624	-	-1.6059	0.0006	0.0351	0.2423	0.4378	0.4357	5.0063	5.0374	3.7499	4.9929	4.8546
ENSRNOT00000088304	chr8	ENSRNOG00000013610	Chrna5	59561817	59590172	+	-Inf	0.0000	0.0015	0	0	0	0	0	2.3722	0	0

ENSRNOT00000088381	chr12	ENSRNOG00000037446	Pxmp2	52452331	52462659	+	-Inf	0.0002	0.0139	0	0	0	0	0.2368	0	0.4059	0
ENSRNOT00000088441	chr14	ENSRNOG00000002278	Tec	37918652	38027920	+	-3.4324	0.0008	0.0414	0.0326	0.0160	0.0087	0	0.0405	0.0051	0.5724	0
ENSRNOT00000088624	chr18	ENSRNOG00000017610	Nedd4l	60496778	60718471	+	-Inf	0.0000	0.0000	0	0	0	0	0	16.8411	28.4505	27.9173
ENSRNOT00000088674	chr14	ENSRNOG00000002855	Rfc1	44580216	44689001	+	-Inf	0.0000	0.0014	0	0	0	0	2.4638	0	0	0
ENSRNOT00000088905	chr12	ENSRNOG00000001274	Vps29	39690237	39699088	-	-Inf	0.0000	0.0003	0	0	0	0	0	0.0018	10.9609	0
ENSRNOT00000088936	chr20	ENSRNOG00000000838	Lta	4852496	4854294	+	-Inf	0.0009	0.0438	0	0	0	0	0.0300	0.0216	0.0576	0
ENSRNOT00000089060	chr7	ENSRNOG00000059605	Ddn	140479711	140483693	-	-0.5296	0.0003	0.0226	296.6284	228.5071	175.5612	207.8975	290.0889	324.1024	372.8899	324.4932
ENSRNOT00000089196	chr1	ENSRNOG00000015036	Ctgf	21851660	21854763	-	-2.9825	0.0000	0.0020	1.0503	0.1649	1.4647	0.4064	9.5259	1.4958	0.1212	13.2501
ENSRNOT00000089290	chr7	ENSRNOG00000048982	LOC100912282	144307509	144322240	-	-10.7982	0.0000	0.0002	0.0058	0	0	0	0	0.0596	0	10.3555
ENSRNOT00000089306	chr20	ENSRNOG00000038999	RT1-A1	5351605	5418011	+	-Inf	0.0002	0.0142	0	0	0	0	0.6562	0	0	0
ENSRNOT00000089339	chr2	ENSRNOG00000016550	Dclk2	186117778	186245163	-	-Inf	0.0000	0.0000	0	0	0	0	0.7080	0	1.2019	0.7261
ENSRNOT00000089417	chr20	ENSRNOG00000057125	Ddr1	3556975	3575780	+	-1.7911	0.0000	0.0012	2.6722	5.5574	5.0551	5.8878	30.6841	2.2281	10.4600	22.9785
ENSRNOT00000089434	chr3	ENSRNOG00000007925	Pak6	110466790	110487526	+	-0.9376	0.0001	0.0072	13.3065	5.4513	8.3920	5.0425	13.1874	15.4210	20.1478	12.9031
ENSRNOT00000089897	chr4	ENSRNOG00000005775	Phf14	38255413	38420205	+	-4.7328	0.0000	0.0002	0.1364	0.1557	0.2043	0.1853	17.6528	0.1457	0.1508	0.1761
ENSRNOT00000090502	KL568417.1	ENSRNOG00000053118	LOC103694867	15968	35620	+	-6.3741	0.0000	0.0028	0	0	0.0122	0.0833	3.8349	4.0844	0	0
ENSRNOT00000090848	chr13	ENSRNOG00000004018	Tdrd5	73872621	73921969	-	-4.7098	0.0001	0.0075	0	0	0	0.0191	0.0252	0	0.2344	0.2408
ENSRNOT00000091046	chr17	ENSRNOG00000000105	Cplx2	10756285	10818835	-	-0.5137	0.0004	0.0247	274.5070	206.9550	178.3610	193.2545	302.1818	335.8139	318.7545	261.2285
ENSRNOT00000091048	chr3	ENSRNOG00000049056	AABR07051450.1	11653529	11657768	+	-Inf	0.0000	0.0014	0	0	0	0	0	0	0	9.4766
ENSRNOT00000091122	chr10	ENSRNOG00000058393	Taf15	70689863	70721892	+	-Inf	0.0004	0.0241	0	0	0	0	0.4671	0	0	0
ENSRNOT00000091215	chr9	ENSRNOG00000054698	AABR07066792.1	16347795	16351503	-	-Inf	0.0000	0.0024	0	0	0	0	0.0801	0.1391	0	0.2208
ENSRNOT00000091242	chr10	ENSRNOG00000047933	LOC103690164	37032056	37051519	+	-Inf	0.0001	0.0100	0	0	0	0	0	0.9483	0	0
ENSRNOT00000091263	chr6	ENSRNOG00000006867	Etv1	58468155	58553701	+	-1.0638	0.0011	0.0493	4.2813	6.8509	4.1317	21.1292	25.0131	15.2708	6.1830	29.6113
ENSRNOT00000091283	chr9	ENSRNOG00000016486	Nop58	66495840	66519788	+	-2.4691	0.0011	0.0475	0.0402	0.0666	0.0336	0.3612	0.0224	0.0530	1.5427	1.1593
ENSRNOT00000091560	chr6	ENSRNOG00000056756	Actn1	103375799	103470427	-	-0.7548	0.0011	0.0492	14.3170	8.0028	8.7850	6.6153	11.1362	30.3959	11.6161	10.5001
ENSRNOT00000091610	chr10	ENSRNOG00000046271	LOC100911685	4312863	4446200	+	-1.2229	0.0004	0.0279	3.1677	2.9336	3.3121	3.0215	2.8878	3.2279	3.8364	19.0727
ENSRNOT00000091680	chr1	ENSRNOG00000018708	Ppp1ca	219440192	219443564	+	-1.6389	0.0001	0.0065	1.3944	5.5427	0.8584	6.1057	9.0081	14.3839	14.3262	5.5755
ENSRNOT00000091760	chr1	ENSRNOG00000020014	Myh14	100608966	100669684	-	-1.3969	0.0000	0.0006	1.8448	1.1945	1.7824	1.5025	1.8881	5.8526	3.2567	5.6562
ENSRNOT00000092269	chr13	ENSRNOG00000027962	Smg7	70260865	70321752	-	-3.2767	0.0010	0.0449	0.0103	0.0020	0.0021	0.0174	0.2952	0.0015	0.0028	0.0089

ENSRNOT00000092327	chr1	ENSRNOG00000019207	Shank1	100299626	100343976	+	-0.5030	0.0009	0.0431	52.2217	30.4878	33.8988	28.3711	44.4066	54.6435	54.9095	51.4945
ENSRNOT00000092357	chr1	ENSRNOG00000050206	Shank2	217459321	217589488	+	-5.8216	0.0000	0.0022	0	0	0.1886	0	4.8175	0	2.9062	2.9434
ENSRNOT00000092649	chr13	ENSRNOG00000027962	Smg7	70266013	70321750	-	-Inf	0.0009	0.0433	0	0	0	0	0.1824	0	0	0
ENSRNOT00000092802	chr13	ENSRNOG00000025701	Nifk	34267614	34273463	+	-4.2963	0.0000	0.0018	0.0222	0.0901	0.0302	0.0683	0.9874	0.0701	2.9837	0.0995
ENSRNOT00000092916	chr1	ENSRNOG00000047280	Cttn	217602698	217638152	-	-0.7400	0.0010	0.0461	2.4263	4.8495	4.7653	4.6943	9.4075	4.9185	5.7634	7.8617
ENSRNOT00000092976	chr1	ENSRNOG00000017030	Arid1b	45923222	46230523	+	-Inf	0.0000	0.0016	0	0	0	0	0	0	0	1.3513
ENSRNOT00000093107	chr8	ENSRNOG00000013610	Chrna5	59561721	59589144	+	-5.2077	0.0000	0.0042	0.0137	0.0219	0.0086	0.0294	0.0147	2.6451	0	0.0623
ENSRNOT00000093127	chr13	ENSRNOG00000002376	Clasp1	34498749	34584651	+	-3.6739	0.0001	0.0090	1.6470	0	0.4450	0	0.8046	11.4743	10.4785	3.9433
ENSRNOT00000093241	chr6	ENSRNOG00000004067	Nrcam	64790112	64863954	+	-0.4452	0.0009	0.0426	58.5111	49.0008	41.1815	45.0319	58.1263	66.5677	69.1944	69.8755
ENSRNOT00000093392	chr19	ENSRNOG00000011976	Nudt7	46455859	46482050	+	-Inf	0.0001	0.0068	0	0	0	0	0.3625	0	0	1.3011
ENSRNOT00000093571	chr15	ENSRNOG00000059334	Fbxl3	93647310	93667355	-	-Inf	0.0000	0.0002	0	0	0	0	0	15.5752	0	0
ENSRNOT00000093730	chr9	ENSRNOG00000015633	Cul3	86046187	86129073	-	-7.3320	0.0000	0.0013	0	0	0.0095	0	0	0	0.5535	0.9831
ENSRNOT00000000026	chr4	ENSRNOG00000010388	Slc21a4	176255603	176295050	-	Inf	0.0001	0.0135	0.0109	0.0130	0.0245	0.0079	0	0	0	0
ENSRNOT00000000133	chr5	ENSRNOG00000000121	Pigv	151886136	151898022	-	4.8451	0.0000	0.0002	0.1084	2.0180	2.0295	0.0220	0.0192	0.0232	0.0309	0.0719
ENSRNOT00000001527	chr20	ENSRNOG00000001152	Glp1r	9586075	9626228	+	2.4395	0.0002	0.0144	0.0955	0.3619	0.1409	0.5936	0.1128	0.0620	0.0096	0.0353
ENSRNOT00000001642	chr20	ENSRNOG00000001227	Adarb1	11972381	12101021	+	0.6968	0.0006	0.0350	23.5811	39.6019	64.9809	55.3870	34.6539	26.7874	21.9909	29.8046
ENSRNOT00000001667	chr12	ENSRNOG00000001241	Iqce	15857805	16067921	+	5.4925	0.0002	0.0162	1.0921	0	0.0135	0.0133	0	0.0249	0	0
ENSRNOT00000001686	chr12	ENSRNOG00000001251	Grifin	16170162	16172129	+	2.6105	0.0003	0.0210	0.2733	1.9910	0.9293	4.2292	0.4960	0	0.4293	0.2901
ENSRNOT00000001730	chr12	ENSRNOG00000001284	Uncx	17182163	17186679	-	4.2290	0.0002	0.0137	0	1.2060	0.0890	0.7506	0.0970	0	0	0.0121
ENSRNOT00000001905	chr12	ENSRNOG00000001407	Tfr2	22177382	22194299	-	Inf	0.0001	0.0125	0	0	0	0.3982	0	0	0	0
ENSRNOT00000002010	chr12	ENSRNOG00000001478	Gtf2ird1	25305823	25370780	+	2.9225	0.0005	0.0304	2.5361	4.6151	0.8440	0.8668	0.2089	0.9600	0	0
ENSRNOT00000002558	chr11	ENSRNOG00000001873	P2rx6	87435185	87445219	+	1.4734	0.0006	0.0324	0.6466	2.5272	4.4627	2.9708	1.2138	0.3151	1.3355	0.9557
ENSRNOT00000002887	chr14	ENSRNOG00000002117	Barhl2	4362717	4367605	+	2.2587	0.0011	0.0490	0.0389	1.6812	0.6892	1.8115	0.6415	0.0249	0.1419	0.0736
ENSRNOT00000002984	chr14	ENSRNOG00000002192	Rell1	45982311	46030937	+	4.2849	0.0001	0.0119	0.0203	0.0254	0.0370	1.5237	0.0387	0.0041	0.0396	0
ENSRNOT00000003082	chr14	ENSRNOG00000022242	Cxcl9	17228856	17234712	+	2.9565	0.0009	0.0434	0.0406	0.0644	0.3125	0.0208	0	0	0.0198	0.0367
ENSRNOT00000003638	chr18	ENSRNOG00000002697	Mtmr1	291785	325377	-	2.2774	0.0001	0.0089	4.7070	4.5596	3.7779	5.0330	0.3163	2.9894	0.3875	0.0356
ENSRNOT00000003895	chrX	ENSRNOG00000002862	Clcn5	16170576	16196691	+	1.1934	0.0002	0.0152	2.3542	7.1206	4.3576	5.9182	2.9722	1.8998	1.7439	2.0205
ENSRNOT00000004153	chr14	ENSRNOG00000003067	Npffr2	20148913	20193083	-	4.1858	0.0004	0.0265	0	0.2102	0.4935	0.1166	0.0451	0	0	0

ENSRNOT00000004184	chrX	ENSRNOG00000003081	Ribc1	21699368	21710678	-	3.4883	0.0002	0.0187	0.1901	0.0620	0.1946	0.0293	0.0007	0	0.0335	0.0082
ENSRNOT00000004279	chr13	ENSRNOG00000003171	Mpz	89524329	89530068	+	3.0553	0.0001	0.0081	0.2922	4.6773	0.1770	0.1341	0.2173	0.2615	0.0714	0.0849
ENSRNOT00000004385	chr14	ENSRNOG00000003253	Qdpr	70164650	70178284	+	0.6551	0.0003	0.0213	111.3807	159.2802	194.4291	184.6068	128.6748	84.8159	105.5583	93.5225
ENSRNOT00000004717	chr10	ENSRNOG00000003476	Slc6a4	63153651	63176463	-	4.1239	0.0000	0.0012	0.0370	0.1249	0.1448	2.6053	0.0881	0.0118	0.0496	0.0175
ENSRNOT00000005157	chr7	ENSRNOG00000003880	Tph2	58042281	58149061	-	2.1838	0.0001	0.0135	0.5523	0.4134	0.4640	5.2279	0.3774	0.5190	0.2574	0.3114
ENSRNOT00000005176	chr10	ENSRNOG00000003845	Wnt3	91830654	91874793	+	1.8216	0.0002	0.0179	0.2726	1.2529	2.6273	2.1430	0.7651	0.2109	0.2681	0.5370
ENSRNOT00000005545	chr7	ENSRNOG00000004013	Grip1	64768742	64864404	+	Inf	0.0001	0.0073	0	0	0.6228	0	0	0	0	0
ENSRNOT00000005988	chr6	ENSRNOG00000004498	Scin	59976003	60054279	-	Inf	0.0004	0.0271	0	0.0143	0.0495	0.0145	0	0	0	0
ENSRNOT00000006128	chr6	ENSRNOG00000004359	Wars	132771021	132797675	-	10.9281	0.0000	0.0001	0.0002	0.0009	0.0008	8.8419	0.0014	0.0010	0.0008	0.0013
ENSRNOT00000006174	chr7	ENSRNOG00000004632	Pmch	28654733	28657522	+	6.1275	0.0000	0.0000	0.1361	102.0805	1.4782	3.0978	0.9520	0.2096	0.2108	0.1552
ENSRNOT00000006238	chr10	ENSRNOG00000004713	Kcnj16	99388130	99389898	+	1.2990	0.0007	0.0385	2.9053	22.9026	11.8054	19.5277	9.6660	1.3882	4.4686	7.7004
ENSRNOT00000006642	chr14	ENSRNOG00000004670	Ddx56	86379010	86387608	-	1.8166	0.0001	0.0125	1.4934	0.9157	3.2968	2.8964	0.7866	0.8244	0.6690	0.1620
ENSRNOT00000007073	chr6	ENSRNOG00000004908	Smc6	36941233	36994465	+	1.1541	0.0001	0.0089	15.5149	14.1439	16.5976	14.7868	3.9439	3.9736	15.2868	4.2250
ENSRNOT00000007091	chr7	ENSRNOG00000005332	Csdc2	123168811	123183335	+	0.7240	0.0006	0.0346	19.0006	35.7593	33.6702	56.3434	24.4453	20.9528	21.3459	20.9015
ENSRNOT00000007109	chr6	ENSRNOG00000005343	Esr2	99164875	99204590	-	Inf	0.0000	0.0046	0	0.0177	0.1941	0.0984	0	0	0	0
ENSRNOT00000007602	chr6	ENSRNOG00000005776	Bcl11b	131836674	131914028	-	2.7835	0.0003	0.0230	0.0385	1.9109	0.2675	0.2043	0.0229	0.0415	0.2739	0.0133
ENSRNOT00000007999	chr7	ENSRNOG00000005333	Azin1	77345646	77372398	-	2.4313	0.0002	0.0177	0.1135	1.1753	0.1359	0.0511	0.0619	0.0627	0.0534	0.0956
ENSRNOT00000008342	chr4	ENSRNOG00000005697	Slc6a11	146106386	146223228	+	1.1486	0.0000	0.0044	32.3661	141.8146	73.9089	131.0574	57.7993	32.6281	36.5473	44.0425
ENSRNOT00000008358	chr4	ENSRNOG00000006383	Atoh1	95498003	95500089	+	Inf	0.0009	0.0432	0.0146	0.0726	0.0030	0	0	0	0	0
ENSRNOT00000008386	chr3	ENSRNOG00000006395	Tldc2	153197644	153207816	+	Inf	0.0001	0.0077	0.0481	0.0975	0.0287	0.0877	0	0	0	0
ENSRNOT00000009146	chr5	ENSRNOG00000006971	AABR07047593.1	38922527	38923095	-	Inf	0.0001	0.0123	0.0727	0.0631	0.0995	0.0332	0	0	0	0
ENSRNOT00000009377	chr6	ENSRNOG00000006178	Dync1h1	134958973	135022584	+	1.9261	0.0000	0.0000	10.2405	47.3414	10.8408	55.8230	7.9402	7.8683	7.9105	8.9759
ENSRNOT00000009817	chr3	ENSRNOG00000007457	Serping1	72161189	72171078	-	1.4969	0.0002	0.0185	5.4745	2.8627	15.9169	3.1564	2.2302	1.0844	1.9384	4.4592
ENSRNOT00000009883	chr4	ENSRNOG00000007270	Il12rb2	98052842	98141482	-	1.8148	0.0003	0.0232	0.2294	0.3000	0.9391	0.8967	0.2356	0.1648	0.1309	0.1410
ENSRNOT00000009940	chr6	ENSRNOG00000009284	Foxa1	78545804	78549669	-	5.2101	0.0000	0.0058	0	1.0756	0.0980	0.1534	0.0359	0	0	0
ENSRNOT00000010019	chr4	ENSRNOG00000007608	Fezf1	50309281	50312608	-	4.2005	0.0004	0.0275	0.0502	2.7438	0.1265	0	0.1427	0.0162	0	0
ENSRNOT00000010126	chr5	ENSRNOG00000007683	Prdm13	35823983	35831712	-	Inf	0.0000	0.0037	0.0133	0.1867	0.0715	0	0	0	0	0
ENSRNOT00000010151	chr4	ENSRNOG00000007544	Il23r	98203958	98305173	-	4.6370	0.0001	0.0115	0	0.0967	0.1405	0.1162	0.0142	0	0	0

ENSRNOT00000010183	chr5	ENSRNOG00000007574	Padi2	159428515	159471144	+	0.5999	0.0010	0.0462	15.7361	26.5402	30.4329	23.7052	18.4310	12.6729	16.9610	15.5489
ENSRNOT00000010211	chr5	ENSRNOG00000007551	Patj	117052260	117340336	+	0.9387	0.0002	0.0184	2.3045	5.2152	5.8547	6.5561	3.5222	1.8926	2.1326	2.8503
ENSRNOT00000010263	chr4	ENSRNOG00000007808	Nap1l5	89149317	89151184	-	0.7909	0.0002	0.0168	96.1728	247.4769	133.1545	237.5368	126.7941	89.6735	94.6809	101.7382
ENSRNOT00000010579	chr4	ENSRNOG00000007743	Mgst1	172119331	172134607	+	1.1169	0.0009	0.0421	3.9687	10.1812	8.6042	7.6293	4.7468	2.3789	2.6484	4.2351
ENSRNOT00000010991	chr8	ENSRNOG00000007926	Map2k5	68055977	68282590	-	Inf	0.0000	0.0003	0.6099	0	0	0.8599	0	0	0	0
ENSRNOT00000011327	chr4	ENSRNOG00000008465	Tmem176b	78450724	78458179	-	0.8149	0.0011	0.0493	15.2482	14.8439	23.6470	10.5554	9.6073	10.1198	6.8694	9.9524
ENSRNOT00000011359	chr4	ENSRNOG00000008594	Tex47	23714752	23718047	-	Inf	0.0011	0.0492	0.0223	0.0125	0.0101	0	0	0	0	0
ENSRNOT00000011641	chr2	ENSRNOG00000008765	Zfhx4	98423569	98610368	-	0.8523	0.0000	0.0060	4.7060	6.4885	5.3423	6.9804	3.5264	4.1893	2.2644	3.0461
ENSRNOT00000011794	chr3	ENSRNOG00000008859	Tank	47439076	47515700	+	Inf	0.0001	0.0098	0	0.0081	0	0.4576	0	0	0	0
ENSRNOT00000011849	chr7	ENSRNOG00000008857	Adamts10	18409178	18425692	+	2.3165	0.0003	0.0229	0.0877	0.6804	0.5015	0.0919	0.0717	0.0454	0.0359	0.1203
ENSRNOT00000011983	chr1	ENSRNOG00000008890	Slc18a2	280423079	280457148	+	3.6221	0.0000	0.0000	0.4802	8.5090	1.5964	4.0726	0.5676	0.1428	0.2109	0.2692
ENSRNOT00000011998	chr15	ENSRNOG00000009074	AABR07017902.1	32763067	32777733	+	Inf	0.0000	0.0036	0.0779	0.0293	0.0523	0.0389	0	0	0	0
ENSRNOT00000011999	chr13	ENSRNOG00000009068	Phlda3	52588917	52592001	+	0.8988	0.0010	0.0457	6.5660	19.3759	11.2794	20.0288	9.8320	5.5712	6.8009	8.5020
ENSRNOT00000012022	chr3	ENSRNOG00000008825	Ssrp1	72447969	72457615	+	1.1394	0.0007	0.0361	13.4880	14.1976	3.1272	2.9472	3.4866	5.2566	3.8196	2.7620
ENSRNOT00000012461	chr7	ENSRNOG00000009336	Mapk15	117055623	117060957	+	1.5990	0.0011	0.0493	0.2498	1.0252	1.2736	0.7745	0.2267	0.1504	0.2537	0.4662
ENSRNOT00000012644	chr8	ENSRNOG00000009450	Hcn4	63600663	63639654	+	0.8636	0.0005	0.0314	2.5647	6.5726	6.2401	5.9273	3.8264	2.6318	2.1509	3.0998
ENSRNOT00000012657	chr4	ENSRNOG00000009197	Asb4	30313102	30501329	+	2.4376	0.0002	0.0157	0.1339	2.9506	1.0890	0.7738	0.6425	0.0660	0.0316	0.1732
ENSRNOT00000012726	chr5	ENSRNOG00000009589	Angptl7	165312130	165316652	-	3.5990	0.0002	0.0165	0.0232	0.1201	0.1366	0.1136	0.0325	0	0	0
ENSRNOT00000012995	chr4	ENSRNOG00000009711	Hepacam2	28403125	28437676	-	Inf	0.0004	0.0261	0	0.1171	0	0.0638	0	0	0	0
ENSRNOT00000013128	chr4	ENSRNOG00000009324	LOC103692166	113981653	113988246	-	Inf	0.0000	0.0007	0	3.1610	0	0	0	0	0	0
ENSRNOT00000013483	chr1	ENSRNOG00000010158	Magel2	123015746	123019522	+	1.1499	0.0011	0.0492	1.0739	6.0902	1.9305	3.2830	2.1177	1.0179	0.9451	1.4973
ENSRNOT00000013694	chr2	ENSRNOG00000010121	Lef1	236233239	236345056	+	1.3424	0.0001	0.0077	2.6018	5.8953	12.4670	11.6866	4.2079	2.8185	2.3682	3.4821
ENSRNOT00000013833	chr4	ENSRNOG00000010347	Styk1	165529294	165541314	-	1.8358	0.0008	0.0389	0.2995	0.5420	1.4550	0.8228	0.2860	0.3065	0.0778	0.2035
ENSRNOT00000013910	chr4	ENSRNOG00000010053	Calcr	28627442	28702559	-	2.4351	0.0009	0.0417	0.0201	3.6010	1.1187	0.0682	0.5943	0.0884	0.1087	0.0976
ENSRNOT00000014036	chr2	ENSRNOG00000010560	Otp	24546536	24554683	+	3.6272	0.0007	0.0362	0	3.8277	0.3607	0.5022	0.3542	0	0.0254	0
ENSRNOT00000014147	chr16	ENSRNOG00000010392	Nrg1	63837216	64057434	+	Inf	0.0001	0.0069	0	0	0.5503	0	0	0	0	0
ENSRNOT00000014437	chr4	ENSRNOG00000010806	Npvf	80391785	80395502	-	5.9315	0.0007	0.0386	0	5.7635	0	0	0.0944	0	0	0
ENSRNOT00000014452	chr3	ENSRNOG00000010551	Lhx2	22640604	22657887	+	5.3817	0.0000	0.0000	11.3657	12.8106	0.1727	11.7859	0.1949	0.2884	0.1893	0.1942

ENSRNOT00000014718	chr4	ENSRNOG00000010609	Abcf2	7122916	7135604	+	1.9095	0.0004	0.0271	16.2773	17.7769	15.8402	20.3532	0.6033	0.8891	0.4120	16.7939
ENSRNOT00000014747	chr15	ENSRNOG00000010997	Ednrb	88006977	88036354	-	0.8028	0.0009	0.0443	15.1411	53.0342	44.7615	42.1159	28.4554	12.7096	22.7761	24.9423
ENSRNOT00000015147	chr17	ENSRNOG00000011335	Gpr50	36771639	36776225	+	4.3591	0.0001	0.0080	0.0147	0.6596	0.1021	0.1092	0.0432	0	0	0
ENSRNOT00000015150	chr10	ENSRNOG00000011196	Krt25	87225343	87232723	-	3.7557	0.0002	0.0170	0.0211	0.0779	0.0288	0.4123	0.0200	0	0	0.0199
ENSRNOT00000015272	chr19	ENSRNOG00000011180	Irx5	16417817	16420511	+	4.1675	0.0006	0.0333	0	1.4224	0.0291	1.6856	0.1746	0	0	0
ENSRNOT00000015583	chr19	ENSRNOG00000011533	Irx3	15838714	15840990	-	3.2968	0.0002	0.0145	0.0194	1.0903	0.6179	2.3387	0.3406	0	0.0185	0.0547
ENSRNOT00000015944	chr4	ENSRNOG00000011824	Trh	124110716	124113242	-	1.5359	0.0011	0.0490	1.3745	9.7116	12.7588	0.9744	3.3728	1.6607	1.0428	2.4831
ENSRNOT00000016232	chr4	ENSRNOG00000012195	Tas2r108	68656928	68657842	+	3.3405	0.0009	0.0424	0.0996	0.0828	0.0184	0.0683	0	0	0.0266	0
ENSRNOT00000016452	chr16	ENSRNOG00000012283	Chrna6	68860018	68866718	+	5.8986	0.0000	0.0002	0	2.3348	0.6269	0.9722	0	0	0.0338	0.0322
ENSRNOT00000016475	chr2	ENSRNOG00000011923	Mgarp	140444965	140453107	-	3.0209	0.0000	0.0026	0.1009	0.8940	2.1378	1.3671	0.2628	0.0647	0.1227	0.1041
ENSRNOT00000016568	chr15	ENSRNOG00000011548	Ebf2	43905099	44103742	+	1.7671	0.0003	0.0225	0.1615	0.6306	0.3246	0.7053	0.2025	0.1542	0.1145	0.0641
ENSRNOT00000016656	chr16	ENSRNOG00000012448	Chrnb3	68875709	68913628	-	4.6086	0.0000	0.0000	0.0273	2.6028	0.4838	1.2185	0.0818	0.0234	0.0311	0.0414
ENSRNOT00000016841	chr10	ENSRNOG00000012134	Scn4a	94505026	94557764	-	1.2780	0.0008	0.0406	0.3304	0.7150	0.4226	0.7106	0.2201	0.1743	0.1944	0.3096
ENSRNOT00000016843	chr2	ENSRNOG00000012478	Shox2	164118191	164126783	-	2.4619	0.0006	0.0343	0.0544	4.6222	16.8571	18.4389	3.8409	0.0753	0.2517	3.0873
ENSRNOT00000016915	chr3	ENSRNOG00000012647	Nkx2-4	141304005	141305471	-	4.5139	0.0001	0.0100	0.0286	1.2999	0.0261	0.0294	0.0336	0	0.0270	0
ENSRNOT00000017317	chr19	ENSRNOG00000012789	Mlnr	55176258	55183557	-	1.3204	0.0001	0.0098	1.6800	4.2589	7.6119	6.6980	2.8923	1.7079	1.4665	2.0414
ENSRNOT00000017651	chr3	ENSRNOG00000012682	Nr5a1	22999616	23020441	-	4.3324	0.0002	0.0176	0	0.9598	0.0400	0.0252	0.0265	0.0179	0.0065	0
ENSRNOT00000017725	chr3	ENSRNOG00000013209	Barhl1	7491234	7498555	-	4.0163	0.0000	0.0048	0	1.0038	0.1417	2.4158	0.1156	0.0163	0.0683	0.0199
ENSRNOT00000017742	chr3	ENSRNOG00000013133	Foxa2	142383278	142387481	+	Inf	0.0000	0.0026	0	0.2710	0	0.2849	0	0	0	0
ENSRNOT00000018126	chr7	ENSRNOG00000013076	Csnk1e	120652697	120672350	-	Inf	0.0002	0.0141	0.6898	0	0	0	0	0	0	0
ENSRNOT00000018556	chr1	ENSRNOG00000013436	Pde7b	15893533	16203838	-	9.9614	0.0000	0.0001	0.0060	0	1.6952	1.3695	0	0	0.0031	0
ENSRNOT00000018562	chr4	ENSRNOG00000013668	Capg	100407658	100419446	+	1.0917	0.0009	0.0432	3.2698	3.7465	4.2910	3.4261	1.7530	1.9997	1.2597	1.9004
ENSRNOT00000018581	chr3	ENSRNOG00000013859	Bpifa1	149624712	149630613	+	3.9413	0.0002	0.0186	0.0214	1.0322	0.0173	0.0774	0	0.0278	0.0470	0
ENSRNOT00000018652	chr9	ENSRNOG00000013670	Pax3	84005183	84101172	-	Inf	0.0000	0.0011	0	0.0173	0.0690	0.4122	0	0	0	0
ENSRNOT00000018846	chr14	ENSRNOG00000013720	Aebp1	86101277	86111306	+	1.0013	0.0001	0.0085	6.8627	6.9849	12.4381	9.5128	4.5585	3.4392	3.2182	6.6673
ENSRNOT00000018953	chr5	ENSRNOG00000028064	Fhad1	160437896	160555083	-	1.8278	0.0001	0.0100	0.3255	1.4379	1.6772	1.5615	0.3373	0.0769	0.6156	0.3792
ENSRNOT00000018990	chr1	ENSRNOG00000013925	Nox4	150797084	150976194	+	2.2286	0.0001	0.0124	0.0870	0.1391	0.1398	0.1380	0.0460	0.0310	0.0076	0.0230
ENSRNOT00000019319	chr1	ENSRNOG00000014290	Grm1	4753144	5165859	-	0.8324	0.0005	0.0286	3.5315	7.8284	8.2598	8.4221	5.1674	3.4483	2.5933	4.5396

ENSRNOT00000019349	chr2	ENSRNOG00000014385	Wnt2b	207440886	207455258	-	1.9282	0.0005	0.0302	0.1312	1.4402	1.8630	1.9647	0.7227	0.1860	0.2279	0.2820
ENSRNOT00000019408	chr19	ENSRNOG00000014452	Zfhx3	42758127	42920344	-	1.3827	0.0000	0.0013	2.7869	9.4937	9.2472	10.6495	4.8246	2.2768	1.7700	3.4686
ENSRNOT00000019681	chr1	ENSRNOG00000049232	Tcf7l2	276659542	276730514	+	1.9736	0.0000	0.0031	2.3637	13.3267	28.3707	40.0818	10.8608	2.1112	2.6810	5.7706
ENSRNOT00000019860	chr8	ENSRNOG00000014644	Zic1	98734295	98738446	-	1.7180	0.0000	0.0004	12.1479	11.3145	36.8797	16.8484	7.9506	4.5986	2.5383	8.3764
ENSRNOT00000019862	chr17	ENSRNOG00000014616	Iars	15356016	15402631	-	5.9666	0.0001	0.0103	2.9213	0	0	3.7729	0	0	0.1070	0
ENSRNOT00000019863	chr10	ENSRNOG00000014740	Gast	88245532	88248484	+	3.3066	0.0003	0.0204	0.2633	1.1461	0.1242	0.5479	0.0649	0	0	0.1455
ENSRNOT00000019913	chr1	ENSRNOG00000047098	LOC103694855	168945567	168953025	+	1.1312	0.0008	0.0408	11.1716	5.5148	15.2944	15.4150	6.6662	6.3622	3.3566	5.2535
ENSRNOT00000019931	chr2	ENSRNOG00000014867	Synpo2	227255902	227411964	-	2.0206	0.0000	0.0024	1.0735	5.9332	14.9719	9.2257	3.4056	1.1923	0.5936	2.4989
ENSRNOT00000019938	chr8	ENSRNOG00000014871	Zic4	98745310	98761840	+	1.7346	0.0000	0.0054	1.1086	0.8588	2.5881	1.1016	0.4782	0.4723	0.2598	0.4896
ENSRNOT00000020204	chr17	ENSRNOG00000014900	Crem	57040023	57088502	+	4.9364	0.0000	0.0042	0.0215	0.4541	0.0251	0.4235	0	0	0	0.0302
ENSRNOT00000020700	chr19	ENSRNOG00000014776	Adcy7	19704859	19727081	-	1.1201	0.0005	0.0303	1.1879	3.2990	1.8133	3.0230	1.3248	0.8343	0.8553	1.2747
ENSRNOT00000020926	chr3	ENSRNOG00000015550	Ptgds	2686123	2689084	-	0.8167	0.0005	0.0308	197.7533	185.7645	355.6343	517.5367	185.0659	109.9291	148.2154	270.2596
ENSRNOT00000021218	chr8	ENSRNOG00000015835	Cacna2d2	116154736	116283746	+	0.9083	0.0009	0.0430	3.4705	4.9988	3.2332	4.6094	3.3040	1.9680	1.3362	2.0833
ENSRNOT00000021337	chr8	ENSRNOG00000015896	Rbpms2	71167305	71200289	+	Inf	0.0002	0.0141	0.5634	0	0	0	0	0	0	0
ENSRNOT00000021432	chr9	ENSRNOG00000015408	Vil1	81689802	81717621	+	3.1087	0.0001	0.0070	0.0410	0.0563	0.1607	0.1715	0.0088	0	0.0178	0.0233
ENSRNOT00000021471	chr1	ENSRNOG00000016011	Plekhg1	40389638	40520447	+	1.0977	0.0002	0.0186	5.0667	14.6714	31.4303	21.9425	11.3034	4.4174	7.1157	11.3245
ENSRNOT00000021702	chr1	ENSRNOG00000016102	Ebf3	209523157	209641123	-	4.0323	0.0000	0.0006	0.0251	2.7956	0.6382	4.1103	0.3631	0.0321	0.0288	0.0387
ENSRNOT00000021740	chr3	ENSRNOG00000016179	Acoxl	120414395	120724809	+	Inf	0.0003	0.0200	0	0.0418	0.0357	0.0039	0	0	0	0
ENSRNOT00000021826	chr4	ENSRNOG00000016255	Podxl2	120785495	120817377	-	0.5917	0.0008	0.0417	44.2279	77.3191	49.9558	71.4819	46.0311	39.3825	35.4186	40.4000
ENSRNOT00000021918	chr17	ENSRNOG00000016283	Meig1	78817529	78829411	+	Inf	0.0002	0.0151	0	0.1683	0.1739	0.0408	0	0	0	0
ENSRNOT00000021972	chr18	ENSRNOG00000026136	Tnfaip8	44737154	44779914	+	3.0021	0.0001	0.0100	0.1264	0.1827	0.1000	3.6395	0.0975	0.0720	0.1949	0.1409
ENSRNOT00000022282	chr5	ENSRNOG00000016553	Vwa5b1	157002826	157059508	-	1.1007	0.0006	0.0339	1.7627	4.7517	2.3978	1.8143	2.0607	0.7789	0.9520	1.2102
ENSRNOT00000022383	chr1	ENSRNOG00000016147	Slc17a6	106998623	107038704	+	2.0055	0.0000	0.0003	5.3135	45.0955	46.5533	61.2789	18.3180	6.0498	6.3911	8.6517
ENSRNOT00000022577	chr1	ENSRNOG00000029745	AABR07002677.1	80330049	80331626	-	Inf	0.0007	0.0375	0.1106	0.0991	0.0736	0.0486	0	0	0	0
ENSRNOT00000022784	chr18	ENSRNOG00000016944	Rax	61638352	61642056	-	Inf	0.0002	0.0146	0.0183	0.2099	0.0416	0	0	0	0	0
ENSRNOT00000022943	chr19	ENSRNOG00000016977	Calb2	41482728	41509658	+	1.5195	0.0001	0.0112	6.4273	70.3499	29.9835	54.2714	25.8219	5.1519	11.3998	13.7971
ENSRNOT00000022953	chr3	ENSRNOG00000017019	Lmx1b	12609574	12686869	-	4.4560	0.0011	0.0484	0	1.4452	0	0.1554	0.0729	0	0	0
ENSRNOT00000022968	chr5	ENSRNOG00000016782	Tnfrsf8	163186349	163231578	-	2.6757	0.0002	0.0169	0.0445	0.1575	0.2830	0.2610	0	0.0575	0.0123	0.0470

ENSRNOT00000023060	chr5	ENSRNOG00000017073	Car9	59008933	59015528	+	2.0090	0.0007	0.0376	0.0825	0.1625	0.2998	0.1196	0.0396	0.0189	0.0717	0.0348
ENSRNOT00000023240	chr4	ENSRNOG00000017066	Zfp384	157523771	157552596	+	Inf	0.0000	0.0000	1.2554	1.0633	1.1273	1.4104	0	0	0	0
ENSRNOT00000023899	chr5	ENSRNOG00000017686	Pi15	790833	819326	-	2.7298	0.0004	0.0270	0.0346	0.2623	0.4341	0.0787	0.0427	0	0.0548	0.0246
ENSRNOT00000023984	chr16	ENSRNOG00000017689	Itih3	6992171	7007051	-	Inf	0.0000	0.0001	0	0	0	23.1081	0	0	0	0
ENSRNOT00000024000	chr1	ENSRNOG00000017619	Aldh1a1	238222521	238264330	+	1.0289	0.0002	0.0185	6.3602	15.3651	5.5431	15.4272	6.0013	4.9172	4.5497	5.4557
ENSRNOT00000024060	chr1	ENSRNOG00000017767	Mrvi1	175681960	175796040	-	1.1228	0.0008	0.0398	0.7293	1.3809	2.1497	2.4969	1.1673	0.5526	0.4985	0.8844
ENSRNOT00000024072	chr10	ENSRNOG00000017893	Baiap3	14599197	14613878	-	1.6862	0.0001	0.0078	2.8161	29.0923	5.6112	11.8016	8.3629	1.9310	2.1122	2.9205
ENSRNOT00000024430	chr17	ENSRNOG00000018087	Vim	80882666	80891212	+	1.0114	0.0001	0.0110	31.4984	31.9057	80.7668	26.6211	20.9323	13.3766	20.7360	29.6797
ENSRNOT00000024458	chr17	ENSRNOG00000018092	Cd83	24416651	24435790	+	0.7223	0.0010	0.0459	10.0642	17.8054	12.2239	18.2983	10.7351	8.7164	7.2318	8.7104
ENSRNOT00000024464	chr5	ENSRNOG00000018109	Clic4	153568745	153625869	-	0.6714	0.0008	0.0400	28.6266	40.4705	46.6111	42.2347	30.3301	19.6317	23.3841	25.8284
ENSRNOT00000024529	chr8	ENSRNOG00000018153	Rbm5	116503160	116531784	-	1.6701	0.0008	0.0403	0.5556	0.5252	0.5876	5.2097	0.3209	0.5858	0.6257	0.6289
ENSRNOT00000024765	chr1	ENSRNOG00000018337	Caly	212537848	212549477	-	0.8795	0.0007	0.0353	15.1093	55.7796	26.0573	44.6038	22.8588	12.2995	22.1092	19.6739
ENSRNOT00000025293	chr8	ENSRNOG00000018711	Ppcdc	62085547	62109166	-	Inf	0.0000	0.0040	0.9207	0	0	0	0	0	0	0
ENSRNOT00000025344	chr18	ENSRNOG00000018735	Cd74	56071478	56080849	+	1.5240	0.0004	0.0242	8.0921	2.4464	37.6498	8.9377	5.6264	2.7122	3.9740	7.5514
ENSRNOT00000025354	chr18	ENSRNOG00000018735	Cd74	56071574	56080397	+	1.6304	0.0009	0.0426	1.9145	1.0216	8.8294	2.0233	0.9205	0.7429	1.1207	1.6696
ENSRNOT00000025488	chr5	ENSRNOG00000018739	Pax7	158214616	158313426	-	6.4684	0.0001	0.0097	0	0.1894	0	1.5057	0.0191	0	0	0
ENSRNOT00000025547	chr10	ENSRNOG00000018892	Hcrt	88669214	88670430	-	4.3796	0.0000	0.0036	0.0522	54.3247	2.5318	0.9991	0.8251	0	1.4035	0.5533
ENSRNOT00000025858	chr16	ENSRNOG00000016346	Prkcd	6655120	6675746	-	2.0078	0.0000	0.0009	7.5582	25.2365	77.9897	48.7054	16.4658	3.6928	5.1324	14.3651
ENSRNOT00000026262	chr19	ENSRNOG00000021521	Chst5	44115120	44135387	-	1.3506	0.0007	0.0353	0.8648	1.8127	3.1918	1.8672	0.9376	0.6728	0.6884	0.7350
ENSRNOT00000026370	chr16	ENSRNOG00000019473	Dcun1d2	81593532	81614553	+	1.6597	0.0009	0.0441	2.0033	0.7321	0.5925	2.3032	0.1086	0.2159	0.2677	1.1900
ENSRNOT00000026607	chr1	ENSRNOG00000019613	Syt9	171592797	171769780	+	1.0130	0.0006	0.0353	3.8322	12.0783	9.6978	9.9338	5.7291	4.4643	3.3659	4.0526
ENSRNOT00000026702	chr1	ENSRNOG00000019706	Phox2a	166893734	166898252	+	Inf	0.0001	0.0123	0.0242	0	0.0753	0.0417	0	0	0	0
ENSRNOT00000026878	chr10	ENSRNOG00000019830	Hsd17b1	88987558	88989757	+	1.8687	0.0007	0.0376	0.2841	0.2495	0.3818	0.3730	0.0898	0.0271	0.1517	0.0842
ENSRNOT00000027132	chr1	ENSRNOG00000020014	Myh14	100608975	100671074	-	1.4270	0.0002	0.0164	5.1156	8.0790	9.1006	7.7856	5.9439	0.4648	3.7252	1.0533
ENSRNOT00000027237	chr4	ENSRNOG00000007281	Flnc	56711049	56738791	+	0.8200	0.0004	0.0255	2.0306	2.7061	3.7846	3.9248	2.0645	1.5189	1.5884	1.8780
ENSRNOT00000027552	chr8	ENSRNOG00000020284	Prkar2a	117486083	117545564	+	3.5526	0.0000	0.0019	0.2841	0.3635	11.7319	0.3224	0.2674	0.3064	0.2555	0.2533
ENSRNOT00000027682	chr1	ENSRNOG00000020410	Th	216073031	216080287	-	3.2871	0.0000	0.0003	0.7210	19.8145	0.6873	6.3400	1.0383	0.4492	0.6770	0.6590
ENSRNOT00000027690	chr2	ENSRNOG00000020421	Fndc7	211655482	211690716	-	1.4320	0.0006	0.0332	0.4356	1.4398	1.2608	0.9811	0.4043	0.2622	0.2408	0.6187

ENSRNOT00000027847	chr7	ENSRNOG00000020501	Tjp3	11301957	11314535	+	6.8365	0.0003	0.0195	0	0	1.1456	0	0	0	0.0100	0
ENSRNOT00000028084	chr10	ENSRNOG00000020684	Vat1	89366899	89374516	-	0.8294	0.0003	0.0194	17.1834	43.5083	23.2306	23.1282	20.4624	12.7606	13.3376	13.6823
ENSRNOT00000028483	chr1	ENSRNOG00000020980	AABR07006278.1	227892956	227904737	+	Inf	0.0011	0.0471	0	0.0837	0.0361	0.0286	0	0	0	0
ENSRNOT00000028544	chr1	ENSRNOG00000021023	Mag	89345429	89360733	-	0.5929	0.0011	0.0485	29.5131	43.3538	49.2223	42.1738	35.7230	23.3762	23.7258	26.0808
ENSRNOT00000028570	chr1	ENSRNOG00000021046	Sult2b1	101712255	101741441	-	Inf	0.0008	0.0398	0	0	0.4787	0	0	0	0	0
ENSRNOT00000028608	chr1	ENSRNOG00000021074	Olr337	228684136	228685348	+	Inf	0.0001	0.0085	0.0251	0.0313	0.0228	0.0258	0	0	0	0
ENSRNOT00000028615	chr1	ENSRNOG00000021063	Grin2d	101820461	101856881	-	0.8198	0.0010	0.0456	2.7547	7.7413	5.0391	8.0354	4.2174	2.4722	2.9776	3.6859
ENSRNOT00000028653	chr1	ENSRNOG00000021102	Scn1b	89550739	89560719	-	Inf	0.0000	0.0001	0	0	0	64.6593	0	0	0	0
ENSRNOT00000028829	chr3	ENSRNOG00000021225	Oxt	123106694	123107534	+	3.6785	0.0003	0.0236	0	19.2730	17.1411	1.5266	2.6261	0.0635	0.2737	0
ENSRNOT00000028833	chr3	ENSRNOG00000021229	Avp	123117492	123119460	-	3.5318	0.0000	0.0014	0.2340	50.3932	12.4398	1.8533	1.8937	0.4026	1.5902	1.7268
ENSRNOT00000028909	chr8	ENSRNOG00000024438	Bsx	45061670	45065430	+	Inf	0.0000	0.0013	0.0932	1.3236	0.0338	0	0	0	0	0
ENSRNOT00000031058	chr3	ENSRNOG00000026203	Pax8	1527279	1584946	-	4.6208	0.0003	0.0212	0	0.0298	0.0272	0.2181	0	0	0	0.0112
ENSRNOT00000031173	chr1	ENSRNOG00000045741	AABR07071891.3	84639114	84640268	+	5.1974	0.0000	0.0024	0.6933	1.3325	0.3301	0.8199	0.0372	0.0367	0.0068	0.0059
ENSRNOT00000031243	chr19	ENSRNOG00000021517	Tmem231	44137423	44158621	-	2.8741	0.0000	0.0000	3.8389	5.4099	5.5546	1.2749	0.7574	0.2902	0.4428	0.7027
ENSRNOT00000032236	chr13	ENSRNOG00000027024	Rgs16	71179910	71185216	+	1.2617	0.0002	0.0142	3.2403	10.9206	20.8174	14.5142	7.4108	2.9264	3.8269	6.4774
ENSRNOT00000032250	chr12	ENSRNOG00000022483	Trim50	24348321	24365324	-	3.3889	0.0005	0.0293	0.0147	0.0850	0.0259	0.0151	0	0	0	0.0134
ENSRNOT00000032280	chr8	ENSRNOG00000008095	Onecut1	81766041	81793577	+	2.3074	0.0002	0.0140	0.0772	0.5201	0.1722	0.3674	0.0863	0.0583	0.0237	0.0613
ENSRNOT00000032342	chr2	ENSRNOG00000021867	AABR07009508.1	100955041	100955793	+	Inf	0.0003	0.0232	0.0688	0.0798	0.0678	0.0429	0	0	0	0
ENSRNOT00000032634	chr9	ENSRNOG00000018740	Ugt1a9	95241609	95302822	+	3.1274	0.0002	0.0183	0.0099	0.4424	1.6938	0.4962	0.1919	0.0052	0.0060	0.0993
ENSRNOT00000033265	chr4	ENSRNOG00000026306	Clec5a	68810891	68819872	-	6.0454	0.0005	0.0302	0	0.1076	0.3090	0	0	0	0.0063	0
ENSRNOT00000033373	chr5	ENSRNOG00000024729	Pax5	60007587	60191941	-	5.2113	0.0005	0.0286	0	0.0802	0	1.1488	0	0.0332	0	0
ENSRNOT00000034449	chr3	ENSRNOG00000021472	Ermn	44080352	44086006	-	0.5865	0.0010	0.0449	54.1311	60.6871	89.9867	72.4807	59.6466	38.7897	44.4504	41.7734
ENSRNOT00000035040	chr5	ENSRNOG00000021546	Sit1	58985829	58987760	-	Inf	0.0004	0.0266	0.0202	0	0.0908	0.0207	0	0	0	0
ENSRNOT00000035606	chr9	ENSRNOG00000013461	Ralbp1	113579107	113598477	-	3.8768	0.0000	0.0061	0.1879	5.4071	0.0376	0.0009	0.1292	0.0637	0.1236	0.0671
ENSRNOT00000035886	chr10	ENSRNOG00000021987	Tnfrsf17	4251941	4257868	-	3.4167	0.0006	0.0337	0.0315	0.0393	0.1128	0.1364	0.0300	0	0	0
ENSRNOT00000035906	chr4	ENSRNOG00000025848	Sspo	78080310	78133654	+	2.4763	0.0005	0.0298	0.0085	0.0125	0.0215	0.3098	0.0147	0.0123	0.0139	0.0224
ENSRNOT00000035977	chr20	ENSRNOG00000025883	Ip6k3	5733482	5754773	-	2.0115	0.0011	0.0483	0.0423	0.0879	0.2814	0.2748	0.0263	0.0359	0.0417	0.0663
ENSRNOT00000036094	chr13	ENSRNOG00000025987	LOC498236	53030768	53086231	-	Inf	0.0011	0.0493	0	0.0136	0.0100	0.0228	0	0	0	0

ENSRNOT00000037047	chrX	ENSRNOG00000025637	Saxol1	29520263	29522420	+	Inf	0.0006	0.0345	0.0282	0.0176	0.0128	0	0	0	0	0
ENSRNOT00000037529	chr18	ENSRNOG00000022957	Ctxn3	52550739	52551805	+	1.4918	0.0002	0.0150	2.3930	8.1841	5.2530	6.6665	3.0522	1.3136	1.3430	2.2902
ENSRNOT00000038248	chr5	ENSRNOG00000027595	Tfap2e	144740956	144761787	-	Inf	0.0005	0.0312	0.0164	0	0.0150	0.0675	0	0	0	0
ENSRNOT00000038433	chr15	ENSRNOG00000022337	Slitrk6	95507640	95514259	-	1.8461	0.0000	0.0024	0.9256	5.0135	8.3105	4.8899	2.2643	0.6267	0.8756	1.5570
ENSRNOT00000039133	chr1	ENSRNOG00000028274	Myrf	226260558	226292480	-	0.5707	0.0008	0.0411	17.7763	24.1495	31.3417	25.4404	18.5715	14.8991	15.5527	17.4378
ENSRNOT00000039281	chr12	ENSRNOG00000001173	Cabp1	47193964	47209784	+	Inf	0.0011	0.0491	0	0	0.4767	0	0	0	0	0
ENSRNOT00000039331	chr16	ENSRNOG00000028744	Mtnr1a	50339358	50358809	-	Inf	0.0005	0.0311	0.0171	0.0426	0	0.0440	0	0	0	0
ENSRNOT00000040132	chr10	ENSRNOG00000050441	AABR07028989.1	1622573	1674701	+	Inf	0.0000	0.0011	1.1098	0.1068	0	0	0	0	0	0
ENSRNOT00000040291	chr1	ENSRNOG00000017302	Slc6a3	32323011	32363983	-	5.8573	0.0001	0.0095	0	2.1115	0	0.8227	0.0506	0	0	0
ENSRNOT00000040306	chr3	ENSRNOG00000006645	Ryr3	104118063	104502471	-	Inf	0.0002	0.0190	0	0	0.0616	0	0	0	0	0
ENSRNOT00000040422	chr7	ENSRNOG00000014314	Slc39a4	117675720	117680004	-	1.6872	0.0010	0.0447	0.1916	0.3490	0.4305	0.1995	0.1025	0.0668	0.1453	0.0489
ENSRNOT00000041096	chr3	ENSRNOG00000029342	Scn7a	52777214	52849907	-	2.2329	0.0000	0.0016	0.5296	1.4522	2.1146	0.8530	0.3352	0.0834	0.1530	0.4813
ENSRNOT00000041216	chr16	ENSRNOG00000017689	Itih3	6992062	7007287	-	0.7497	0.0004	0.0249	53.8189	162.5887	96.2783	103.7193	71.0878	45.2947	58.8642	72.4024
ENSRNOT00000041527	chr1	ENSRNOG00000038607	Tmem86b	72785001	72787367	+	7.2782	0.0003	0.0233	0.0008	0	0.1679	0.3198	0	0	0.0023	0.0008
ENSRNOT00000041569	chr3	ENSRNOG00000010262	Hdc	119057528	119075606	-	Inf	0.0000	0.0009	0	4.5267	0	0	0	0	0	0
ENSRNOT00000041655	chr8	ENSRNOG00000000204	Syncrip	96107240	96132634	-	Inf	0.0000	0.0023	0	0.4025	0	0.4268	0	0	0	0
ENSRNOT00000041726	chr17	ENSRNOG00000012160	Syk	12614311	12669573	-	Inf	0.0008	0.0402	0	0	0	0.1005	0	0	0	0
ENSRNOT00000041894	chr8	ENSRNOG00000029567	Cxadrl1	107249306	107272122	-	Inf	0.0004	0.0246	0	0.7155	0	0	0	0	0	0
ENSRNOT00000042220	chr11	ENSRNOG00000047658	AABR07034718.1	85042348	85042794	+	Inf	0.0000	0.0054	2.7929	0.2764	0	0	0	0	0	0
ENSRNOT00000042300	chr4	ENSRNOG00000047415	AABR07060788.1	93791054	93791500	+	Inf	0.0000	0.0034	3.4328	0.3847	0	0	0	0	0	0
ENSRNOT00000042717	chr8	ENSRNOG00000031675	Panx3	40126342	40137390	-	3.2844	0.0002	0.0158	0.0377	0.0895	0.0556	0.1290	0	0.0320	0	0
ENSRNOT00000042754	chr11	ENSRNOG00000002171	Phldb2	57404196	57481567	+	5.8146	0.0004	0.0239	0	0.7616	0	0	0.0068	0	0	0.0068
ENSRNOT00000042790	chr9	ENSRNOG00000019598	Vegfa	17340341	17355681	+	Inf	0.0000	0.0000	0	6.8780	0.2635	0.2681	0	0	0	0
ENSRNOT00000042853	chr3	ENSRNOG00000033119	Plcb4	128756799	128971704	+	0.7562	0.0004	0.0240	13.8546	15.3524	27.3030	20.7380	14.8675	12.5014	7.4072	10.9603
ENSRNOT00000042872	chr4	ENSRNOG00000037076	LOC689757	162181158	162230859	-	4.3283	0.0001	0.0091	0.0502	0.0416	0.1333	0.0945	0.0159	0	0	0
ENSRNOT00000043544	chr18	ENSRNOG00000027103	Gpr151	36892403	36893797	-	5.8938	0.0000	0.0020	0	0.8117	0.8135	0.2161	0	0	0	0.0310
ENSRNOT00000044133	chr19	ENSRNOG00000033368	Dand5	25930601	25936026	-	Inf	0.0006	0.0330	0.0665	0	0.1143	0.0764	0	0	0	0
ENSRNOT00000044232	chr2	ENSRNOG00000010681	Pitx2	233602732	233621051	+	7.0190	0.0000	0.0042	0	3.9172	0	0.4842	0.0339	0	0	0

ENSRNOT00000044291	chr16	ENSRNOG00000029264	Kcnn1	20336817	20347956	+	7.6222	0.0005	0.0294	0	0	0.6744	0.0016	0.0010	0.0013	0	0.0011
ENSRNOT00000044348	chr5	ENSRNOG00000013656	Lpar1	75557042	75676584	-	0.5796	0.0010	0.0450	23.5927	28.1368	41.1231	32.9136	23.8056	19.3775	20.4227	20.5494
ENSRNOT00000044378	chr12	ENSRNOG00000030927	Grid2ip	13221513	13251112	-	1.9278	0.0000	0.0024	0.8473	2.3321	5.9702	3.2760	1.3777	0.5613	0.4631	0.8636
ENSRNOT00000044532	chr1	ENSRNOG00000021023	Mag	89345748	89358166	-	0.6203	0.0007	0.0354	43.4642	62.1210	78.2690	64.2785	51.0387	31.4668	36.2932	42.6232
ENSRNOT00000044690	chr1	ENSRNOG00000033609	Irx1	33910912	33916741	+	2.4844	0.0001	0.0093	0.1165	1.9985	1.3042	3.2636	0.6002	0.0832	0.0948	0.4160
ENSRNOT00000044972	chr13	ENSRNOG00000030515	Nfasc	49335408	49487892	-	1.6777	0.0000	0.0033	9.9659	1.5535	2.1824	1.3660	1.3911	1.1780	1.1082	1.0328
ENSRNOT00000045016	chr2	ENSRNOG00000014613	Ddah1	251634431	251766014	+	0.5872	0.0012	0.0499	20.0635	32.2841	41.9977	31.8735	21.7523	22.8193	20.3642	19.0780
ENSRNOT00000045556	chrX	ENSRNOG00000026315	Taf7l	105292338	105308554	-	2.8648	0.0010	0.0447	0.0163	0.0203	0.0247	0.0470	0	0	0.0149	0
ENSRNOT00000045642	chr1	ENSRNOG00000013993	AABR07007000.1	276011416	276012351	-	1.5557	0.0007	0.0355	1.6183	9.0766	14.3545	17.1971	5.6645	2.5313	2.1158	4.0596
ENSRNOT00000046192	chr4	ENSRNOG00000012098	Adcyap1r1	85662892	85711696	+	Inf	0.0000	0.0017	0	0	0.4964	0	0	0	0	0
ENSRNOT00000046456	chr6	ENSRNOG00000004711	Mta1	137924568	137950066	+	Inf	0.0000	0.0003	0	0	9.5973	0	0	0	0	0
ENSRNOT00000047669	chr1	ENSRNOG00000050994	Cttn	215255381	215290845	-	6.4220	0.0003	0.0224	0	0	6.8651	0	0	0	0.0801	0
ENSRNOT00000047685	chr4	ENSRNOG00000014751	Ret	150202058	150244372	-	1.4693	0.0001	0.0136	0.8160	9.5969	3.7844	4.8791	2.5084	0.8174	1.4538	2.1099
ENSRNOT00000048029	chr20	ENSRNOG00000033087	Cdh23	29857637	29990111	-	1.5067	0.0003	0.0228	0.2599	1.9945	0.4881	0.7702	0.4089	0.2340	0.2285	0.3648
ENSRNOT00000048270	chr20	ENSRNOG00000000323	Prdm1	49464919	49486550	-	Inf	0.0001	0.0082	0	0	0.3079	0	0	0	0	0
ENSRNOT00000048400	chr4	ENSRNOG00000046854	AABR07061022.1	102794168	102794614	+	Inf	0.0003	0.0208	2.4031	0	0	0	0	0	0	0
ENSRNOT00000048770	chr5	ENSRNOG00000019484	Slc6a9	136669674	136703697	+	0.7857	0.0002	0.0187	21.4909	49.1068	38.3791	49.6451	31.2936	17.0598	19.8211	23.8392
ENSRNOT00000049672	chr6	ENSRNOG00000005705	Qpct	1657331	1689559	+	1.2765	0.0003	0.0194	2.9659	8.3711	3.4166	6.7261	2.6835	2.1961	1.6054	2.3817
ENSRNOT00000049898	chr18	ENSRNOG00000012852	Bin1	25163604	25222132	+	0.8866	0.0001	0.0068	26.4028	35.8302	50.2960	45.9213	13.9907	20.2156	25.5602	25.9379
ENSRNOT00000050180	chr1	ENSRNOG00000014791	Peg3	70222964	70237443	+	0.5738	0.0011	0.0492	100.2254	215.8253	126.7812	174.9594	123.8618	95.1424	91.0226	105.0269
ENSRNOT00000050656	chr1	ENSRNOG00000012286	Il20ra	15180328	15222785	+	3.1816	0.0008	0.0401	0.0385	0.4762	0.0537	0.0151	0.0540	0	0	0.0103
ENSRNOT00000050760	chr1	ENSRNOG00000020369	Igf2	215828102	215838209	-	Inf	0.0005	0.0281	0	0	0	0.1960	0	0	0	0
ENSRNOT00000050927	chr11	ENSRNOG00000001742	Clcn2	83883879	83897394	+	Inf	0.0000	0.0006	0	0	4.1077	0	0	0	0	0
ENSRNOT00000051009	chr2	ENSRNOG00000010681	Pitx2	233615739	233621129	+	3.8831	0.0000	0.0037	0.0149	0.8824	0.0271	0.2886	0.0352	0.0191	0.0145	0.0134
ENSRNOT00000051213	chr11	ENSRNOG00000001916	Tmprss15	17561596	17684903	-	4.0068	0.0001	0.0076	0.0149	0.0636	0.1535	0.0955	0.0204	0	0	0
ENSRNOT00000051352	chr19	ENSRNOG00000017905	Map1lc3b	53629779	53646055	+	3.8468	0.0000	0.0005	0.2308	0.1991	10.1334	12.2383	0.2163	0.6217	0.3373	0.4096
ENSRNOT00000051512	chr13	ENSRNOG00000030515	Nfasc	49335408	49522415	-	1.1962	0.0000	0.0054	8.8487	2.8354	3.1384	2.5722	2.1951	1.4873	1.8934	2.0156
ENSRNOT00000051525	chr4	ENSRNOG00000030012	Clec4a2	155792761	155867708	-	4.3397	0.0003	0.0224	0.0451	0	0.3109	0.0967	0	0	0	0.0224

ENSRNOT00000052250	chr11	ENSRNOG00000029216	AABR07072266.1	87767159	87769761	-	Inf	0.0000	0.0000	8.2260	11.7561	8.8555	0	0	0	0	0
ENSRNOT00000055274	chr7	ENSRNOG00000036830	RGD1563200	145056693	145062956	-	3.8945	0.0000	0.0056	0.1447	0.2644	0.7532	0.1508	0	0	0.0359	0.0524
ENSRNOT00000056693	chr12	ENSRNOG00000037456	P2rx2	52397830	52400751	+	7.6719	0.0000	0.0009	0	0.6291	0.0212	0.5745	0	0	0.0060	0
ENSRNOT00000056694	chr12	ENSRNOG00000037456	P2rx2	52397812	52401005	+	2.7407	0.0003	0.0222	0.0743	0.3660	0.5234	0.0565	0.0381	0	0.0819	0.0327
ENSRNOT00000057051	chr14	ENSRNOG00000004327	Ddc	91905919	91989307	-	1.2390	0.0005	0.0299	1.1223	3.1785	0.8723	2.1693	0.9192	1.0760	0.4878	0.6277
ENSRNOT00000057062	chr2	ENSRNOG00000016550	Dclk2	186117355	186245342	-	1.1191	0.0005	0.0280	14.6988	16.3937	15.9016	13.8879	5.3259	15.4744	5.4478	1.7802
ENSRNOT00000058152	chr18	ENSRNOG00000038197	Cd226	86299463	86392713	+	Inf	0.0006	0.0349	0	0	0	0.2537	0	0	0	0
ENSRNOT00000058314	chr20	ENSRNOG00000038300	Gstt4	13778178	13784827	+	2.5438	0.0010	0.0457	0.0560	0.2390	0.2361	0.2890	0.0893	0	0.0514	0
ENSRNOT00000058362	chr10	ENSRNOG00000038328	Gjc2	45526745	45534570	-	0.6460	0.0011	0.0477	18.3059	25.5110	30.1184	25.5672	21.2663	13.8257	14.7556	13.7383
ENSRNOT00000058497	chr13	ENSRNOG00000046452	Fcgr2b	89329304	89343868	-	Inf	0.0000	0.0059	0.4414	0	0.0975	0	0	0	0	0
ENSRNOT00000058833	chrX	ENSRNOG00000032472	Adgrg2	36930186	37003642	-	6.6849	0.0002	0.0154	0	0	0	1.2340	0.0120	0	0	0
ENSRNOT00000059496	chr10	ENSRNOG00000006557	Cyfip2	31278746	31419235	-	1.3376	0.0001	0.0083	256.3277	143.5668	35.8236	34.5592	44.0370	51.8284	45.7520	44.4623
ENSRNOT00000060351	chr19	ENSRNOG00000031773	Cngb1	10142496	10206618	+	2.5785	0.0009	0.0423	0.0636	0.1767	0.0327	0.0476	0.0221	0	0.0315	0
ENSRNOT00000060515	chr14	ENSRNOG00000050800	LOC100911576	88543630	88574195	+	2.2753	0.0004	0.0256	0.4611	0.3833	16.4034	0.6939	0.7795	1.1485	0.9873	0.7909
ENSRNOT00000060767	chr1	ENSRNOG00000019549	Akap12	40816107	40906581	+	1.1141	0.0000	0.0030	7.1312	21.6573	21.6346	26.8131	11.3254	7.3042	7.2748	9.7764
ENSRNOT00000061032	chr2	ENSRNOG00000010720	Mast4	32444565	32518643	-	0.6646	0.0011	0.0470	6.9705	9.6033	6.1473	10.4707	3.7727	6.2034	6.6974	4.2653
ENSRNOT00000061047	chr11	ENSRNOG00000001628	Pcp4	36851038	36912229	+	0.8231	0.0004	0.0246	135.3865	161.7445	315.0032	234.3994	168.8966	140.1023	72.9873	96.4867
ENSRNOT00000061616	chr8	ENSRNOG00000008990	Amotl1	13042641	13109487	-	0.9686	0.0002	0.0156	4.6161	12.4852	10.5445	10.8927	6.3018	3.9536	4.1501	5.2870
ENSRNOT00000062073	chr4	ENSRNOG00000028311	AABR07058998.1	476977	482645	-	Inf	0.0000	0.0021	0	0.7015	0.2049	1.3270	0	0	0	0
ENSRNOT00000063800	chr15	ENSRNOG00000031100	Klhl1	80271731	80713153	-	1.3533	0.0005	0.0314	1.6359	2.8354	1.0261	3.2059	1.1192	1.2135	0.3932	0.6805
ENSRNOT00000063821	chr2	ENSRNOG00000029212	Vcan	18490102	18565842	-	Inf	0.0000	0.0032	0	0	0.9015	0	0	0	0	0
ENSRNOT00000063889	chr10	ENSRNOG00000013588	Glra1	40855559	40953651	-	2.7427	0.0000	0.0016	0.2785	6.7365	3.0111	9.5645	1.4940	0.2054	0.4200	0.8076
ENSRNOT00000064064	chr10	ENSRNOG00000030726	Asgr2	56710475	56723601	+	3.6135	0.0004	0.0246	0.1029	0.1376	0.0191	0.1179	0	0	0	0.0308
ENSRNOT00000064203	chr3	ENSRNOG00000060572	Tlk1	57013025	57104030	-	4.1807	0.0001	0.0114	0	8.6293	9.4888	1.2807	0.0635	0	0.0167	0.9895
ENSRNOT00000064788	chr16	ENSRNOG00000010189	Rps24	757403	760568	+	4.7162	0.0000	0.0000	2.9448	129.2406	146.3670	1.7448	3.0843	3.5595	1.2301	2.7893
ENSRNOT00000065052	chr3	ENSRNOG00000048056	Spag4	151609602	151613942	+	Inf	0.0002	0.0170	0.6419	0	0	0	0	0	0	0
ENSRNOT00000065126	chr3	ENSRNOG00000006639	Scn9a	52583951	52664209	-	1.4865	0.0002	0.0179	0.4037	2.3493	0.7916	0.5688	0.5631	0.3341	0.2453	0.3254
ENSRNOT00000065292	chr10	ENSRNOG00000028812	Trim80	103992309	104004059	+	Inf	0.0005	0.0306	0.0141	0.0192	0.0386	0	0	0	0	0

ENSRNOT00000065890	chr20	ENSRNOG00000000302	Sesn1	46667454	46758305	+	Inf	0.0000	0.0051	0	0	0.7620	0	0	0	0	0
ENSRNOT00000065899	chr7	ENSRNOG00000004682	Parpbp	28654707	28715224	-	2.8261	0.0007	0.0367	0.0155	0.7704	0.0999	0.0212	0.0716	0	0.0368	0.0195
ENSRNOT00000065947	chr10	ENSRNOG00000004147	Abca8a	98319480	98390384	-	0.6381	0.0009	0.0418	10.1556	14.9321	19.5376	14.0845	11.5040	8.7518	10.1023	7.3656
ENSRNOT00000066048	chr18	ENSRNOG00000017503	Ppargc1b	56626736	56728185	-	6.4570	0.0001	0.0098	0.0002	0.0012	0.5481	0	0.0012	0.0015	0.0006	0.0030
ENSRNOT00000066715	chr6	ENSRNOG00000043137	Lbhd2	135856218	135861059	+	1.8854	0.0000	0.0057	3.3420	4.2447	4.2642	2.5724	1.2265	1.0402	0.8083	0.8289
ENSRNOT00000066928	chr15	ENSRNOG00000005985	Kcnma1	344710	1047914	+	3.5005	0.0000	0.0050	0.1649	4.9977	0.2606	0.2448	0.3225	0.0144	0.1564	0.0076
ENSRNOT00000067389	chr5	ENSRNOG00000006553	Bnc2	102415847	102743417	-	2.3539	0.0001	0.0090	0.2460	0.5005	0.3556	2.2892	0.2330	0.0524	0.0719	0.3060
ENSRNOT00000067392	chr1	ENSRNOG00000021149	Ush1c	102207096	102255459	-	2.7390	0.0003	0.0199	0.0567	0.0700	0.2267	0.1946	0.0399	0.0292	0.0130	0
ENSRNOT00000067396	chr4	ENSRNOG00000023077	Cpne9	145238947	145262442	+	1.0511	0.0003	0.0218	4.8782	11.7589	19.0933	17.0877	9.1435	5.7733	4.3327	6.2403
ENSRNOT00000067500	chr3	ENSRNOG00000022514	Tor2a	11795679	11799074	+	Inf	0.0000	0.0021	0.2346	0.4572	0.1552	0	0	0	0	0
ENSRNOT00000067639	chr16	ENSRNOG00000016346	Prkcd	6655662	6669045	-	3.4324	0.0001	0.0106	0.0167	0.0406	0.2641	0.1478	0.0153	0	0.0145	0.0137
ENSRNOT00000067700	chr3	ENSRNOG00000010128	Slc27a2	119014620	119052531	+	1.6670	0.0011	0.0479	0.2271	1.3348	0.3658	2.0634	0.5183	0.1401	0.2164	0.3821
ENSRNOT00000067875	chr14	ENSRNOG00000043451	Spp1	6673686	6679901	-	2.4236	0.0000	0.0014	1.5385	2.8518	7.1947	16.4742	1.4412	0.8820	0.4310	2.4759
ENSRNOT00000068030	chr6	ENSRNOG00000005151	Dync2li1	7901018	7933793	+	2.7560	0.0001	0.0096	2.2490	0.3820	1.5248	2.0885	0.0555	0.6718	0.1885	0.0086
ENSRNOT00000068747	chr9	ENSRNOG00000019474	Rsph9	17225122	17245093	+	3.6467	0.0004	0.0268	0.0978	0.2160	0.1746	0.0562	0.0024	0.0009	0.0003	0.0398
ENSRNOT00000068764	chr19	ENSRNOG00000042274	Fbxo31	53487619	53625673	-	1.4804	0.0000	0.0055	35.1099	6.3277	6.6881	30.6437	7.8480	6.3102	7.4626	6.6090
ENSRNOT00000070923	chr6	ENSRNOG00000049315	AABR07065827.1	143065206	143065639	-	Inf	0.0001	0.0121	0.3421	0.4212	0.0739	0	0	0	0	0
ENSRNOT00000071131	chr17	ENSRNOG00000045684	LOC100910978	14696308	14717420	-	Inf	0.0010	0.0455	0	0	0	0.2369	0	0	0	0
ENSRNOT00000071218	chr10	ENSRNOG00000047546	Gas2l2	70639862	70646495	-	Inf	0.0001	0.0088	0	0	0.5421	0	0	0	0	0
ENSRNOT00000071235	chr1	ENSRNOG00000047651	LOC103690160	215214853	215241723	+	5.5518	0.0000	0.0036	20.4227	11.9093	0.0019	0	0.1991	0.1504	0.3397	0
ENSRNOT00000071545	chr18	ENSRNOG00000024426	AABR07032520.1	69841053	69849389	+	5.3463	0.0002	0.0184	1.7368	0.0456	0	0.0379	0	0	0	0.0447
ENSRNOT00000071612	chr18	ENSRNOG00000049893	LOC100910934	55391388	55392641	+	3.3646	0.0000	0.0018	1.8445	0.5242	0.5104	0.0640	0.0663	0.0457	0.1206	0.0531
ENSRNOT00000071735	chr6	ENSRNOG00000047300	Bdkrb2	129399468	129429676	+	Inf	0.0000	0.0000	0.3306	0.3546	0	0.1348	0	0	0	0
ENSRNOT00000071856	chr9	ENSRNOG00000016503	Smarcal1	79944132	79990229	+	2.9166	0.0007	0.0377	0.0389	0.0007	0.1539	0.0467	0.0035	0.0056	0.0034	0.0193
ENSRNOT00000071914	chr7	ENSRNOG00000046069	Higd1c	142001338	142020705	+	Inf	0.0000	0.0025	0.2373	0.3090	0.2472	0.0958	0	0	0	0
ENSRNOT00000071930	chr4	ENSRNOG00000049943	Sinhcaf	183403000	183424449	-	Inf	0.0003	0.0200	0	0	0.5332	0	0	0	0	0
ENSRNOT00000072377	chr20	ENSRNOG00000047657	C4a	2651702	2678141	-	3.9872	0.0000	0.0004	1.9971	0.2272	0.2667	0.7343	0	0.0937	0.0906	0.0190
ENSRNOT00000072386	chr14	ENSRNOG00000049895	LOC100910143	2410342	2438592	-	Inf	0.0000	0.0000	3.3578	3.8441	0	0	0	0	0	0

ENSRNOT00000072502	chr3	ENSRNOG00000024923	Nnat	154043873	154046330	+	1.2216	0.0000	0.0036	35.2524	120.4194	49.1432	98.4842	44.5063	16.9120	29.4909	39.1460
ENSRNOT00000072745	chr15	ENSRNOG00000049773	LOC688340	29359765	29360198	-	Inf	0.0006	0.0352	0.3062	0.0741	0	0.0839	0	0	0	0
ENSRNOT00000073192	chr5	ENSRNOG00000050760	LOC103690035	64566804	64584309	+	7.1790	0.0000	0.0040	0.1111	0	7.1856	0	0	0	0	0.0504
ENSRNOT00000073330	chr11	ENSRNOG00000048273	Apod	72705129	72726301	+	0.7498	0.0003	0.0232	80.1572	126.4672	168.3021	171.2252	113.0492	56.1157	77.1835	78.4370
ENSRNOT00000073428	chr6	ENSRNOG00000049223	Vipas39	111271283	111295917	-	Inf	0.0000	0.0001	0	1.2401	1.0778	0	0	0	0	0
ENSRNOT00000073466	chr4	ENSRNOG00000045831	LOC100909784	51553454	51561174	+	Inf	0.0000	0.0006	0	0.1218	0.3572	0.0282	0	0	0	0
ENSRNOT00000074042	chr5	ENSRNOG00000050670	AABR07049326.1	117354794	117360654	+	Inf	0.0000	0.0043	0.0083	0.0155	0.0196	0.0602	0	0	0	0
ENSRNOT00000074108	chr7	ENSRNOG00000047194	Arl13b	1122567	1188209	-	2.5887	0.0001	0.0088	0.2077	3.6621	0.7328	0.3033	0.5210	0.1139	0.0889	0.0918
ENSRNOT00000074198	chr4	ENSRNOG00000048642	LOC100912564	170117014	170117325	-	2.8051	0.0000	0.0018	8.3393	6.8410	2.9284	7.9429	0.2448	1.3830	1.8123	0.2874
ENSRNOT00000074238	chr8	ENSRNOG00000009878	Crtapl1	122398276	122402127	-	2.4417	0.0010	0.0455	0.0532	0.1130	0.7472	0.5002	0.0660	0.0665	0.0781	0.0496
ENSRNOT00000074295	chr3	ENSRNOG00000050946	Fam110a	147476580	147479472	-	4.6185	0.0000	0.0027	0.0239	0	1.1949	1.0652	0.0253	0.0304	0.0249	0.0124
ENSRNOT00000075073	chr10	ENSRNOG00000047790	LOC108348055	47785033	47792590	+	4.8709	0.0000	0.0042	0	5.8270	7.9100	3.9331	0.5809	0	0.0230	0
ENSRNOT00000075127	chr5	ENSRNOG00000050106	Rcc1	150509592	150525254	-	9.1276	0.0000	0.0001	0.0066	2.7587	3.1806	0	0	0.0106	0	0
ENSRNOT00000075128	chr5	ENSRNOG00000049700	LOC100909857	156757044	156781291	-	4.5009	0.0005	0.0313	0	0.3740	0.2482	0.8602	0	0	0	0.0655
ENSRNOT00000075494	chr9	ENSRNOG00000046834	C3	9721137	9747077	+	1.0412	0.0003	0.0236	10.6464	4.3763	24.3807	6.5435	4.3025	5.6527	5.7331	6.6384
ENSRNOT00000075628	chr19	ENSRNOG00000011989	Vat1l	46525919	46693838	+	0.6771	0.0008	0.0398	22.0282	57.5216	38.5539	31.4565	29.3294	18.4956	22.0498	23.6611
ENSRNOT00000075641	chr12	ENSRNOG00000046184	RGD1561143	21866077	21923281	-	Inf	0.0011	0.0476	0	0	0.1146	0.0440	0	0	0	0
ENSRNOT00000075863	chr14	ENSRNOG00000015033	Mxd4	81819459	81830780	+	3.6427	0.0011	0.0472	0.2445	0.0399	0.2235	0.0005	0.0004	0.0003	0.0003	0.0397
ENSRNOT00000075916	chr5	ENSRNOG00000015206	Alad	78368869	78376032	-	5.2483	0.0000	0.0047	3.2559	0	0.0266	0.0213	0.0172	0.0120	0.0222	0.0356
ENSRNOT00000075989	chr14	ENSRNOG00000043451	Spp1	6673746	6679878	-	Inf	0.0000	0.0000	0.3965	0	1.8403	1.1248	0	0	0	0
ENSRNOT00000076326	chr2	ENSRNOG00000009955	Plch1	154256393	154418920	-	1.0402	0.0009	0.0443	0.6633	2.1418	1.6076	2.6412	0.6893	0.7020	0.9198	1.1189
ENSRNOT00000076429	chr4	ENSRNOG00000010601	Srpk2	8166082	8184587	+	Inf	0.0005	0.0283	0	0	0.1852	0.2204	0	0	0	0
ENSRNOT00000076710	chr2	ENSRNOG00000033134	Mef2c	11658568	11820594	+	2.2301	0.0001	0.0108	1.2160	13.4018	0.6034	0.4316	0.7517	0.9336	0.9418	0.7093
ENSRNOT00000076918	chr5	ENSRNOG00000011504	Akap2	74874306	74954873	+	3.2079	0.0002	0.0182	0.0247	0.2601	3.4531	2.8725	0.0293	0.0199	0.3174	0.3489
ENSRNOT00000077219	chr4	ENSRNOG00000040108	RGD1565355	14151374	14246665	+	Inf	0.0000	0.0002	0.0848	0.1032	0.1179	0.0200	0	0	0	0
ENSRNOT00000077374	chr4	ENSRNOG00000059810	Txnrd3	121612332	121650541	+	6.2442	0.0000	0.0001	0.0009	0.5501	4.0194	0.1185	0.0017	0.0038	0.0036	0.0528
ENSRNOT00000077452	chr16	ENSRNOG00000049792	Glra3	37676962	38063821	-	Inf	0.0002	0.0160	0	0.7950	0	0	0	0	0	0
ENSRNOT00000077458	chr6	ENSRNOG00000012094	Ltbp2	108500119	108596446	-	Inf	0.0000	0.0022	0	0	0.0760	0.1187	0	0	0	0

ENSRNOT00000077616	chr7	ENSRNOG00000006700	Wdyhv1	97957003	97994586	-	6.8913	0.0007	0.0359	0.1148	0.0086	0.1771	0	0.0009	0	0	0.0016
ENSRNOT00000077719	chr1	ENSRNOG00000038600	Dnaaf3	72874583	72883002	+	Inf	0.0000	0.0045	0	0.7660	0.0249	0	0	0	0	0
ENSRNOT00000077793	chr4	ENSRNOG00000054251	Clec7a	163217813	163227334	-	Inf	0.0001	0.0090	0.1778	0	0.6867	0	0	0	0	0
ENSRNOT00000077883	chr2	ENSRNOG00000020485	Vav3	212257225	212585818	+	1.8950	0.0006	0.0327	0.1183	0.6365	1.1398	1.5395	0.2772	0.2191	0.0235	0.4035
ENSRNOT00000078017	chr2	ENSRNOG00000052613	Casp6	235341365	235353967	+	Inf	0.0006	0.0320	0	0.3856	0	0	0	0	0	0
ENSRNOT00000078144	chr1	ENSRNOG00000026296	Saxo2	144586447	144601335	-	2.0046	0.0011	0.0489	0.1966	0.3541	0.6454	0.1698	0.1682	0.1211	0.0465	0.0045
ENSRNOT00000078187	chr2	ENSRNOG00000009582	Rpe65	266141581	266169197	+	1.4539	0.0006	0.0347	0.7513	1.3820	2.0511	0.9717	0.4103	0.2836	0.5148	0.6735
ENSRNOT00000078284	chr1	ENSRNOG00000024000	Cd22	89314632	89329185	-	2.5969	0.0002	0.0161	0.1686	0.0941	2.1241	0.1345	0.1638	0.0793	0.0518	0.1218
ENSRNOT00000078451	chr20	ENSRNOG00000000818	Nrm	3397834	3401276	-	5.2765	0.0000	0.0058	0.2910	0.1817	0.0454	0.0001	0.0132	0	0.0001	0
ENSRNOT00000078494	chr1	ENSRNOG00000019358	Esr1	41325531	41590619	+	Inf	0.0002	0.0178	0	0	0	0.4914	0	0	0	0
ENSRNOT00000078561	chr10	ENSRNOG00000003807	Wnt9b	91787848	91797317	-	2.9428	0.0000	0.0051	0.3071	1.3467	0.7800	1.7060	0.0177	0.0384	0.0252	0.4571
ENSRNOT00000078746	chr14	ENSRNOG00000028043	Cxcl3	18837821	18839595	-	Inf	0.0005	0.0296	0.0276	0.1280	0	0.0202	0	0	0	0
ENSRNOT00000078819	chr3	ENSRNOG00000054086	Sp5	56766475	56769277	+	2.3584	0.0001	0.0135	0.0549	0.3393	0.2489	0.5586	0.0476	0.0552	0.0908	0.0408
ENSRNOT00000078871	chr20	ENSRNOG00000047657	C4a	2651599	2652952	-	Inf	0.0000	0.0062	0	0	2.8471	0	0	0	0	0
ENSRNOT00000078874	chr19	ENSRNOG00000015519	Ces1d	15195565	15239638	+	Inf	0.0001	0.0095	0	0.2644	0.0287	0.0076	0	0	0	0
ENSRNOT00000078969	chr18	ENSRNOG00000049629	Reep2	27632901	27640573	+	Inf	0.0000	0.0043	0	0.8816	0.4219	0	0	0	0	0
ENSRNOT00000078978	chr3	ENSRNOG00000022141	Ctdspl2	113918629	113960101	+	Inf	0.0000	0.0005	3.5466	0	0	0	0	0	0	0
ENSRNOT00000079186	chr1	ENSRNOG00000027422	Fastkd3	37734410	37741709	-	Inf	0.0002	0.0136	0	0	0.5487	0	0	0	0	0
ENSRNOT00000079215	chr16	ENSRNOG00000059865	Dnah12	2379480	2534131	+	1.2361	0.0007	0.0362	0.5768	1.2926	2.9223	0.5681	0.5626	0.2346	0.8044	0.6737
ENSRNOT00000079235	chr7	ENSRNOG00000056493	Mybpc1	29086156	29171783	-	2.4198	0.0000	0.0004	0.6605	1.1274	5.5284	2.5970	0.7149	0.3154	0.3493	0.4731
ENSRNOT00000079260	chr17	ENSRNOG00000014886	Fam107b	78491945	78499881	-	Inf	0.0010	0.0453	0	0.7386	0	0	0	0	0	0
ENSRNOT00000079509	chr20	ENSRNOG00000001152	Glp1r	9586091	9624571	+	Inf	0.0000	0.0039	0.0296	0.0592	0.0064	0.1454	0	0	0	0
ENSRNOT00000079511	chr4	ENSRNOG00000059679	Rtkn	113968995	113984632	+	Inf	0.0000	0.0007	0	0	6.2423	0	0	0	0	0
ENSRNOT00000079547	chr4	ENSRNOG00000059679	Rtkn	113970079	113984292	+	Inf	0.0000	0.0004	0	0	11.4400	0	0	0	0	0
ENSRNOT00000079672	chr3	ENSRNOG00000004544	Ube2e3	65816569	65870698	+	Inf	0.0000	0.0001	0	5.3207	0	8.6709	0	0	0	0
ENSRNOT00000079718	chr1	ENSRNOG00000014166	Smoc2	56288832	56374120	+	Inf	0.0000	0.0002	0.3690	0	0.0888	0.0540	0	0	0	0
ENSRNOT00000079830	chr10	ENSRNOG00000052564	Gpx3	40247436	40255422	+	1.3376	0.0001	0.0082	8.0565	38.4540	11.5067	14.8650	11.2910	6.5782	5.1996	5.7685
ENSRNOT00000079850	chr17	ENSRNOG00000026235	Hk3	10138810	10152956	+	4.1594	0.0004	0.0245	0.0796	0.0600	0.0636	0	0	0.0114	0	0

ENSRNOT00000079926	chr10	ENSRNOG00000027194	Sgsm2	61705343	61744976	-	Inf	0.0001	0.0071	0	0	0	0.5736	0	0	0	0
ENSRNOT00000079987	chr1	ENSRNOG00000017302	Slc6a3	32321580	32362359	-	5.2138	0.0000	0.0006	0.0354	14.0779	0.0286	5.5484	0.3350	0.0116	0.1755	0.0085
ENSRNOT00000080006	chr10	ENSRNOG00000016475	Nt5c3b	88342599	88357025	-	9.0245	0.0000	0.0000	7.4344	10.0115	0	10.0633	0	0.0528	0	0
ENSRNOT00000080101	chr17	ENSRNOG00000016767	Ggps1	53962444	53971443	+	3.9253	0.0002	0.0139	0.0116	0.0031	0.0094	0.5114	0.0022	0.0146	0.0122	0.0062
ENSRNOT00000080146	chr16	ENSRNOG00000018669	Jak3	20110148	20120662	+	4.7015	0.0000	0.0006	0.0341	0.0965	0.0354	0.9545	0.0139	0	0.0143	0.0149
ENSRNOT00000080236	chr8	ENSRNOG00000057447	Kri1	22301498	22308706	-	Inf	0.0000	0.0014	4.1042	0	0	0	0	0	0	0
ENSRNOT00000080442	chr1	ENSRNOG00000047651	LOC103690160	215214853	215241723	+	Inf	0.0000	0.0006	0	4.3190	0	0	0	0	0	0
ENSRNOT00000080584	chr10	ENSRNOG00000010756	Tp53	56193856	56198031	+	3.2219	0.0003	0.0235	0.0662	0.0379	0.1867	0.0471	0.0120	0	0.0242	0
ENSRNOT00000080616	chr13	ENSRNOG00000034164	Cr2	113890272	113927877	-	2.6372	0.0001	0.0114	0.0177	0.0532	0.2861	0.1681	0.0339	0.0057	0.0257	0.0190
ENSRNOT00000080744	chr5	ENSRNOG00000024501	Rgs3	78428669	78567288	+	1.2868	0.0006	0.0341	1.7043	5.4082	8.7042	0.8980	1.7985	1.3464	2.3788	1.3268
ENSRNOT00000080832	chr16	ENSRNOG00000046973	Tmco3	80904125	81072145	+	Inf	0.0002	0.0159	0.3066	0	0	0	0	0	0	0
ENSRNOT00000081247	chr10	ENSRNOG00000019075	Stat5b	88686208	88712309	-	Inf	0.0000	0.0022	0	1.9736	0	0	0	0	0	0
ENSRNOT00000081399	chr18	ENSRNOG00000023433	Gata6	2416871	2446338	+	Inf	0.0006	0.0348	0	0.0192	0.0302	0.0340	0	0	0	0
ENSRNOT00000081620	chr1	ENSRNOG00000055858	Myb	16659442	16687817	-	3.5002	0.0003	0.0193	0.0155	0.2182	0.6306	0.0689	0	0.0234	0.0590	0
ENSRNOT00000081780	chr12	ENSRNOG00000001120	Med13l	43423905	43576804	-	Inf	0.0000	0.0000	1.5824	1.4118	0	0	0	0	0	0
ENSRNOT00000081825	chr5	ENSRNOG00000007490	Gabrr2	48313599	48341642	+	3.2812	0.0002	0.0152	0.1046	0.2535	0.0125	0.1834	0	0	0.0106	0.0464
ENSRNOT00000082005	chr9	ENSRNOG00000014876	Lpin2	119542328	119588715	+	4.1170	0.0011	0.0494	0.0190	0.0068	0.0227	0.0047	0	0.0031	0	0
ENSRNOT00000082164	chr1	ENSRNOG00000015086	Plin1	141458181	141471010	-	3.5362	0.0010	0.0464	0	0.2363	0.0284	0.0797	0.0198	0	0	0.0099
ENSRNOT00000082175	chr4	ENSRNOG00000008775	Lrguk	61420009	61544446	+	4.8809	0.0007	0.0384	0.9405	0	0	0	0	0	0.0166	0.0154
ENSRNOT00000082312	chr4	ENSRNOG00000049593	Wbp11	170772163	170810080	-	Inf	0.0000	0.0000	0	0	10.4330	9.9919	0	0	0	0
ENSRNOT00000082403	chr18	ENSRNOG00000017116	Zfp532	61258911	61368371	+	2.7363	0.0000	0.0005	1.5907	1.1994	1.4427	6.2172	0.2029	1.0981	0.1115	0.1558
ENSRNOT00000082417	chr11	ENSRNOG00000002126	Ncam2	20474483	20656340	+	0.8034	0.0004	0.0240	6.1864	10.6598	11.6681	11.4397	6.1688	4.9137	6.2279	5.5828
ENSRNOT00000082462	chr12	ENSRNOG00000008470	Lrrc43	38507089	38516475	-	2.6203	0.0006	0.0320	0.0729	0.2969	0.1312	0.1007	0.0189	0	0.0790	0
ENSRNOT00000082511	chr10	ENSRNOG00000059827	LOC691995	57669530	57671080	-	0.8309	0.0007	0.0377	55.2812	164.1197	43.5820	77.9246	65.4764	43.2015	37.6333	45.3351
ENSRNOT00000082794	chr3	ENSRNOG00000010477	Pomt1	11254056	11271872	+	1.7529	0.0004	0.0259	0.2148	1.7620	0.4339	1.7094	0.2864	0.3003	0.4188	0.2170
ENSRNOT00000083007	chr13	ENSRNOG00000056580	En1	36532758	36537093	+	3.3579	0.0008	0.0389	0	0.7093	0.1403	2.0128	0.1312	0	0.1480	0
ENSRNOT00000083208	chr6	ENSRNOG00000006985	Ccnk	132090218	132113118	+	1.3568	0.0007	0.0355	1.1498	10.6305	4.1668	2.0199	2.1292	1.8124	1.4832	1.5903
ENSRNOT00000083223	chr20	ENSRNOG00000057125	Ddr1	3552929	3574898	+	2.4736	0.0006	0.0328	0.9853	0.8508	0.5277	0.3898	0	0.4269	0.0276	0.0413

ENSRNOT00000083239	chr4	ENSRNOG00000056052	AABR07060980.1	102147211	102147775	+	Inf	0.0002	0.0137	1.3944	0.0850	0	0	0	0	0	0
ENSRNOT00000083374	chr2	ENSRNOG00000054375	Nhlh2	204427608	204432946	+	1.7857	0.0005	0.0288	0.1213	0.8007	0.9404	1.1881	0.3091	0.2165	0.0475	0.3116
ENSRNOT00000083550	chr1	ENSRNOG00000057578	Prodh2	88955135	88968443	+	Inf	0.0001	0.0097	0	0.2700	0.1898	0	0	0	0	0
ENSRNOT00000083682	chr2	ENSRNOG00000012436	Adh6	243550639	243561757	+	3.2475	0.0003	0.0235	0.0452	0.1689	0.1142	0.0910	0	0	0.0442	0
ENSRNOT00000083702	chr1	ENSRNOG00000019549	Akap12	40879747	40906581	+	1.6520	0.0011	0.0478	1.2893	1.5023	2.0767	1.5397	0.4920	0.0086	0.3070	1.2314
ENSRNOT00000084053	chr20	ENSRNOG00000054549	Lss	12842884	12855891	-	6.3879	0.0000	0.0014	0.0135	1.4406	0.0056	0.0022	0.0090	0.0015	0.0053	0.0017
ENSRNOT00000084381	chr4	ENSRNOG00000061902	Wbp1	114817814	114819848	-	Inf	0.0008	0.0402	0	0	0	0.6459	0	0	0	0
ENSRNOT00000084906	chr12	ENSRNOG00000000986	Camsap3	2140203	2157381	+	8.0920	0.0000	0.0001	0.0381	0.0621	0.0399	0.0534	0	0	0	0.0007
ENSRNOT00000084940	chr7	ENSRNOG00000056487	Oxr1	80625010	80788093	+	5.1493	0.0000	0.0006	0.0340	0.0303	4.2550	0.0111	0.0245	0.0352	0.0360	0.0264
ENSRNOT00000085110	chrX	ENSRNOG00000006967	Xiap	128416809	128452969	+	0.8230	0.0004	0.0272	13.3648	11.4746	13.3979	24.0163	8.9579	8.8861	10.9625	6.3826
ENSRNOT00000085139	chrX	ENSRNOG00000051836	Zfp92	157384608	157392054	-	Inf	0.0000	0.0022	3.6147	0	0	0	0	0	0	0
ENSRNOT00000085530	chr15	ENSRNOG00000007206	LOC361016	17834635	17924302	+	1.0405	0.0001	0.0102	13.7164	19.5447	26.2622	26.7444	15.6055	6.5342	8.4149	11.3859
ENSRNOT00000085933	chr6	ENSRNOG00000019584	Dlk1	133576568	133583751	+	2.0720	0.0000	0.0025	3.9729	38.6272	5.7895	11.7090	6.8617	1.3326	2.8007	3.2981
ENSRNOT00000086247	chr6	ENSRNOG00000014879	Ttc7a	10912488	11013048	+	Inf	0.0003	0.0227	0	0	0	0.2706	0	0	0	0
ENSRNOT00000086425	chr3	ENSRNOG00000020086	Aar2	152626757	152648244	+	5.0195	0.0000	0.0012	0.0222	2.8415	0.0386	0.0406	0.0372	0.0178	0.0210	0.0147
ENSRNOT00000086468	chr14	ENSRNOG00000053766	Ramp3	86922223	86939719	+	0.9562	0.0005	0.0318	8.8433	9.8447	24.0704	19.3432	9.6626	6.8305	7.1970	8.3184
ENSRNOT00000086474	chr14	ENSRNOG00000061527	Gck	86149146	86190659	-	4.3948	0.0001	0.0112	0.0094	0.5723	0.0564	0.0239	0.0203	0.0112	0	0
ENSRNOT00000086514	chr11	ENSRNOG00000058590	AABR07034739.2	86094567	86096397	+	Inf	0.0003	0.0215	0.1746	0	0.3377	0	0	0	0	0
ENSRNOT00000086550	chr7	ENSRNOG00000023781	Plec	117230321	117259791	-	Inf	0.0000	0.0056	0.0029	0	0.0342	0.0014	0	0	0	0
ENSRNOT00000086679	chr18	ENSRNOG00000010440	Gnal	62805410	62944630	+	1.3349	0.0001	0.0086	4.7448	9.8966	13.7173	21.3342	7.7558	3.4154	4.0572	4.4715
ENSRNOT00000086710	chr10	ENSRNOG00000003170	Nlrp3	45893106	45918254	+	6.8942	0.0001	0.0095	0	0.6216	0.0111	0	0	0	0.0053	0
ENSRNOT00000086792	chr7	ENSRNOG00000006103	Tbc1d31	97760134	97839663	+	5.7238	0.0000	0.0020	0.0141	0.9236	0.0083	0.0357	0.0072	0.0113	0	0
ENSRNOT00000086810	chr4	ENSRNOG00000061595	Tfec	44065045	44136815	-	2.4201	0.0004	0.0277	0.1656	0.0468	0.3890	0.0894	0.0313	0.0241	0.0243	0.0494
ENSRNOT00000086919	chr10	ENSRNOG00000018693	Asgr1	56662242	56666086	+	Inf	0.0000	0.0049	0	0.2109	0	0.5769	0	0	0	0
ENSRNOT00000086933	chr3	ENSRNOG00000016708	Necab3	150048341	150064438	-	Inf	0.0006	0.0335	0.0774	0.0102	0	0.0250	0	0	0	0
ENSRNOT00000087038	chr7	ENSRNOG00000051563	Giot1	13104481	13108630	-	Inf	0.0000	0.0028	0.3631	0.7392	0	0	0	0	0	0
ENSRNOT00000087080	chr11	ENSRNOG00000029614	Robo1	9642365	10141977	+	1.7870	0.0006	0.0321	1.5090	0.6445	13.2905	0.4741	1.4808	1.2330	1.1597	0.7393
ENSRNOT00000087094	chr12	ENSRNOG00000001068	Rac1	13097269	13110592	+	3.1521	0.0003	0.0201	0.1046	6.0264	0.2152	0.0495	0.0837	0.1191	0.0904	0.4263

ENSRNOT00000087156	chr10	ENSRNOG00000002314	LOC103689943	28321	68056	+	Inf	0.0000	0.0002	0	0	0	5.5482	0	0	0	0
ENSRNOT00000087376	chr7	ENSRNOG00000006235	Nell2	136527135	136853154	-	Inf	0.0002	0.0174	0.3974	0	0	0	0	0	0	0
ENSRNOT00000087408	chr18	ENSRNOG00000013867	Fgf1	32273770	32359824	+	1.5715	0.0000	0.0005	2.4079	6.9271	6.0693	8.5202	2.7327	2.0614	1.3047	1.9510
ENSRNOT00000087668	chr1	ENSRNOG00000015071	Zim1	70253650	70265524	+	1.7873	0.0003	0.0195	0.7173	3.6654	1.3183	2.0164	1.0609	0.2544	0.3868	0.5336
ENSRNOT00000087669	chr1	ENSRNOG00000053260	Lilrb3a	63759645	64013263	+	4.4116	0.0002	0.0142	0	0.7809	0.7292	0.3164	0	0	0.0858	0
ENSRNOT00000087873	chr16	ENSRNOG00000014363	Arhgef3	2743823	3025019	+	1.9877	0.0008	0.0410	0.1236	0.0513	0.3100	0.3412	0.0782	0.0576	0.0200	0.0525
ENSRNOT00000088070	chr4	ENSRNOG00000032517	Cntn6	136676254	136904224	+	4.9458	0.0001	0.0087	0	0.4340	0.0244	0.2480	0.0229	0	0	0
ENSRNOT00000088198	chr10	ENSRNOG00000061543	Ap2b1	70520206	70621965	+	Inf	0.0000	0.0000	0	35.6027	0	28.8462	0	0	0	0
ENSRNOT00000088416	chr9	ENSRNOG00000056847	Gsta3	27408333	27417023	+	Inf	0.0005	0.0285	0	0	0.0725	0.1951	0	0	0	0
ENSRNOT00000088981	chr15	ENSRNOG00000051706	Tep1	27751180	27798408	-	Inf	0.0000	0.0008	0	0.4053	0.0012	0.0005	0	0	0	0
ENSRNOT00000089138	chr17	ENSRNOG00000019232	RGD1311345	6684621	6690723	+	Inf	0.0001	0.0069	0	0	0.0568	0.3923	0	0	0	0
ENSRNOT00000089149	chr1	ENSRNOG00000048297	LOC108348175	50828134	50931579	+	1.1039	0.0000	0.0050	25.2578	75.5681	80.8483	65.8285	24.0512	45.8477	20.8259	24.4301
ENSRNOT00000089292	chr4	ENSRNOG00000011292	Col1a2	31534225	31568806	+	Inf	0.0000	0.0004	4.4767	0	0	0	0	0	0	0
ENSRNOT00000089392	chr12	ENSRNOG00000050841	Ache	22472358	22478752	-	0.9480	0.0001	0.0118	16.8938	47.6203	22.6770	38.2280	21.7149	12.0307	14.3472	16.9203
ENSRNOT00000089455	chr15	ENSRNOG00000054879	Zic2	108908607	108912737	+	1.0918	0.0001	0.0096	7.2906	8.8608	15.3457	10.5588	5.7947	3.8693	5.1477	4.9194
ENSRNOT00000089456	chr1	ENSRNOG00000016117	Myof	256585898	256734730	-	2.1484	0.0004	0.0256	0.1519	0.1773	1.7023	0.0868	0.0859	0.0929	0.0599	0.2391
ENSRNOT00000089482	chr4	ENSRNOG00000013547	Slc6a12	153921900	153940963	+	Inf	0.0005	0.0283	0	0	0.0647	0.0849	0	0	0	0
ENSRNOT00000089607	chr1	ENSRNOG00000059224	Reps1	13212324	13298347	+	5.1345	0.0000	0.0019	1.7598	0.0383	0.0380	0.0002	0.0244	0.0062	0.0003	0.0214
ENSRNOT00000089635	chr4	ENSRNOG00000051433	LOC103692167	114776797	114779446	+	Inf	0.0000	0.0000	2.6703	2.4036	0	3.7744	0	0	0	0
ENSRNOT00000089807	chr17	ENSRNOG00000051684	St8sia6	80960785	81002838	-	2.1209	0.0001	0.0116	0.6243	1.7709	1.2674	1.5793	0.4692	0.1030	0.2720	0.3609
ENSRNOT00000089952	chr9	ENSRNOG00000014797	Tmbim1	81575197	81586116	-	2.9812	0.0012	0.0496	0.6887	0.0545	0.1363	0.0941	0.0485	0.0171	0	0.0577
ENSRNOT00000090080	chr20	ENSRNOG00000057125	Ddr1	3553455	3575777	+	1.7244	0.0000	0.0051	26.0779	31.8766	39.6900	30.5649	0.8888	20.8680	12.4850	4.5588
ENSRNOT00000090207	chr10	ENSRNOG00000050441	AABR07028989.1	1622573	1674701	+	Inf	0.0000	0.0040	0	0	1.3770	0	0	0	0	0
ENSRNOT00000090211	chr3	ENSRNOG00000008604	Nsfl1c	146981984	147005439	+	5.2622	0.0000	0.0050	1.9308	0.0052	0.0108	0.0126	0.0153	0.0150	0.0084	0.0123
ENSRNOT00000090288	chr8	ENSRNOG00000018184	Tpm1	72815356	72842228	-	Inf	0.0000	0.0001	0.0055	6.1352	0.0044	0.0185	0	0	0	0
ENSRNOT00000090630	chr12	ENSRNOG00000052647	AABR07036505.1	45319501	45320954	+	Inf	0.0000	0.0047	0	0.0798	0.4234	0.1549	0	0	0	0
ENSRNOT00000090745	chr1	ENSRNOG00000061299	LOC100134871	168957460	168958836	+	1.2260	0.0003	0.0213	79.9212	31.8535	129.6209	143.9550	45.5658	80.6014	17.2741	21.2990
ENSRNOT00000090920	chr9	ENSRNOG00000011823	Tfap2b	25410717	25440411	+	3.1666	0.0001	0.0096	0.2868	0.0660	0.1604	2.5231	0	0.1324	0.0697	0.1361

ENSRNOT00000090989	chr3	ENSRNOG00000047374	Gnas	172385672	172433441	+	2.5070	0.0010	0.0467	0.0391	0.7586	0.1318	0.1859	0.1481	0	0.0270	0.0211
ENSRNOT00000091044	chr20	ENSRNOG00000053288	Ank3	20378861	20480623	+	2.1899	0.0003	0.0200	0.0460	0.1016	0.1458	0.2293	0.0169	0.0485	0.0102	0.0390
ENSRNOT00000091150	chr1	ENSRNOG00000016697	Lrrc56	214183724	214197065	+	Inf	0.0001	0.0118	0	0.7514	0	0	0	0	0	0
ENSRNOT00000091292	chr1	ENSRNOG00000016416	Rnh1	214151343	214159157	-	4.5489	0.0000	0.0001	0.9773	0.0549	1.2611	1.2469	0.0145	0.0629	0.0497	0.0241
ENSRNOT00000091359	chr4	ENSRNOG00000008369	Gimap4	78320241	78327026	+	Inf	0.0008	0.0415	0	0	0.1064	0.0622	0	0	0	0
ENSRNOT00000091552	chr4	ENSRNOG00000054251	Clec7a	163216163	163227242	-	2.5751	0.0010	0.0465	0.2658	0.0312	1.7114	0.0597	0.0239	0.0161	0.0359	0.2712
ENSRNOT00000091574	chr1	ENSRNOG00000018454	Apoe	80612895	80615525	-	0.6519	0.0002	0.0162	448.9355	286.9875	511.0985	381.1714	237.5754	216.8826	270.1522	311.6540
ENSRNOT00000092174	chr15	ENSRNOG00000060662	Pou4f1	88620470	88622413	-	5.1877	0.0000	0.0058	0	0.4041	0.1604	1.2976	0.0511	0	0	0
ENSRNOT00000092200	chr8	ENSRNOG00000046261	Acp5	23142734	23146689	-	Inf	0.0000	0.0010	0.9780	0	0.3715	0	0	0	0	0
ENSRNOT00000092517	chr2	ENSRNOG00000019620	Pmf1	187752282	187771857	-	Inf	0.0009	0.0436	0.3788	0	0.1321	0	0	0	0	0
ENSRNOT00000093260	chr15	ENSRNOG00000010479	Mycbp2	87506959	87540378	-	5.3098	0.0000	0.0010	17.6915	0.1420	0.1141	0.1008	0.1135	0.1265	0.1180	0.0970
ENSRNOT00000093432	chrX	ENSRNOG00000007552	Arhgap36	136488691	136491310	+	2.2640	0.0004	0.0248	0.1389	2.6787	0.6694	1.3488	0.3726	0.2259	0.1196	0.2887
ENSRNOT00000093538	chrX	ENSRNOG00000007552	Arhgap36	136460215	136495646	+	2.0778	0.0002	0.0171	0.4411	7.5068	1.3459	2.4105	1.6662	0.1685	0.2331	0.7047
ENSRNOT00000093647	chrX	ENSRNOG00000039323	Dgkk	16442899	16526028	-	2.2327	0.0003	0.0217	0.0961	0.8660	0.3010	0.3608	0.1621	0.0074	0.0506	0.1254

Supplementary Table S5. Details of differentially expressed miRNAs in day-7 and day-28 ICH samples when compared with Sham. log2(FC): The log scale of fold change (the absolute ratio of normalized intensities between ICH and Sham groups). ***P* Value:** Significance index of statistical difference. ***Q* Value:** The corrected *P* Value. **ICH 1 to 4** and **Sham 1 to 4:** Read count after TPM normalization correction of each sample.

Day-7 miRNA	log2(FC)	*P* Value	*Q* Value	ICH1	ICH2	ICH3	ICH4	Sham1	Sham2	Sham3	Sham4
novel_206	-0.8094	0.0016	0.0241	0.8858	1.7143	3.3440	0.2930	6.0861	3.7702	4.5439	1.8452
novel_274	-0.8463	0.0013	0.0212	0.0681	0.0952	0.0458	0.0732	0.1808	0.3927	0.1893	0.5827
novel_289	0.8940	0.0002	0.0068	0.2044	0.6190	0.1374	0.0732	0	0	0	0
novel_310	-0.6930	0.0032	0.0381	0	0	0.0916	0	0.1808	0.2356	0.2840	0.0971
novel_602	-0.7538	0.0007	0.0142	0	0	0	0	0.1808	0.0785	0.2840	0.0971
novel_757	-0.7538	0.0007	0.0142	0	0	0	0	0.1808	0.0785	0.2840	0.0971
novel_768	1.0558	0.0001	0.0031	0.4089	0.3810	0.1374	0.2930	0.0603	0	0	0
rno-let-7a-5p	0.3302	0.0000	0.0000	3632.3680	4226.3554	4351.7696	4267.3494	3814.0130	3441.9137	3539.2140	3193.6204

rno-let-7c-5p	0.3187	0.0037	0.0416	2693.5100	3128.4097	2984.8558	3863.5406	2684.9461	2510.6860	2572.6915	2950.0565
rno-let-7e-5p	0.2248	0.0000	0.0004	1276.5008	1452.5626	1433.9310	1310.5289	1382.8162	1194.1959	1253.7339	1170.0392
rno-let-7f-5p	0.3762	0.0000	0.0000	27839.2739	33312.8462	35055.9317	32874.3381	27259.4610	25620.7792	26216.1404	26430.6686
rno-let-7g-5p	0.3023	0.0001	0.0031	8200.8003	9406.8955	8837.3446	9269.8020	8341.1876	7742.2441	7084.1078	7611.8585
rno-let-7i-5p	0.3265	0.0000	0.0009	7894.8435	9788.6075	8089.8022	9246.4363	7455.4452	7368.1350	7677.1796	7207.8609
rno-miR-106b-3p	-0.3182	0.0001	0.0030	64.5984	91.0947	78.4693	64.8233	113.2261	96.0603	105.9293	84.8783
rno-miR-10b-5p	0.5895	0.0022	0.0304	40.3400	46.0473	83.7830	66.5080	40.0118	60.4010	34.7418	20.7826
rno-miR-124-3p	-0.4768	0.0000	0.0012	2869.9973	3408.5508	3867.6238	2664.2001	5536.3267	4296.3246	6036.7421	3570.2316
rno-miR-129-5p	-0.2724	0.0002	0.0069	5116.0200	6163.0103	5201.8763	4722.2110	7084.6133	6091.7812	8152.2997	6206.7044
rno-miR-132-3p	-0.5500	0.0005	0.0111	843.5958	547.4253	582.1293	534.2610	944.4343	1036.0065	854.7239	1239.3792
rno-miR-132-5p	-0.5181	0.0004	0.0091	1271.0494	987.5655	913.0471	836.7696	1252.7780	1611.7403	1602.7612	1840.7140
rno-miR-134-3p	-0.3925	0.0012	0.0201	7.9045	7.7142	9.4365	5.7132	11.2684	10.4465	10.5077	11.6538
rno-miR-138-2-3p	-0.5743	0.0018	0.0263	1.7035	2.3333	1.9697	1.9044	3.1937	1.8851	4.8279	3.6904
rno-miR-139-3p	-0.5410	0.0000	0.0002	68.0055	80.5233	77.5531	48.7823	115.0941	107.2137	125.7142	87.0149
rno-miR-139-5p	-0.3292	0.0014	0.0221	2604.9938	2855.6494	2233.3281	1955.8318	3362.6755	2845.5227	3404.4120	3435.1449
rno-miR-140-5p	0.3676	0.0001	0.0036	109.6402	104.4279	110.3975	107.4528	84.7840	100.3803	82.3579	87.1120
rno-miR-145-5p	0.5568	0.0005	0.0112	456.4821	312.8553	294.5461	404.7609	232.2972	264.6961	215.7399	312.4183
rno-miR-151-3p	-0.2964	0.0005	0.0116	1321.5425	1619.6093	1244.6519	1292.8032	1624.6343	1844.3115	1993.7247	1754.2819
rno-miR-182	0.9936	0.0000	0.0001	16.8310	22.7618	11.9559	32.0820	12.2325	10.4465	9.5611	8.0605
rno-miR-183-5p	0.7706	0.0008	0.0159	9.5399	18.7142	5.7718	22.1937	11.0273	8.0116	5.1119	5.6327
rno-miR-187-3p	-0.8343	0.0001	0.0027	42.7931	71.2853	63.6733	49.2950	123.1085	97.8669	220.7571	53.0247
rno-miR-18a-3p	-0.7366	0.0042	0.0469	0.1363	0.2857	0.1374	0.0732	0.6026	0.3927	0.3787	0.3885
rno-miR-193b-3p	-0.4721	0.0000	0.0002	30.1187	38.9045	33.7148	30.1044	48.1467	47.2840	48.0895	54.2872
rno-miR-195-3p	0.3791	0.0025	0.0331	51.4471	46.6188	49.2895	62.7724	35.7334	45.7916	41.4630	46.7122
rno-miR-1b	0.7284	0.0000	0.0007	366.1260	284.2840	566.6462	406.4456	197.6485	304.7540	264.8707	226.7631
rno-miR-204-5p	0.8962	0.0000	0.0011	454.3697	1527.8003	1114.1446	1030.8732	557.2723	584.2166	676.4710	279.9820
rno-miR-210-3p	-0.3167	0.0029	0.0361	11.6523	12.1904	12.5056	12.0124	15.6070	19.9504	14.4836	14.7615
rno-miR-212-3p	-0.7068	0.0000	0.0000	95.2623	77.4757	97.9377	70.6830	150.6467	140.5167	149.0963	168.2028

rno-miR-212-5p	-0.5600	0.0022	0.0305	96.6932	78.1900	137.2411	58.4508	132.2678	106.8995	165.1892	208.8939
rno-miR-24-3p	-0.3082	0.0012	0.0205	2317.9804	2201.8914	1952.2496	1876.4325	2460.1812	2899.8757	3011.6499	2766.6066
rno-miR-26b-5p	0.2290	0.0039	0.0439	346.9782	478.9495	410.9445	410.5474	386.0171	376.0727	345.1460	379.6218
rno-miR-3068-3p	0.1580	0.0036	0.0413	302.2771	351.3312	363.6705	335.9091	326.2405	333.5800	365.7828	272.6984
rno-miR-31a-5p	0.2961	0.0013	0.0213	13.7646	16.7142	17.9110	17.9454	16.6314	13.2741	14.9570	12.4307
rno-miR-323-5p	-0.3881	0.0021	0.0294	5.6558	7.0000	6.7796	5.4203	11.7504	8.1687	9.3718	6.1182
rno-miR-329-5p	0.5382	0.0000	0.0019	143.9837	142.7610	151.1667	173.5213	125.0368	99.5163	88.5111	125.4723
rno-miR-335	0.6190	0.0016	0.0240	1.4310	1.7143	2.4736	2.1242	1.6872	1.0211	1.3253	0.8740
rno-miR-337-5p	-0.2748	0.0006	0.0129	66.4383	72.0472	69.9948	52.2249	88.5200	79.3303	87.7538	82.6447
rno-miR-339-5p	-0.3413	0.0021	0.0297	38.4320	40.7140	31.7908	31.7158	44.7119	60.0083	49.8881	41.0796
rno-miR-344b-1-3p	-0.2554	0.0010	0.0184	81.4976	104.7137	116.1693	84.8196	139.5591	114.5184	128.4594	112.3618
rno-miR-34b-3p	0.5870	0.0031	0.0374	6.6779	19.5713	11.4978	11.9392	6.6285	9.7396	11.7384	4.3702
rno-miR-34b-5p	1.0052	0.0001	0.0034	1.0903	4.8571	2.5653	2.7834	1.2654	1.5709	1.2306	0.2913
rno-miR-34c-5p	0.6686	0.0009	0.0178	601.3516	1747.7037	1182.5819	1236.3301	702.9175	910.4133	974.2849	328.1509
rno-miR-3544	-0.4351	0.0028	0.0354	9.1310	10.6666	9.0242	4.8343	12.6543	12.6457	15.1463	9.8086
rno-miR-3553	0.7706	0.0008	0.0159	9.5399	18.7142	5.7718	22.1937	11.0273	8.0116	5.1119	5.6327
rno-miR-3556a	-0.2911	0.0003	0.0084	195.1582	202.0940	196.6084	163.7795	273.0321	227.3873	298.6658	203.4555
rno-miR-3574	-0.3167	0.0029	0.0361	11.6523	12.1904	12.5056	12.0124	15.6070	19.9504	14.4836	14.7615
rno-miR-3596a	0.3299	0.0000	0.0000	3626.6441	4220.1650	4345.9977	4259.8050	3810.5180	3436.8083	3535.4274	3188.0849
rno-miR-3596c	0.2250	0.0000	0.0004	1270.2999	1447.8484	1429.3502	1304.8157	1377.9955	1189.3261	1249.5687	1163.9210
rno-miR-3596d	0.3690	0.0000	0.0000	26762.3605	32239.2336	34000.5131	31622.4065	26478.9303	24785.7689	25532.0015	25598.6861
rno-miR-374-3p	0.3018	0.0012	0.0202	166.5386	176.7608	220.6576	201.1353	147.4530	172.4059	195.0084	145.6722
rno-miR-376c-3p	0.3192	0.0026	0.0334	12.8107	13.4761	13.6050	12.3054	9.7016	13.5097	10.9811	10.0999
rno-miR-383-5p	-0.3380	0.0024	0.0324	644.9624	689.3292	694.2676	440.0658	921.5963	736.4365	808.5277	887.7264
rno-miR-384-3p	0.2514	0.0025	0.0327	129.6739	142.9039	143.2877	156.4548	113.9492	134.8615	143.2271	120.3252
rno-miR-412-3p	-0.6635	0.0003	0.0084	2.1805	4.0476	3.6646	2.0509	6.2669	3.2989	6.8158	5.4384
rno-miR-431	-0.4427	0.0018	0.0266	28.8921	35.9045	31.8824	20.2161	45.1940	39.2724	61.8158	29.5229
rno-miR-450a-5p	0.3872	0.0000	0.0011	87.1534	116.5231	100.8694	100.2014	72.4912	79.3303	97.8829	78.5659

rno-miR-450b-5p	0.3650	0.0003	0.0076	84.4277	115.0469	97.7087	87.6030	66.3448	76.3456	98.4509	74.8755
rno-miR-455-5p	0.3620	0.0014	0.0220	34.7523	43.4283	39.7614	41.7506	25.9715	39.9793	35.9724	29.1344
rno-miR-463-3p	-0.7082	0.0035	0.0406	0	0.0476	0.1832	0.0732	1.2052	0.0785	0.0947	0.3885
rno-miR-483-3p	0.9527	0.0003	0.0081	0.2044	0.9048	0.1374	0.8057	0.1808	0.0785	0	0
rno-miR-499-5p	0.4051	0.0001	0.0027	105.8924	167.5228	161.5194	136.8980	109.6105	101.9512	130.4474	112.4589
rno-miR-543-5p	-0.6019	0.0000	0.0003	3.8841	5.0476	3.8937	3.8088	7.8939	6.2050	7.1945	6.3125
rno-miR-667-5p	0.3592	0.0011	0.0187	18.2620	26.2379	29.1340	20.8753	23.6817	18.9293	19.9742	15.1499
rno-miR-674-3p	-0.5129	0.0001	0.0028	326.3993	307.7124	247.6386	207.4345	364.3843	438.9086	490.1717	406.7168
rno-miR-674-5p	-0.3986	0.0008	0.0163	15.5363	12.8094	12.2308	12.6717	16.7519	18.6937	20.2582	20.5883
rno-miR-741-3p	-0.8732	0.0005	0.0120	1.0221	1.1905	3.6188	0.4395	5.7848	3.6131	4.0706	2.9134
rno-miR-743b-5p	-1.1421	0.0000	0.0016	0.1363	0.1905	0.3207	0.1465	0.6628	1.2567	0.5680	0.6798
rno-miR-7a-5p	0.7314	0.0001	0.0030	26916.3610	20258.9251	26589.9534	21129.8983	24763.2451	11000.4414	13213.8102	9031.8712
rno-miR-7b	0.7149	0.0000	0.0012	2772.8952	2252.1292	2863.4644	2441.9698	2616.4922	1234.9606	1457.4515	1170.1363
rno-miR-874-3p	-0.3540	0.0003	0.0077	21.4647	20.0951	18.5065	16.9200	30.1293	23.7991	26.5060	25.7354
rno-miR-98-5p	0.3291	0.0010	0.0184	67.3923	88.7137	75.8582	87.0903	82.2531	55.1385	67.9690	64.0958
rno-miR-9a-3p	0.2420	0.0033	0.0389	1267.5061	1324.2777	1488.2594	1210.8402	1041.9931	1252.7118	1237.8303	1207.1370

**Day-28 miRNA**	**log2(FC)**	***P* Value**	***Q* Value**	**ICH1**	**ICH2**	**ICH3**	**ICH4**	**Sham1**	**Sham2**	**Sham3**	**Sham4**
novel_518	-0.6262	0.0016	0.0407	0	0	0	0	0.2221	0.1756	0.1016	0
novel_552	-0.6307	0.0016	0.0394	0	0	0	0	0	0.2341	0.2541	0.0545
novel_645	0.7288	0.0008	0.0263	0.0905	0.1497	0.1505	0.1160	0	0	0	0
rno-let-7a-1-3p	0.4283	0.0013	0.0341	14.8391	18.0613	22.4698	18.2687	10.3283	14.3378	10.8761	15.1025
rno-miR-10b-5p	0.8829	0.0006	0.0213	7.6910	83.0222	43.3346	196.3740	34.9275	5.5596	3.6084	15.5387
rno-miR-1224	-0.5056	0.0012	0.0336	11.6055	11.6458	8.7415	14.2302	12.6675	20.6059	13.6925	22.3864
rno-miR-130b-5p	0.8146	0.0001	0.0077	1.1310	2.7441	2.5579	2.1458	0.7774	0.9363	1.1689	1.1450
rno-miR-133a-5p	0.8046	0.0007	0.0235	0.0905	0.0998	0.3511	0.2320	0	0	0.0508	0
rno-miR-135a-3p	0.6642	0.0000	0.0010	27.4613	49.1447	37.3660	41.8730	20.7122	21.8286	19.4651	28.1332
rno-miR-135a-5p	0.7201	0.0001	0.0098	298.4553	881.8114	350.2375	526.0806	338.6686	275.5783	233.3780	244.0934
rno-miR-137-3p	-0.4616	0.0004	0.0176	132.6920	124.2339	86.0672	154.2690	195.2939	141.1542	167.1052	165.1461

rno-miR-138-5p	-0.4678	0.0005	0.0199	297.4600	233.3003	204.1339	252.9200	356.8820	382.5559	317.3372	281.1136
rno-miR-16-3p	0.9694	0.0002	0.0131	0.2714	0.3493	0.6019	1.1019	0.1666	0.1170	0.2541	0.0545
rno-miR-200a-3p	0.7158	0.0017	0.0408	1.9454	1.9458	1.5047	0.5800	0.9995	0.7608	0.5082	0.7088
rno-miR-216b-5p	1.3399	0.0000	0.0006	0.1357	1.8460	0.6019	1.7399	0.2776	0	0.1016	0.1090
rno-miR-217-5p	1.3314	0.0000	0.0001	4.2979	26.9423	11.0343	13.8030	5.3863	1.7556	3.7101	4.3617
rno-miR-218a-5p	-0.5555	0.0000	0.0048	8885.5262	7578.6189	6310.1938	11154.8104	13104.1278	9068.8624	13030.8479	13614.6018
rno-miR-218b	-0.5691	0.0000	0.0059	7942.4762	6355.0893	5585.1926	10377.2017	11959.4622	8038.8232	11623.3122	12450.2919
rno-miR-224-5p	1.1160	0.0000	0.0024	1.1763	4.2908	2.8087	1.1019	0.6108	0.2926	0.8640	1.0904
rno-miR-298-3p	0.7898	0.0019	0.0434	0.1810	1.1974	0.7523	0.5800	0.3332	0.1756	0.0508	0.3817
rno-miR-298-5p	1.2203	0.0000	0.0006	8.2791	55.5311	21.0654	16.1229	11.9386	4.9158	5.6921	7.5785
rno-miR-29a-5p	-0.4101	0.0011	0.0312	8.0529	6.5859	6.5202	7.1915	11.2723	7.9004	8.5890	9.1596
rno-miR-3065-3p	0.4154	0.0003	0.0151	99.8470	118.0971	173.3382	152.2972	101.5062	81.2865	95.5976	100.9196
rno-miR-30b-3p	0.7723	0.0003	0.0156	1.0858	1.1974	1.1034	1.2759	0.7219	0.6437	0.4574	0.4362
rno-miR-320-3p	0.2780	0.0018	0.0414	225.6624	254.2056	246.7662	292.2412	237.4956	190.4879	187.0277	178.1222
rno-miR-338-5p	0.4057	0.0004	0.0161	105.2307	124.8825	180.2597	157.6908	106.7814	85.2660	101.3914	106.1537
rno-miR-3547	1.0579	0.0001	0.0068	0.2262	3.6422	1.4044	1.7979	0.9440	0	0.1525	0.1636
rno-miR-3552	0.8181	0.0007	0.0230	0.5881	1.5966	1.6050	2.1458	0.8329	0.4682	0.2033	0.9814
rno-miR-3560	0.6770	0.0001	0.0079	3.8455	7.4341	6.3196	4.3497	2.7764	2.9261	3.0494	3.5439
rno-miR-374-3p	-0.3146	0.0009	0.0274	205.5302	188.5961	176.4980	208.9591	254.8205	229.0536	227.6350	220.7582
rno-miR-433-5p	0.4961	0.0003	0.0145	98.8970	191.0408	157.0878	132.8106	99.7293	94.1613	91.7859	95.0858
rno-miR-452-5p	0.8211	0.0014	0.0364	0.4072	1.9957	1.0533	0.6960	0.9440	0.0585	0.1016	0.2181
rno-miR-489-3p	0.7566	0.0022	0.0492	0.3167	2.5944	0.5517	1.1019	0.9995	0	0.1016	0.0545
rno-miR-501-3p	0.6770	0.0001	0.0079	3.8455	7.4341	6.3196	4.3497	2.7764	2.9261	3.0494	3.5439
rno-miR-551b-5p	-0.9441	0.0002	0.0112	0.3167	0.2994	0.4012	0.2900	0.2776	0.8778	1.0673	1.1450
rno-miR-653-3p	1.0478	0.0001	0.0059	0.1357	2.1953	0.7022	0.4060	0.3332	0	0.0508	0
rno-miR-666-5p	0.8449	0.0003	0.0142	7.8719	35.6237	12.8900	29.9839	15.9922	4.1550	7.2168	10.0320
rno-miR-673-5p	-0.6964	0.0005	0.0206	1.0405	1.0478	0.9530	0.8119	2.4433	1.2290	1.2197	1.8537
rno-miR-708-5p	-0.4074	0.0009	0.0267	39.3597	29.7363	34.4068	54.1102	50.1423	43.7157	50.3145	56.8661

Supplementary Table S6. Primers for qRT-PCR verification.

Day 7			Day 28		
**Name**	**Primer Sequence (5'→3')**		**Name**	**Primer Sequence (5'→3')**	
U6	RT	CGCTTCACGAATTTGCGTGTC	U6	RT	CGCTTCACGAATTTGCGTGTC
	Forward	TCGCTTCGGCAGCACATATAC		Forward	TCGCTTCGGCAGCACATATAC
	Reverse	GTCGTATCCAGTGCAGGGT		Reverse	GTCGTATCCAGTGCAGGGT
rno-miR-134-3p	RT	GTCGTATCCAGTGCAGGGTCCGAGGTATTCGCACTGGATACGACTTGGTG	rno-miR-1224	RT	GTCGTATCCAGTGCAGGGTCCGAGGTATTCGCACTGGATACGACCTCCAC
	Forward	AACAATCTGTGGGCCACCTA		Forward	AACTTCGTGAGGACTGGGG
	Reverse	GTCGTATCCAGTGCAGGGT		Reverse	GTCGTATCCAGTGCAGGGT
rno-miR-145-5p	RT	GTCGTATCCAGTGCAGGGTCCGAGGTATTCGCACTGGATACGACAGGGAT	rno-miR-298-5p	RT	GTCGTATCCAGTGCAGGGTCCGAGGTATTCGCACTGGATACGACGGGAAG
	Forward	AACAAGGTCCAGTTTTCCCAG		Forward	AAGAATGGCAGAGGAGGGC
	Reverse	GTCGTATCCAGTGCAGGGT		Reverse	GTCGTATCCAGTGCAGGGT
rno-miR-212-3p	RT	GTCGTATCCAGTGCAGGGTCCGAGGTATTCGCACTGGATACGACTGGCCG	rno-miR-551b-5p	RT	GTCGTATCCAGTGCAGGGTCCGAGGTATTCGCACTGGATACGACAGGTCT
	Forward	AACGGCTAACAGTCTCCAGT		Forward	AACCGGGAAATCAAGCTTGG
	Reverse	GTCGTATCCAGTGCAGGGT		Reverse	GTCGTATCCAGTGCAGGGT
rno-miR-667-5p	RT	GTCGTATCCAGTGCAGGGTCCGAGGTATTCGCACTGGATACGACGTGCTC	rno-miR-673-5p	RT	GTCGTATCCAGTGCAGGGTCCGAGGTATTCGCACTGGATACGACCTCCAA
	Forward	AATATGCGGTGCTGGTGGA		Forward	AACTTCCTCACAGCTCCGG
	Reverse	GTCGTATCCAGTGCAGGGT		Reverse	GTCGTATCCAGTGCAGGGT
GAPDH	Forward	CCCCAATGTATCCGTTGTG	GAPDH	Forward	CCCCAATGTATCCGTTGTG
	Reverse	CTCAGTGTAGCCCAGGATGC		Reverse	CTCAGTGTAGCCCAGGATGC
novel_circ_0002488	Forward	GCCTACTTTGCGGCTGTTCT	novel_circ_0001449	Forward	TTGAAGAACACCTGGCCGAAG
	Reverse	TGTTTCCAAATCCTCTTCCTTCGT		Reverse	TCCACAGGCGTCTTCTCACTA
novel_circ_0004272	Forward	GCCCACACCTTCAGGAAAACT	novel_circ_0019710	Forward	CTGCTCACCCACCTGTACCA
	Reverse	AAGCACAAGCAGATCAGGGA		Reverse	GGCCCCCAGTATCTGCTCTT
novel_circ_0007008	Forward	TAATCGCGCATCCACGGAGA	novel_circ_0019788	Forward	TCTTCCCTGGTCATTGCGTC
	Reverse	GAGCAGGATAATTTGTGGAAGCC		Reverse	GCTGTATGCTGCCAAACTCCA

novel_circ_0017497	Forward	TTGAAGCGATCACCGTCAGC
	Reverse	AGACGGGGTTCTCCAATCTCA
ENSRNOT00000005016	Forward	GTGAGGGACGACAGATTCTCC
	Reverse	TGAGCATCCACAAACCCGAA
ENSRNOT00000020926	Forward	CACCTACAACAGCCCCCACTG
	Reverse	GGCCCTGGTCCTTGCTAAAG
ENSRNOT00000086397	Forward	CCCAGTATCCGGAGTGTGATG
	Reverse	ATCTTTAAATAGCAGGAAGCCGA
ENSRNOT00000088441	Forward	GCTGTCTCCTGTCCTGTGTCAG
	Reverse	TTGAAGATCCTGGGATTCCCTTCTG
novel_circ_0020253	Forward	AGCAGTTGGCAAAACGCATA
	Reverse	GGGCCAGTGGAAAGGGAAAAT
ENSRNOT00000006128	Forward	GGAGCAGATCAGAAAGGATTACAC
	Reverse	GGTTTGTCACAGAAAAGAGGCA
ENSRNOT00000028653	Forward	GCTGCACAAGAGAATGCCTCG
	Reverse	AGGTCCATCAACCTTACCAGC
ENSRNOT00000042790	Forward	CAGAAAGCCCATGAAGTGGTGA
	Reverse	GCATTAGGGGCACACAGGAC
ENSRNOT00000080188	Forward	GAGACCCACAGTTATGCCGC
	Reverse	CCGGATGTAGGCATGGAAGG
